# Quantitative Proteomics Reveals the Role of Lysine 2-Hydroxyisobutyrylation Pathway Mediated by Tip60

**DOI:** 10.1155/2022/4571319

**Published:** 2022-02-08

**Authors:** Ning Wang, Yue Jiang, Ping Peng, Guobin Liu, Shankang Qi, Kun Liu, Qi Mei, Jian Li

**Affiliations:** ^1^Department of Neurosurgery, The First Affiliated Hospital of Xi'an Jiaotong University, Xi'an 710061, China; ^2^School of Mechanical Engineering and Automation, Northeastern University, Shenyang 110819, China; ^3^Shanghai Institute of Materia Medica, Chinese Academy of Sciences, Shanghai 201203, China; ^4^Department of Oncology, Tongji Hospital, Tongji Medical College, Huazhong University of Science and Technology, Wuhan 430030, China; ^5^School of Chinese Materia Medica, Nanjing University of Chinese Medicine, Nanjing 210023, China; ^6^Institute of Molecular Medicine and Experimental Immunology, University Clinic of Rheinische Friedrich-Wilhelms-University, Germany

## Abstract

Lysine 2-hydroxyisobutyrylation (Khib) is a new type of posttranslational modifications (PTMs) extensively reported on eukaryotic cell histones. It is evolutionarily conserved and participates in diverse important biological processes, such as transcription and cell metabolism. Recently, it has been demonstrated that Khib can be regulated by p300 and Tip60. Although the specific Khib substrates mediated by p300 have been revealed, how Tip60 regulates diverse cellular processes through the Khib pathway and the different roles between Tip60 and p300 in regulating Khib remain largely unknown, which prevents us from understanding how this modification executes its biological functions. In this study, we report the first Khib proteome mediated by Tip60. In total, 3502 unique Khib sites from 1050 proteins were identified. Among them, 536 Khib sites from 406 proteins were present only in Tip60 overexpressing cells and 13 Khib sites increased more than 2-fold in response to Tip60 overexpression, indicating that Tip60 significantly affected global Khib. Notably, only 5 of the 549 Tip60-targeted Khib sites overlapped with the 149 known Khib sites targeted by p300, indicating the different Khib substrate preferences of Tip60 and p300. In addition, the Khib substrates regulated by Tip60 are deeply involved in processes such as nucleic acid metabolism and translation, and some are associated with Parkinson's and Prion diseases. In summary, our research reveals the Khib substrates targeted by Tip60, which elucidates the effect of Tip60 in regulating various cellular processes through the Khib pathway, and proposes novel views into the functional mechanism of Tip60.

## 1. Introduction

Protein posttranslational modifications (PTMs) play crucial roles in multiple biological processes such as transcriptional regulation and signal transduction [1–3]. Many studies have demonstrated that aberrant regulation of PTMs is linked to diverse diseases [4–7]. Lysine 2-hydroxyisobutyrylation (Khib) is a new type of PTMs extensively reported on eukaryotic cell histones and is evolutionarily conserved [8]. Cellular levels of 2-hydroxyisobutyrate, the corresponding short-chain fatty acid precursor of Khib, possibly modify environmental implications on biological processes and the epigenome by the Khib pathway [9, 10]. The chemical structure of Khib is distinctive; thus, it exhibits different characteristics from the well-studied lysine methylation (Kme) and acetylation (Kac) markers. This modification adds the enormous hydrophilic 2-hydroxyisobutyryl groups to the electropositive lysine side chains, thus being able to interfere with cofactor binding or bring conformational variations that cause changed turnover and substrate binding. Emerging evidence has shown that Khib has diverse cellular functions, such as transcriptional and metabolic regulation. For example, in colon tumor cells, Khib has been verified to regulate the catalytic activities of glycolytic enzymes [10]. In addition, mutation of H4K8hib (K8A) causes expression level changes in genes associated with carbon transport and carbon metabolism [11].

The levels of lysine acylations in cells are dynamic and modified by the opposite effect of two enzyme families, lysine acetyltransferase (KAT), and lysine deacetylase (KDAC) [12, 13]. For example, H4K8hib is regulated by KDAC Hos3p and Rpd3p in Saccharomyces cerevisiae, while HDAC2 and HDAC3 are the main enzymes that erase Khib in mammalian cells [11]. On the other hand, Esa1p in budding yeast and Tip60/p300 in mammalian cells can write Khib to the substrate protein [9]. A quantitative Khib proteomics study revealed that p300 can regulate glycolysis through the Khib on a key glycolytic enzyme ENO1. Interestingly, p300 can selectively catalyze Khib or Kac on distinct protein substrates, thereby mediating different downstream biological processes [10].

Despite the above progress, how Tip60 regulates cellular processes through the Khib pathway and the different roles of Tip60 and p300 in regulating Khib remain largely unknown, which prevents us from understanding how this modification executes its biological functions. Therefore, to reveal the role of the Khib pathway mediated by Tip60, we report a quantitative proteomics study in which candidate Khib substrates regulated by Tip60 were identified.

In our study, the global Khib proteome from wild type (WT) and Tip60 overexpressing (OE) cells was quantified using Stable Isotope Labeling by/with Amino acids in Cell culture (SILAC) and mass spectrometry. In total, we determined 3502 unique Khib sites from 1050 proteins, where 536 Khib sites from 406 proteins were only present in Tip60 overexpressing cells and 13 Khib sites increased more than 2-fold in response to Tip60 overexpression, indicating that Tip60 significantly affected global Khib. Interestingly, only 5 out of the 549 Khib sites regulated by Tip60 overlapped with 149 previously reported p300-regulated Khib sites, suggesting that Tip60 and p300 have a different substrate preference for Khib. Pathway analysis of Tip60-targeted Khib substrates revealed significant roles of Tip60 in mediating nucleic acid metabolism and translation and Parkinson's and Prion diseases, which suggests the potential connection of Tip60 to a variety of biological processes and diseases by the regulation of Khib.

## 2. Methods

### 2.1. Materials

Tip60 was cloned into pcDNA3.0 with a Flag tag. Pan-Khib antibody (PTM Biolabs, China, catalog no. PTM-801, PTM-1204), PARK7 antibody (Cell Signaling Technology, catalog no. 12255), HEK293 cell line (National Collection of Authenticated Cell Cultures, catalog no. GNHu 43), L-Lysine HCl (Lys0) (J&K, catalog no. 611898), and ^13^C_6_^14^N_2_-L-Lysine (Lys8) (Silantes, catalog no. 211604102) were purchased and used without further authentication. Trypsin was obtained from Promega (USA). C18 ZipTips were purchased from Millipore Corporation (USA).

### 2.2. Stable Isotope Labeling of Cells and Transfections

HEK293 cells were cultured in SILAC DMEM containing 10% FBS and isotopically enriched forms of “heavy” (^13^C_6_^15^N_2_-L-Lysine) or “light” (^12^C_6_^14^N_2_-L-Lysine) lysine (100 mg/L). Before being collected, cells were cultured for more than seven generations and analyzed by LC-MS/MS to ensure complete incorporation of the isotopic label (labeling efficiency higher than 98%). Transient overexpression transfection was carried out with Lipofectamine 2000 (Invitrogen) essentially followed by product manuals. HEK293 cells were grown in DMEM (containing 10% FBS, 1% P/S) at 37°C in a 5% CO_2_ incubator.

### 2.3. Protein Extraction and Trypsin Digestion

A total of 2 × 10^7^ cells were washed with ice-cold phosphate-buffered saline (PBS) twice. Then, the cells were lysed in buffer (8 M urea, 3 *μ*M Trichostatin A, 2 mM EDTA, 5 mM DTT, 50 mM Nicotinamide, and 1% Protease inhibitor cocktail (Roche, Switzerland)) and sonicated in a ultrasonic processor (JY96-IIN, Jingxin Technology) on ice. The lysate supernatant was collected after centrifugation (16,100 × g) at 4°C for 15 min, and equal volumes of proteins from Tip60 OE HEK293 and WT cells were mixed.

The 2 mg mixed proteins were subjected to reduction by reacting with 10 mM DTT at 37°C for 1 h, followed by alkylation using 20 mM iodoacetamide at 25°C for 45 min in the dark, and the reaction was ended using 20 mM cysteine. The urea concentration was reduced to 2 M using 100 mM NH_4_HCO_3_. The first digestion was performed by adding trypsin at 1/50 of the weight of the substrate overnight at 37°C. The generated proteolytic peptides were dried in SpeedVac (ThermoFisher Scientific).

### 2.4. Immunoaffinity Enrichment

Khib peptides were immunoaffinity enriched as previously described [9]. Briefly, the peptides obtained from above were resuspended in NTEN buffer (100 mM NaCl, 50 mM Tris-HCl, 1 mM EDTA, and 0.5% Nonidet P-40) and incubated with 30 *μ*L of pan anti-Khib beads (PTM Biolabs, China) with tender shaking overnight at 4°C. The beads were washed four times with NTEN buffer and twice with ddH_2_O. Peptides bonded on the beads were eluted by 0.1% trifluoroacetic acid, vacuum-dried and desalted using C18 ZipTip (Millipore Corporation, USA).

### 2.5. LC-MS/MS Analysis

The Khib peptides collected above were analyzed on a Q-Exactive mass spectrometer (ThermoFisher Scientific, MA) connected to an EASY-nLC 1000 UHPLC (ThermoFisher Scientific, MA). Peptides in 0.1% formic acid (FA) solution were separated on a reversed-phase HPLC column (75 *μ*m inner diameter with 10 cm length), which was filled with Reprosil 100 C18 resin (3 *μ*m particle size). The HPLC mobile phase consisted of a gradient of 2-90% buffer B (0.1% (v/v) FA in 90% acetonitrile) in buffer A (0.1% (v/v) FA in water). The samples were eluted at a velocity of 200 nL/min over 60 min. The mass spectrometer was worked in data-dependent acquisition (DDA) mode that alternated between once full mass scan at 70,000 mass resolution; then, the top 15 most intense precursor ions were implemented with 25 second-dynamic exclusions. MS/MS fragmentation was carried out with the top 15 most intense precursor ions using higher-energy collision dissociation (HCD), with a normalized collision energy (NCE) of 27%, and then analyzed with 17,500 resolution in the Orbitrap. The AGC numbers were 3e6 and 1e5 for MS1 and MS2, respectively. The isolation window was set to 1.5 m/z.

### 2.6. Database Search and Data Filter Criteria

The experimental MS/MS results were searched with the Swiss-Prot human database (20368 entries, https://www.uniprot.org) using MaxQuant (v.1.6.15.0) and the integrated Andromeda search engine. Enzyme specificity was set as full cleavage by trypsin, and two missing cleavages were allowed at most. Acetylation on protein N-terminal, oxidation on methionine, and 2-hydroxyisobutyrylation on lysine were set as dynamic modifications. Carbamidomethylation on cysteine was set as a fixed modification. The approximated FDR thresholds of modification sites, peptides, and proteins were set to 1%, and the modified peptides with PTM score of more than 40 were chosen for additional bioinformatics analysis. The quality of 100 randomly selected MS/MS spectra of Khib peptides and additional 40 randomly selected MS/MS spectra of Tip60-mediated Khib peptides were manually checked.

### 2.7. Bioinformatics Analysis

Pathway analysis was carried out with a hypergeometric test in the clusterProfiler package in R [14]. The protein-protein interaction network of Tip60-targeted Khib substrates was established based on the STRING database (v11, http://www.string-db.org/) [15] and visualized in Cytoscape (v.3.8.2) [16].

## 3. Results

### 3.1. Characterization of the Khib Proteome in Response to Tip60 OE

**Figure 1:**
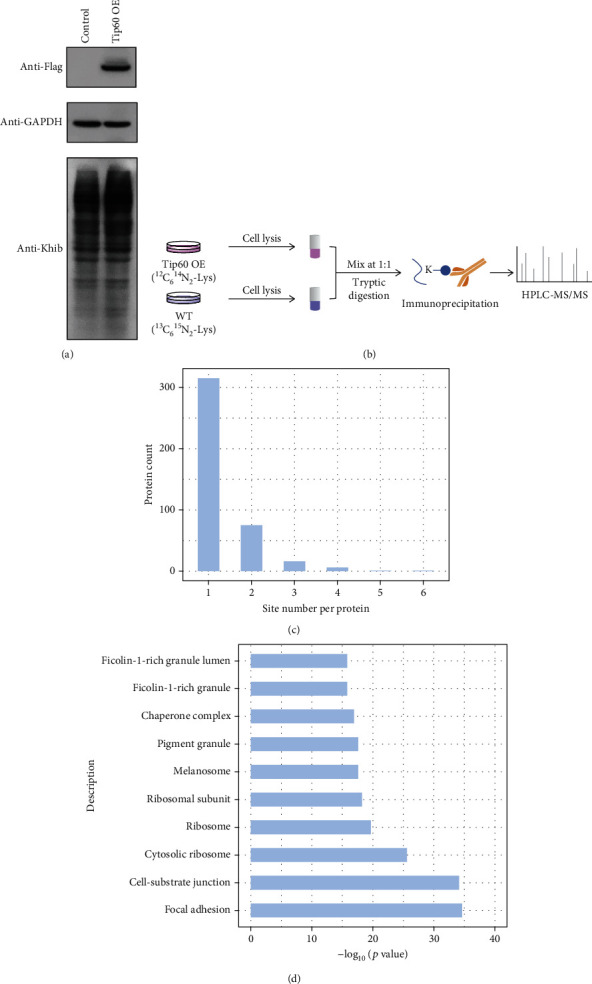
Profiling of Khib proteome. (a) Validation of the Khib dynamics in response to Tip60 overexpression. (b) Schematic diagram of the protocol for identifying and quantifying Khib in WT and Tip60 OE cells. (c) Distribution of the amounts of Tip60-targeted Khib sites of each protein. (d) Cellular compartment distribution of Tip60-targeted Khib proteins.

Given that Tip60 can catalyze Khib reactions, we sought to identify the candidate Khib substrates regulated by Tip60. Indeed, overexpression of Tip60 in HEK293 cells substantially increased Khib levels globally (Figure 1(a)), verifying the significance of Tip60 in regulating Khib modification on a variety of proteins. To determine the dynamics of Khib sites in response to Tip60, we quantified a global Khib proteome between WT and Tip60 OE cells using SILAC and mass spectrometry (Figure 1(b) and Figure S1). Tip60 OE cells and WT HEK293 cells were metabolically labeled with “light” lysine (^12^C_6_^14^N_2_-Lys) and “heavy” lysine (^13^C_6_^15^N_2_-Lys), respectively (Figure 1(b)). Equal volumes of proteins from “light” and “heavy” cell lysates were mixed and digested with trypsin. Then, the Khib-containing peptides were captured with anti-Khib antibody and identified by HPLC-MS/MS. The mass data were searched by MaxQuant (v.1.6.15.0) with a total FDR of less than 1%. To enhance the credibility of the identified Khib peptides, we deleted those peptides with scores lower than 40 and localization probability lower than 0.75. In addition, the proteins in the SILAC sample were quantified, and the ratios (WT to OE) of all quantifiable Khib sites were normalized by quantified protein expression level.

In total, we obtained 3502 unique Khib sites from 1050 proteins, where 536 Khib sites were only detected in Tip60 OE cells and the abundance of 13 Khib sites increased more than 2-fold (−log_2_(WT/OE) > 1) in response to Tip60 OE (Table S1). Among these potential Tip60-targeted Khib proteins, 315 (76%) had a single Tip60-targeted Khib site, while the others contained 2-7 Tip60-targeted Khib sites (Figure 1(c) and Table S1).

To describe the subcellular location of Tip60-targeted Khib proteins, we conducted cellular component analysis for Tip60-targeted Khib proteins by Gene Ontology (GO) databases. A significant number of Tip60-targeted Khib proteins were annotated in focal adhesion, cell-substrate junction, and cytosolic ribosome (Figure 1(d)). The diverse distribution of Tip60-targeted Khib proteins suggests that the main functions of the Tip60-mediated Khib pathway may be widely present in different subcellular compartments.

### 3.2. Quantitative Analysis of the Tip60-Targeted Khib Proteome

**Figure 2:**
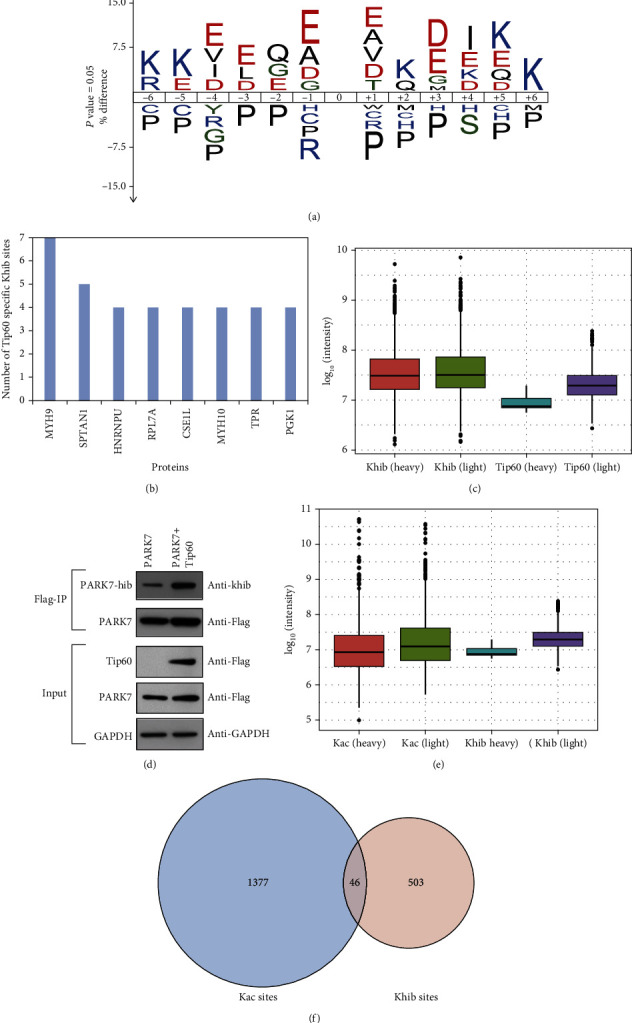
Characteristics of the Tip60-targeted Khib proteome. (a) Consensus sequence logo plots illustrate a representative sequence of Tip60-targeted Khib sites. (b) Distribution of amounts of Tip60-targeted Khib sites on various enzymes. (c) Abundance distribution of global and Tip60-targeted Khib peptides. (d) Western blot validation of the Khib dynamics on PARK7 in response to Tip60 OE. (e) Abundance distribution of Tip60-targeted Kac and Khib peptides. (f) Overlaps between Tip60-targeted Kac and Khib sites.

To explore whether Tip60-targeted Khib peptides have common sequence motifs, we used IceLogo to compare the amino-acid sequences around Khib sites and human background sequences. The results showed that the electronegative amino acid (glutamic acid) was enriched at many positions, the electropositive amino acid (lysine) was enriched at the -6, -5, +2, +5, and +6 positions, and proline was depleted at most positions (Figure 2(a)). The unique flanking sequence features of Tip60-targeted Khib peptides indicate that Tip60 may have a unique substrate preference for Khib. Consistently, although many proteins contain more than 10 Khib sites, only one or a few Khib sites were mediated by Tip60 (Table S1). For example, 20 Khib sites were identified on heterogeneous nuclear ribonucleoprotein U (HNRNPU); however, there were only 4 Tip60-targeted sites (Figure 2(b) and Table S1).

Next, to investigate whether the Tip60-targeted Khib peptides are enriched from high-abundant peptides, we compared the abundance distribution of global Khib peptides with Tip60-targeted Khib peptides. The results show that the abundance of Tip60-targeted Khib peptides is distributed by two orders of magnitude, and that the median abundance of the Tip60-targeted Khib peptides is lower than that of the global Khib peptides, indicating that the screening is not biased toward high-abundance proteins (Figure 2(c) and Table S1).

Among the Tip60-targeted Khib substrates, K148hib on Parkinson disease protein 7 (PARK7) was identified in Tip60 OE cells, while it could not be detected in WT cells, suggesting that the function of PARK7 may be affected at the posttranslational level, potentially by Tip60-targeted Khib. To confirm the mass data, we performed a western blot verification. Flag-tagged PARK7 in combination with either the vector or Tip60 plasmids was cotransfected into 293T cells. After incubation, the immunoprecipitated Flag-tagged PARK7 was analyzed using anti-Flag and anti-Khib antibodies. The results showed that overexpression of Tip60 obviously increased Khib levels on PARK7, while there was almost no change in PARK7 expression (Figure 2(d)), which confirmed our quantitative Khib proteome results.

Because Tip60 is a well-known lysine acetyltransferase, we next compared Tip60-mediated Kac and Khib sites. As expected, Tip60 OE increased the abundance of Kac (Figure 2(e)). Interestingly, only 46 out of the 549 Tip60-mediated Khib sites overlapped with the Tip60-mediated Kac sites (Figure 2(f) and Table S1, Table S2), indicating that Tip60 has different substrate preferences for Khib and Kac. This result is not surprising because a similar phenomenon was also observed for p300-mediated Khib and Kac sites [10].

### 3.3. Cellular Pathway Analysis and Functional Annotation of the Tip60-Targeted Khib Proteome

**Figure 3:**
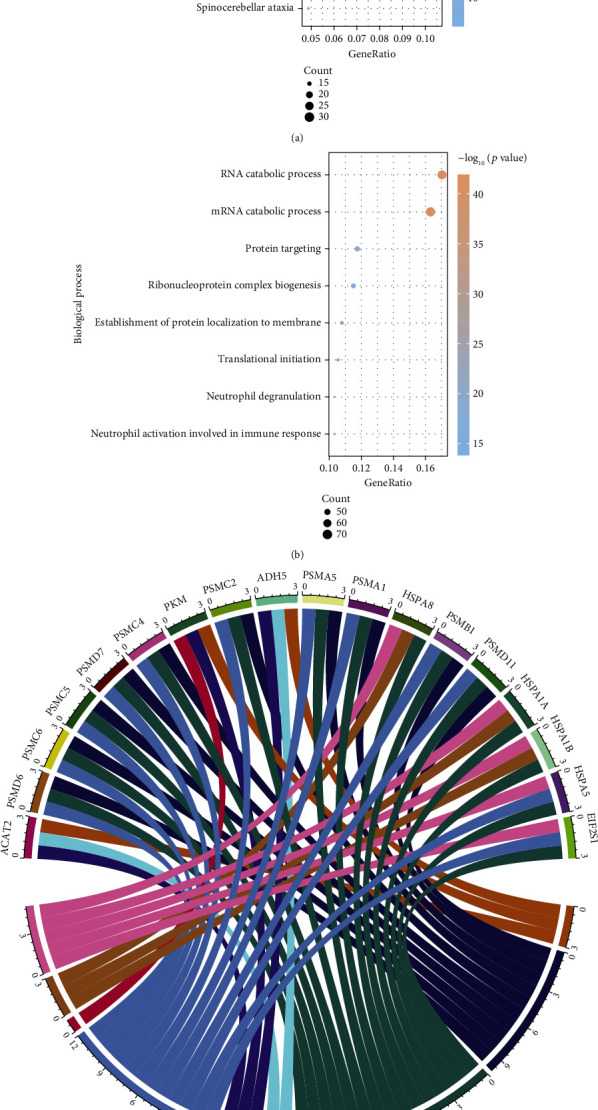
Pathway and biological process annotations enriched with the Tip60-targeted Khib proteome. (a) Representative pathway enriched with the Tip60-targeted Khib proteome. (b) GO chord graph shows that Tip60-targeted substrates are involved in multiple pathways. (c) Representative biological process annotations enriched with the Tip60-targeted Khib proteome.

To explore the possible cellular pathways and biological functions of Tip60-targeted Khib substrates, we employed Kyoto Encyclopedia of Genes and Genomes (KEGG) and GO databases for enrichment analysis. Our results showed that a total of 30 Tip60-targeted Khib proteins were highly correlated with ribosomal pathways (adjusted *p* = 4.76*E* − 12), while 16 and 22 Tip60-targeted Khib proteins were deeply involved in the biosynthesis of amino acids (adjusted *p* = 2.98*E* − 7) and carbon metabolism pathways (adjusted *p* = 7.10*E* − 9), respectively (Figure 3(a)). Interestingly, some Tip60-targeted Khib proteins are involved in multiple pathways. For example, proteasome subunit alpha type-1 (PSMA1), 26S proteasome regulatory subunit 7 (PSMC2), 26S proteasome non-ATPase regulatory subunit 7 (PSMD7), 26S proteasome regulatory subunit 8 (PSMC5), and the other six proteasome complex members are involved in three pathways, including proteasome (adjusted *p* = 1.53*E* − 05), Parkinson disease (adjusted *p* = 1.16*E* − 03), Prion disease (adjusted *p* = 3.74*E* − 04), and amyotrophic lateral sclerosis (adjusted *p* = 6.80*E* − 04) (Figure 3(b)).

In addition, protein function annotation revealed that the Tip60-targeted Khib proteins were significantly enriched in the processes related to nucleic acid metabolism and translation, such as RNA catabolic process (adjusted *p* = 4.54*E* − 39), mRNA catabolic process (adjusted *p* = 4.54*E* − 39), and translational initiation (adjusted *p* = 5.08*E* − 29) (Figure 3(c)), suggesting that Tip60 may influence the biosynthesis process of certain proteins by mediating Khib. Additionally, cotranslational protein targeting to membrane (adjusted *p* = 1.17*E* − 25) and SRP-dependent cotranslational protein targeting to membrane (adjusted *p* = 5.04*E* − 25) were enriched, implying the potential role of Tip60-mediated Khib in the cotranslational translocation pathway.

### 3.4. The Differences in Khib Substrates Regulated by p300 and Tip60

A previous study identified p300 as a Khib “writer” that can regulate glycolysis through the Khib pathway in response to nutritional cues [10]. Considering the Khib-transferase activity of Tip60, does Tip60-mediated Khib have unique roles? To this end, we next sought to compare the Khib substrates regulated by p300 and Tip60 and investigate the differences in related pathways and biological processes.

**Figure 4:**
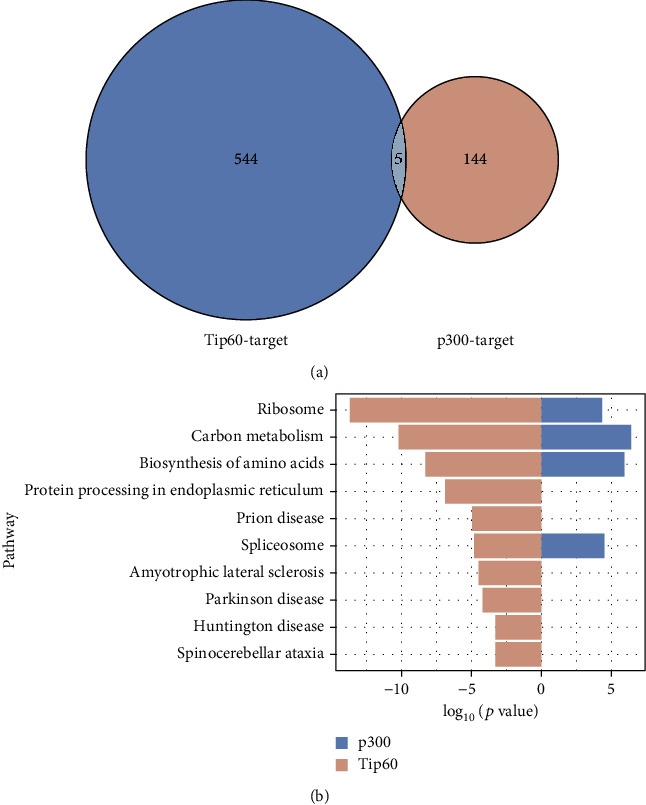
Tip60 and P300 regulate different Khib substrates. (a) Overlap between Tip60-targeted Khib and p300-targeted Khib. (b) Bar graphs show representative pathways enriched with p300- and Tip60- targeted Khib proteomes.

Strikingly, only 5 out of the 549 Khib sites regulated by Tip60 coincided with the 149 Khib sites regulated by p300 (Figure 4(a)). Although the Khib levels in different cell lines may be different, the lower coincidence between Tip60- and p300-targeted Khib suggests that Tip60 and p300 have diverse substrate selectivity to Khib and may differentially regulate downstream biological processes through the Khib pathway. In support of this notion, although both Tip60- and p300-targeted Khib substrates are involved in several pathways such as ribosome (adjusted *p* = 4.76*E* − 12), biosynthesis of amino acids (adjusted *p* = 2.98*E* − 07), and carbon metabolism (adjusted *p* = 7.10*E* − 09), Tip60-mediated Khib substrates are uniquely enriched in protein processing in endoplasmic reticulum (adjusted *p* = 6.10*E* − 06), Prion disease (adjusted *p* = 3.74*E* − 04), Parkinson disease (adjusted *p* = 1.16*E* − 03), and amyotrophic lateral sclerosis (adjusted *p* = 6.80*E* − 04) (Figure 4(b)).

### 3.5. Interaction Network of Tip60-Targeted Khib Substrates

**Figure 5:**
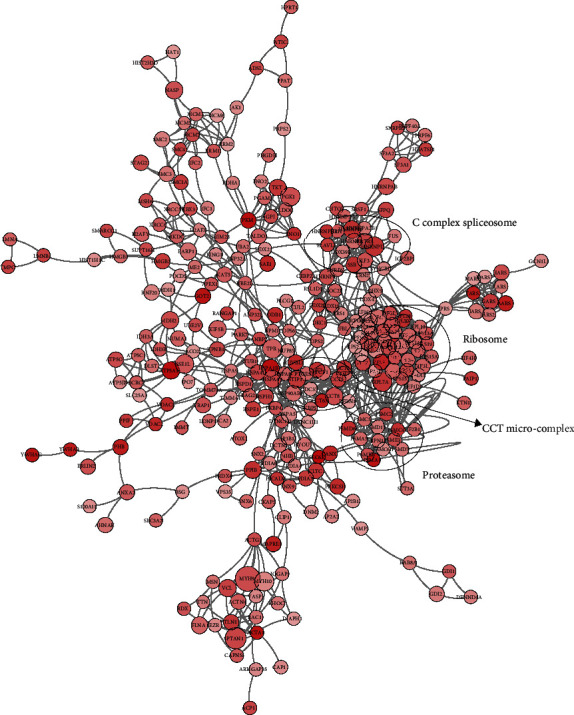
Interaction network of Tip60-targeted Khib substrates based on the STRING database (v11). The network is visualized in Cytoscape (v.3.2.1). A larger protein node represents more Khib sites. Darker color represents higher scores.

Numerous proteins act by binding to partners and the deficiency of particular protein-protein interactions (PPIs) results in a variety of diseases [17–19]. PTMs can manage PPIs due to the ability to recruit binding proteins. Therefore, we next visualized the interaction network of Tip60-targeted Khib proteins based on the STRING database [15]. The network indicated that Tip60-targeted Khib proteins are highly connected. Some protein nodes have plural Tip60-targeted Khib sites and represent the subcenters of the protein interaction network mediated by Tip60 (Figure 5). The undulation of these Khib sites mediated by Tip60 may result in great changes in the PPI network and dysfunction of the complex.

Our data reveal several protein complexes that are significantly regulated by Tip60, including Nop56p-associated pre-rRNA complex (adjusted *p* = 1.85*E* − 42), ribosome (adjusted *p* = 1.95*E* − 35), CCT microcomplex (adjusted *p* = 6.71*E* − 13), and C complex spliceosome (adjusted *p* = 8.15*E* − 10). The Nop56p-associated pre-rRNA complex affects 60S subunit biogenesis in the early-to-mid stages, and the C complex spliceosome plays a vital role in eliminating noncoding introns from nascent pre-mRNAs [20, 21]. Khib on most components of these complexes was only detected in Tip60 OE cells. In addition, the CCT microcomplex is highly involved in protein folding, assembly, and transportation [22, 23], and Khib on all the components of this complex could only be detected in Tip60 OE cells. These results imply the potential roles of Tip60 in protein folding, assembly, and transport processes by regulating Khib.

### 3.6. Possible Impact of Tip60 OE on Khib Substrate Functions

To explore the possible impact of Tip60 OE on the function of Khib substrates, we annotated the Tip60-targeted Khib site based on the UniProt database (http://www.uniprot.org). Interestingly, we found that 5 Khib sites are key positions for binding to substrates or cofactors (Table 1). For example, pyruvate kinase (PKM), the rate-limiting enzyme in the glycolysis pathway, catalyzes the transfer of the phosphoryl group from phosphoenolpyruvate (PEP) to ADP and generates ATP [24, 25]. We identified the Tip60-targeted Khib sites at PKM K207 and PGK1 K220, which are the key residues for ATP binding. Hydroxymethylglutaryl-CoA synthase (HMGCS1) catalyzes the condensation of acetyl-CoA and acetoacetyl-CoA to formulate HMG-CoA, and K46 of HMGCS1 is a key residue for coenzyme A binding [26, 27]. Khib on these residues will most likely interrupt substrate/cofactor binding and introduce harmful effects on protein functions.

In addition, some Tip60-targeted Khib sites are located at positions that are important for PPIs. For example, heat shock protein HSP 90-*β* (HSP90AB1) is involved in many important biological processes and contributes to the structural maintenance, ripeness, and proper regulation of particular target proteins [28, 29]. K69 of HSP90AB1 is the key site for interacting with TP53, BIRC2, and NR3C1. Tip60-mediated Khib at this position may affect the interaction of HSP90AB1 with its binding partners.

Moreover, several Tip60-targeted Khib were identified on cancer biomarkers, such as plectin (PLEC, related to ovarian cancer), alcohol dehydrogenase class-3 (ADH5, related to breast cancer), and PARK7 (related to lung cancer), which therefore links Tip60 and the Khib pathway to cancer.

## 4. Discussion

Tip60 and p300 have been identified as Khib “writers,” and p300-catalyzed Khib has been revealed to regulate cellular glucose metabolism [9, 10]. However, Tip60-targeted Khib substrates have not yet been fully studied, hindering our understanding of Khib functions mediated by Tip60. In this study, a quantitative proteomics study was performed and represents the first comprehensive analysis of Khib substrates in response to Tip60 OE. A total of 3502 Khib sites from 1050 proteins were determined in human cells, of which 536 Khib sites from 406 proteins were only detected in Tip60 OE cells, and the abundance of 13 sites in 13 proteins increased more than 2-fold in response to Tip60 OE. These proteins were designated as potential Tip60 substrates. Furthermore, the Tip60-targeted Khib distributes in diverse subcellular compartments, suggesting that the functions of the Tip60-mediated Khib pathway are likely widespread.

We found that some Tip60-targeted Khib substrates are highly correlated with ribosomal pathways, such as mRNA translation and protein cofolding. In addition, Khib sites in all the components of the CCT microcomplex, a complex that is highly involved in protein folding, assembly, and transportation, could only be detected in Tip60 OE cells. This is consistent with the roles of Tip60 in regulating transcription and implies other cellular processes related to Khib.

Emerging evidence has demonstrated that Tip60 and p300 play different roles in diverse cellular processes by differentially regulating PTMs [30]. We also revealed that Tip60- and p300-targeted Khib sites are quite different. Given that the Khib substrates regulated by Tip60 and p300 are associated with different biological functions and enriched in different pathways, this study provides new insights into the different regulatory mechanisms by which Tip60 and p300 exert their functions.

As a widely known acetyltransferase, Tip60 regulates other acylations as well. For example, in a recent study, Tip60 accurately controlled spindle positioning during mitosis by mediating crotonylation at Lys66 on EB1 [31]. Interestingly, the Lys66 site can also be acetylated by Tip60 in vitro, which suggests that Tip60 regulates its target substrates and corresponding biological processes in a complex way.

Moreover, 23 and 25 Tip60-targeted Khib proteins are involved in the Parkinson's disease and Prion disease pathways, respectively, and 43 Tip60-targeted Khib proteins are connected with cancer genes or cancer biomarkers, which therefore link the Tip60-targeted Khib pathway to diseases.

## Figures and Tables

**Table 1:** Tip60-targeted Khib sites on key residues that participate in cofactor/substrate binding, protein interactions, and cancer biomarkers.

Gene name	Site	Function
PKM	207	ATP-binding site
PGK1	220	ATP-binding site
FDPS	123	Isopentenyl diphosphate binding site
HMGCS1	46	Coenzyme A binding site
LRPPRC	1357	RNA-binding site
PLEC	3505	Ovarian cancer biomarker
ADH5	107	Breast cancer biomarker
PARK7	148	Lung cancer biomarker
HSP90AB1	69	Interaction with TP53, BIRC2, NR3C1
LMNA	78	Interaction with MLIP; Spitzoid tumor gene

## Data Availability

The mass spectrometry proteomics data have been deposited to the ProteomeXchange Consortium via the PRIDE partner repository with the dataset identifier PXD029297.

## Supplementary Materials

Supplementary Materials Tables S1 and S2 are the complete lists of identified Khib and Kac sites in WT and Tip60 OE cells. Supplementary Materials Figure S1 is the representative MS2 spectra of the Tip60-targeted Khib peptides. Table S1: complete list of identified Khib sites in WT and Tip60 OE cells. Table S2: complete list of identified Kac sites in WT and Tip60 OE cells. Figure S1: the representative MS2 spectra of the Tip60-targeted Khib peptides, including K147hib of EF1G, K115hib of KPYM, K416hib of SEC63, and K624hib of HS90B.
