# Supplementary material for: Quantitative Proteomics Reveals the Role of Lysine 2-Hydroxyisobutyrylation Pathway Mediated by Tip60
Source: Oxid Med Cell Longev. 2022 Feb 8;2022:4571319. doi: 10.1155/2022/4571319 (PMC8847014; doi:10.1155/2022/4571319)
Supplement: Supplementary Materials — Tables S1 and S2 are the complete lists of identified Khib and Kac sites in WT and Tip60 OE cells. Supplementary Materials Figure S1 is the representative MS2 spectra of the Tip60-targeted Khib peptides. Table S1: complete list of identified Khib sites in WT and Tip60 OE cells. Table S2: complete list of identified Kac sites in WT and Tip60 OE cells. Figure S1: the representative MS2 spectra of the Tip60-targeted Khib peptides, including K147hib of EF1G, K115hib of KPYM, K416hib of SEC63, and K624hib of HS90B. [file 4571319.f1.zip › SI_Table S1.pdf]

Table S1. Complete list of identified Khib sites in WT and Tip60 OE cells.

Column describtion

**Protein:**	Identifiers of proteins this site is associated with
**Gene names:**	NCBI Gene names of leading proteins
**Position:**	For each protein identifier in the 'Proteins' column you find here the psoition of the site in the respective protein sequence
**Protein names:**	NCBI Gene identifiers of leading proteins
**Score:**	Andromeda score of the identified MS/MS spectra
**Hib (K) Probabilities:**	Sequence representation of the peptide including PTM positioning probabilities ([0..1], where 1 is best match) for 'Hib (K)'.
**Ratio WT/OE:**	Abundance ratio of the site, WT/OE

Protein	Gene names	Position	Protein names	Score	Hib (K) Probabilities	Ratio WT/OE
P49588	AARS	762	Alanine--tRNA ligase, cytoplasmic	91.855	IVAVTGAEAQK(1)ALR	0.81
P49588	AARS	74	Alanine--tRNA ligase, cytoplasmic	91.9	AANTQK(1)CIR	0.85
P49588	AARS	834	Alanine--tRNA ligase, cytoplasmic	73.248	ASK(1)ADVQK	0.91
P49588	AARS	451	Alanine--tRNA ligase, cytoplasmic	119.27	LAQLK(1)SQGK	0.94
P49588	AARS	396	Alanine--tRNA ligase, cytoplasmic	88.37	K(1)IQSLGDSK	0.96
P49588	AARS	934	Alanine--tRNA ligase, cytoplasmic	70.942	GGGK(1)DVSAQATGK	0.99
P49588	AARS	677	Alanine--tRNA ligase, cytoplasmic	40.844	AVYTQDCPLAAAK(1)AIQGLR	1.04
P49588	AARS	338	Alanine--tRNA ligase, cytoplasmic	106.29	YAHEK(1)LNASR	1.17
P49588	AARS	625	Alanine--tRNA ligase, cytoplasmic	156.24	SVLGEADQK(1)GSLVAPDR	Tip60 OE only
P49588	AARS	782	Alanine--tRNA ligase, cytoplasmic	127.02	VK(1)AQTAPNK	Tip60 OE only
Q9NRN7	AASDHPPT	45	L-aminoadipate-semialdehyde dehydrogenase-ph	138.54	SIQPEEK(1)ER	0.98
Q9NRN7	AASDHPPT	180	L-aminoadipate-semialdehyde dehydrogenase-ph	78.934	NWALK(1)ESFIK	1.57
Q9NRN7	AASDHPPT	155	L-aminoadipate-semialdehyde dehydrogenase-ph	93.178	FTNK(1)EWETIR	Tip60 OE only
Q9NRN7	AASDHPPT	58	L-aminoadipate-semialdehyde dehydrogenase-ph	57.177	DAK(1)AAMAGR	unquantifiable
P33527	ABCC1	952	Multidrug resistance-associated protein 1	47.261	LMEADK(1)AQTGQVK	Tip60 OE only
P28288	ABCD3	61	ATP-binding cassette sub-family D member 3	64.841	AVVDK(1)VFFSR	Tip60 OE only
P61221	ABCE1	343	ATP-binding cassette sub-family E member 1	90.108	VAETANEEEVK(1)K	0.91
Q8NE71	ABCF1	430	ATP-binding cassette sub-family F member 1	127.76	ATGAAAAEAK(1)AR	1
Q8NE71	ABCF1	543	ATP-binding cassette sub-family F member 1	71.359	MYQQK(1)QK	1.02
Q9UG63	ABCF2	66	ATP-binding cassette sub-family F member 2	65.246	ELEDFEMK(1)K	unquantifiable
P42765	ACAA2	181	3-ketoacyl-CoA thiolase, mitochondrial	83	EECDK(1)YALQSQQR	1.31
Q709F0	ACAD11	423	Acyl-CoA dehydrogenase family member 11	54.157	PLVIDK(1)LK	Tip60 OE only

Q9H845	ACAD9	456	Acyl-CoA dehydrogenase family member 9, mitoc	102.77	IHELK(1)QAK	1
P11310	ACADM	301	Medium-chain specific acyl-CoA dehydrogenase,	84.658	ALDEATK(1)YALER	Tip60 OE only
P24752	ACAT1	190	Acetyl-CoA acetyltransferase, mitochondrial	53.342	DGLTDVYNK(1)IHMGSCAENT	0.85
P24752	ACAT1	257	Acetyl-CoA acetyltransferase, mitochondrial	59.205	EDEEYK(1)R	1.12
Q9BWD1	ACAT2	180	Acetyl-CoA acetyltransferase, cytosolic	81.625	EDQDK(1)VAVLSQNR	0.75
Q9BWD1	ACAT2	217	Acetyl-CoA acetyltransferase, cytosolic	82.831	GLIEVK(1)TDEFPR	Tip60 OE only
P53396	ACLY	166	ATP-citrate synthase	54.205	LNPEDIK(1)K	0.81
P53396	ACLY	780	ATP-citrate synthase	41.704	NQALK(1)EAGVFVPR	0.86
P53396	ACLY	272	ATP-citrate synthase	114.6	LTLLNPK(1)GR	0.89
P53396	ACLY	978	ATP-citrate synthase	144.09	VK(1)SINNPDMR	0.91
P53396	ACLY	265	ATP-citrate synthase	108.31	SGASLK(1)LTLLNPK	0.94
P53396	ACLY	4	ATP-citrate synthase	100.79	SAK(1)AISEQTGK	0.96
P53396	ACLY	97	ATP-citrate synthase	113.77	LGQEATVGK(1)ATGFLK	1.01
P53396	ACLY	944	ATP-citrate synthase	53.569	FGGALDAAAK(1)MFSK	1.08
P53396	ACLY	230	ATP-citrate synthase	103.26	VDATADYICK(1)VK	1.14
Q99798	ACO2	309	Aconitate hydratase, mitochondrial	44.692	YLSK(1)TGR	0.5
Q99798	ACO2	520	Aconitate hydratase, mitochondrial	57.175	FNPETDYLTGTDGK(1)K	0.75
Q9Y305	ACOT9	103	Acyl-coenzyme A thioesterase 9, mitochondrial	116.3	EK(1)YLTVQNTVR	1.14
P24666	ACP1	113	Low molecular weight phosphotyrosine protein	101.93	AK(1)IELLGSYDPQK	Tip60 OE only
O95573	ACSL3	220	Long-chain-fatty-acid--CoA ligase 3	71.296	ELLQTK(1)LK	Tip60 OE only
P68133	ACTA1;ACTC1	86	Actin, alpha skeletal muscle;Actin, alpha cardiac	176.48	YPIEHGIITNWDDMEK(1)IWH	Tip60 OE only
P63261	ACTG1;ACTB	113	Actin, cytoplasmic 2;Actin, cytoplasmic 2, N-terr	192.54	VAPEEHPVLLTEAPLNPK(1)AN	0.96
P63261	ACTG1;ACTB	291	Actin, cytoplasmic 2;Actin, cytoplasmic 2, N-terr	227.4	K(1)DLYANTVLSGGTTMYPGI	1
P63261	ACTG1;ACTB;ACTA1;	50	Actin, cytoplasmic 2;Actin, cytoplasmic 2, N-terr	268.2	HQGVMVGMGQK(1)DSYVGD	0.98
P63261	ACTG1;ACTB;ACTA1;	315	Actin, cytoplasmic 2;Actin, cytoplasmic 2, N-terr	118.87	MQK(1)EITALAPSTMK	0.98
P63261	ACTG1;ACTB;ACTA1;	61	Actin, cytoplasmic 2;Actin, cytoplasmic 2, N-terr	179.1	DSYVGDEAQSK(1)R	1.02
P63261	ACTG1;ACTB;ACTA1;	326	Actin, cytoplasmic 2;Actin, cytoplasmic 2, N-terr	138.22	EITALAPSTMK(1)IK	1.04
P63261	ACTG1;ACTB;ACTBL2	328	Actin, cytoplasmic 2;Actin, cytoplasmic 2, N-terr	101.72	IK(1)IIAPPER	1.03
P63261	ACTG1;ACTB;POTEE;F	215	Actin, cytoplasmic 2;Actin, cytoplasmic 2, N-terr	235.76	EK(1)LCYVALDFEQEMATAASS	0.92
P63261	ACTG1;ACTB;POTEE;F	213	Actin, cytoplasmic 2;Actin, cytoplasmic 2, N-terr	95.608	EIVRDIK(1)EK	Tip60 OE only
P12814	ACTN1	312	Alpha-actinin-1	95.767	VPENTMHAMQQK(1)LEDFR	1.14
O43707	ACTN4	760	Alpha-actinin-4	134.13	DAK(1)GISQEQMQEFR	0.8
O43707	ACTN4	632	Alpha-actinin-4	107.66	VQQLVPK(1)R	1.06
O43707	ACTN4	437	Alpha-actinin-4	101.33	EAMLK(1)HR	1.09

O43707	ACTN4	859	Alpha-actinin-4	116.01	VLAGDK(1)NFITAEELR	1.1
O43707	ACTN4	518	Alpha-actinin-4	66.073	EALEK(1)TEK	1.11
O43707	ACTN4	331	Alpha-actinin-4	196.76	TIQEMQQK(1)LEDFR	1.11
O43707	ACTN4	214	Alpha-actinin-4	54.524	HRPELIEYDK(1)LR	1.27
O43707	ACTN4	323	Alpha-actinin-4	126.03	VPQK(1)TIQEMQQK	1.32
O43707	ACTN4	432	Alpha-actinin-4	85.522	ASIHEAWTDGK(1)EAMLK	Tip60 OE only
O43707	ACTN4	346	Alpha-actinin-4	60.434	VHK(0.002)PPK(0.998)VQEK	Tip60 OE only
O43707	ACTN4;ACTN1;ACTN2	455	Alpha-actinin-4;Alpha-actinin-1;Alpha-actinin-2	85.619	K(1)HEAFESDLAAHQDR	0.96
O43707	ACTN4;ACTN3;ACTN1	122	Alpha-actinin-4;Alpha-actinin-3;Alpha-actinin-1	84.213	ALDFIASK(1)GVK	0.79
O43707	ACTN4;ACTN3;ACTN1	50	Alpha-actinin-4;Alpha-actinin-3;Alpha-actinin-1;	107.4	DLLLDPAWEK(1)QQR	0.98
P61160	ACTR2	253	Actin-related protein 2	70.69	IIK(1)VGGER	0.85
P61160	ACTR2	219	Actin-related protein 2	177.1	EK(1)LCYVGYNIEQEQK	0.91
P61160	ACTR2	322	Actin-related protein 2	87.476	ELK(1)QLYLER	0.93
P61158	ACTR3	42	Actin-related protein 3	53.751	ESAK(1)VGDQAQR	1.05
P35611	ADD1	295	Alpha-adducin	42.914	SK(1)VLILR	unquantifiable
P11766	ADH5	107	Alcohol dehydrogenase class-3	85.377	FCLNPK(1)TNLCQK	Tip60 OE only
P11766	ADH5	338	Alcohol dehydrogenase class-3	68.224	LVSEYMSK(1)K	Tip60 OE only
P30566	ADSL	229	Adenylosuccinate lyase	97.798	MVTEK(1)AGFK	Tip60 OE only
Q9Y4W6	AFG3L2	719	AFG3-like protein 2	92.439	TVALLTEK(1)K	unquantifiable
P23526	AHCY	408	Adenosylhomocysteinase	87.754	LTK(1)LTEK	0.86
P23526	AHCY	43	Adenosylhomocysteinase	54.157	YSASK(1)PLK	0.91
P23526	AHCY	405	Adenosylhomocysteinase	58.815	LNVK(1)LTK	0.92
P23526	AHCY	186	Adenosylhomocysteinase	199.7	VPAINVNDSVTK(1)SK	0.96
P23526	AHCY	226	Adenosylhomocysteinase	267.75	VAVVAGYGDVGK(1)GCAQALI	1.03
P23526	AHCY	188	Adenosylhomocysteinase	193.41	SK(1)FDNLYGCR	1.04
P23526	AHCY	20	Adenosylhomocysteinase	107.85	K(1)ALDIAENEMPGLMR	1.14
O43865	AHCYL1	359	Putative adenosylhomocysteinase 2	48.157	VVK(1)LNEVIR	unquantifiable
Q09666	AHNAK	775	Neuroblast differentiation-associated protein AH	152.34	AEGPEVDVNLPK(1)ADVDISG	0.76
Q09666	AHNAK	1408	Neuroblast differentiation-associated protein AH	68.355	LPK(1)ADVDVSGPK	0.94
Q09666	AHNAK	1280	Neuroblast differentiation-associated protein AH	72.652	LPK(1)ADIDVSGPK	0.99
Q09666	AHNAK	805	Neuroblast differentiation-associated protein AH	54.056	LKGPK(1)FK	1.05
Q09666	AHNAK	884	Neuroblast differentiation-associated protein AH	83.998	MPK(1)FSMPGFK	1.1
Q09666	AHNAK	807	Neuroblast differentiation-associated protein AH	48.036	FK(1)MPEMNIK	1.26
Q09666	AHNAK	2920	Neuroblast differentiation-associated protein AH	52.522	AEGPEVDVNLPK(1)ADVDVSG	Tip60 OE only

Q09666	AHNAK	1305	Neuroblast differentiation-associated protein AH	67.997	VDVEVPDVSLEGPEGK(1)LK	Tip60 OE only
Q12904	AIMP1	54	Aminoacyl tRNA synthase complex-interacting m	42.229	VENAK(1)LK	1.29
Q12904	AIMP1	35	Aminoacyl tRNA synthase complex-interacting m	61.65	EK(1)AILQATLR	1.33
Q13155	AIMP2	70	Aminoacyl tRNA synthase complex-interacting m	88.441	LYELK(1)AAVDGLSK	0.82
P00568	AK1	27	Adenylate kinase isoenzyme 1	60.436	GTQCEK(1)IVQK	0.91
P00568	AK1	83	Adenylate kinase isoenzyme 1	51.927	DAMVAK(1)VNTSK	Tip60 OE only
P54819	AK2	65	Adenylate kinase 2, mitochondrial;Adenylate kin	66.692	LK(1)ATMDAGK	1.02
Q99996	AKAP9	1515	A-kinase anchor protein 9	43.808	EEFK(1)PLSK	unquantifiable
P15121	AKR1B1	263	Aldose reductase	83	NLVVIPK(1)SVTPER	unquantifiable
P54886	ALDH18A1	409	Delta-1-pyrroline-5-carboxylate synthase;Glutam	58.433	K(1)DLEEAEGR	0.71
P54886	ALDH18A1	311	Delta-1-pyrroline-5-carboxylate synthase;Glutam	50.292	VGMGGMEAK(1)VK	0.8
P54886	ALDH18A1	643	Delta-1-pyrroline-5-carboxylate synthase;Glutam	113.71	VEQVK(1)IHAGPK	0.9
P54886	ALDH18A1	550	Delta-1-pyrroline-5-carboxylate synthase;Glutam	68.893	LDK(1)MIDLIIPR	Tip60 OE only
P30837	ALDH1B1	280	Aldehyde dehydrogenase X, mitochondrial	77.674	AAGDSNLK(1)R	Tip60 OE only
P05091	ALDH2	280	Aldehyde dehydrogenase, mitochondrial	50.284	VIQVAAGSSNLK(1)R	2.32
P51649	ALDH5A1	411	Succinate-semialdehyde dehydrogenase, mitocho	69.825	GATVVTGGK(1)R	unquantifiable
P49189	ALDH9A1	68	4-trimethylaminobutyraldehyde dehydrogenase	120.12	IWSQK(1)SGMER	1.04
P49189	ALDH9A1	344	4-trimethylaminobutyraldehyde dehydrogenase	42.109	VLGFVK(1)VAK	1.06
P04075	ALDOA	312	Fructose-bisphosphate aldolase A	157.24	ALQASALK(1)AWGGK	0.84
P04075	ALDOA	28	Fructose-bisphosphate aldolase A	126.48	IVAPGK(1)GILAADESTGSIAK	0.85
P04075	ALDOA	322	Fructose-bisphosphate aldolase A	83.617	ENLK(1)AAQEEYVK	0.89
P04075	ALDOA	330	Fructose-bisphosphate aldolase A	194.27	AAQEEYVK(1)R	0.93
P04075	ALDOA	13	Fructose-bisphosphate aldolase A	64.73	PYQYPALTPEQK(1)K	0.99
P04075	ALDOA	14	Fructose-bisphosphate aldolase A	76.943	K(1)ELSDIAHR	1
P04075	ALDOA	42	Fructose-bisphosphate aldolase A	172.8	GILAADESTGSIAK(1)R	1.02
P04075	ALDOA;ALDOC	140	Fructose-bisphosphate aldolase A;Fructose-bisph	60.91	K(1)DGADFAK	1.12
P04075	ALDOA;ALDOC	147	Fructose-bisphosphate aldolase A;Fructose-bisph	109.01	DGADFAK(1)WR	1.22
P09972	ALDOC	111	Fructose-bisphosphate aldolase C	82.877	VDK(1)GVVPLAGTDGETTTQG	Tip60 OE only
Q86V81	ALYREF	134	THO complex subunit 4	44.203	K(1)AAVHYDR	unquantifiable
P39687	ANP32A	86	Acidic leucine-rich nuclear phosphoprotein 32 fa	60.472	VSGGLEVLAEK(1)CPNLTHLNL	Tip60 OE only
P39687	ANP32A	68	Acidic leucine-rich nuclear phosphoprotein 32 fa	85.064	K(1)LELSDNR	unquantifiable
Q92688	ANP32B	99	Acidic leucine-rich nuclear phosphoprotein 32 fa	52.372	LPNLTHLNLSGNK(1)LK	0.95
Q92688	ANP32B	28	Acidic leucine-rich nuclear phosphoprotein 32 fa	82.417	ELVLDNCK(1)SNDGK	0.98
Q9BTT0	ANP32E	65	Acidic leucine-rich nuclear phosphoprotein 32 fa	94.262	LPSLNK(1)LR	Tip60 OE only

P04083	ANXA1	245	Annexin A1	87.696	YSK(1)HDMNK	0.7
P04083	ANXA1	281	Annexin A1	75.479	PAFFAEK(1)LHQAMK	0.73
P50995	ANXA11	248	Annexin A11	57.59	TAYGK(1)DLIK	0.86
P50995	ANXA11	255	Annexin A11	118.4	DLK(1)SELSGNFEK	0.91
P07355	ANXA2;ANXA2P2	148	Annexin A2;Putative annexin A2-like protein	69.383	VYK(1)EMYK	0.84
P07355	ANXA2;ANXA2P2	157	Annexin A2;Putative annexin A2-like protein	194.7	TDLEK(1)DIISDTSGDFR	0.88
P07355	ANXA2;ANXA2P2	47	Annexin A2;Putative annexin A2-like protein	126.19	DALNIETAIK(1)TK	0.9
P07355	ANXA2;ANXA2P2	324	Annexin A2;Putative annexin A2-like protein	160.15	SLYYYIQQDTK(1)GDYQK	0.91
P07355	ANXA2;ANXA2P2	115	Annexin A2;Putative annexin A2-like protein	180.09	TPAQYDASELK(1)ASMK	0.94
P07355	ANXA2;ANXA2P2	152	Annexin A2;Putative annexin A2-like protein	83.005	EMYK(1)TDLEK	0.96
P07355	ANXA2;ANXA2P2	204	Annexin A2;Putative annexin A2-like protein	114.78	DLYDAGVK(1)R	0.97
P07355	ANXA2;ANXA2P2	169	Annexin A2;Putative annexin A2-like protein	85.265	K(1)LMVALAK	0.98
P07355	ANXA2;ANXA2P2	233	Annexin A2;Putative annexin A2-like protein	65.842	YK(1)SYSPYDMLESIR	0.99
P07355	ANXA2;ANXA2P2	176	Annexin A2;Putative annexin A2-like protein	91.041	LMVALAK(1)GR	1.08
P07355	ANXA2;ANXA2P2	227	Annexin A2;Putative annexin A2-like protein	84.479	SVPHLQK(1)VFDR	Tip60 OE only
P07355	ANXA2;ANXA2P2	302	Annexin A2;Putative annexin A2-like protein	84.568	SEVDMLK(1)IR	Tip60 OE only
P09525	ANXA4	246	Annexin A4	63.216	NK(1)SAYFAEK	unquantifiable
P08758	ANXA5	76	Annexin A5	66.692	SELTGK(1)FEK	1.02
P08758	ANXA5	97	Annexin A5	68.44	LYDAYELK(1)HALK	1.12
P08133	ANXA6	81	Annexin A6	117.89	YELTGK(1)FER	1.03
P20073	ANXA7	228	Annexin A7	51.059	AAFK(1)TSYGK	unquantifiable
O94973	AP2A2;AP2A1	6	AP-2 complex subunit alpha-2;AP-2 complex sub	55.549	PAVSK(1)GDGMR	Tip60 OE only
P63010	AP2B1;AP1B1	335	AP-2 complex subunit beta;AP-1 complex subuni	48.284	YNDPIYVK(1)LEK	Tip60 OE only
O00203	AP3B1	90	AP-3 complex subunit beta-1	64.654	NVASK(1)NIEIK	Tip60 OE only
P27695	APEX1	276	DNA-(apurinic or apyrimidinic site) lyase;DNA-(ap	89.427	SK(1)NVGWR	Tip60 OE only
Q9BZZ5	API5	369	Apoptosis inhibitor 5	63.301	LNAEK(1)LK	0.73
Q9BZZ5	API5	409	Apoptosis inhibitor 5	99.919	TEENK(1)IK	0.9
Q9BZZ5	API5	153	Apoptosis inhibitor 5	69.198	AIK(1)FLSTK	0.9
Q9BZZ5	API5	202	Apoptosis inhibitor 5	158.31	ILSGLK(1)SLQTVSGR	0.91
Q9BZZ5	API5	411	Apoptosis inhibitor 5	50.855	IK(1)VVALK	0.92
Q9BZZ5	API5	398	Apoptosis inhibitor 5	115.78	LALQGK(1)TGEALK	0.98
Q9BZZ5	API5	158	Apoptosis inhibitor 5	64.803	FLSTK(1)LK	1.03
Q9BZZ5	API5	160	Apoptosis inhibitor 5	74.533	LK(1)TLPDEVLTK	Tip60 OE only
Q9BZZ5	API5	404	Apoptosis inhibitor 5	74.162	TGEALK(1)TEENK	Tip60 OE only

P07741	APRT	114	Adenine phosphoribosyltransferase	149.23	AELEIQK(1)DALEPGQR	1.11
P07741	APRT	34	Adenine phosphoribosyltransferase	97.734	DISPVLK(1)DPASFR	1.32
P48444	ARCN1	243	Coatomer subunit delta	92.19	LK(1)SEGETIMSSSMGK	0.88
P48444	ARCN1	44	Coatomer subunit delta	112.3	LMNTGK(1)QHTFVETESVR	Tip60 OE only
P62330	ARF6	12	ADP-ribosylation factor 6	83.753	IFGNK(1)EMR	1.21
Q8N6T3	ARFGAP1	114	ADP-ribosylation factor GTPase-activating protei	51.276	DK(1)VVALAEGR	unquantifiable
Q9NRY4	ARHGAP35	124	Rho GTPase-activating protein 35	51.268	AAATK(1)LASAEK	Tip60 OE only
P52565	ARHGDIA	52	Rho GDP-dissociation inhibitor 1	131.41	YK(1)EALLGR	0.95
P52565	ARHGDIA	141	Rho GDP-dissociation inhibitor 1	122.55	IDK(1)TDYMVGSYGPR	1.16
P36405	ARL3	11	ADP-ribosylation factor-like protein 3	122.28	LK(1)SAPDQEVR	0.94
Q15041	ARL6IP1	124	ADP-ribosylation factor-like protein 6-interacting	79.027	AVGWWK(1)R	unquantifiable
Q9NVJ2	ARL8B	141	ADP-ribosylation factor-like protein 8B	98.156	DLPNALDEK(1)QLIEK	Tip60 OE only
O15144	ARPC2	117	Actin-related protein 2/3 complex subunit 2	72.652	DSIVHQAGMLK(1)R	1.09
O15145	ARPC3	56	Actin-related protein 2/3 complex subunit 3	68.893	ANVFFK(1)NYEIK	unquantifiable
O15511	ARPC5	67	Actin-related protein 2/3 complex subunit 5	145.25	NPPINTK(1)SQAVK	1.1
P08243	ASNS	467	Asparagine synthetase [glutamine-hydrolyzing]	117.2	PK(1)EAFSDGITSVK	1.12
Q12797	ASPH	110	Aspartyl/asparaginyl beta-hydroxylase	70.525	VLLGLK(1)ER	unquantifiable
Q8NBU5	ATAD1	60	ATPase family AAA domain-containing protein 1	45.952	LMK(1)QIGVK	0.76
Q8NBU5	ATAD1	112	ATPase family AAA domain-containing protein 1	47.082	DTVILPIK(1)K	1.2
Q9NVI7	ATAD3A	549	ATPase family AAA domain-containing protein 3A	78.692	YVLKPATEGK(1)QR	1.23
P31939	ATIC	254	Bifunctional purine biosynthesis protein PURH;P	75.479	ELK(1)EALGIPAAASFK	0.84
P31939	ATIC	461	Bifunctional purine biosynthesis protein PURH;P	102.61	LAGDK(1)ANYWWLR	Tip60 OE only
O00244	ATOX1	38	Copper transport protein ATOX1	79.713	YDIDLPNK(1)K	Tip60 OE only
P05023	ATP1A1	508	Sodium/potassium-transporting ATPase subunit	78.661	NPNTSEPQHLLVMK(1)GAPER	0.81
P05023	ATP1A1	661	Sodium/potassium-transporting ATPase subunit	111.12	DAK(1)ACVVHGSDLK	1.06
P05023	ATP1A1;ATP1A3;ATP	605	Sodium/potassium-transporting ATPase subunit	71.692	AAVPDAVGK(1)CR	1.03
P05023	ATP1A1;ATP1A3;ATP	698	Sodium/potassium-transporting ATPase subunit	78.908	TSPQQK(1)LIIVEGCQR	0.92
P05023	ATP1A1;ATP1A3;ATP	629	Sodium/potassium-transporting ATPase subunit	109.79	AIAK(1)GVGIISEGNETVEDIAA	0.95
P05023	ATP1A1;ATP1A3;ATP	625	Sodium/potassium-transporting ATPase subunit	69.485	VIMVTGDHPITAK(1)AIAK	0.99
P05023	ATP1A1;ATP1A3;ATP	773	Sodium/potassium-transporting ATPase subunit	94.006	LIFDNLK(1)K	1.07
P54709	ATP1B3	266	Sodium/potassium-transporting ATPase subunit	45.915	SQDDRDK(1)FLGR	0.93
P54709	ATP1B3	259	Sodium/potassium-transporting ATPase subunit	158.05	IDGSANLK(1)SQDDR	Tip60 OE only
P16615	ATP2A2	541	Sarcoplasmic/endoplasmic reticulum calcium AT	131.22	VPMTSGVK(1)QK	1.05
P16615	ATP2A2;ATP2A1	514	Sarcoplasmic/endoplasmic reticulum calcium AT	60.434	MFVK(1)GAPEGVIDR	0.95

P25705	ATP5A1	261	ATP synthase subunit alpha, mitochondrial	111.65	STVAQLVK(1)R	0.82
P25705	ATP5A1	126	ATP synthase subunit alpha, mitochondrial	80.438	LIK(1)EGDIVK	0.89
P25705	ATP5A1	531	ATP synthase subunit alpha, mitochondrial	67.726	ADGK(1)ISEQSDAK	0.92
P25705	ATP5A1	167	ATP synthase subunit alpha, mitochondrial	97.798	GPIGSK(1)TR	0.92
P25705	ATP5A1	503	ATP synthase subunit alpha, mitochondrial	58.815	LEPSK(1)ITK	0.93
P25705	ATP5A1	132	ATP synthase subunit alpha, mitochondrial	74.415	EGDIVK(1)R	1
P25705	ATP5A1	239	ATP synthase subunit alpha, mitochondrial	112.84	FNDGSDEK(1)K	1.01
P25705	ATP5A1	539	ATP synthase subunit alpha, mitochondrial	120.15	ISEQSDAK(1)LK	1.02
P25705	ATP5A1	427	ATP synthase subunit alpha, mitochondrial	78.113	AMK(1)QVAGTMK	1.66
P25705	ATP5A1	230	ATP synthase subunit alpha, mitochondrial	185.29	TSIAIDTIINQK(1)R	Tip60 OE only
P25705	ATP5A1	175	ATP synthase subunit alpha, mitochondrial	58.433	VGLK(1)APGIIPR	Tip60 OE only
P25705	ATP5A1	161	ATP synthase subunit alpha, mitochondrial	116.51	VVDALGNAIDGK(1)GPIGSK	Tip60 OE only
P25705	ATP5A1	194	ATP synthase subunit alpha, mitochondrial	93.958	EPMQTGIK(1)AVDSLVPIGR	unquantifiable
P06576	ATP5B	133	ATP synthase subunit beta, mitochondrial	95.264	VLDSGAPIK(1)IPVGPETLGR	0.76
P06576	ATP5B	522	ATP synthase subunit beta, mitochondrial	83.753	ADK(1)LAEEHSS	0.84
P06576	ATP5B	201	ATP synthase subunit beta, mitochondrial	84.17	GGK(1)IGLFGGAGVGK	0.85
P06576	ATP5B	198	ATP synthase subunit beta, mitochondrial	84.17	VVDLLAPYAK(1)GGK	0.88
P06576	ATP5B	485	ATP synthase subunit beta, mitochondrial	71.501	LVPLK(1)ETIK	0.94
P06576	ATP5B	264	ATP synthase subunit beta, mitochondrial	148.72	DATSK(1)VALVYGQMNEPPG/	1.02
P06576	ATP5B	124	ATP synthase subunit beta, mitochondrial	88.681	GQK(1)VLDSGAPIK	1.07
P06576	ATP5B	426	ATP synthase subunit beta, mitochondrial	122.13	GVQK(1)ILQDYK	1.17
P36542	ATP5C1	49	ATP synthase subunit gamma, mitochondrial	52.786	SMK(1)MVAAAK	0.92
P36542	ATP5C1	55	ATP synthase subunit gamma, mitochondrial	79.597	MVAAAK(1)YAR	1.06
P36542	ATP5C1	90	ATP synthase subunit gamma, mitochondrial	75.294	K(1)HLLIGVSSDR	Tip60 OE only
P30049	ATP5D	136	ATP synthase subunit delta, mitochondrial	187.16	ANLEK(1)AQAELVGTADEATR	0.91
P56381	ATP5E	37	ATP synthase subunit epsilon, mitochondrial	43.512	ANAEK(1)TSGSNVK	unquantifiable
P56381	ATP5E	44	ATP synthase subunit epsilon, mitochondrial	87.476	TSGSNVK(1)IVK	unquantifiable
P24539	ATP5F1	131	ATP synthase F(0) complex subunit B1, mitochon	162.26	LNEQK(1)LAQLEEAK	0.83
P24539	ATP5F1	233	ATP synthase F(0) complex subunit B1, mitochon	48.741	HVVQSISTQQEK(1)ETIAK	1.1
O75947	ATP5H	63	ATP synthase subunit d, mitochondrial	132.5	ANVAK(1)AGLVDDFEK	0.7
O75947	ATP5H	109	ATP synthase subunit d, mitochondrial	118.4	SCAEWVSLSK(1)AR	1.03
O75947	ATP5H	117	ATP synthase subunit d, mitochondrial	104.17	IVEYEK(1)EMEK	1.2
O75947	ATP5H	32	ATP synthase subunit d, mitochondrial	74.46	AIASSLK(1)SWNETLTSR	unquantifiable
P18859	ATP5J	46	ATP synthase-coupling factor 6, mitochondrial	113.25	LFVDK(1)IR	unquantifiable

P56134	ATP5J2	54	ATP synthase subunit f, mitochondrial	55.902	YYNK(1)YINVK	Tip60 OE only
O75964	ATP5L	66	ATP synthase subunit g, mitochondrial	109.84	IVNSAQTGSFK(1)QLTVK	0.97
P48047	ATP5O	73	ATP synthase subunit O, mitochondrial	70.942	EPK(1)VAASVLNPYVK	0.72
P48047	ATP5O	70	ATP synthase subunit O, mitochondrial	76.064	VAQILK(1)EPK	0.88
P48047	ATP5O	51	ATP synthase subunit O, mitochondrial	160.36	YATALYSAASK(1)QNK	0.98
P48047	ATP5O	176	ATP synthase subunit O, mitochondrial	69.864	LEAK(1)TDPSILGGMIVR	1.33
P48047	ATP5O	199	ATP synthase subunit O, mitochondrial	76.827	YVDMSVK(1)TK	Tip60 OE only
P48047	ATP5O	162	ATP synthase subunit O, mitochondrial	96.015	TVLK(1)SFLSQGQVLK	Tip60 OE only
Q5TC12	ATPAF1	83	ATP synthase mitochondrial F1 complex assembl	53.554	DK(1)IQLLR	unquantifiable
O95816	BAG2	200	BAG family molecular chaperone regulator 2	79.693	GAGSK(1)TLQQNAESR	Tip60 OE only
O75531	BANF1	72	Barrier-to-autointegration factor;Barrier-to-auto	91.584	DTCGANAK(1)QSR	1.2
P80723	BASP1	52	Brain acid soluble protein 1	62.891	ESEPQAAAEPAEAK(1)EGK	0.86
P80723	BASP1	18	Brain acid soluble protein 1	50.878	GYNVNDEK(1)AK	1.38
P51572	BCAP31	199	B-cell receptor-associated protein 31	66.595	LKDELASTK(1)QK	0.81
P51572	BCAP31	72	B-cell receptor-associated protein 31	69.423	K(1)YDDVTEK	1
P51572	BCAP31	158	B-cell receptor-associated protein 31	80.165	YMEENDQLK(1)K	1.07
O15382	BCAT2	321	Branched-chain-amino-acid aminotransferase, m	58.815	TITMK(1)QLLR	0.85
Q9NYF8	BCLAF1	580	Bcl-2-associated transcription factor 1	62.924	LLASTLVHSVK(1)K	1.04
Q8TD16	BICD2	359	Protein bicaudal D homolog 2	73.082	EK(1)AGLLATLQDTQK	unquantifiable
O95861	BPNT1	49	3'(2'),5'-bisphosphate nucleotidase 1	76.228	TCATDLQTK(1)ADR	unquantifiable
P35613	BSG	179	Basigin	69.349	GGVVLK(1)EDALPGQK	0.71
P35613	BSG	173	Basigin	46.426	WLK(1)GGVVLK	Tip60 OE only
P20290	BTF3	54	Transcription factor BTF3	131.62	ETIMNQEK(1)LAK	unquantifiable
P20290	BTF3;BTF3L4	57	Transcription factor BTF3;Transcription factor BT	88.942	LAK(1)LQAQVR	0.82
Q13895	BYSL	242	Bystin	99.136	IFASNLK(1)ER	unquantifiable
Q9Y6E2	BZW2	192	Basic leucine zipper and W2 domain-containing p	58.433	LFK(1)AWMAEK	Tip60 OE only
Q9Y224	C14orf166	171	UPF0568 protein C14orf166	176.48	LTQDAVAK(1)ANQTK	0.94
Q9BQ61	C19orf43	128	Uncharacterized protein C19orf43	48.157	LALK(1)TGIVAK	unquantifiable
P00918	CA2	153	Carbonic anhydrase 2	41.876	VGSAK(1)PGLQK	0.73
P00918	CA2	167	Carbonic anhydrase 2	134.59	VVDVLDSIK(1)TK	0.74
P00918	CA2	251	Carbonic anhydrase 2	66.993	PAQPLK(1)NR	1
P00918	CA2	171	Carbonic anhydrase 2	62.466	GK(1)SADFTNFDPR	Tip60 OE only
Q9HB71	CACYBP	35	Calcyclin-binding protein	83.206	SK(1)IETEIK	1.08
Q9HB71	CACYBP	207	Calcyclin-binding protein	101.11	IYEDGDDDMK(1)R	1.21

Q9HB71	CACYBP	146	Calcyclin-binding protein	128.86	VK(1)TDTVLILCR	Tip60 OE only
Q9HB71	CACYBP	85	Calcyclin-binding protein	80.69	ISNYGWDQSDK(1)FVK	Tip60 OE only
Q9HB71	CACYBP	8	Calcyclin-binding protein	80.979	ASEELQK(1)DLEEVK	Tip60 OE only
P27708	CAD	778	CAD protein;Glutamine-dependent carbamoyl-pl	78.934	SFEEAFQK(1)ALR	1.03
P27708	CAD	1953	CAD protein;Glutamine-dependent carbamoyl-pl	76.035	MMVQK(1)ER	1.04
P27708	CAD	1211	CAD protein;Glutamine-dependent carbamoyl-pl	57.836	DDQLK(1)VIECNVR	1.3
P27797	CALR	43	Calreticulin	117.16	HK(1)SDFGK	0.92
P27797	CALR	55	Calreticulin	136.96	FVLSSGK(1)FYGDEEK	0.94
P27797	CALR	153	Calreticulin	92.677	GK(1)NVLINK	0.96
P27797	CALR	48	Calreticulin	89.548	SDFGK(1)FVLSSGK	0.99
P27797	CALR	159	Calreticulin	119.27	NVLINK(1)DIR	0.99
P27797	CALR	143	Calreticulin	119.27	K(1)VHVIFNYK	1.01
P27797	CALR	209	Calreticulin	68.481	IK(1)DPDASKPEDWDER	1.04
P27797	CALR	64	Calreticulin	140.16	DK(1)GLQTSQDAR	1.05
P27797	CALR	151	Calreticulin	101.99	VHVIFNYK(1)GK	1.13
P27797	CALR	62	Calreticulin	103.56	FYGDEEK(1)DK	1.14
O43852	CALU	281	Calumenin	52.79	HLVYESDQNK(1)DGK	0.81
O43852	CALU	165	Calumenin	48.741	MADK(1)DGDLIATK	1
O43852	CALU	70	Calumenin	78.655	TFDQLTPEESK(1)ER	1.15
O43852	CALU	84	Calumenin	75.479	IDGDK(1)DGFVTVDELK	Tip60 OE only
Q86VP6	CAND1	971	Cullin-associated NEDD8-dissociated protein 1	117.25	LK(1)GYLISGSSYAR	1.02
Q86VP6	CAND1	586	Cullin-associated NEDD8-dissociated protein 1	144.77	AADIDQEVK(1)ER	1.03
Q86VP6	CAND1	1158	Cullin-associated NEDD8-dissociated protein 1	41.502	VK(1)ANSVK	1.05
Q86VP6	CAND1	1156	Cullin-associated NEDD8-dissociated protein 1	46.994	ATCTTK(1)VK	1.08
Q86VP6	CAND1;CAND2	20	Cullin-associated NEDD8-dissociated protein 1;C	107.01	MTSSDK(1)DFR	0.98
P27824	CANX	89	Calnexin	68.893	AK(0.986)K(0.014)DDTDDEI/	0.75
P27824	CANX	137	Calnexin	53.756	LNK(1)PFLFDTK	0.86
P27824	CANX	227	Calnexin	122.39	RPDADLK(1)TYFTDK	0.88
P27824	CANX	217	Calnexin	73.887	TGIYEEK(1)HAK	0.93
P27824	CANX	103	Calnexin	104.22	YDGK(1)WEVEEMK	0.94
P27824	CANX	110	Calnexin	101.65	WEVEEMK(1)ESK	0.96
P27824	CANX	118	Calnexin	84.188	LPGDK(1)GLVLMSR	0.96
P27824	CANX	87	Calnexin	89.171	GTLSGWILSK(1)AK	0.98
P27824	CANX	380	Calnexin	98.249	PVIDNPNYK(1)GK	1.08

P27824	CANX	99	Calnexin	123.79	DDTDDEIAK(1)YDGK	1.28
P27824	CANX	90	Calnexin	86.772	K(1)DDTDDEIAK	1.33
P27824	CANX	458	Calnexin	138.25	IVDDWANDGWGLK(1)K	Tip60 OE only
P27824	CANX	113	Calnexin	69.905	ESK(1)LPGDK	Tip60 OE only
Q01518	CAP1	404	Adenylyl cyclase-associated protein 1	66.498	VQVMGK(1)VPTISINK	1
Q01518	CAP1	366	Adenylyl cyclase-associated protein 1	163.77	GK(1)INSITVDNCK	1.12
Q01518	CAP1	81	Adenylyl cyclase-associated protein 1	51.135	HAEMVHTGLK(1)LER	Tip60 OE only
P07384	CAPN1	583	Calpain-1 catalytic subunit	76.465	TK(1)GFSLESCR	1.27
P04632	CAPNS1	179	Calpain small subunit 1	87.298	WQAIYK(1)QFDTDR	Tip60 OE only
Q14444	CAPRIN1	223	Caprin-1	62.558	EK(1)PVCGTTYK	1.01
P52907	CAPZA1	13	F-actin-capping protein subunit alpha-1	100.45	VSDEEK(1)VR	1.05
P52907	CAPZA1	118	F-actin-capping protein subunit alpha-1	73.927	EASDPQPEEADGGLK(1)SWR	1.13
P52907	CAPZA1	97	F-actin-capping protein subunit alpha-1	73.985	ISFK(1)FDHLR	1.18
P52907	CAPZA1;CAPZA2	268	F-actin-capping protein subunit alpha-1;F-actin-c	60.478	TK(1)IDWNK	1.07
P47756	CAPZB	57	F-actin-capping protein subunit beta	106.16	VVGK(1)DYLLCDYNR	1.13
P47756	CAPZB	223	F-actin-capping protein subunit beta	123.92	LVEDMENK(1)IR	1.21
Q9Y2V2	CARHSP1	68	Calcium-regulated heat stable protein 1	108.98	GVCK(1)CFCR	1.13
P16152	CBR1	148	Carbonyl reductase [NADPH] 1	81.972	ALK(1)SCSPELQQK	0.89
P16152	CBR1	157	Carbonyl reductase [NADPH] 1	55.567	SCSPELQQK(1)FR	1.19
P16152	CBR1	180	Carbonyl reductase [NADPH] 1	58.981	FVEDTK(1)K	unquantifiable
P83916	CBX1	35	Chromobox protein homolog 1	58.917	GK(1)VEYLLK	1.4
Q13185	CBX3	143	Chromobox protein homolog 3	97.813	WK(1)DSDEADLVLAK	Tip60 OE only
Q8IX12	CCAR1	492	Cell division cycle and apoptosis regulator protei	47.067	FLVGMK(1)GK	Tip60 OE only
Q8N163	CCAR2	54	Cell cycle and apoptosis regulator protein 2	54.549	HLQGGEK(1)QR	1.01
Q9H6F5	CCDC86	291	Coiled-coil domain-containing protein 86	46.426	HLEEEK(1)ER	Tip60 OE only
P78371	CCT2	154	T-complex protein 1 subunit beta	171.29	EALLSSAVDHGSDEVK(1)FR	0.74
P78371	CCT2	248	T-complex protein 1 subunit beta	129.89	ILIANTGMDTDK(1)IK	0.92
P78371	CCT2	272	T-complex protein 1 subunit beta	157.97	VAEIEHAEK(1)EK	0.94
P78371	CCT2	203	T-complex protein 1 subunit beta	83.652	GSGNLEAIHIIK(1)K	0.95
P78371	CCT2	191	T-complex protein 1 subunit beta	94.538	LK(1)GSGNLEAIHIIK	0.95
P78371	CCT2	431	T-complex protein 1 subunit beta	98.902	TPGK(1)EAVAMESYAK	0.98
P78371	CCT2	250	T-complex protein 1 subunit beta	97.473	IK(1)IFGSR	1.03
P78371	CCT2	522	T-complex protein 1 subunit beta	87.308	VDNIIK(1)AAPR	1.05
P78371	CCT2	263	T-complex protein 1 subunit beta	77.593	VDSTAK(1)VAEIEHAEK	1.11

P78371	CCT2	230	T-complex protein 1 subunit beta	129.16	IGVNQPK(1)R	1.12
P78371	CCT2	441	T-complex protein 1 subunit beta	188.87	EAVAMESYAK(1)ALR	1.12
P78371	CCT2	120	T-complex protein 1 subunit beta	108.35	K(1)IHPQTIIAGWR	1.29
P78371	CCT2	181	T-complex protein 1 subunit beta	86.344	DHFTK(1)LAVEAVLR	Tip60 OE only
P78371	CCT2	236	T-complex protein 1 subunit beta	124.07	IENAK(1)ILIANTGMDTDK	Tip60 OE only
P78371	CCT2	50	T-complex protein 1 subunit beta	55.401	GMDK(1)ILLSSGR	unquantifiable
P49368	CCT3	249	T-complex protein 1 subunit gamma	130.01	K(1)GESQTDIEITR	0.88
P49368	CCT3	21	T-complex protein 1 subunit gamma	48.796	K(1)VQSGNINAAK	0.94
P49368	CCT3	367	T-complex protein 1 subunit gamma	83.617	IGDEYFTFITDCK(1)DPK	0.95
P49368	CCT3	15	T-complex protein 1 subunit gamma	138.4	PVLVLSQNTK(1)R	0.96
P49368	CCT3	381	T-complex protein 1 subunit gamma	139.86	GASK(1)EILSEVER	0.96
P49368	CCT3	138	T-complex protein 1 subunit gamma	116.3	ALDDMISTLK(1)K	0.99
P49368	CCT3	248	T-complex protein 1 subunit gamma	123.88	IVLLDSSLEYK(1)K	Tip60 OE only
P49368	CCT3	222	T-complex protein 1 subunit gamma	86.624	GVMINK(1)DVTHPR	Tip60 OE only
P49368	CCT3	128	T-complex protein 1 subunit gamma	76.143	K(1)ALDDMISTLK	unquantifiable
P50991	CCT4	338	T-complex protein 1 subunit delta	106.29	EDIEFICK(1)TIGTK	0.78
P50991	CCT4	139	T-complex protein 1 subunit delta	65.943	GIHPTIISESFQK(1)ALEK	0.8
P50991	CCT4	531	T-complex protein 1 subunit delta	133.81	SILK(1)IDDVVNTR	0.91
P50991	CCT4	395	T-complex protein 1 subunit delta	82.417	GSNK(1)LVIEEAER	0.93
P50991	CCT4	55	T-complex protein 1 subunit delta	60.398	TSLGPK(1)GMDK	0.94
P50991	CCT4	321	T-complex protein 1 subunit delta	76.833	MK(1)IMVIK	0.98
P50991	CCT4	326	T-complex protein 1 subunit delta	103.56	IMVIK(1)DIER	1
P50991	CCT4	65	T-complex protein 1 subunit delta	89.541	MIQDGK(1)GDVTITNDGATILI	1.02
P50991	CCT4	302	T-complex protein 1 subunit delta	142.1	TGCNVLLIQK(1)SILR	1.04
P50991	CCT4	42	T-complex protein 1 subunit delta	142.76	FSNISAAK(1)AVADAIR	1.06
P50991	CCT4	375	T-complex protein 1 subunit delta	73.885	LLK(1)ITGCASPGK	1.09
P50991	CCT4	489	T-complex protein 1 subunit delta	122.51	HAQGEK(1)TAGINVR	1.2
P50991	CCT4	384	T-complex protein 1 subunit delta	116.54	ITGCASPGK(1)TVTIVVR	1.32
P48643	CCT5	284	T-complex protein 1 subunit epsilon	131.62	EK(1)FEEMIQQIK	0.54
P48643	CCT5	259	T-complex protein 1 subunit epsilon	93.821	IAILTCPFEPPK(1)PK	0.76
P48643	CCT5	399	T-complex protein 1 subunit epsilon	116.54	MIIEEAK(1)R	0.91
P48643	CCT5	170	T-complex protein 1 subunit epsilon	98.942	DTEPLIQTAK(1)TTLGSK	0.96
P48643	CCT5	223	T-complex protein 1 subunit epsilon	101.28	LEDTK(1)LIK	0.98
P48643	CCT5	378	T-complex protein 1 subunit epsilon	131.44	MLVIEQCK(1)NSR	1.02

P48643	CCT5	226	T-complex protein 1 subunit epsilon	59.153	LIK(1)GVIVDK	1.14
P48643	CCT5	514	T-complex protein 1 subunit epsilon	70.17	K(1)QQISLATQMVR	2.09
P48643	CCT5	370	T-complex protein 1 subunit epsilon	108.15	DK(1)MLVIEQCK	Tip60 OE only
P48643	CCT5	275	T-complex protein 1 subunit epsilon	69.721	LDVTSVEDYK(1)ALQK	Tip60 OE only
P40227	CCT6A	127	T-complex protein 1 subunit zeta	150.61	IITEGFEAAK(1)EK	0.83
P40227	CCT6A	199	T-complex protein 1 subunit zeta	91.313	HK(1)SETDTSLIR	0.85
P40227	CCT6A	129	T-complex protein 1 subunit zeta	112.36	EK(1)ALQFLEEVK	0.87
P40227	CCT6A	10	T-complex protein 1 subunit zeta	54.982	TLNPK(1)AEVAR	0.92
P40227	CCT6A	365	T-complex protein 1 subunit zeta	154.49	FTFIEK(1)CNNPR	0.95
P40227	CCT6A	138	T-complex protein 1 subunit zeta	132.78	ALQFLEEVK(1)VSR	0.96
P40227	CCT6A	251	T-complex protein 1 subunit zeta	130.01	TEVNSGFFYK(1)SAEER	1.17
P40227	CCT6A	388	T-complex protein 1 subunit zeta	152.54	HTLTQIK(1)DAVR	Tip60 OE only
Q99832	CCT7	430	T-complex protein 1 subunit eta	107.24	TIPGK(1)QQLLIGAYAK	0.77
Q99832	CCT7	144	T-complex protein 1 subunit eta	54.611	EIAVTVK(1)K	0.88
Q99832	CCT7	55	T-complex protein 1 subunit eta	145.91	GK(1)ATISNDGATILK	0.89
Q99832	CCT7	230	T-complex protein 1 subunit eta	104.52	TFSYAGFEMQPK(1)K	0.91
Q99832	CCT7	77	T-complex protein 1 subunit eta	53.773	LLDVVHPAAK(1)TLVDIAK	0.92
Q99832	CCT7	280	T-complex protein 1 subunit eta	53.089	LEK(1)IHHSGAK	0.94
Q99832	CCT7	172	T-complex protein 1 subunit eta	133.86	LISQQK(1)AFFAK	0.96
Q99832	CCT7	137	T-complex protein 1 subunit eta	89.047	IK(1)EIAVTVK	0.98
Q99832	CCT7	135	T-complex protein 1 subunit eta	137.14	TATQLAVNK(1)IK	1
Q99832	CCT7	368	T-complex protein 1 subunit eta	76.478	AK(1)TCTFILR	1.03
Q99832	CCT7	287	T-complex protein 1 subunit eta	41.218	IHHSGAK(1)VVLSK	1.04
Q99832	CCT7	250	T-complex protein 1 subunit eta	114.28	AEK(1)DNAEIR	1.08
Q99832	CCT7	157	T-complex protein 1 subunit eta	59.265	LLEK(1)CAMTALSSK	Tip60 OE only
Q99832	CCT7	366	T-complex protein 1 subunit eta	77.677	YNFFTGCPK(1)AK	Tip60 OE only
P50990	CCT8	296	T-complex protein 1 subunit theta	47.807	AIADTGANVVVTGGK(1)VADN	0.8
P50990	CCT8	367	T-complex protein 1 subunit theta	88.441	HEK(1)EDGAISTIVLR	0.88
P50990	CCT8	224	T-complex protein 1 subunit theta	79.013	ILGSGISSSSVLHGMVFK(1)K	0.92
P50990	CCT8	466	T-complex protein 1 subunit theta	105.39	ANEVISK(1)LYAVHQEGNK	0.92
P50990	CCT8	459	T-complex protein 1 subunit theta	169.09	ALAENSGVK(1)ANEVISK	0.93
P50990	CCT8	235	T-complex protein 1 subunit theta	104.82	ETEGDVTSVK(1)DAK	0.96
P50990	CCT8	37	T-complex protein 1 subunit theta	190.86	NIQACK(1)ELAQTTR	1.01
P50990	CCT8	326	T-complex protein 1 subunit theta	98.048	LCK(1)TVGATALPR	1.04

P50990	CCT8	318	T-complex protein 1 subunit theta	84.498	LNSK(1)WDLR	1.39
P50990	CCT8	152	T-complex protein 1 subunit theta	85.988	AHEILPNLVCCSAK(1)NLR	Tip60 OE only
P50990	CCT8	171	T-complex protein 1 subunit theta	96.103	TSIMSK(1)QYGNEVFLAK	Tip60 OE only
P50990	CCT8	476	T-complex protein 1 subunit theta	63.165	LYAVHQEGNK(1)NVGLDIEAE\	Tip60 OE only
P50990	CCT8	400	T-complex protein 1 subunit theta	132.32	AVDDGVNTFK(1)VLTR	unquantifiable
P08962	CD63	188	CD63 antigen	84.615	AIHK(1)EGCVEK	Tip60 OE only
P21926	CD9	135	CD9 antigen	43.124	TK(1)DEPQR	unquantifiable
Q16543	CDC37	69	Hsp90 co-chaperone Cdc37;Hsp90 co-chaperone	60.434	K(0.088)LK(0.912)ELEVAEGC	0.93
Q16543	CDC37	101	Hsp90 co-chaperone Cdc37;Hsp90 co-chaperone	90.629	SWEQK(1)LEEMR	0.98
Q16543	CDC37	47	Hsp90 co-chaperone Cdc37;Hsp90 co-chaperone	57.559	EK(1)EELDR	1.02
Q16543	CDC37	78	Hsp90 co-chaperone Cdc37;Hsp90 co-chaperone	72.2	ELEVAEGGK(1)AELER	1.24
Q16543	CDC37	121	Hsp90 co-chaperone Cdc37;Hsp90 co-chaperone	46.481	SMPWNVDTLSK(1)DGFSK	Tip60 OE only
P60953	CDC42	153	Cell division control protein 42 homolog	126.19	AVK(1)YVECSALTQK	0.85
P60953	CDC42	163	Cell division control protein 42 homolog	132.91	YVECSALTQK(1)GLK	0.91
Q99459	CDC5L	187	Cell division cycle 5-like protein	99.511	LAALQK(1)R	0.71
Q99459	CDC5L	576	Cell division cycle 5-like protein	72.234	SEELIK(1)K	0.83
Q6P1J9	CDC73	209	Parafibromin	80.596	TDLDDDITALK(1)QR	unquantifiable
P06493	CDK1	56	Cyclin-dependent kinase 1	92.439	EISLLK(1)ELR	1.2
Q9NYV4	CDK12	796	Cyclin-dependent kinase 12	83.397	EIVTDK(1)QDALDFK	Tip60 OE only
Q03701	CEBPZ	323	CCAAT/enhancer-binding protein zeta	95.573	LEQLSSGNK(1)DSR	Tip60 OE only
Q92879	CELF1	101	CUGBP Elav-like family member 1	46.844	PADSEK(1)NNAVEDR	0.89
Q92879	CELF1	30	CUGBP Elav-like family member 1	72.485	TWSEK(1)DLR	1.17
Q92879	CELF1;CELF2	436	CUGBP Elav-like family member 1;CUGBP Elav-lik	107.15	VFIDK(1)QTNLSK	unquantifiable
P23528	CFL1	34	Cofilin-1	91.858	K(1)AVLFCLSEDKK	0.83
P23528	CFL1	78	Cofilin-1	97.033	MLPDK(1)DCR	0.92
P23528	CFL1	44	Cofilin-1	138.4	AVLFCLSEDK(1)K	0.96
P23528	CFL1	45	Cofilin-1	90.601	K(1)NIILEEGK	0.97
P23528	CFL1	144	Cofilin-1	154.28	HELQANCYEEVK(1)DR	1.02
P23528	CFL1	132	Cofilin-1	117.65	LTGIK(1)HELQANCYEEVK	1.13
P23528	CFL1;CFL2	92	Cofilin-1;Cofilin-2	149.96	YALYDATYETK(1)ESK	1
P23528	CFL1;CFL2	19	Cofilin-1;Cofilin-2	101.69	VFNDMK(1)VR	1.04
P23528	CFL1;CFL2;DSTN	114	Cofilin-1;Cofilin-2;Destrin	99.802	SK(1)MIYASSK	1.02
P23528	CFL1;CFL2;DSTN	121	Cofilin-1;Cofilin-2;Destrin	113.22	MIYASSK(1)DAIK	1.24
Q9UFW8	CGGBP1	65	CGG triplet repeat-binding protein 1	73.039	SK(1)THTK(1)R	0.8

Q9UFW8	CGGBP1	69	CGG triplet repeat-binding protein 1	73.039	SK(1)THTK(1)R	0.8
Q9NX63	CHCHD3	76	MICOS complex subunit MIC19	135.55	VAEELALEQAK(1)K	0.78
Q9NX63	CHCHD3	142	MICOS complex subunit MIC19	52.495	K(0.002)QDAFYK(0.998)EQL/	1.13
Q14839	CHD4	192	Chromodomain-helicase-DNA-binding protein 4	77.741	PLIAAK(1)NPK	1.22
Q14839	CHD4	1051	Chromodomain-helicase-DNA-binding protein 4	56.258	ASGK(1)LLLLQK	1.37
Q9HD42	CHMP1A	83	Charged multivesicular body protein 1a	67.153	VQTAVTMK(1)GVTK	1.03
Q9HD42	CHMP1A	107	Charged multivesicular body protein 1a	136.27	ALSTMDLQK(1)VSSVMDR	1.08
Q9HD42	CHMP1A	75	Charged multivesicular body protein 1a	71.176	VDAVASK(1)VQTAVTMK	Tip60 OE only
Q9UHD1	CHORDC1	144	Cysteine and histidine-rich domain-containing pr	56.851	LSSGNEENK(0.173)K(0.827)I	0.84
Q9UHD1	CHORDC1	289	Cysteine and histidine-rich domain-containing pr	84.498	LWGVIDVK(1)R	1.3
Q9Y3Y2	CHTOP	213	Chromatin target of PRMT1 protein	100.19	PVLTK(1)EQLDNQLDAYMSK	Tip60 OE only
Q14011	CIRBP	84	Cold-inducible RNA-binding protein	62.303	VDQAGK(1)SSDNR	Tip60 OE only
Q07065	CKAP4	290	Cytoskeleton-associated protein 4	76.847	ALK(1)EAVK	0.78
Q07065	CKAP4	223	Cytoskeleton-associated protein 4	107.09	DLSDGIHVVK(1)DAR	0.82
Q07065	CKAP4	287	Cytoskeleton-associated protein 4	74.786	VASLEESEGNK(0.161)QDLK((	0.97
Q07065	CKAP4	283	Cytoskeleton-associated protein 4	172.17	VASLEESEGNK(1)QDLK	1.03
Q07065	CKAP4	272	Cytoskeleton-associated protein 4	152.94	AK(1)VASLEESEGNK	1.03
Q07065	CKAP4	405	Cytoskeleton-associated protein 4	121.76	HSEAFEALQQK(1)SQGLDSR	1.04
Q07065	CKAP4	182	Cytoskeleton-associated protein 4	126.71	SSQHK(1)QDLTEK	1.06
Q07065	CKAP4	332	Cytoskeleton-associated protein 4	180.67	ELVSLK(1)QEQQAFK	1.07
Q07065	CKAP4	270	Cytoskeleton-associated protein 4	53.351	EINDMK(1)AK	1.1
Q07065	CKAP4	301	Cytoskeleton-associated protein 4	111.95	EIQTSAK(1)SR	1.18
Q14008	CKAP5	1302	Cytoskeleton-associated protein 5	81.548	VGEPK(1)DVIR	Tip60 OE only
P12277	CKB	298	Creatine kinase B-type	75.416	AGVHIK(1)LPNLGK	0.8
P12277	CKB	307	Creatine kinase B-type	144.77	HEK(1)FSEVLK	0.82
P12277	CKB	304	Creatine kinase B-type	52.725	LPNLGK(1)HEK	0.92
P12277	CKB	242	Creatine kinase B-type	122.52	VISMQK(1)GGNMK	0.95
P12277	CKB	313	Creatine kinase B-type	159.14	FSEVLK(1)R	1.01
O75122	CLASP2;CLASP1	793	CLIP-associating protein 2;CLIP-associating prote	53.554	VK(1)VAILK	unquantifiable
O00299	CLIC1	135	Chloride intracellular channel protein 1	50.12	GLLK(1)ALK	1.02
P30622	CLIP1	1162	CAP-Gly domain-containing linker protein 1	58.916	EIETLK(1)QAAAQK	Tip60 OE only
O96005	CLPTM1	534	Cleft lip and palate transmembrane protein 1	106.4	LK(1)SVAHLPWR	unquantifiable
O96005	CLPTM1	436	Cleft lip and palate transmembrane protein 1	47.082	ITK(1)VMDVR	unquantifiable
P09496	CLTA	136	Clathrin light chain A	91.087	KQEAEWK(1)EK	unquantifiable

Q00610	CLTC	1501	Clathrin heavy chain 1	108.31	LEK(1)HELIEFR	0.96
Q00610	CLTC	367	Clathrin heavy chain 1	131.13	K(1)FNALFAQGNYSEAAK	0.99
Q00610	CLTC	637	Clathrin heavy chain 1	150.04	ALEHFTDLYDIK(1)R	0.99
Q00610	CLTC	78	Clathrin heavy chain 1	72.568	RPISADSAIMNPASK(1)VIALK	1.02
Q00610	CLTC	1609	Clathrin heavy chain 1	70.69	EYLTK(1)VDK	1.04
Q00610	CLTC	1612	Clathrin heavy chain 1	94.692	VDK(1)LDASESLR	1.06
Q00610	CLTC	1331	Clathrin heavy chain 1	72.234	FK(0.001)PQK(0.999)MR	1.1
Q00610	CLTC	619	Clathrin heavy chain 1	143	AHIAQLCEK(1)AGLLQR	1.1
Q00610	CLTC	1254	Clathrin heavy chain 1	102.4	TWK(1)EVCFACVDGK	1.13
Q00610	CLTC	487	Clathrin heavy chain 1	148.96	ANVPNK(1)VIQCFAETGQVQK	1.2
Q00610	CLTC	1118	Clathrin heavy chain 1	58.782	AQLQK(1)GMVK	1.21
Q00610	CLTC	1264	Clathrin heavy chain 1	169.65	EVCFACVDGK(1)EFR	1.3
Q00610	CLTC	907	Clathrin heavy chain 1	63.419	VVGK(1)YCEK	1.41
Q00610	CLTC	1535	Clathrin heavy chain 1	107.09	DSLYK(1)DAMQYASESK	1.53
Q00610	CLTC	1522	Clathrin heavy chain 1	119.27	WK(1)QSVELCK	Tip60 OE only
Q00610	CLTC;CLTCL1	737	Clathrin heavy chain 1;Clathrin heavy chain 2	143.03	YIQAACK(1)TGQIK	0.94
Q00610	CLTC;CLTCL1	83	Clathrin heavy chain 1;Clathrin heavy chain 2	69.383	VIALK(1)AGK	0.97
Q00610	CLTC;CLTCL1	96	Clathrin heavy chain 1;Clathrin heavy chain 2	103.22	TLQIFNIEMK(1)SK	0.98
Q00610	CLTC;CLTCL1	1347	Clathrin heavy chain 1;Clathrin heavy chain 2	94.407	VNIPK(1)VLR	0.98
Q00610	CLTC;CLTCL1	506	Clathrin heavy chain 1;Clathrin heavy chain 2	62.842	IVLYAK(1)K	1
Q00610	CLTC;CLTCL1	1392	Clathrin heavy chain 1;Clathrin heavy chain 2	73.233	EGQFK(1)DIITK	1.01
Q00610	CLTC;CLTCL1	742	Clathrin heavy chain 1;Clathrin heavy chain 2	113.71	TGQIK(1)EVER	1.15
Q00610	CLTC;CLTCL1	456	Clathrin heavy chain 1;Clathrin heavy chain 2	83.617	EDK(1)LECSEELGDLVK	Tip60 OE only
Q00610	CLTC;CLTCL1	851	Clathrin heavy chain 1;Clathrin heavy chain 2	103.75	GQFSTDELVAEVEK(1)R	unquantifiable
Q96DG6	CMBL	36	Carboxymethylenebutenolidase homolog	40.715	AYVTK(1)SPVDAGK	0.61
Q96DG6	CMBL	193	Carboxymethylenebutenolidase homolog	101.25	DVSLLTQK(1)LK	1.02
P62633	CNBP	8	Cellular nucleic acid-binding protein	48.283	SSNECFK(1)CGR	unquantifiable
Q15417	CNN3	17	Calponin-3	78.655	GPSYGLSAEVK(1)NK	Tip60 OE only
A5YKK6	CNOT1	877	CCR4-NOT transcription complex subunit 1	44.767	FK(1)DSTIK	0.89
Q9Y2B0	CNPY2	90	Protein canopy homolog 2	60.549	MK(1)EYGEQIDPSTHR	0.82
Q9Y2B0	CNPY2	160	Protein canopy homolog 2	45.68	EADNVK(1)DK	0.98
A6NMZ7	COL6A6	2216	Collagen alpha-6(VI) chain	47.067	EDVLQK(1)AK	unquantifiable
Q8NBJ5	COLGALT1	445	Procollagen galactosyltransferase 1	103.29	FEIFFK(1)R	1.31
P53621	COPA	1028	Coatomer subunit alpha;Xenin;Proxenin	124.98	FEEAVEK(1)FR	1.03

P53621	COPA	487	Coatomer subunit alpha;Xenin;Proxenin	65.224	TLASVK(1)ISK	1.12
P53621	COPA	600	Coatomer subunit alpha;Xenin;Proxenin	94.407	FK(1)LALINR	1.16
P53621	COPA	671	Coatomer subunit alpha;Xenin;Proxenin	86.136	ALDDK(1)NCWEK	1.26
P53621	COPA	480	Coatomer subunit alpha;Xenin;Proxenin	78.342	DADSITLFDVQQK(1)R	Tip60 OE only
P53621	COPA	411	Coatomer subunit alpha;Xenin;Proxenin	97.456	DADSQNPDAPEGK(1)R	Tip60 OE only
P53618	COPB1	651	Coatomer subunit beta	65.305	LEEEK(1)LSQK	Tip60 OE only
P53618	COPB1	494	Coatomer subunit beta	59.198	SLGEIPIVESEIK(1)K	unquantifiable
P35606	COPB2	64	Coatomer subunit beta'	52.683	AAK(1)FVAR	0.65
Q9Y678	COPG1	414	Coatomer subunit gamma-1	83.265	EEGGFEYK(1)R	0.99
Q9Y678	COPG1;COPG2	145	Coatomer subunit gamma-1;Coatomer subunit g	133.86	YMK(1)QAIVDK	1.06
P61201	COPS2	64	COP9 signalosome complex subunit 2	75.78	VLELEGEK(1)GEWGFK	Tip60 OE only
Q7L5N1	COPS6	102	COP9 signalosome complex subunit 6	71.016	IIIDK(1)EYYYTK	Tip60 OE only
Q9BR76	CORO1B	400	Coronin-1B	73.665	EAYVPSK(1)QR	Tip60 OE only
Q9ULV4	CORO1C	212	Coronin-1C	104.22	KQEIVAEK(1)EK	0.98
Q9ULV4	CORO1C	452	Coronin-1C	143.96	SIK(1)DTICNQDER	1
Q9ULV4	CORO1C	329	Coronin-1C	91.867	GLDVNK(1)CEIAR	1.22
P13073	COX4I1	60	Cytochrome c oxidase subunit 4 isoform 1, mito	117.53	HLSASQK(1)ALK	0.89
P13073	COX4I1	87	Cytochrome c oxidase subunit 4 isoform 1, mito	93.178	FK(1)ESFAEMNR	0.98
P13073	COX4I1	53	Cytochrome c oxidase subunit 4 isoform 1, mito	73.953	DHPLPEVAHVK(1)HLSASQK	1
P13073	COX4I1	67	Cytochrome c oxidase subunit 4 isoform 1, mito	114.86	EK(1)ASWSSLSMDEK	1.43
O75131	CPNE3	118	Copine-3	58.815	PLVMK(1)TGR	1.16
P23786	CPT2	433	Carnitine O-palmitoyltransferase 2, mitochondri	50.607	EK(1)FDATMK	Tip60 OE only
P23786	CPT2	431	Carnitine O-palmitoyltransferase 2, mitochondri	62.823	TGITAAK(1)EK	unquantifiable
P29373	CRABP2	93	Cellular retinoic acid-binding protein 2	51.268	WESENK(1)MVCEQK	unquantifiable
O75390	CS	321	Citrate synthase, mitochondrial	73.985	EVGK(1)DVSDEK	0.93
O75390	CS	215	Citrate synthase, mitochondrial	73.931	LPCVAAK(1)IYR	0.96
O75390	CS	327	Citrate synthase, mitochondrial	91.906	DVSDEK(1)LR	1.11
O75390	CS	450	Citrate synthase, mitochondrial	85.355	PK(1)SMSTEGLMK	1.16
O75390	CS	459	Citrate synthase, mitochondrial	47.712	SMSTEGLMK(1)FVDSK	1.39
P55060	CSE1L	832	Exportin-2	64.121	IIIPEIQK(1)VSGNVEK	0.81
P55060	CSE1L	425	Exportin-2	77.923	NPSVNWK(1)HK	Tip60 OE only
P55060	CSE1L	481	Exportin-2	76.262	SANVNEFPVLK(1)ADGIK	Tip60 OE only
P55060	CSE1L	165	Exportin-2	41.704	HEFK(1)SNELWTEIK	Tip60 OE only
P55060	CSE1L	782	Exportin-2	86.833	LQNSK(1)TTK	Tip60 OE only

Q12996	CSTF3	119	Cleavage stimulation factor subunit 3	43.246	LPSYK(1)EK	1
Q12996	CSTF3	426	Cleavage stimulation factor subunit 3	60.799	DK(1)SVAFK	1.63
Q12996	CSTF3	274	Cleavage stimulation factor subunit 3	52.49	TEDQTLITK(1)R	Tip60 OE only
Q13363	CTBP1;CTBP2	280	C-terminal-binding protein 1;C-terminal-binding	83.783	ALAQALK(1)EGR	unquantifiable
P35221	CTNNA1	695	Catenin alpha-1	135.99	IAEQVASFQEEK(1)SK	0.72
P35221	CTNNA1	163	Catenin alpha-1	84.743	VVEDGILK(1)LR	1.31
Q8WYA6	CTNNBL1	437	Beta-catenin-like protein 1	123.86	FTENDSEK(1)VDR	0.97
Q8WYA6	CTNNBL1	463	Beta-catenin-like protein 1	77.379	IEGEK(1)HDMVR	unquantifiable
P17812	CTPS1	286	CTP synthase 1	62.717	WK(1)EMADR	1.24
Q6PD62	CTR9	393	RNA polymerase-associated protein CTR9 homolo	90.653	ILGSLYAASEDQEK(1)R	unquantifiable
P07339	CTSD	357	Cathepsin D;Cathepsin D light chain;Cathepsin D	52.555	LSPEDYTLK(1)VSQAGK	0.7
Q14247	CTTN	208	Src substrate cortactin	53.569	VDK(1)SAVGFEYQGK	0.97
Q14247	CTTN	110	Src substrate cortactin	80.469	LSK(1)HCSQVDSVR	1.19
Q13616	CUL1	337	Cullin-1	60.518	IQDGLGELK(1)K	1.47
Q13617	CUL2	428	Cullin-2	66.621	YIDDK(1)DVFQK	Tip60 OE only
Q13618	CUL3	444	Cullin-3	78.908	LLTNK(1)SVSDDSEK	1.02
Q13620	CUL4B	576	Cullin-4B	63.408	AGNK(1)EATDEELEK	0.99
Q13620	CUL4B	403	Cullin-4B	104.79	LYAAEGQK(1)LMQER	1.78
Q13948	CUX1	226	Protein CASP;Homeobox protein cut-like 1	45.161	TELFDLK(1)TK	Tip60 OE only
P19875	CXCL2	99	C-X-C motif chemokine 2;GRO-beta(5-73)	62.842	IIEK(1)MLK	unquantifiable
O43169	CYB5B	34	Cytochrome b5 type B	90.986	LEEVAK(1)R	unquantifiable
P00387	CYB5R3	154	NADH-cytochrome b5 reductase 3;NADH-cytochr	105.4	GPSGLLVYQGK(1)GK	1.15
P99999	CYCS	100	Cytochrome c	42.109	ADLIAYLK(1)K	0.89
P99999	CYCS	87	Cytochrome c	64.803	MIFVGIK(1)K	1.12
P14868	DARS	453	Aspartate--tRNA ligase, cytoplasmic	72.23	IK(1)AYIDSFR	0.66
Q6PI48	DARS2	454	Aspartate--tRNA ligase, mitochondrial	71.342	ACSLLGK(1)LR	unquantifiable
Q96EP5	DAZAP1	57	DAZ-associated protein 1	72.234	GFGFVK(1)FK	Tip60 OE only
P07108	DBI	77	Acyl-CoA-binding protein	101.64	AYINK(1)VEELK	unquantifiable
Q13561	DCTN2	125	Dynactin subunit 2	123.86	TTVK(1)ESATEEK	0.92
Q13561	DCTN2	297	Dynactin subunit 2	103.46	HK(1)ASVEDADTQSK	0.99
Q13561	DCTN2	102	Dynactin subunit 2	105.12	ETPQQK(1)YQR	1.14
Q13561	DCTN2	229	Dynactin subunit 2	94.407	VAELEK(1)R	1.15
Q13561	DCTN2	132	Dynactin subunit 2	65.574	ESATEEK(1)LTPVLLAK	Tip60 OE only
Q7Z4W1	DCXR	17	L-xylulose reductase	64.04	VLVTGAGK(1)GIGR	unquantifiable

Q16531	DDB1	864	DNA damage-binding protein 1	98.233	LQTVAEK(1)EVK	0.96
Q16531	DDB1	897	DNA damage-binding protein 1	131.44	LYEWTTEK(1)ELR	Tip60 OE only
Q16531	DDB1	191	DNA damage-binding protein 1	65.224	HVK(1)TYEVSLR	Tip60 OE only
P30046	DDT;DDTL	21	D-dopachrome decarboxylase;D-dopachrome de	72.789	VPAGLEK(1)R	1.16
Q92499	DDX1	358	ATP-dependent RNA helicase DDX1	113.77	LDDLVSTGK(1)LNLSQVR	0.95
Q92499	DDX1	493	ATP-dependent RNA helicase DDX1	119.17	ILK(1)GEYAVR	1.07
Q92499	DDX1	268	ATP-dependent RNA helicase DDX1	53.909	APDGYIVK(1)SQHSGNAQVTQ	1.57
Q92499	DDX1	288	ATP-dependent RNA helicase DDX1	79.82	FLPNAPK(1)ALIVEPSR	Tip60 OE only
Q92499	DDX1	317	ATP-dependent RNA helicase DDX1	107.43	YIDNPK(1)LR	unquantifiable
Q92841	DDX17	132	Probable ATP-dependent RNA helicase DDX17	77.42	FEK(1)NFYVEHPEVAR	0.89
Q92841	DDX17	428	Probable ATP-dependent RNA helicase DDX17	117.04	TIIFVETK(1)R	1.08
Q92841	DDX17	313	Probable ATP-dependent RNA helicase DDX17	158.79	LIDFLESGK(1)TNLR	1.09
Q92841	DDX17	468	Probable ATP-dependent RNA helicase DDX17	51.211	SGK(1)APILIATDVASR	1.15
Q92841	DDX17	528	Probable ATP-dependent RNA helicase DDX17	42.083	GTAYTFFTPGNLK(1)QAR	1.2
Q9NVP1	DDX18	224	ATP-dependent RNA helicase DDX18	91.549	DLLAAAK(1)TGSGK	Tip60 OE only
Q9NR30	DDX21	678	Nucleolar RNA helicase 2	84.566	GK(1)LGVCFDVPTASVTEIQEK	0.89
Q9NR30	DDX21	668	Nucleolar RNA helicase 2	128.17	EQLGEEIDSK(1)VK	0.91
Q9NR30	DDX21	224	Nucleolar RNA helicase 2	73.722	TFHHVYSGK(1)DLIAQAR	0.97
Q9NR30	DDX21	658	Nucleolar RNA helicase 2	133.89	ELK(1)EQLGEEIDSK	1.01
Q9NR30	DDX21	676	Nucleolar RNA helicase 2	50.966	GMVFLK(1)GK	Tip60 OE only
Q9NR30	DDX21	285	Nucleolar RNA helicase 2	63.565	DFSDITK(1)K	unquantifiable
Q9NR30	DDX21	448	Nucleolar RNA helicase 2	46.158	TIIFCETK(1)K	unquantifiable
Q9NR30	DDX21;DDX50	481	Nucleolar RNA helicase 2;ATP-dependent RNA hel	79.597	EITLK(1)GFR	0.91
Q9BUQ8	DDX23	326	Probable ATP-dependent RNA helicase DDX23	59.35	FYGDLMEK(1)R	1.19
Q9BUQ8	DDX23	166	Probable ATP-dependent RNA helicase DDX23	58.604	FLSK(1)AER	1.37
Q9UHL0	DDX25	105	ATP-dependent RNA helicase DDX25	113.14	LK(1)EELLK	Tip60 OE only
Q96GQ7	DDX27	641	Probable ATP-dependent RNA helicase DDX27	112.13	GK(1)EAVVQEPER	Tip60 OE only
Q13838	DDX39A;DDX39B	268	ATP-dependent RNA helicase DDX39A;Spliceosor	134.98	LTLHGLQQYYVK(1)LK	1.21
Q13838	DDX39A;DDX39B	384	ATP-dependent RNA helicase DDX39A;Spliceosor	59.607	FGTK(1)GLAITFVSDENDAK	Tip60 OE only
Q13838	DDX39B	36	Spliceosome RNA helicase DDX39B	68.944	DVK(1)GSYVSIHSSGFR	1.03
Q13838	DDX39B	191	Spliceosome RNA helicase DDX39B	116.77	HIK(1)HFILDECDK	1.03
O00571	DDX3X	215	ATP-dependent RNA helicase DDX3X	93.162	HAIPIIK(1)EK	1.02
O00571	DDX3X;DDX3Y	491	ATP-dependent RNA helicase DDX3X;ATP-depend	63.091	SGK(1)SPILVATAVAAR	1.09
Q9UJV9	DDX41	441	Probable ATP-dependent RNA helicase DDX41	79.693	TPPPVLIFAEK(1)K	unquantifiable

Q9H0S4	DDX47	161	Probable ATP-dependent RNA helicase DDX47	52.891	LIDHLENTK(1)GFNLR	Tip60 OE only
P17844	DDX5	343	Probable ATP-dependent RNA helicase DDX5	50.108	ENK(1)TIVFVETK	0.75
P17844	DDX5	80	Probable ATP-dependent RNA helicase DDX5	79.469	SK(1)EITVR	0.92
P17844	DDX5	236	Probable ATP-dependent RNA helicase DDX5	146.11	LIDFLECGK(1)TNLR	0.99
P17844	DDX5	340	Probable ATP-dependent RNA helicase DDX5	121.6	LMEEIMSEK(1)ENK	1
P17844	DDX5	391	Probable ATP-dependent RNA helicase DDX5	53.252	HGK(1)APILIATDVASR	1.02
P17844	DDX5	351	Probable ATP-dependent RNA helicase DDX5	110.12	TIVFVETK(1)R	1.26
Q92841	DDX5;DDX17	284	Probable ATP-dependent RNA helicase DDX5;Pro	96.342	STCIYGGAPK(1)GPQIR	1
Q92841	DDX5;DDX17	341	Probable ATP-dependent RNA helicase DDX5;Pro	50.284	K(1)IVDQIRPDR	1.2
Q92841	DDX5;DDX17	274	Probable ATP-dependent RNA helicase DDX5;Pro	114.5	LK(1)STCIYGGAPK	1.3
Q9Y2R4	DDX52	131	Probable ATP-dependent RNA helicase DDX52	117.89	LTSGK(1)LENLR	3.01
P26196	DDX6	104	Probable ATP-dependent RNA helicase DDX6	103.55	GNEFEDYCLK(1)R	1.04
P35659	DEK	84	Protein DEK	82.645	EPFTIAQGK(1)GQK	0.85
P35659	DEK	87	Protein DEK	123.98	GQK(1)LCEIER	0.9
Q7Z401	DENND4A	1349	C-myc promoter-binding protein	58.37	FTMYTTSSK(1)DQSSDR	Tip60 OE only
O43583	DENR	119	Density-regulated protein	63.48	VTIAK(1)IPR	1.23
O43143	DHX15	515	Pre-mRNA-splicing factor ATP-dependent RNA he	93.448	SNLGSVVLQLK(1)K	0.98
O43143	DHX15	230	Pre-mRNA-splicing factor ATP-dependent RNA he	113.93	FEDCSSAK(1)TILK	0.99
O43143	DHX15	143	Pre-mRNA-splicing factor ATP-dependent RNA he	60.434	LQLPVWEYK(1)DR	1.13
O43143	DHX15	614	Pre-mRNA-splicing factor ATP-dependent RNA he	114.78	AADEAK(1)MR	1.21
O43143	DHX15	132	Pre-mRNA-splicing factor ATP-dependent RNA he	41.502	YYDILK(1)K	unquantifiable
Q08211	DHX9	193	ATP-dependent RNA helicase A	58.917	EK(1)IQGEYK	0.77
Q08211	DHX9	266	ATP-dependent RNA helicase A	75.294	K(0.092)K(0.908)EGETVEPYK	0.81
Q08211	DHX9	146	ATP-dependent RNA helicase A	117.89	GANLK(1)DYYSR	0.85
Q08211	DHX9	857	ATP-dependent RNA helicase A	76.221	ILAK(1)LPIEPR	0.91
Q08211	DHX9	199	ATP-dependent RNA helicase A	116.86	IQGEYK(1)YTQVGPDHNR	1.07
Q08211	DHX9	467	ATP-dependent RNA helicase A	134.57	GEEPGK(1)SCGYSVR	1.26
Q08211	DHX9	236	ATP-dependent RNA helicase A	63.408	K(1)LAAQSCALSLVR	Tip60 OE only
Q9NR28	DIABLO	146	Diablo homolog, mitochondrial	112.13	AEMTSK(1)HQEYLK	unquantifiable
O60610	DIAPH1	480	Protein diaphanous homolog 1	43.308	SEAK(1)AAELEK	Tip60 OE only
O60610	DIAPH1	202	Protein diaphanous homolog 1	100.4	LHDEK(1)EETAGSYDSR	unquantifiable
Q9Y2L1	DIS3	809	Exosome complex exonuclease RRP44	73.157	HK(1)LADICK	Tip60 OE only
Q9Y2L1	DIS3	815	Exosome complex exonuclease RRP44	81.594	LADICK(1)NLNFR	Tip60 OE only
O60832	DKC1	144	H/ACA ribonucleoprotein complex subunit 4	106.42	LVK(1)SQQSAGK	1.03

O60832	DKC1	151	H/ACA ribonucleoprotein complex subunit 4	101.95	SQQSAGK(1)EYVGIVR	Tip60 OE only
P10515	DLAT	362	Dihydrolipoyllysine-residue acetyltransferase cor	51.854	VFVSPLAK(1)K	0.8
P09622	DLD	159	Dihydrolipoyl dehydrogenase, mitochondrial	95.523	ITGK(1)NQVTATK	0.89
P09622	DLD	127	Dihydrolipoyl dehydrogenase, mitochondrial	138.1	MMEQK(1)STAVK	0.92
P36957	DLST	267	Dihydrolipoyllysine-residue succinyltransferase c	68.383	HK(1)EAFLK	Tip60 OE only
P31689	DNAJA1	130	DnaJ homolog subfamily A member 1	61.78	LALQK(1)NVICDK	0.95
Q99615	DNAJC7	390	DnaJ homolog subfamily C member 7	46.408	ILGVDK(0.999)NASEDEIK(0.(	0.75
Q99615	DNAJC7	245	DnaJ homolog subfamily C member 7	100.38	MAPDHEK(1)ACIACR	0.96
Q99615	DNAJC7	155	DnaJ homolog subfamily C member 7	140.09	IAETDFEK(1)R	1.14
O75937	DNAJC8	108	DnaJ homolog subfamily C member 8	97.965	AYK(1)LLLDQEQK	0.95
O75937	DNAJC8	40	DnaJ homolog subfamily C member 8	105.86	DSVLTSK(1)NQIER	1.06
O75937	DNAJC8	176	DnaJ homolog subfamily C member 8	60.973	LFAELEIK(1)R	1.08
P50570	DNM2	299	Dynamin-2	50.648	SK(1)LQSQLLSLEK	Tip60 OE only
Q14919	DRAP1	52	Dr1-associated corepressor	57.859	K(1)ACQVTQSR	unquantifiable
Q9Y295	DRG1	213	Developmentally-regulated GTP-binding protein	74.786	SILAEYK(1)IHNADVTLR	0.6
P15924	DSP	1552	Desmoplakin	92.439	TVK(1)DQDITR	unquantifiable
P15924	DSP	1228	Desmoplakin	99.013	EISMQK(1)EDDSK	unquantifiable
P33316	DUT	179	Deoxyuridine 5'-triphosphate nucleotidohydrola	52.509	SGLAAK(1)HFIDVGAGVIDEDY	unquantifiable
Q14204	DYNC1H1	4406	Cytoplasmic dynein 1 heavy chain 1	114.86	TVENIK(1)DPLFR	0.87
Q14204	DYNC1H1	1912	Cytoplasmic dynein 1 heavy chain 1	64.798	LGGSPFGPAGTGK(1)TESVK	0.91
Q14204	DYNC1H1	3209	Cytoplasmic dynein 1 heavy chain 1	81.475	IK(1)ETVDQVEELR	1.01
Q14204	DYNC1H1	1610	Cytoplasmic dynein 1 heavy chain 1	89.698	LADLLGK(1)IQK	1.02
Q14204	DYNC1H1	2094	Cytoplasmic dynein 1 heavy chain 1	61.435	ALK(1)SVLVSAGNVK	1.02
Q14204	DYNC1H1	3727	Cytoplasmic dynein 1 heavy chain 1	67.08	AERPDVDEK(1)R	1.09
Q14204	DYNC1H1	3491	Cytoplasmic dynein 1 heavy chain 1	85.161	WEK(1)TSETFK	1.1
Q14204	DYNC1H1	3471	Cytoplasmic dynein 1 heavy chain 1	121.73	ADLAAVEAK(1)VNR	1.1
Q14204	DYNC1H1	624	Cytoplasmic dynein 1 heavy chain 1	41.54	FK(1)VQYPQSQACK	Tip60 OE only
Q13409	DYNC1I2	55	Cytoplasmic dynein 1 intermediate chain 2	107.18	EAVAPVQEESDLEK(1)K	1.17
Q13409	DYNC1I2	255	Cytoplasmic dynein 1 intermediate chain 2	91.855	DLEDK(1)EGEIQAGAK	Tip60 OE only
Q96FJ2	DYNLL2;DYNLL1	49	Dynein light chain 2, cytoplasmic;Dynein light ch	108.56	K(1)YNPTWHCIVGR	unquantifiable
Q13011	ECH1	276	Delta(3,5)-Delta(2,4)-dienoyl-CoA isomerase, mit	90.614	SPVAVQSTK(1)VNLLYSR	0.86
Q13011	ECH1	95	Delta(3,5)-Delta(2,4)-dienoyl-CoA isomerase, mit	102.06	EMVECFNK(1)ISR	0.95
P30084	ECHS1	204	Enoyl-CoA hydratase, mitochondrial	144.28	ISAQDAK(1)QAGLVSK	0.91
P42126	ECI1	89	Enoyl-CoA delta isomerase 1, mitochondrial	133.6	LENDK(1)SFR	0.98

P42126	ECI1	61	Enoyl-CoA delta isomerase 1, mitochondrial	53.569	VLVEPDAGAGVAVMK(1)FK	Tip60 OE only
Q5VYK3	ECM29	315	Proteasome-associated protein ECM29 homolog	49.333	TK(1)EGAVLKPELK	0.9
Q5VYK3	ECM29	1324	Proteasome-associated protein ECM29 homolog	57.019	ATEQEK(1)AAMDSAR	1
O60869	EDF1	98	Endothelial differentiation-related factor 1	45.88	DLATK(0.981)INEK(0.019)PC	0.78
O60869	EDF1	131	Endothelial differentiation-related factor 1	114.67	AIGLK(1)LR	0.94
O60869	EDF1	23	Endothelial differentiation-related factor 1	74.173	GPTAAQAK(1)SK	1.1
Q15075	EEA1	1178	Early endosome antigen 1	90.108	LELQGK(1)ADSLK	0.62
Q15075	EEA1	178	Early endosome antigen 1	44.395	SK(1)YDEER	0.77
P68104	EEF1A1;EEF1A1P5	439	Elongation factor 1-alpha 1;Putative elongation f	100.93	QTVAVGVIK(1)AVDK	0.85
P68104	EEF1A1;EEF1A1P5	392	Elongation factor 1-alpha 1;Putative elongation f	102.06	LEDGPK(1)FLK	0.88
P68104	EEF1A1;EEF1A1P5	179	Elongation factor 1-alpha 1;Putative elongation f	68.787	EVSTYIK(1)K	0.97
P68104	EEF1A1;EEF1A1P5	172	Elongation factor 1-alpha 1;Putative elongation f	138.71	YEEIVK(1)EVSTYIK	0.97
P68104	EEF1A1;EEF1A1P5	395	Elongation factor 1-alpha 1;Putative elongation f	69.864	FLK(1)SGDAAIVDMVPGK	0.97
P68104	EEF1A1;EEF1A1P5	84	Elongation factor 1-alpha 1;Putative elongation f	104.21	FETSK(1)YYVTIIDAPGHR	1.02
P68104	EEF1A1;EEF1A1P5	386	Elongation factor 1-alpha 1;Putative elongation f	76.134	K(1)LEDGPK	Tip60 OE only
P68104	EEF1A1;EEF1A1P5;EE	41	Elongation factor 1-alpha 1;Putative elongation f	88.942	TIEK(1)FEK	0.95
P68104	EEF1A1;EEF1A1P5;EE	44	Elongation factor 1-alpha 1;Putative elongation f	107.15	FEK(1)EAAEMGK	0.96
P68104	EEF1A1;EEF1A1P5;EE	255	Elongation factor 1-alpha 1;Putative elongation f	275.83	LPLQDVYK(1)IGGIGTVPVGR	0.99
P68104	EEF1A1;EEF1A1P5;EE	376	Elongation factor 1-alpha 1;Putative elongation f	67.032	FAELK(1)EK	1.22
P24534	EEF1B2	185	Elongation factor 1-beta	61.999	LVPVGYGIK(1)K	0.99
P24534	EEF1B2	129	Elongation factor 1-beta	111.82	LAQYESK(1)K	unquantifiable
P24534	EEF1B2;EEF1D	133	Elongation factor 1-beta;Elongation factor 1-delt	60.799	K(1)PALVAK	0.98
P29692	EEF1D	17	Elongation factor 1-delta	61.999	FK(1)YDDAER	0.93
P29692	EEF1D	15	Elongation factor 1-delta	60.788	IWFDK(1)FK	Tip60 OE only
P26641	EEF1G	227	Elongation factor 1-gamma	76.1	FAETQPK(1)K	0.67
P26641	EEF1G	277	Elongation factor 1-gamma	54.286	AK(1)DPFAHLPK	0.71
P26641	EEF1G	401	Elongation factor 1-gamma	113.22	K(1)LDPGSEETQTLVR	0.87
P26641	EEF1G	241	Elongation factor 1-gamma	58.676	EEK(1)QKPQAER	0.88
P26641	EEF1G	212	Elongation factor 1-gamma	136.63	LCEK(1)MAQFDAK	0.99
P26641	EEF1G	132	Elongation factor 1-gamma	155.42	QATENAK(1)EEVR	1
P26641	EEF1G	294	Elongation factor 1-gamma	186.3	STFVLDEFK(1)R	1
P26641	EEF1G	434	Elongation factor 1-gamma	93.262	AFNQGK(1)IFK	1.04
P26641	EEF1G	219	Elongation factor 1-gamma	114.6	MAQFDAK(1)K	1.17
P26641	EEF1G	285	Elongation factor 1-gamma	47.397	AK(0.15)DPFAHLPK(0.85)STI	Tip60 OE only

P26641	EEF1G	147	Elongation factor 1-gamma	219.7	ILGLLDAYLK(1)TR	unquantifiable
P26641	EEF1G	220	Elongation factor 1-gamma	71.379	K(1)FAETQPK	unquantifiable
P13639	EEF2	571	Elongation factor 2	112.36	DLEEDHACIPIK(1)K	0.64
P13639	EEF2	638	Elongation factor 2	99.752	YLAEK(1)YEWDVAEAR	0.8
P13639	EEF2	594	Elongation factor 2	190.1	ETVSEESNVLCLSK(1)SPNK	0.8
P13639	EEF2	314	Elongation factor 2	154.24	KEETAK(1)LIEK	0.8
P13639	EEF2	439	Elongation factor 2	120.12	K(1)EDLYLKPIQR	0.96
P13639	EEF2	308	Elongation factor 2	139.19	VFDAIMNFK(1)K	0.97
P13639	EEF2	322	Elongation factor 2	135.8	LDIK(1)LDSEDK	0.99
P13639	EEF2	328	Elongation factor 2	96.492	LDSEDK(1)DK	1
P13639	EEF2	512	Elongation factor 2	99.522	VAVEAK(1)NPADLPK	1
P13639	EEF2	318	Elongation factor 2	83.206	LIEK(1)LDIK	1.01
P13639	EEF2	42	Elongation factor 2	116.84	STLTDSLVCK(1)AGIIASAR	1.02
P13639	EEF2	426	Elongation factor 2	98.254	VFSGLVSTGLK(1)VR	1.03
P13639	EEF2	258	Elongation factor 2	76.478	VEDMMK(1)K	1.03
P13639	EEF2	159	Elongation factor 2	85.522	IKPVLMMNK(1)MDR	1.06
P13639	EEF2	32	Elongation factor 2	106.45	NMSVIAHVDHGK(1)STLTDSL	1.06
P13639	EEF2	239	Elongation factor 2	174.42	FAAK(1)GEGQLGPAER	1.07
P13639	EEF2	152	Elongation factor 2	79.469	IK(1)PVLMMNK	1.08
P13639	EEF2	337	Elongation factor 2	128.75	PLLK(1)AVMR	1.08
P13639	EEF2	275	Elongation factor 2	129.85	FSK(1)SATSPEGK	1.12
P13639	EEF2	283	Elongation factor 2	69.628	SATSPEGK(1)K	1.44
Q15029	EFTUD2	244	116 kDa U5 small nuclear ribonucleoprotein con	51.762	LIK(1)HAVQER	0.94
P47813	EIF1AX	56	Eukaryotic translation initiation factor 1A, X-chr	132.84	LEAMCFDGVK(1)R	1.17
P47813	EIF1AX;EIF1AY	94	Eukaryotic translation initiation factor 1A, X-chr	113.47	ADVILK(1)YNADEAR	0.9
P47813	EIF1AX;EIF1AY	88	Eukaryotic translation initiation factor 1A, X-chr	129.16	DYQDNK(1)ADVILK	0.96
P47813	EIF1AX;EIF1AY	29	Eukaryotic translation initiation factor 1A, X-chr	140.14	ELVFK(1)EDGQEYAQVIK	1.07
Q9BY44	EIF2A	551	Eukaryotic translation initiation factor 2A;Eukary	88.187	AIEQLK(1)EQAATGK	0.9
Q14232	EIF2B1	145	Translation initiation factor eIF-2B subunit alpha	105.57	VLEAAVAAK(1)K	0.89
Q14232	EIF2B1	253	Translation initiation factor eIF-2B subunit alpha	54.157	YK(1)ADTLK	1.06
Q14232	EIF2B1	258	Translation initiation factor eIF-2B subunit alpha	71.555	ADTLK(1)VAQTGQDLK	Tip60 OE only
P05198	EIF2S1	190	Eukaryotic translation initiation factor 2 subunit	91.041	LTPQAVK(1)IR	0.97
P05198	EIF2S1	97	Eukaryotic translation initiation factor 2 subunit	46.706	VSPEEAIK(1)CEDK	Tip60 OE only
P20042	EIF2S2	190	Eukaryotic translation initiation factor 2 subunit	57.348	EK(1)NPDMVAGEK	0.85

Q2VIR3	EIF2S3L;EIF2S3	426	Putative eukaryotic translation initiation factor 2	112.72	ADLGK(1)IVLTNPVCTEVGEK	0.84
Q2VIR3	EIF2S3L;EIF2S3	421	Putative eukaryotic translation initiation factor 2	80.755	VSAVK(1)ADLGK	0.92
Q2VIR3	EIF2S3L;EIF2S3	183	Putative eukaryotic translation initiation factor 2	81.594	LK(1)HILILQNK	0.99
Q2VIR3	EIF2S3L;EIF2S3	191	Putative eukaryotic translation initiation factor 2	135.99	HILILQNK(1)IDLVK	1.16
Q2VIR3	EIF2S3L;EIF2S3	303	Putative eukaryotic translation initiation factor 2	60.518	PGIVSK(1)DSEGK	1.61
Q14152	EIF3A	747	Eukaryotic translation initiation factor 3 subunit	62.842	ALEHK(1)NR	1.04
Q14152	EIF3A	100	Eukaryotic translation initiation factor 3 subunit	110.87	MAEEK(1)TEAAK	1.06
Q14152	EIF3A	720	Eukaryotic translation initiation factor 3 subunit	151.1	IK(1)DMDLWEQQEEER	1.19
Q14152	EIF3A	651	Eukaryotic translation initiation factor 3 subunit	53.166	TELGAK(1)AFK	Tip60 OE only
Q14152	EIF3A	694	Eukaryotic translation initiation factor 3 subunit	79.597	K(1)IDYFER	Tip60 OE only
Q14152	EIF3A	68	Eukaryotic translation initiation factor 3 subunit	104.82	SHLAK(1)EGLYQYK	unquantifiable
P55884	EIF3B	204	Eukaryotic translation initiation factor 3 subunit	53.163	LK(1)NVIHK	0.9
P55884	EIF3B	257	Eukaryotic translation initiation factor 3 subunit	67.897	LDK(1)QHTFR	0.91
P55884	EIF3B	436	Eukaryotic translation initiation factor 3 subunit	42.395	WSHDGK(1)FFAR	0.93
P55884	EIF3B	734	Eukaryotic translation initiation factor 3 subunit	97.068	IFEQK(1)DR	0.97
P55884	EIF3B	744	Eukaryotic translation initiation factor 3 subunit	50.607	ASK(1)ELVER	1.07
Q99613	EIF3C;EIF3CL	742	Eukaryotic translation initiation factor 3 subunit	111.61	EHVVAASK(1)AMK	0.84
Q99613	EIF3C;EIF3CL	643	Eukaryotic translation initiation factor 3 subunit	171.36	AK(1)ELLGQGLLLR	0.97
Q99613	EIF3C;EIF3CL	94	Eukaryotic translation initiation factor 3 subunit	76.774	AK(1)SIVDK	1
Q99613	EIF3C;EIF3CL	558	Eukaryotic translation initiation factor 3 subunit	94.407	YIYAK(1)DR	1.07
Q99613	EIF3C;EIF3CL	493	Eukaryotic translation initiation factor 3 subunit	166.97	YLEEK(1)GTTEEVCR	1.13
Q99613	EIF3C;EIF3CL	664	Eukaryotic translation initiation factor 3 subunit	93.551	NQEQEK(1)VER	1.32
O15371	EIF3D	505	Eukaryotic translation initiation factor 3 subunit	112.84	LEEGK(1)YLILK	0.96
P60228	EIF3E	35	Eukaryotic translation initiation factor 3 subunit	98.943	EIYNEK(1)ELLQGK	Tip60 OE only
O75821	EIF3G	161	Eukaryotic translation initiation factor 3 subunit	84.658	ICK(1)GDHWTTR	1.13
O15372	EIF3H	165	Eukaryotic translation initiation factor 3 subunit	89.827	TAQGSLSLK(1)AYR	0.72
O15372	EIF3H	172	Eukaryotic translation initiation factor 3 subunit	92.062	LTPK(1)LMEVCK	1.05
O75822	EIF3J	166	Eukaryotic translation initiation factor 3 subunit	102.51	DK(1)ITQYEK	1.08
Q9Y262	EIF3L	347	Eukaryotic translation initiation factor 3 subunit	101.69	TK(1)SMFQR	1
Q9Y262	EIF3L	397	Eukaryotic translation initiation factor 3 subunit	47.067	YGDK(1)MLR	1.05
Q9Y262	EIF3L	494	Eukaryotic translation initiation factor 3 subunit	63.486	IQLLVFK(1)HK	Tip60 OE only
Q9Y262	EIF3L	534	Eukaryotic translation initiation factor 3 subunit	80.69	DMIHIADTK(1)VAR	Tip60 OE only
Q7L2H7	EIF3M	324	Eukaryotic translation initiation factor 3 subunit	102.07	MVYCK(1)IDQTQR	1.31
P60842	EIF4A1	238	Eukaryotic initiation factor 4A-I	134.25	K(1)EELTLEGIR	1.01

P60842	EIF4A1	146	Eukaryotic initiation factor 4A-I	100.67	AEVQK(1)LQMEAPHIIVGTPGI	1.06
P60842	EIF4A1	309	Eukaryotic initiation factor 4A-I	229.75	DFTVSAMHGDMDQK(1)ER	1.1
P60842	EIF4A1	174	Eukaryotic initiation factor 4A-I	83.753	YLSPK(1)YIK	1.12
P60842	EIF4A1	193	Eukaryotic initiation factor 4A-I	57.492	GFK(1)DQIYDIFQK	Tip60 OE only
P60842	EIF4A1;EIF4A2	291	Eukaryotic initiation factor 4A-I;Eukaryotic initia	82.171	VDWLTEK(1)MHAR	1.19
P38919	EIF4A3	70	Eukaryotic initiation factor 4A-III;Eukaryotic initi	58.246	AIK(1)QIIK	0.97
P38919	EIF4A3	198	Eukaryotic initiation factor 4A-III;Eukaryotic initi	134.68	GFK(1)EQIYDVYR	1.05
P38919	EIF4A3	152	Eukaryotic initiation factor 4A-III;Eukaryotic initi	52.026	K(1)LDYGQHVVAGTPGR	1.08
P38919	EIF4A3	296	Eukaryotic initiation factor 4A-III;Eukaryotic initi	54.584	KVDWLTEK(1)MR	1.39
P60842	EIF4A3;EIF4A1;EIF4A	54	Eukaryotic initiation factor 4A-III;Eukaryotic initi	122.51	GIYAYGFEK(1)PSAIQQR	1.13
Q04637	EIF4G1	1513	Eukaryotic translation initiation factor 4 gamma	107.21	LLQK(1)YLCDEQK	0.95
Q04637	EIF4G1	1452	Eukaryotic translation initiation factor 4 gamma	57.859	LLK(1)EGSSNQR	1.61
Q04637	EIF4G1;EIF4G3	944	Eukaryotic translation initiation factor 4 gamma	107.06	LLTTIGK(1)DLDFEK	1.12
Q04637	EIF4G1;EIF4G3	950	Eukaryotic translation initiation factor 4 gamma	79.974	DLDFEK(0.989)AK(0.011)PR	unquantifiable
Q15056	EIF4H	80	Eukaryotic translation initiation factor 4H	78.516	DKDTDK(1)FK	0.29
P55010	EIF5	374	Eukaryotic translation initiation factor 5	101.72	VK(1)AEPFIK	0.94
P63241	EIF5A;EIF5A2	39	Eukaryotic translation initiation factor 5A-1;Euka	162.26	GRPCK(1)IVEMSTSK	0.95
P63241	EIF5A;EIF5A2;EIF5AL1	67	Eukaryotic translation initiation factor 5A-1;Euka	91.936	VHLVGIDIFTGK(1)K	0.89
P63241	EIF5A;EIF5A2;EIF5AL1	68	Eukaryotic translation initiation factor 5A-1;Euka	80.609	K(1)YEDICPSTHNMDVPNIK	0.94
P63241	EIF5A;EIF5A2;EIF5AL1	85	Eukaryotic translation initiation factor 5A-1;Euka	127.97	YEDICPSTHNMDVPNIK(1)R	1
P63241	EIF5A;EIF5AL1	121	Eukaryotic translation initiation factor 5A-1;Euka	218.62	LPEGDLGK(1)EIEQK	0.98
O60841	EIF5B	923	Eukaryotic translation initiation factor 5B	53.683	HK(1)EVEAAQGVK	0.91
Q15717	ELAVL1	120	ELAV-like protein 1	112.13	TMTQK(1)DVEDMFSR	Tip60 OE only
Q8N766	EMC1	986	ER membrane protein complex subunit 1	77.058	LAQVK(1)LLNR	1.06
P54851	EMP2	126	Epithelial membrane protein 2	47.082	EDIHDK(1)NAK	unquantifiable
P54851	EMP2	129	Epithelial membrane protein 2	41.876	NAK(1)FYPVTR	unquantifiable
Q8N8S7	ENAH	574	Protein enabled homolog	103.55	LK(1)EELIDAIR	0.71
P06733	ENO1	89	Alpha-enolase	252.21	LNVTEQEK(1)IDK	0.91
P06733	ENO1	80	Alpha-enolase	98.033	TIAPALVSK(1)K	0.95
P06733	ENO1	262	Alpha-enolase	134.13	YDLDFK(1)SPDDPSR	0.96
P06733	ENO1	103	Alpha-enolase	183.64	LMIEMDGTENK(1)SK	0.96
P06733	ENO1	233	Alpha-enolase	104.05	TAIGK(1)AGYTDK	0.96
P06733	ENO1	60	Alpha-enolase	58.716	YMGK(1)GVSK	0.98
P06733	ENO1	5	Alpha-enolase	154.01	SILK(1)IHAR	0.98

P06733	ENO1	64	Alpha-enolase	157.66	GVSK(1)AVEHINK	0.99
P06733	ENO1	71	Alpha-enolase	142.85	AVEHINK(1)TIAPALVSK	1
P06733	ENO1	335	Alpha-enolase	190.86	AVNEK(1)SCNCLLLK	1
P06733	ENO1	202	Alpha-enolase	188.9	YGK(1)DATNVGDEGGFAPNIL	1.02
P06733	ENO1	256	Alpha-enolase	75.378	SGK(1)YDLDFK	1.02
P06733	ENO1	92	Alpha-enolase	147.26	IDK(1)LMIEMDGTENK	1.04
P06733	ENO1	193	Alpha-enolase	136.96	IGAEVYHNLK(1)NVIK	1.05
P06733	ENO1	126	Alpha-enolase	132.84	AGAVEK(1)GVPLYR	1.05
P06733	ENO1	406	Alpha-enolase	121.52	LAK(1)YNQLLR	1.14
P06733	ENO1	326	Alpha-enolase	128.96	FTASAGIQVVGDDLTVTNPK(1	Tip60 OE only
P09104	ENO2	233	Gamma-enolase	88.496	EAIDK(1)AGYTEK	unquantifiable
P09104	ENO2;ENO3	60	Gamma-enolase;Beta-enolase	57.811	YLGK(1)GVLK	Tip60 OE only
O43491	EPB41L2	505	Band 4.1-like protein 2	64.711	LVSPEQPPK(1)AK	0.47
P29322	EPHA8	503	Ephrin type-A receptor 8	40.724	ATVSGLK(1)PGTR	unquantifiable
P07099	EPHX1	286	Epoxide hydrolase 1	85.533	DVELLYPVK(1)EK	unquantifiable
P07814	EPRS	139	Bifunctional glutamate/proline--tRNA ligase;Glut	76.465	GNAAWQEQLK(1)QK	0.96
P07814	EPRS	300	Bifunctional glutamate/proline--tRNA ligase;Glut	151.1	AYVDDTPAEQMK(1)AER	0.99
P07814	EPRS	1389	Bifunctional glutamate/proline--tRNA ligase;Glut	108.59	DTGEK(1)LTVAENEAETK	1.02
P07814	EPRS	512	Bifunctional glutamate/proline--tRNA ligase;Glut	54.525	YVALLK(1)K	1.06
P07814	EPRS	907	Bifunctional glutamate/proline--tRNA ligase;Glut	121.08	VLFDK(1)VASQGEVVR	1.1
P07814	EPRS	774	Bifunctional glutamate/proline--tRNA ligase;Glut	60.518	K(0.003)APK(0.997)EDVDAA	1.19
P07814	EPRS	197	Bifunctional glutamate/proline--tRNA ligase;Glut	43.68	FVELPGAEMGK(1)VTVR	Tip60 OE only
Q8IUD2	ERC1	660	ELKS/Rab6-interacting/CAST family member 1	107.06	EK(1)VSLLQGDLSEK	unquantifiable
P84090	ERH	12	Enhancer of rudimentary homolog	104.41	SHTILLVQPTK(1)RPEGR	0.95
P84090	ERH	41	Enhancer of rudimentary homolog	73.248	MYEEHLK(1)R	0.98
P84090	ERH	84	Enhancer of rudimentary homolog	60.49	ADTQTYQPYNK(1)DWIK	0.98
P84090	ERH	90	Enhancer of rudimentary homolog	87.284	EK(1)IYVLLR	Tip60 OE only
O94905	ERLIN2	241	Erlin-2	120.06	K(1)ISEIEDAAFLAR	1.12
O94905	ERLIN2	285	Erlin-2	92.062	LTPEYLQLMK(1)YK	Tip60 OE only
O94905	ERLIN2	257	Erlin-2	73.499	AK(1)ADAECYTAMK	unquantifiable
O94905	ERLIN2;ERLIN1	199	Erlin-2;Erlin-1	104.76	LLIAAQK(1)QK	0.99
O94905	ERLIN2;ERLIN1	205	Erlin-2;Erlin-1	94.692	VVEK(1)EAETER	1
O94905	ERLIN2;ERLIN1	236	Erlin-2;Erlin-1	43.246	VMEK(1)ETEK	Tip60 OE only
Q96HE7	ERO1L	396	ERO1-like protein alpha	90.71	IMDCVGCFK(1)CR	1

P30040	ERP29	69	Endoplasmic reticulum resident protein 29	77.305	FDTQYPYGEK(1)QDEFK	0.77
P30040	ERP29	54	Endoplasmic reticulum resident protein 29	79.116	SK(1)FVLVK	0.97
Q9BS26	ERP44	343	Endoplasmic reticulum resident protein 44	43.246	DVLIPGK(1)LK	0.94
Q5FWF5	ESCO1	456	N-acetyltransferase ESCO1	61.962	MK(1)EINSEEVK	unquantifiable
P10768	ESD	4	S-formylglutathione hydrolase	81.296	ALK(1)QISSNK	0.89
P10768	ESD	247	S-formylglutathione hydrolase	69.432	K(1)IPVVFR	0.99
P10768	ESD	198	S-formylglutathione hydrolase	147.72	AFSGYLGTDQSK(1)WK	1
P10768	ESD	200	S-formylglutathione hydrolase	57.885	WK(1)AYDATHLVK	unquantifiable
Q9BSJ8	ESYT1	355	Extended synaptotagmin-1	72.096	GLIEGK(1)SDPYALVR	Tip60 OE only
P62495	ETF1	16	Eukaryotic peptide chain release factor subunit 1	61.999	NVEIWK(1)IK	1.12
P13804	ETFA	69	Electron transfer flavoprotein subunit alpha, mit	123.51	VAQDLCK(1)VAGIAK	0.83
P13804	ETFA	206	Electron transfer flavoprotein subunit alpha, mit	107.37	LTK(1)SDRPELTGAK	0.86
P13804	ETFA	75	Electron transfer flavoprotein subunit alpha, mit	105.25	VAGIAK(1)VLVAQHDVYK	0.95
P38117	ETFB	176	Electron transfer flavoprotein subunit beta	76.943	LK(1)LPAVVTADLR	0.98
P38117	ETFB	110	Electron transfer flavoprotein subunit beta	49.418	VLAK(1)LAEK	1.04
P38117	ETFB	11	Electron transfer flavoprotein subunit beta	120.76	VLVAVK(1)R	1.07
P38117	ETFB	116	Electron transfer flavoprotein subunit beta	76.1	EK(1)VDLVLLGK	1.07
P38117	ETFB	248	Electron transfer flavoprotein subunit beta	85.377	VETTEDLVAK(1)LK	Tip60 OE only
Q01844	EWSR1	441	RNA-binding protein EWS	77.984	LK(1)VSLAR	1.04
P15311	EZR	458	Ezrin	57.708	EAQDDLVK(1)TK	0.78
P15311	EZR	450	Ezrin	90.906	AK(1)EAQDDLVK	0.82
P15311	EZR	523	Ezrin	119.27	ITEAEK(1)NER	0.89
P15311	EZR	337	Ezrin	74.415	EK(1)EQMMR	1.01
P15311	EZR	344	Ezrin	113.5	EK(1)EELMLR	1.03
P15311	EZR	143	Ezrin	89.189	EVHK(1)SGYLSSER	1.05
P15311	EZR	438	Ezrin	51.252	K(1)EDEVEEWQHR	Tip60 OE only
P26038	EZR;MSN;RDX	262	Ezrin;Moesin;Radixin	65.305	FVIKPIDK(1)K	0.88
P26038	EZR;MSN;RDX	263	Ezrin;Moesin;Radixin	67.153	K(1)APDFVFYAPR	0.99
P26038	EZR;MSN;RDX	254	Ezrin;Moesin;Radixin	66.692	K(1)FVIKPIDK	Tip60 OE only
P35241	EZR;RDX	316	Ezrin;Radixin	112.17	HQK(1)QLER	0.99
P35241	EZR;RDX	79	Ezrin;Radixin	110.87	ENPLQFK(1)FR	1.12
Q01469	FABP5	17	Fatty acid-binding protein, epidermal	136.33	LVDSK(1)GFDEYMK	0.58
Q13158	FADD	125	FAS-associated death domain protein	69.721	VSDTK(1)IDSIEDR	unquantifiable
Q96C01	FAM136A	130	Protein FAM136A	68.893	MK(1)EALLSIGK	unquantifiable

Q8WVX9	FAR1	29	Fatty acyl-CoA reductase 1	67.032	VLLEK(1)LLR	unquantifiable
Q9NSD9	FARSB	47	Phenylalanine--tRNA ligase beta subunit	71.451	EIISK(1)EQGNVK	0.94
P49327	FASN	1911	Fatty acid synthase;[Acyl-carrier-protein] S-acetyl	101.65	GVQK(1)LVLTSR	0.52
P49327	FASN	1827	Fatty acid synthase;[Acyl-carrier-protein] S-acetyl	58.904	DGVVRPLK(1)CTVFHGAQVED	0.69
P49327	FASN	673	Fatty acid synthase;[Acyl-carrier-protein] S-acetyl	87.696	EGVFAK(1)EVR	0.7
P49327	FASN	1393	Fatty acid synthase;[Acyl-carrier-protein] S-acetyl	113.93	K(1)SFYGSTLFLCR	0.97
P49327	FASN	1847	Fatty acid synthase;[Acyl-carrier-protein] S-acetyl	89.298	YMAQGK(1)HIGK	1
P49327	FASN	1072	Fatty acid synthase;[Acyl-carrier-protein] S-acetyl	198.49	LYTLQDK(1)AQVADVVVSR	1.09
P49327	FASN	2426	Fatty acid synthase;[Acyl-carrier-protein] S-acetyl	80.377	SFYYK(1)LR	1.1
P49327	FASN	1239	Fatty acid synthase;[Acyl-carrier-protein] S-acetyl	155.99	ACLDTAVENMPSLK(1)MK	1.13
P49327	FASN	1927	Fatty acid synthase;[Acyl-carrier-protein] S-acetyl	98.523	TGYQAK(1)QVR	1.16
P49327	FASN	41	Fatty acid synthase;[Acyl-carrier-protein] S-acetyl	87.696	WK(1)AGLYGLPR	1.2
P49327	FASN	2193	Fatty acid synthase;[Acyl-carrier-protein] S-acetyl	138.77	LQELSSK(1)ADEASELACPTPK	Tip60 OE only
P49327	FASN	2186	Fatty acid synthase;[Acyl-carrier-protein] S-acetyl	62.338	K(1)LQELSSK	unquantifiable
P22087	FBL	102	rRNA 2'-O-methyltransferase fibrillarin	41.427	GK(1)EDALVTK	0.96
P22087	FBL	205	rRNA 2'-O-methyltransferase fibrillarin	60.788	DLINLAK(1)K	1.05
P22087	FBL	121	rRNA 2'-O-methyltransferase fibrillarin	83.397	NLVPGESVYGEK(1)R	Tip60 OE only
P14324	FDPS	123	Farnesyl pyrophosphate synthase	99.653	EVLEYNAIGGK(1)YNR	Tip60 OE only
P39748	FEN1	200	Flap endonuclease 1	89.698	HLTASEAK(1)K	0.95
P39748	FEN1	24	Flap endonuclease 1	73.985	ENDIK(1)SYFGR	1
P39748	FEN1	314	Flap endonuclease 1	170.47	FMCGEK(1)QFSEER	1.11
P39748	FEN1	354	Flap endonuclease 1	45.368	VTGSLSSAK(1)R	1.14
P39748	FEN1	252	Flap endonuclease 1	87.323	AVDLIQK(1)HK	1.16
P07954	FH	221	Fumarate hydratase, mitochondrial	139.02	LHDALDAK(1)SK	0.8
Q13642	FHL1	91	Four and a half LIM domains protein 1	155.75	ILCNK(1)CTTR	1.02
Q13642	FHL1	4	Four and a half LIM domains protein 1	88.819	AEK(1)FDCHYCR	1.41
Q96AY3	FKBP10	570	Peptidyl-prolyl cis-trans isomerase FKBP10	68.016	LK(1)SDEDEER	0.74
P62942	FKBP1A	53	Peptidyl-prolyl cis-trans isomerase FKBP1A	122.52	FMLGK(1)QEVIR	unquantifiable
Q00688	FKBP3	86	Peptidyl-prolyl cis-trans isomerase FKBP3	90.657	VSEQVK(1)NVK	0.84
Q00688	FKBP3	170	Peptidyl-prolyl cis-trans isomerase FKBP3	74.415	VGVGK(1)VIR	0.85
Q00688	FKBP3	22	Peptidyl-prolyl cis-trans isomerase FKBP3	67.035	SEQLPK(1)K	0.89
Q00688	FKBP3	99	Peptidyl-prolyl cis-trans isomerase FKBP3	113.63	ETK(1)SEETLDEGPPK	0.97
Q00688	FKBP3	73	Peptidyl-prolyl cis-trans isomerase FKBP3	66.27	RFK(1)GTESISK	1.01
Q02790	FKBP4	250	Peptidyl-prolyl cis-trans isomerase FKBP4;Peptid	89.827	YELHLK(1)SFEK	0.84

Q02790	FKBP4	426	Peptidyl-prolyl cis-trans isomerase FKBP4;Peptid	54.281	AK(1)AEASSGDHPTDTEMK	0.9
Q02790	FKBP4	287	Peptidyl-prolyl cis-trans isomerase FKBP4;Peptid	135.84	YK(1)QALLQYK	0.95
Q02790	FKBP4	83	Peptidyl-prolyl cis-trans isomerase FKBP4;Peptid	63.408	FSFDLGK(1)GEVIK	0.97
Q02790	FKBP4	274	Peptidyl-prolyl cis-trans isomerase FKBP4;Peptid	142.92	LEQSTIVK(1)ER	1
Q02790	FKBP4	390	Peptidyl-prolyl cis-trans isomerase FKBP4;Peptid	138.98	AAK(1)TQLAVCQQR	1.01
Q02790	FKBP4	282	Peptidyl-prolyl cis-trans isomerase FKBP4;Peptid	56.729	GTVYFK(1)EGK	1.01
Q02790	FKBP4	256	Peptidyl-prolyl cis-trans isomerase FKBP4;Peptid	80.763	AK(1)ESWEMNSEEK	1.04
Q02790	FKBP4	181	Peptidyl-prolyl cis-trans isomerase FKBP4;Peptid	70.67	DK(1)LFDQR	1.04
Q02790	FKBP4	76	Peptidyl-prolyl cis-trans isomerase FKBP4;Peptid	66.692	DK(1)FSFDLGK	1.16
Q02790	FKBP4	387	Peptidyl-prolyl cis-trans isomerase FKBP4;Peptid	49.06	VLQLYPNNK(1)AAK	1.64
Q02790	FKBP4	354	Peptidyl-prolyl cis-trans isomerase FKBP4;Peptid	93.258	ALELDSNNEK(1)GLFR	Tip60 OE only
Q13451	FKBP5	272	Peptidyl-prolyl cis-trans isomerase FKBP5;Peptid	66.267	EKLEQAAIVK(1)EK	0.86
Q13451	FKBP5	385	Peptidyl-prolyl cis-trans isomerase FKBP5;Peptid	132.9	VLEVNPQNK(1)AAR	1.01
Q13451	FKBP5	264	Peptidyl-prolyl cis-trans isomerase FKBP5;Peptid	106.42	EK(1)LEQAAIVK	1.05
Q13451	FKBP5	342	Peptidyl-prolyl cis-trans isomerase FKBP5;Peptid	95.401	AVECCDK(1)ALGLDSANEK	1.07
Q13451	FKBP5	208	Peptidyl-prolyl cis-trans isomerase FKBP5;Peptid	77.72	ALEK(1)MQR	Tip60 OE only
P21333	FLNA	1071	Filamin-A	128.9	VK(1)AFGPGLQGGSAGSPAR	0.87
P21333	FLNA	1164	Filamin-A	44.203	VK(1)CSGPGLER	0.88
P21333	FLNA	1824	Filamin-A	82.34	VAQPTITDNK(1)DGTVTVR	1.01
P21333	FLNA	2563	Filamin-A	41.54	VVAK(1)GLGLSK	1.02
P21333	FLNA	508	Filamin-A	96.015	VYTK(1)GAGSGELK	1.04
P21333	FLNA	987	Filamin-A	99.53	VDVGK(1)DQEFTVK	1.21
P21333	FLNA	2387	Filamin-A	53.444	VHSPSGALEECYVTEIDQDK(1)	Tip60 OE only
P21333	FLNA	1452	Filamin-A	75.416	VK(1)CSGPGLSPGMVR	Tip60 OE only
P21333	FLNA	2621	Filamin-A	73.067	LYSVSYLLK(1)DK	Tip60 OE only
P21333	FLNA;FLNC	626	Filamin-A;Filamin-C	108.37	IECDDK(1)GDGSCDVR	1.02
P21333	FLNB;FLNA	127	Filamin-B;Filamin-A	99.215	LVSIDSK(1)AIVDGNLK	0.69
P21333	FLNB;FLNA	2141	Filamin-B;Filamin-A	69.998	VK(1)ESITR	0.93
P21333	FLNB;FLNA	120	Filamin-B;Filamin-A	63.184	ESIK(1)LVSIDSK	0.99
P21333	FLNB;FLNA;FLNC	493	Filamin-B;Filamin-A;Filamin-C	88.591	GLQPK(1)GVR	1.28
Q14315	FLNC	120	Filamin-C	99.215	LVSIDSK(1)AIVDGNLK	1.21
O75955	FLOT1	174	Flotillin-1	112.84	TAQVQK(1)DAR	unquantifiable
Q14254	FLOT2	307	Flotillin-2	126.67	VK(1)QVLLAQAEAEK	0.48
Q14254	FLOT2	305	Flotillin-2	94.692	IQQIAEGEK(1)VK	0.73

Q14254	FLOT2	211	Flotillin-2	72.006	FMADTK(1)IADSK	0.83
Q16658	FSCN1	241	Fascin	64.64	YLAPSGPSGTLK(1)AGK	0.84
Q16658	FSCN1	74	Fascin	220.94	YLAADK(1)DGNVTCER	1.01
Q16658	FSCN1	220	Fascin	89.673	SGK(1)VAFR	Tip60 OE only
Q16658	FSCN1	399	Fascin	93.649	K(1)VTGTLDANR	unquantifiable
Q8IY81	FTSJ3	778	pre-rRNA processing protein FTSJ3	75.269	EK(1)VAQLR	1.38
Q96AE4	FUBP1	400	Far upstream element-binding protein 1	109.6	GGETIK(1)SISQQSGAR	1.48
Q96I24	FUBP3	186	Far upstream element-binding protein 3	105.4	GGETIK(1)QLQER	0.86
Q96I24	FUBP3	220	Far upstream element-binding protein 3	98.249	ITGDAFK(1)VQQAR	1.05
P35637	FUS	365	RNA-binding protein FUS	60.49	EFSGNPIK(1)VSFATR	1.15
P35637	FUS	334	RNA-binding protein FUS	62.924	LK(1)GEATVSFDDPPSAK	Tip60 OE only
P51114	FXR1;FXR2	310	Fragile X mental retardation syndrome-related pr	115.18	VIQEIVDK(1)SGVVR	Tip60 OE only
Q13283	G3BP1;G3BP2	413	Ras GTPase-activating protein-binding protein 1;	81.297	LNVEEK(1)K	0.83
Q14697	GANAB	48	Neutral alpha-glucosidase AB	111.95	TCEESSFCK(1)R	0.92
P04406	GAPDH	254	Glyceraldehyde-3-phosphate dehydrogenase	113.93	PAK(1)YDDIK	0.83
P04406	GAPDH	139	Glyceraldehyde-3-phosphate dehydrogenase	102.23	VIISAPSADAPMFVMGVNHEK	0.9
P04406	GAPDH	263	Glyceraldehyde-3-phosphate dehydrogenase	131.44	VVK(1)QASEGPLK	0.92
P04406	GAPDH	215	Glyceraldehyde-3-phosphate dehydrogenase	111.22	GALQNIIPASTGAAK(1)AVGK	0.96
P04406	GAPDH	194	Glyceraldehyde-3-phosphate dehydrogenase	129.1	TVDGPSGK(1)LWR	0.99
P04406	GAPDH	117	Glyceraldehyde-3-phosphate dehydrogenase	160.36	AGAHLQGGAK(1)R	1
P04406	GAPDH	334	Glyceraldehyde-3-phosphate dehydrogenase	141.91	VVDLMAHMASK(1)E	1.18
Q14C86	GAPVD1	36	GTPase-activating protein and VPS9 domain-con	45.997	LNADVLK(1)TAEK	unquantifiable
Q9NY12	GAR1	134	H/ACA ribonucleoprotein complex subunit 1	91.658	LSENMK(1)ASSFK	1.14
P41250	GARS	108	Glycine--tRNA ligase	111.65	VLEAK(1)ELALQPK	0.98
P41250	GARS	733	Glycine--tRNA ligase	99.653	YPLFEGQETGK(1)K	Tip60 OE only
P41250	GARS	501	Glycine--tRNA ligase	113.77	TVNVVQFEPSK(1)GAIGK	Tip60 OE only
P22102	GART	156	Trifunctional purine biosynthetic protein adenos	102.06	ASGLAAGK(1)GVIVAK	0.98
P22102	GART	251	Trifunctional purine biosynthetic protein adenos	111.82	IK(1)DTVLQR	1.05
Q9H0R5	GBP3	388	Guanylate-binding protein 3	49.358	LAAQLDK(1)K	unquantifiable
Q92616	GCN1L1	2366	Translational activator GCN1	52.867	LK(1)AADALGK	0.83
Q92616	GCN1L1	24	Translational activator GCN1	101.64	VTTASVK(1)ER	1.19
Q92616	GCN1L1	308	Translational activator GCN1	51.875	GLAGHLK(1)SNSPR	Tip60 OE only
P31150	GDI1	269	Rab GDP dissociation inhibitor alpha	84.213	VVGVK(1)SEGEVAR	Tip60 OE only
P50395	GDI1;GDI2	210	Rab GDP dissociation inhibitor alpha;Rab GDP di	98.033	IK(1)LYSESLAR	Tip60 OE only

P50395	GDI2	164	Rab GDP dissociation inhibitor beta	76.827	TFEGIDPK(1)K	0.95
P50395	GDI2	269	Rab GDP dissociation inhibitor beta	93.111	VIGVK(1)SEGEIAR	1.08
P50395	GDI2	112	Rab GDP dissociation inhibitor beta	60.518	VTEGSFVYK(1)GGK	Tip60 OE only
Q7L5D6	GET4	58	Golgi to ER traffic protein 4 homolog	52.693	YMSQSK(1)HTEAR	unquantifiable
Q06210	GFPT1	556	Glutamine--fructose-6-phosphate aminotransfera	60.918	LATELYHQK(1)SVLIMGR	1.29
Q04760	GLO1	151	Lactoylglutathione lyase	51.066	FVK(0.99)K(0.01)PDDGK	0.64
Q04760	GLO1	44	Lactoylglutathione lyase	99.802	K(1)SLDFYTR	0.92
Q04760	GLO1	88	Lactoylglutathione lyase	113.38	DEK(1)IAWALSR	1.21
Q04760	GLO1	148	Lactoylglutathione lyase	52.482	FEELGVK(1)FVK	Tip60 OE only
Q9HC38	GLOD4	185	Glyoxalase domain-containing protein 4	104.17	IYEKDEEK(1)QR	Tip60 OE only
O76003	GLRX3	240	Glutaredoxin-3	42.869	LK(1)VLTNK	0.98
P00367	GLUD1	503	Glutamate dehydrogenase 1, mitochondrial	81.357	ISGASEK(1)DIVHSGLAYTMER	1.37
P36959	GMPR;GMPR2	323	GMP reductase 1;GMP reductase 2	53.683	STCTYVGAAK(1)LK	unquantifiable
P49915	GMPS	487	GMP synthase [glutamine-hydrolyzing]	106.62	VK(1)ACTTEEDQEK	0.9
P49915	GMPS	289	GMP synthase [glutamine-hydrolyzing]	54.571	K(1)LGIQVK	unquantifiable
P29992	GNA11	72	Guanine nucleotide-binding protein subunit alpl	46.159	IIHGAGYSEEDK(1)R	2.78
P63092	GNAS	186	Guanine nucleotide-binding protein G(s) subunit	105.03	IDVIK(1)QADYVPSDQDLLR	unquantifiable
P63092	GNAS;GNAL;GNAT1;(	53	Guanine nucleotide-binding protein G(s) subunit	114.82	LLLLGAGESGK(1)STIVK	unquantifiable
P63244	GNB2L1	175	Guanine nucleotide-binding protein subunit beta	130.27	LVK(1)VWNLANCK	1.03
P63244	GNB2L1	38	Guanine nucleotide-binding protein subunit beta	72.485	DK(1)TIIMWK	1.04
P63244	GNB2L1	271	Guanine nucleotide-binding protein subunit beta	180.18	IIVDELK(1)QEVISTSSK	1.11
P63218	GNG5	27	Guanine nucleotide-binding protein G(I)/G(S)/G(	60.518	VK(1)VSQAAADLK	unquantifiable
P46926	GNPDA1	273	Glucosamine-6-phosphate isomerase 1	75.294	LVDPLYSIK(1)EK	1.09
Q13439	GOLGA4	498	Golgin subfamily A member 4	47.849	K(0.001)EQELTK(0.999)K	unquantifiable
Q14789	GOLGB1	2109	Golgin subfamily B member 1	48.283	ELQSNK(1)ESVK	Tip60 OE only
P00505	GOT2	234	Aspartate aminotransferase, mitochondrial	48.787	EIATVVK(1)K	0.86
P00505	GOT2	296	Aspartate aminotransferase, mitochondrial	99.752	VGAFTMVCK(1)DADEAK	0.89
P00505	GOT2	363	Aspartate aminotransferase, mitochondrial	64.654	TQLVSNLK(1)K	0.92
P00505	GOT2	90	Aspartate aminotransferase, mitochondrial	170	KAEAQIAAK(1)NLDK	Tip60 OE only
P00505	GOT2	227	Aspartate aminotransferase, mitochondrial	134.2	PEQWK(1)EIATVVK	Tip60 OE only
P06744	GPI	241	Glucose-6-phosphate isomerase	61.648	DPSAVAK(1)HFVALSTNTTK	0.78
P06744	GPI	142	Glucose-6-phosphate isomerase	45.368	SGDWK(1)GYTGK	0.83
P06744	GPI	116	Glucose-6-phosphate isomerase	121.45	SNTPILVDGK(1)DVMPEVNK	0.9
P06744	GPI	252	Glucose-6-phosphate isomerase	117.67	HFVALSTNTTK(1)VK	0.94

P06744	GPI	73	Glucose-6-phosphate isomerase	63.216	MLVDLAK(1)SR	0.96
P06744	GPI	454	Glucose-6-phosphate isomerase	226.73	ELQAAGK(1)SPEDLER	0.96
P06744	GPI	130	Glucose-6-phosphate isomerase	139.92	MK(1)SFCQR	0.97
P06744	GPI	366	Glucose-6-phosphate isomerase	85.064	YITK(1)SGTR	0.98
P06744	GPI	440	Glucose-6-phosphate isomerase	65.252	GK(1)STEEAR	1.01
P06744	GPI	234	Glucose-6-phosphate isomerase	99.215	EWFLQAAK(1)DPSAVAK	Tip60 OE only
Q13098	GPS1	422	COP9 signalosome complex subunit 1	103.34	VDSHSK(1)ILYAR	unquantifiable
Q13098	GPS1	337	COP9 signalosome complex subunit 1	83.31	FYESK(1)YASCLK	unquantifiable
P62993	GRB2	20	Growth factor receptor-bound protein 2	169.09	ATADDELSFK(1)R	1.09
Q9HAV7	GRPEL1	169	GrpE protein homolog 1, mitochondrial	43.611	LNPVGAK(1)FDPYEHEALFHTF	1.11
P15170	GSPT1	151	Eukaryotic peptide chain release factor GTP-bind	69.998	AYFETEK(1)K	1.06
P15170	GSPT1;GSPT2	208	Eukaryotic peptide chain release factor GTP-bind	115.78	EHAMLAK(1)TAGVK	0.86
P15170	GSPT1;GSPT2	311	Eukaryotic peptide chain release factor GTP-bind	53.683	YK(1)DMGTVVLGK	0.9
P15170	GSPT1;GSPT2	138	Eukaryotic peptide chain release factor GTP-bind	69.984	GK(1)TVEVGR	0.96
P15170	GSPT1;GSPT2	213	Eukaryotic peptide chain release factor GTP-bind	89.548	TAGVK(1)HLIVLINK	1.11
P15170	GSPT1;GSPT2	448	Eukaryotic peptide chain release factor GTP-bind	65.347	FVK(1)QDQVCIAR	1.31
P00390	GSR	296	Glutathione reductase, mitochondrial	92.47	FSQVK(1)EVK	Tip60 OE only
P48637	GSS	172	Glutathione synthetase	52.725	HVLSVLSK(1)TK	Tip60 OE only
P78417	GSTO1	160	Glutathione S-transferase omega-1	167.24	LEEVLTNK(1)K	0.81
P78417	GSTO1	198	Glutathione S-transferase omega-1	42.718	LNECVDHTPK(1)LK	0.98
P78347	GTF2I	477	General transcription factor II-I	69.721	K(1)FAEALGSTEAK	0.49
P78347	GTF2I	744	General transcription factor II-I	83.314	VENLFNEK(1)CGEALGLK	0.67
Q9UKN8	GTF3C4	123	General transcription factor 3C polypeptide 4	46.462	VGSK(1)TEVAECK	Tip60 OE only
Q9BZE4	GTPBP4	332	Nucleolar GTP-binding protein 1	93.429	VK(1)TEACDR	0.77
P16104	H2AFX	128	Histone H2AX	59.542	TSATVGPK(1)APSGGK	Tip60 OE only
O75367	H2AFY	295	Core histone macro-H2A.1	47.532	TVK(1)NCLALADDKK	0.96
O75367	H2AFY	304	Core histone macro-H2A.1	76.465	NCLALADDK(1)K	1.23
O75367	H2AFY	116	Core histone macro-H2A.1	52.185	GVTIASGGVLPNIHPELLAK(1)I	Tip60 OE only
O75367	H2AFY	235	Core histone macro-H2A.1	82.426	DDLGNTLEK(1)K	Tip60 OE only
O75367	H2AFY	307	Core histone macro-H2A.1	82.417	LK(1)SIAFPSIGSGR	unquantifiable
Q9P035	HACD3	85	Very-long-chain (3R)-3-hydroxyacyl-CoA dehydra	88.358	K(1)VSQWWER	0.95
Q16836	HADH	127	Hydroxyacyl-coenzyme A dehydrogenase, mitoch	78.324	VK(1)NELFK	0.89
Q16836	HADH	241	Hydroxyacyl-coenzyme A dehydrogenase, mitoch	100.72	GDASK(1)EDIDTAMK	0.93
Q16836	HADH	212	Hydroxyacyl-coenzyme A dehydrogenase, mitoch	70.889	HPVSCK(1)DTPGFIVNR	0.96

P40939	HADHA	60	Trifunctional enzyme subunit alpha, mitochondr	46.706	INSPNSK(1)VNTLSK	0.79
P40939	HADHA	531	Trifunctional enzyme subunit alpha, mitochondr	56.359	DTSASAVAVGLK(1)QGK	0.89
P40939	HADHA	634	Trifunctional enzyme subunit alpha, mitochondr	55.452	SGK(1)GFYIYQEGVK	1.02
P40939	HADHA	334	Trifunctional enzyme subunit alpha, mitochondr	101.38	FGELVMTK(1)ESK	1.14
P40939	HADHA	390	Trifunctional enzyme subunit alpha, mitochondr	110.84	TILK(1)DATLTALDR	Tip60 OE only
P40939	HADHA	644	Trifunctional enzyme subunit alpha, mitochondr	118.5	GFYIYQEGVK(1)R	Tip60 OE only
P55084	HADHB	41	Trifunctional enzyme subunit beta, mitochondri	53.327	AAPAVQTK(1)TK	1.13
P55084	HADHB	201	Trifunctional enzyme subunit beta, mitochondri	77.923	LSLISK(1)FR	Tip60 OE only
P55084	HADHB	188	Trifunctional enzyme subunit beta, mitochondri	73.26	LMLDLNK(1)AK	Tip60 OE only
P12081	HARS	106	Histidine--tRNA ligase, cytoplasmic	73.781	ETLMGK(1)YGEDSK	0.87
P12081	HARS	75	Histidine--tRNA ligase, cytoplasmic	44.349	EK(1)VFDVIIR	0.89
P12081	HARS	42	Histidine--tRNA ligase, cytoplasmic	102.06	LK(1)AQLGPDESK	1.25
P12081	HARS	403	Histidine--tRNA ligase, cytoplasmic	90.657	LEALEEK(1)IR	Tip60 OE only
P12081	HARS	257	Histidine--tRNA ligase, cytoplasmic	93.958	NEMVGEK(1)GLAPEVADR	Tip60 OE only
O14929	HAT1	15	Histone acetyltransferase type B catalytic subuni	43.512	FLVEYK(1)SAVEK	Tip60 OE only
Q7Z4V5	HDGFRP2	554	Hepatoma-derived growth factor-related protein	95.502	VLGPK(1)IEAVQK	0.96
Q00341	HDLBP	1031	Vigilin	67.981	AK(1)AGLLER	1.04
Q00341	HDLBP	526	Vigilin	48.283	TIIGQK(1)GER	Tip60 OE only
Q00341	HDLBP	494	Vigilin	94.692	IEGDPQGVQQAK(1)R	Tip60 OE only
P07686	HEXB	161	Beta-hexosaminidase subunit beta;Beta-hexosam	86.833	EPVAVLK(1)ANR	1.95
P49773	HINT1	30	Histidine triad nucleotide-binding protein 1	83.397	EIPAK(1)IIFEDDR	0.75
P49773	HINT1	7	Histidine triad nucleotide-binding protein 1	121.96	ADEIAK(1)AQVAR	0.86
P49773	HINT1	21	Histidine triad nucleotide-binding protein 1	163.16	PGGDTIFGK(1)IIR	0.91
P16403	HIST1H1C;HIST1H1E;	46	Histone H1.2;Histone H1.4;Histone H1.3	79.568	KASGPPVSELITK(1)AVAASK	0.81
P16403	HIST1H1C;HIST1H1E;	63	Histone H1.2;Histone H1.4;Histone H1.3	66.692	SGVSLAALK(1)K	0.88
P16403	HIST1H1C;HIST1H1E;	90	Histone H1.2;Histone H1.4;Histone H1.3;Histone	157.66	SLVSK(1)GTLVQTK	0.95
P16403	HIST1H1C;HIST1H1E;	97	Histone H1.2;Histone H1.4;Histone H1.3;Histone	186.17	GTLVQTK(1)GTGASGSFK	1
P16403	HIST1H1C;HIST1H1E;	106	Histone H1.2;Histone H1.4;Histone H1.3;Histone	48.028	GTGASGSFK(1)LNK	Tip60 OE only
P16403	HIST1H1C;HIST1H1E;	75	Histone H1.2;Histone H1.4;Histone H1.3;Histone	134.13	ALAAAGYDVEK(1)NNSR	1.12
P16403	HIST1H1C;HIST1H1E;	52	Histone H1.2;Histone H1.4;Histone H1.3;Histone	91.626	AVAASK(1)ER	1.1
Q99878	HIST1H2AJ;HIST1H2A	37	Histone H2A type 1-J;Histone H2A type 1-H;Histo	86.472	K(1)GNYAER	0.92
Q99878	HIST1H2AJ;HIST1H2A	96	Histone H2A type 1-J;Histone H2A type 1-H;Histo	166.09	NDEELNK(1)LLGK	1.09
P62807	HIST1H2BC;HIST1H2	6	Histone H2B type 1-C/E/F/G/I;Histone H2B type 1	85.958	PEPAK(1)SAPAPK	1.08
P58876	HIST1H2BD	6	Histone H2B type 1-D	78.934	PEPTK(1)SAPAPK	0.71

P62807	HIST1H2BD;HIST1H2	109	Histone H2B type 1-D;Histone H2B type 1-C/E/F/(	166.62	LLLPGELAK(1)HAVSEGTK	1.02
P62807	HIST1H2BD;HIST1H2	117	Histone H2B type 1-D;Histone H2B type 1-C/E/F/(	150.46	HAVSEGTK(1)AVTK	1.17
P62807	HIST1H2BD;HIST1H2	121	Histone H2B type 1-D;Histone H2B type 1-C/E/F/(	69.598	AVTK(1)YTSSK	1.47
P62807	HIST1H2BD;HIST1H2	44	Histone H2B type 1-D;Histone H2B type 1-C/E/F/(	53.327	ESYSVYVYK(1)VLK	0.97
P62807	HIST1H2BD;HIST1H2	35	Histone H2B type 1-D;Histone H2B type 1-C/E/F/(	135.71	K(1)ESYSVYVYK	1.03
P62807	HIST1H2BD;HIST1H2	47	Histone H2B type 1-D;Histone H2B type 1-C/E/F/(	100.93	VLK(1)QVHPDTGISSK	0.96
P62807	HIST1H2BD;HIST1H2	86	Histone H2B type 1-D;Histone H2B type 1-C/E/F/(	130.84	LAHYNK(1)R	1.06
P62805	HIST1H4A	78	Histone H4	163.32	DAVTYTEHAK(1)R	0.98
P62805	HIST1H4A	92	Histone H4	190.05	TVTAMDVVYALK(1)R	1.02
P62805	HIST1H4A	80	Histone H4	147.91	RK(1)TVTAMDVVYALK	1.02
Q16778	HIST2H2BE;HIST1H2I	47	Histone H2B type 2-E;Histone H2B type 1-B;Histo	100.93	VLK(1)QVHPDTGISSK	0.92
Q5QNW6	HIST2H2BF	21	Histone H2B type 2-F	66.994	AVTK(1)VQK	1.04
Q71DI3	HIST2H3A;H3F3A;HIS	24	Histone H3.2;Histone H3.3;Histone H3.1;Histone	72.23	KQLATK(1)AAR	0.98
Q71DI3	HIST2H3A;H3F3A;HIS	80	Histone H3.2;Histone H3.3;Histone H3.1;Histone	143.7	EIAQDFK(1)TDLR	1.15
Q71DI3	HIST2H3A;H3F3A;HIS	123	Histone H3.2;Histone H3.3;Histone H3.1;Histone	132.01	VTIMPK(1)DIQLAR	0.96
Q71DI3	HIST2H3A;H3F3A;HIS	15	Histone H3.2;Histone H3.3;Histone H3.1;Histone	55.441	STGGK(1)APR	1.74
Q71DI3	HIST2H3A;H3F3A;HIS	57	Histone H3.2;Histone H3.3;Histone H3.1;Histone	74.162	RYQK(1)STELLIR	Tip60 OE only
P19367	HK1	763	Hexokinase-1	56.729	NILIDFTK(1)K	0.76
P19367	HK1	777	Hexokinase-1	105.52	GQISETLK(1)TR	0.9
P19367	HK1	738	Hexokinase-1	148.96	LVDEYSLNAGK(1)QR	0.93
P19367	HK1	176	Hexokinase-1	84.198	FK(1)ASGVEGADVVK	1.01
P19367	HK1;HK2	312	Hexokinase-1;Hexokinase-2	57.559	LILVK(1)MAK	0.88
P19367	HK1;HK2	315	Hexokinase-1;Hexokinase-2	83.862	MAK(1)EGLLFEGR	3.19
P19367	HK1;HKDC1	544	Hexokinase-1;Putative hexokinase HKDC1	109.88	VLLVK(1)IR	0.99
Q8TCT9	HM13	370	Minor histocompatibility antigen H13	69.672	EGTEASASK(1)GLEK	1.12
Q8TCT9	HM13	361	Minor histocompatibility antigen H13	82.069	DPAAVTESK(1)EGTEASASK	Tip60 OE only
P08397	HMBS	70	Porphobilinogen deaminase	57.348	ILDTALSK(1)IGEK	Tip60 OE only
P09429	HMGB1;HMGB1P1	114	High mobility group protein B1;Putative high mc	52.569	IK(1)GEHPGLSIGDVAK	0.57
P09429	HMGB1;HMGB1P1	127	High mobility group protein B1;Putative high mc	73.666	GEHPGLSIGDVAK(1)K	Tip60 OE only
P26583	HMGB1;HMGB1P1;H	50	High mobility group protein B1;Putative high mc	71.342	WK(1)TMSAK	0.82
P26583	HMGB1;HMGB1P1;H	55	High mobility group protein B1;Putative high mc	56.547	TMSAK(1)EK	Tip60 OE only
P09429	HMGB1;HMGB1P1;S	59	High mobility group protein B1;Putative high mc	94.662	GK(1)FEDMAK	1.04
P26583	HMGB1;HMGB2	157	High mobility group protein B1;High mobility gr	128.69	YEK(1)DIAAYR	1.04
P26583	HMGB2	141	High mobility group protein B2	77.282	DK(1)QPYEQK	0.89

P26583	HMGB2	59	High mobility group protein B2	90.37	SK(1)FEDMAK	0.89
P26583	HMGB2	114	High mobility group protein B2	81.526	IK(1)SEHPGLSIGDTAK	0.92
P26583	HMGB2	127	High mobility group protein B2	112.13	SEHPGLSIGDTAK(1)K	Tip60 OE only
Q01581	HMGCS1	46	Hydroxymethylglutaryl-CoA synthase, cytoplasm	62.14	YDGVDAGK(1)YTIGLGQAK	0.46
Q01581	HMGCS1	321	Hydroxymethylglutaryl-CoA synthase, cytoplasm	43.084	AFMK(1)ASSELFSQK	0.85
Q01581	HMGCS1	273	Hydroxymethylglutaryl-CoA synthase, cytoplasm	66.429	LVQK(1)SLAR	0.98
Q01581	HMGCS1	409	Hydroxymethylglutaryl-CoA synthase, cytoplasm	115.78	ITASLCDLK(1)SR	1.19
P09651	HNRNPA1	3	Heterogeneous nuclear ribonucleoprotein A1;He	116.55	SK(1)SESPKEPEQLR	0.97
P09651	HNRNPA1	8	Heterogeneous nuclear ribonucleoprotein A1;He	138.98	SESPK(1)EPEQLR	1.16
P09651	HNRNPA1;HNRNPA1	105	Heterogeneous nuclear ribonucleoprotein A1;He	64.439	EDSQRPGAHLTVK(1)K	1.01
P09651	HNRNPA1;HNRNPA1	78	Heterogeneous nuclear ribonucleoprotein A1;He	40.103	PHK(1)VDGR	1.03
P22626	HNRNPA2B1	112	Heterogeneous nuclear ribonucleoproteins A2/B	57.532	EESGKPGAHVTVK(1)K	0.82
P22626	HNRNPA2B1	104	Heterogeneous nuclear ribonucleoproteins A2/B	52.49	EESGK(1)PGAHVTVK	0.87
P22626	HNRNPA2B1	3	Heterogeneous nuclear ribonucleoproteins A2/B	63.32	MEK(1)TLETVPLER	Tip60 OE only
P51991	HNRNPA3	148	Heterogeneous nuclear ribonucleoprotein A3	55.676	DYFEK(1)YGK	0.9
P51991	HNRNPA3	134	Heterogeneous nuclear ribonucleoprotein A3	132.76	IFVGGIK(1)EDTEEYNLR	0.95
P51991	HNRNPA3	151	Heterogeneous nuclear ribonucleoprotein A3	104.79	YGK(1)IETIEVMEDR	1.01
P51991	HNRNPA3	126	Heterogeneous nuclear ribonucleoprotein A3	79.639	EDSVKPGAHLTVK(1)K	Tip60 OE only
Q99729	HNRNPAB	101	Heterogeneous nuclear ribonucleoprotein A/B	99.671	FGEVVDCTIK(1)MDPNTGR	0.82
Q99729	HNRNPAB	215	Heterogeneous nuclear ribonucleoprotein A/B	123.35	K(1)FHTVSGSK	0.96
Q99729	HNRNPAB	130	Heterogeneous nuclear ribonucleoprotein A/B	154.35	VLDQK(1)EHR	0.99
Q99729	HNRNPAB	86	Heterogeneous nuclear ribonucleoprotein A/B	50.04	DLK(1)DYFTK	1.13
Q99729	HNRNPAB	125	Heterogeneous nuclear ribonucleoprotein A/B	91.658	DAASVEK(1)VLDQK	Tip60 OE only
Q99729	HNRNPAB	82	Heterogeneous nuclear ribonucleoprotein A/B	94.616	MFVGGLSWDTSK(1)K	Tip60 OE only
Q99729	HNRNPAB	232	Heterogeneous nuclear ribonucleoprotein A/B	77.078	VAQPK(1)EVYQQQQYGSGGR	Tip60 OE only
P07910	HNRNPC	243	Heterogeneous nuclear ribonucleoproteins C1/C	72.006	SEEEQSSSSVK(1)K	0.93
P07910	HNRNPC	232	Heterogeneous nuclear ribonucleoproteins C1/C	85.288	NDK(1)SEEEQSSSSVK	1.07
P07910	HNRNPC	176	Heterogeneous nuclear ribonucleoproteins C1/C	109.11	SGFNSK(1)SGQR	1.16
P07910	HNRNPC	89	Heterogeneous nuclear ribonucleoproteins C1/C	94.688	MIAGQVLDINLAAEPK(1)VNR	Tip60 OE only
P07910	HNRNPC	8	Heterogeneous nuclear ribonucleoproteins C1/C	112.3	ASNVTNK(1)TDPR	unquantifiable
P07910	HNRNPC;HNRNPCL1;	219	Heterogeneous nuclear ribonucleoproteins C1/C	113.7	IEK(1)EQSK	1
P07910	HNRNPC;HNRNPCL1;	223	Heterogeneous nuclear ribonucleoproteins C1/C	54.982	EQSK(1)QAVEMK	Tip60 OE only
P07910	HNRNPC;HNRNPCL1;	197	Heterogeneous nuclear ribonucleoproteins C1/C	42.336	LKGDDLQAIK(1)K	0.66
P07910	HNRNPC;HNRNPCL1;	39	Heterogeneous nuclear ribonucleoproteins C1/C	123.63	SDVEAIFSK(1)YGK	0.75

P07910	HNRNPC;HNRNPCL1;	29	Heterogeneous nuclear ribonucleoproteins C1/C	120.68	VFIGNLNTLVVK(1)K	0.95
P07910	HNRNPC;HNRNPCL1;	30	Heterogeneous nuclear ribonucleoproteins C1/C	106.62	K(1)SDVEAIFSK	1.01
P07910	HNRNPC;HNRNPCL1;	189	Heterogeneous nuclear ribonucleoproteins C1/C	110.76	LK(1)GDDLQAIK	1.04
P07910	HNRNPC;HNRNPCL1;	204	Heterogeneous nuclear ribonucleoproteins C1/C	76.847	ELTQIK(1)QK	0.91
P07910	HNRNPC;HNRNPCL1;	42	Heterogeneous nuclear ribonucleoproteins C1/C	95.775	YGK(1)IVGCSVHK	1.03
P07910	HNRNPC;RALYL	50	Heterogeneous nuclear ribonucleoproteins C1/C	100.28	IVGCSVHK(1)GFAFVQYVNER	0.98
P07910	HNRNPC;RALYL	198	Heterogeneous nuclear ribonucleoproteins C1/C	75.189	K(1)ELTQIK	1.18
Q14103	HNRNPD	153	Heterogeneous nuclear ribonucleoprotein D0	102.06	ESESVDK(1)VMDQK	0.89
Q14103	HNRNPD	158	Heterogeneous nuclear ribonucleoprotein D0	141.78	VMDQK(1)EHK	0.9
Q14103	HNRNPD	251	Heterogeneous nuclear ribonucleoprotein D0	152.07	YHNVGLSK(1)CEIK	0.92
Q14103	HNRNPD	183	Heterogeneous nuclear ribonucleoprotein D0	77.593	K(1)IFVGGLSPDTPEEK	1.33
O14979	HNRNPDL	209	Heterogeneous nuclear ribonucleoprotein D-like	98.299	VLELK(1)EHK	0.88
O14979	HNRNPDL	311	Heterogeneous nuclear ribonucleoprotein D-like	123.69	VAQPK(1)EVYR	1.17
O14979	HNRNPDL	302	Heterogeneous nuclear ribonucleoprotein D-like	95.573	YHQIGSGK(1)CEIK	1.24
O14979	HNRNPDL	142	Heterogeneous nuclear ribonucleoprotein D-like	148.32	INASK(1)NQQDDGK	Tip60 OE only
P52597	HNRNPF	224	Heterogeneous nuclear ribonucleoprotein F;Hete	94.692	YIGIVK(1)QAGLER	0.91
P52597	HNRNPF	87	Heterogeneous nuclear ribonucleoprotein F;Hete	110.87	YIEVFK(1)SHR	0.92
P52597	HNRNPF	185	Heterogeneous nuclear ribonucleoprotein F;Hete	84.507	YIEVFK(1)SSQEEVR	1.06
P55795	HNRNPH2;HNRNPH1	349	Heterogeneous nuclear ribonucleoprotein H2;He	124.37	DK(1)ANMQHR	Tip60 OE only
P55795	HNRNPH2;HNRNPH1	185	Heterogeneous nuclear ribonucleoprotein H2;He	111.95	YIEIFK(1)SSR	unquantifiable
P61978	HNRNPK	60	Heterogeneous nuclear ribonucleoprotein K	97.431	NAGAVIGK(1)GGK	0.91
P61978	HNRNPK	52	Heterogeneous nuclear ribonucleoprotein K	134.68	ILLQSK(1)NAGAVIGK	0.95
P61978	HNRNPK	179	Heterogeneous nuclear ribonucleoprotein K	84.499	ENTQTTIK(1)LFQECCPHSTDR	0.98
P61978	HNRNPK	198	Heterogeneous nuclear ribonucleoprotein K	103.56	VVLIGGK(1)PDR	0.98
P61978	HNRNPK	163	Heterogeneous nuclear ribonucleoprotein K	77.506	LLIHQSLAGGIIGVK(1)GAK	1.03
P61978	HNRNPK	405	Heterogeneous nuclear ribonucleoprotein K	190.24	DLAGSIIGK(1)GGQR	1.07
P61978	HNRNPK	34	Heterogeneous nuclear ribonucleoprotein K	57.328	RPAEDMEEEQAFK(1)R	1.08
P14866	HNRNPL	533	Heterogeneous nuclear ribonucleoprotein L	87.568	RPSSVK(1)VFSGK	0.96
P14866	HNRNPL	493	Heterogeneous nuclear ribonucleoprotein L	78.334	FSTPEQAAK(1)NR	1.29
P52272	HNRNPM	672	Heterogeneous nuclear ribonucleoprotein M	115.8	DK(1)FNECGHVLYADIK	0.44
P52272	HNRNPM	242	Heterogeneous nuclear ribonucleoprotein M	70.977	ADILEDK(0.002)DGK(0.998):	0.73
P52272	HNRNPM	69	Heterogeneous nuclear ribonucleoprotein M	45.997	FEPYANPTK(1)R	0.75
P52272	HNRNPM	134	Heterogeneous nuclear ribonucleoprotein M	79.633	AAEVLNK(1)HSLSGR	0.82
P52272	HNRNPM	145	Heterogeneous nuclear ribonucleoprotein M	49.448	VK(1)EDPDGEHAR	0.84

P52272	HNRNPM	126	Heterogeneous nuclear ribonucleoprotein M	68.422	MEESMK(1)K	1.02
P52272	HNRNPM	239	Heterogeneous nuclear ribonucleoprotein M	108.98	ADILEDK(1)DGK	1.02
P52272	HNRNPM	88	Heterogeneous nuclear ribonucleoprotein M	48.794	WQSLK(1)DLVK	1.12
P52272	HNRNPM	277	Heterogeneous nuclear ribonucleoprotein M	70.908	PMHVK(1)MDER	1.12
P52272	HNRNPM	651	Heterogeneous nuclear ribonucleoprotein M	97.068	K(1)ACQIFVR	1.22
P52272	HNRNPM	698	Heterogeneous nuclear ribonucleoprotein M	125.74	GCGVVK(1)FESPEVAER	Tip60 OE only
P52272	HNRNPM	388	Heterogeneous nuclear ribonucleoprotein M	81.632	GEIIAK(1)QGGGGGGGSVPGI	Tip60 OE only
O43390	HNRNPR	103	Heterogeneous nuclear ribonucleoprotein R	55.426	SAFLCGVMK(1)TYR	0.21
O43390	HNRNPR	94	Heterogeneous nuclear ribonucleoprotein R	46.131	ESDLSHVQNK(1)SAFLCGVMK	0.95
O43390	HNRNPR	255	Heterogeneous nuclear ribonucleoprotein R	57.59	LFVGSIPK(1)NK	0.98
O43390	HNRNPR	366	Heterogeneous nuclear ribonucleoprotein R	96.331	SFSEFGK(1)LER	0.99
O43390	HNRNPR	259	Heterogeneous nuclear ribonucleoprotein R	121.9	TK(1)ENILEEFSK	1
O43390	HNRNPR	374	Heterogeneous nuclear ribonucleoprotein R	58.21	LK(1)DYAFVHFEDR	1.03
O43390	HNRNPR	41	Heterogeneous nuclear ribonucleoprotein R	70.488	TLIEAGLPQK(1)VAER	Tip60 OE only
O43390	HNRNPR;SYNCRIP	313	Heterogeneous nuclear ribonucleoprotein R;Hete	60.358	LMSGK(1)VK	1
O43390	HNRNPR;SYNCRIP	341	Heterogeneous nuclear ribonucleoprotein R;Hete	121.68	VK(1)VLFVR	1.05
Q00839	HNRNPU	265	Heterogeneous nuclear ribonucleoprotein U	140.3	GYFEYIEENK(1)YSR	0.62
Q00839	HNRNPU	9	Heterogeneous nuclear ribonucleoprotein U	74.127	SSSPVNVK(1)K	0.77
Q00839	HNRNPU	635	Heterogeneous nuclear ribonucleoprotein U	114.5	DLPEHAVLK(1)MK	0.77
Q00839	HNRNPU	664	Heterogeneous nuclear ribonucleoprotein U	98.033	EEAQK(1)LLEQYK	0.84
Q00839	HNRNPU	28	Heterogeneous nuclear ribonucleoprotein U	53.554	LSDK(1)GLK	0.85
Q00839	HNRNPU	12	Heterogeneous nuclear ribonucleoprotein U	87.323	LK(1)VSELK	0.87
Q00839	HNRNPU	516	Heterogeneous nuclear ribonucleoprotein U	138.89	TTWVTK(1)HAAENPGK	0.9
Q00839	HNRNPU	343	Heterogeneous nuclear ribonucleoprotein U	114.78	VTEK(1)IPVR	0.96
Q00839	HNRNPU	524	Heterogeneous nuclear ribonucleoprotein U	65.184	HAAENPGK(1)YNILGTNTIMDI	0.97
Q00839	HNRNPU	333	Heterogeneous nuclear ribonucleoprotein U	63.565	GK(1)VCFEMK	0.98
Q00839	HNRNPU	17	Heterogeneous nuclear ribonucleoprotein U	135.83	VSELK(1)EELK	1.03
Q00839	HNRNPU	614	Heterogeneous nuclear ribonucleoprotein U	121.68	DEDYK(1)QR	1.05
Q00839	HNRNPU	602	Heterogeneous nuclear ribonucleoprotein U	81.297	K(1)AVVVCPK	1.05
Q00839	HNRNPU	436	Heterogeneous nuclear ribonucleoprotein U	156.51	ISK(1)EVLAGR	1.05
Q00839	HNRNPU	387	Heterogeneous nuclear ribonucleoprotein U	186.46	GIK(1)TCNCETEDYGEK	1.1
Q00839	HNRNPU	352	Heterogeneous nuclear ribonucleoprotein U	174.39	HLYTK(1)DIDIHEVR	1.1
Q00839	HNRNPU	31	Heterogeneous nuclear ribonucleoprotein U	80.438	GLK(1)AELMER	Tip60 OE only
Q00839	HNRNPU	609	Heterogeneous nuclear ribonucleoprotein U	125.56	AVVVCPK(1)DEDYK	Tip60 OE only

Q00839	HNRNPU	186	Heterogeneous nuclear ribonucleoprotein U	77.746	EAAGK(1)SSGPTSLFAVTVAPP	Tip60 OE only
Q00839	HNRNPU	543	Heterogeneous nuclear ribonucleoprotein U	50.966	MMVAGFK(1)K	Tip60 OE only
Q5SSJ5	HP1BP3	160	Heterochromatin protein 1-binding protein 3	109.6	PK(1)MDAILTEAIK	0.48
P00492	HPRT1	159	Hypoxanthine-guanine phosphoribosyltransfera:	49.715	MVK(1)VASLLVK	0.91
P00492	HPRT1	103	Hypoxanthine-guanine phosphoribosyltransfera:	78.342	LK(1)SYCNDQSTGDIK	Tip60 OE only
Q99714	HSD17B10	79	3-hydroxyacyl-CoA dehydrogenase type-2	146.11	DVQTALALAK(1)GK	1.05
Q53GQ0	HSD17B12	86	Very-long-chain 3-oxoacyl-CoA reductase	126.1	SK(0.001)DK(0.999)LDQVSSI	0.8
Q53GQ0	HSD17B12	72	Very-long-chain 3-oxoacyl-CoA reductase	110.76	SYAEELAK(1)HGMK	0.83
Q53GQ0	HSD17B12	84	Very-long-chain 3-oxoacyl-CoA reductase	125.82	SK(1)DKLDQVSSEIK	0.86
Q53GQ0	HSD17B12	95	Very-long-chain 3-oxoacyl-CoA reductase	126.39	LDQVSSEIK(1)EK	0.96
Q53GQ0	HSD17B12	261	Very-long-chain 3-oxoacyl-CoA reductase	105.98	SAIK(1)TVGLQSR	1.04
P51659	HSD17B4	68	Peroxisomal multifunctional enzyme type 2;(3R)-	88.101	GGK(1)AVANYDSVEEGEK	0.8
P51659	HSD17B4	81	Peroxisomal multifunctional enzyme type 2;(3R)-	100.72	AVANYDSVEEGEK(1)VVK	Tip60 OE only
P07900	HSP90AA1	446	Heat shock protein HSP 90-alpha	78.486	NIK(1)LGIHEDSQNR	0.73
P07900	HSP90AA1	431	Heat shock protein HSP 90-alpha	70.235	KCLELFTELAEDK(1)ENYK	0.86
P07900	HSP90AA1	632	Heat shock protein HSP 90-alpha	153.72	K(1)HLEINPDHSIIETLR	0.87
P07900	HSP90AA1	436	Heat shock protein HSP 90-alpha	83.313	K(1)FYEQFSK	0.92
P07900	HSP90AA1	58	Heat shock protein HSP 90-alpha	242.88	ELISNSSDALDK(1)IR	0.93
P07900	HSP90AA1	443	Heat shock protein HSP 90-alpha	103.83	FYEQFSK(1)NIK	0.95
P07900	HSP90AA1	585	Heat shock protein HSP 90-alpha	144.77	KVEK(1)VVVSNR	0.95
P07900	HSP90AA1	489	Heat shock protein HSP 90-alpha	220.9	ENQK(1)HIYYITGETK	0.97
P07900	HSP90AA1	582	Heat shock protein HSP 90-alpha	94.363	K(0.903)VEK(0.097)VVVSNR	1
P07900	HSP90AA1	478	Heat shock protein HSP 90-alpha	190.27	YYTSASGDEMVSLK(1)DYCTR	1
P07900	HSP90AA1	631	Heat shock protein HSP 90-alpha	83.783	DNSTMGYMAAK(1)K	1.05
P07900	HSP90AA1	458	Heat shock protein HSP 90-alpha	114.72	K(1)LSELLR	1.05
P07900	HSP90AA1	576	Heat shock protein HSP 90-alpha	93.143	IMK(1)DILEK	1.07
P07900	HSP90AA1	499	Heat shock protein HSP 90-alpha	197.2	HIYYITGETK(1)DQVANSAFVEF	1.21
P07900	HSP90AA1;HSP90AA	74	Heat shock protein HSP 90-alpha;Heat shock pro	103.91	LDSGK(1)ELHINLIPNK	1.05
P08238	HSP90AA1;HSP90AA	286	Heat shock protein HSP 90-alpha;Heat shock pro	79.597	TK(1)PIWTR	1.03
P08238	HSP90AA1;HSP90AA	107	Heat shock protein HSP 90-alpha;Heat shock pro	192.86	ADLINNLGTIAK(1)SGTK	0.96
P08238	HSP90AA1;HSP90AA	275	Heat shock protein HSP 90-alpha;Heat shock pro	156.69	EK(1)YIDQEELNK	0.91
P08238	HSP90AA1;HSP90AA	186	Heat shock protein HSP 90-alpha;Heat shock pro	162.6	VILHLK(1)EDQTEYLEER	0.94
P08238	HSP90AA1;HSP90AA	180	Heat shock protein HSP 90-alpha;Heat shock pro	61.56	GTK(1)VILHLK	1
P08238	HSP90AA1;HSP90AA	64	Heat shock protein HSP 90-alpha;Heat shock pro	120.96	YESLTDPSK(1)LDSGK	0.95

P07900	HSP90AA1;HSP90AA	546	Heat shock protein HSP 90-alpha;Putative heat sł	187.63	TLVSVTK(1)EGLELPEDEEEK	0.88
P07900	HSP90AA1;HSP90AA	539	Heat shock protein HSP 90-alpha;Putative heat sł	102.52	EFEGK(1)TLVSVTK	0.94
P08238	HSP90AA1;HSP90AA	607	Heat shock protein HSP 90-alpha;Putative heat sł	95.793	IMK(1)AQALR	1.02
P07900	HSP90AA1;HSP90AA	567	Heat shock protein HSP 90-alpha;Putative heat sł	137.9	TK(1)FENLCK	0.87
P07900	HSP90AA1;HSP90AA	565	Heat shock protein HSP 90-alpha;Putative heat sł	81.95	K(0.902)TK(0.098)FENLCK	0.91
P07900	HSP90AA1;HSP90AA	559	Heat shock protein HSP 90-alpha;Putative heat sł	97.69	EGLELPEDEEEK(0.125)K(0.87	0.94
P07900	HSP90AA1;HSP90AA	558	Heat shock protein HSP 90-alpha;Putative heat sł	140.07	EGLELPEDEEEK(1)K	0.99
P08238	HSP90AA1;HSP90AB	399	Heat shock protein HSP 90-alpha;Heat shock prot	143.01	EMLQQSK(1)ILK	0.93
P08238	HSP90AB1	411	Heat shock protein HSP 90-beta	141.85	K(1)CLELFSELAEDKENYK	0.94
P08238	HSP90AB1	435	Heat shock protein HSP 90-beta	137.84	FYEAFSK(1)NLK	0.99
P08238	HSP90AB1	438	Heat shock protein HSP 90-beta	68.809	NLK(1)LGIHEDSTNR	1
P08238	HSP90AB1	481	Heat shock protein HSP 90-beta	101.71	ETQK(1)SIYYITGESK	1.04
P08238	HSP90AB1	557	Heat shock protein HSP 90-beta	66.993	MEESK(1)AK	1.1
P08238	HSP90AB1	491	Heat shock protein HSP 90-beta	222.16	SIYYITGESK(1)EQVANSAFVER	1.13
P08238	HSP90AB1	624	Heat shock protein HSP 90-beta	130.56	K(1)HLEINPDHPIVETLR	Tip60 OE only
P08238	HSP90AB1;HSP90AB	347	Heat shock protein HSP 90-beta;Putative heat sh	187	RAPFDLFENK(1)K	0.9
P08238	HSP90AB1;HSP90AB	551	Heat shock protein HSP 90-beta;Putative heat sh	97.69	EGLELPEDEEEK(0.125)K(0.87	0.9
P08238	HSP90AB1;HSP90AB	538	Heat shock protein HSP 90-beta;Putative heat sh	153.32	SLVSVTK(1)EGLELPEDEEEK	0.94
P08238	HSP90AB1;HSP90AB	550	Heat shock protein HSP 90-beta;Putative heat sh	140.07	EGLELPEDEEEK(1)K	0.95
P08238	HSP90AB1;HSP90AB	204	Heat shock protein HSP 90-beta;Putative heat sh	119.25	K(1)HSQFIGYPITLYLEK	1
P08238	HSP90AB1;HSP90AB	199	Heat shock protein HSP 90-beta;Putative heat sh	72.138	RVK(1)EVVK	1
P08238	HSP90AB1;HSP90AB	577	Heat shock protein HSP 90-beta;Putative heat sh	163.16	KVEK(1)VTISNR	1
P08238	HSP90AB1;HSP90AB	72	Heat shock protein HSP 90-beta;Putative heat sh	94.616	ELK(1)IDIIPNPQER	1.03
P08238	HSP90AB1;HSP90AB	574	Heat shock protein HSP 90-beta;Putative heat sh	70.856	EILDK(0.037)K(0.963)VEK	1.04
P08238	HSP90AB1;HSP90AB	531	Heat shock protein HSP 90-beta;Putative heat sh	99.973	EFDGK(1)SLVSVTK	1.08
P08238	HSP90AB1;HSP90AB	69	Heat shock protein HSP 90-beta;Putative heat sh	71.342	LDSGK(1)ELK	Tip60 OE only
P08238	HSP90AB1;HSP90AB	568	Heat shock protein HSP 90-beta;Putative heat sh	80.219	LMK(1)EILDK	1.13
P08238	HSP90AB1;HSP90AB	53	Heat shock protein HSP 90-beta;Putative heat sh	252.3	ELISNASDALDK(1)IR	0.99
Q58FF6	HSP90AB4P	161	Putative heat shock protein HSP 90-beta 4	162.6	VILHLK(1)EDQTEYLEER	0.91
Q58FF6	HSP90AB4P	458	Putative heat shock protein HSP 90-beta 4	140.07	EGLELPEDEEEK(1)K	0.92
P14625	HSP90B1	348	Endoplasmin	156.51	PSK(1)EVEEDEYK	0.86
P14625	HSP90B1	161	Endoplasmin	114.4	EELVK(1)NLGTIAK	0.93
P14625	HSP90B1	603	Endoplasmin	107.9	FDESEK(1)TK	0.95
P14625	HSP90B1	479	Endoplasmin	172.27	IADDK(1)YNDTFWK	0.95

P14625	HSP90B1	168	Endoplasmin	163.71	NLGTIAK(1)SGTSEFLNK	0.96
P14625	HSP90B1	733	Endoplasmin	110.08	SGYLLPDTK(1)AYGDR	0.97
P14625	HSP90B1	97	Endoplasmin	107.66	NK(1)EIFLR	0.97
P14625	HSP90B1	486	Endoplasmin	84.208	IADDKYNDTFWK(1)EFGTNIK	1.02
P14625	HSP90B1	633	Endoplasmin	116.37	IEK(1)AVVSQR	1.07
P14625	HSP90B1	356	Endoplasmin	111.65	EVEEDEYK(1)AFYK	1.15
P14625	HSP90B1	75	Endoplasmin	130.98	SEK(1)FAFQAEVNR	1.17
P14625	HSP90B1	537	Endoplasmin	81.475	QDK(1)IYFMAGSSR	1.19
P14625	HSP90B1	682	Endoplasmin	77.379	DISTNYYASQK(1)K	1.24
P14625	HSP90B1	142	Endoplasmin	106.82	EK(1)NLLHVTDTGVGMTR	1.26
P14625	HSP90B1;HSP90B2P	683	Endoplasmin;Putative endoplasmin-like protein	108.46	K(1)TFEINPR	0.71
P14625	HSP90B1;HSP90B2P	473	Endoplasmin;Putative endoplasmin-like protein	67.081	KTLDMIK(1)K	0.79
P14625	HSP90B1;HSP90B2P	404	Endoplasmin;Putative endoplasmin-like protein	124.08	GLFDEYGSK(1)K	0.89
P14625	HSP90B1;HSP90B2P	597	Endoplasmin;Putative endoplasmin-like protein	83.783	EGVK(1)FDESEK	0.9
P14625	HSP90B1;HSP90B2P	467	Endoplasmin;Putative endoplasmin-like protein	82.671	K(1)TLDMIK	0.95
P14625	HSP90B1;HSP90B2P	493	Endoplasmin;Putative endoplasmin-like protein	44.567	EFGTNIK(1)LGVIEDHSNR	0.95
P14625	HSP90B1;HSP90B2P	593	Endoplasmin;Putative endoplasmin-like protein	135.83	FQNVAK(1)EGVK	0.97
P14625	HSP90B1;HSP90B2P	455	Endoplasmin;Putative endoplasmin-like protein	146.58	ETLQQHK(1)LLK	0.98
P14625	HSP90B1;HSP90B2P	663	Endoplasmin;Putative endoplasmin-like protein	144.77	IMK(1)AQAYQTGK	1.03
P14625	HSP90B1;HSP90B2P	410	Endoplasmin;Putative endoplasmin-like protein	47.187	K(0.001)SDYIK(0.999)LYVR	1.08
P14625	HSP90B1;HSP90B2P	671	Endoplasmin;Putative endoplasmin-like protein	124.3	AQAYQTGK(1)DISTNYYASQK	1.09
P0DMV9	HSPA1B;HSPA1A	246	Heat shock 70 kDa protein 1B;Heat shock 70 kDa	155.5	LVNHFVEEFK(1)R	0.81
P0DMV9	HSPA1B;HSPA1A	539	Heat shock 70 kDa protein 1B;Heat shock 70 kDa	146.16	VSAK(1)NALESYAFNMK	0.85
P0DMV9	HSPA1B;HSPA1A	56	Heat shock 70 kDa protein 1B;Heat shock 70 kDa	151.56	LIGDAAK(1)NQVALNPQNTVF	0.91
P0DMV9	HSPA1B;HSPA1A	71	Heat shock 70 kDa protein 1B;Heat shock 70 kDa	262.56	NQVALNPQNTVFDAK(1)R	0.94
P0DMV9	HSPA1B;HSPA1A	328	Heat shock 70 kDa protein 1B;Heat shock 70 kDa	186.08	LDK(1)AQIHDLVLVGGSTR	1
P0DMV9	HSPA1B;HSPA1A	524	Heat shock 70 kDa protein 1B;Heat shock 70 kDa	148.38	MVQEAEK(1)YK	1.01
P0DMV9	HSPA1B;HSPA1A	550	Heat shock 70 kDa protein 1B;Heat shock 70 kDa	130.24	NALESYAFNMK(1)SAVEDEGLI	1.02
P0DMV9	HSPA1B;HSPA1A	512	Heat shock 70 kDa protein 1B;Heat shock 70 kDa	105.57	LSK(1)EEIER	1.02
P0DMV9	HSPA1B;HSPA1A	526	Heat shock 70 kDa protein 1B;Heat shock 70 kDa	168.23	YK(1)AEDEVQR	1.02
P0DMV9	HSPA1B;HSPA1A	88	Heat shock 70 kDa protein 1B;Heat shock 70 kDa	73.389	FGDPVVQSDMK(1)HWPFQVI	1.03
P0DMV9	HSPA1B;HSPA1A	595	Heat shock 70 kDa protein 1B;Heat shock 70 kDa	121.29	DEFEHK(1)R	1.03
P0DMV9	HSPA1B;HSPA1A	77	Heat shock 70 kDa protein 1B;Heat shock 70 kDa	124.42	K(1)FGDPVVQSDMK	1.1
P0DMV9	HSPA1B;HSPA1A	257	Heat shock 70 kDa protein 1B;Heat shock 70 kDa	101.33	DISQNK(1)R	1.39

P0DMV9	HSPA1B;HSPA1A	597	Heat shock 70 kDa protein 1B;Heat shock 70 kDa	131.81	RK(1)ELEQVCNPIISGLYQGAG	Tip60 OE only
P0DMV9	HSPA1B;HSPA1A	3	Heat shock 70 kDa protein 1B;Heat shock 70 kDa	180.49	AK(1)AAAIGIDLGTTYSCVGVF(	Tip60 OE only
P0DMV9	HSPA1B;HSPA1A	126	Heat shock 70 kDa protein 1B;Heat shock 70 kDa	77.597	AFYPEEISSMVLTK(1)MK	Tip60 OE only
P0DMV9	HSPA1B;HSPA1A;HSF	500	Heat shock 70 kDa protein 1B;Heat shock 70 kDa	130.31	ANK(1)ITITNDK	0.81
P0DMV9	HSPA1B;HSPA1A;HSF	507	Heat shock 70 kDa protein 1B;Heat shock 70 kDa	174.27	ITITNDK(1)GR	1.05
P0DMV9	HSPA1B;HSPA1A;HSF	451	Heat shock 70 kDa protein 1B;Heat shock 70 kDa	118.71	AMTK(1)DNNLLGR	1.16
P0DMV9	HSPA1B;HSPA1A;HSF	319	Heat shock 70 kDa protein 1B;Heat shock 70 kDa	161.79	STLEPVEK(1)ALR	1.13
P34932	HSPA4	53	Heat shock 70 kDa protein 4	191.31	SIGAAAK(1)SQVISNAK	0.85
P34932	HSPA4	679	Heat shock 70 kDa protein 4	84.213	LAELK(1)NLGQPIK	0.87
P34932	HSPA4	126	Heat shock 70 kDa protein 4	115.71	LK(1)ETAESVLK	0.89
P34932	HSPA4	356	Heat shock 70 kDa protein 4	50.966	ISK(1)FFGK	0.92
P34932	HSPA4	329	Heat shock 70 kDa protein 4	107.66	SVLEQTK(1)LK	0.95
P34932	HSPA4	305	Heat shock 70 kDa protein 4	116.23	GK(1)FLEMCNDLLAR	0.99
P34932	HSPA4	668	Heat shock 70 kDa protein 4	60.036	LEDTENWLYEDGEDQPK(1)Q\	1.04
P34932	HSPA4	717	Heat shock 70 kDa protein 4	58.917	IISSFK(1)NK	1.11
P34932	HSPA4	686	Heat shock 70 kDa protein 4	82.671	NLGQPIK(1)IR	1.21
P34932	HSPA4	557	Heat shock 70 kDa protein 4	75.479	AESEEMETSQAGSK(1)DK	1.27
P34932	HSPA4	61	Heat shock 70 kDa protein 4	128.67	SQVISNAK(1)NTVQGFK	Tip60 OE only
P34932	HSPA4	754	Heat shock 70 kDa protein 4	101.47	LNLQNK(1)QSLTMDPVVK	Tip60 OE only
O95757	HSPA4L	774	Heat shock 70 kDa protein 4L	107.57	VSEIVAK(1)SK	0.63
O95757	HSPA4L	351	Heat shock 70 kDa protein 4L	75.387	IPAVK(1)EQITK	0.76
O95757	HSPA4L	708	Heat shock 70 kDa protein 4L	81.381	K(1)IQLVMK	0.86
O95757	HSPA4L	578	Heat shock 70 kDa protein 4L	91.961	VK(1)SIDLPIQSSLCR	Tip60 OE only
O95757	HSPA4L	564	Heat shock 70 kDa protein 4L	103.56	SAVSDK(1)QDR	Tip60 OE only
P34932	HSPA4L;HSPA4;HSPH	609	Heat shock 70 kDa protein 4L;Heat shock 70 kDa	95.099	MIMQDK(1)LEK	1.03
P34932	HSPA4L;HSPA4;HSPH	388	Heat shock 70 kDa protein 4L;Heat shock 70 kDa	103.91	GCALQCAILSPAFK(1)VR	Tip60 OE only
P11021	HSPA5	340	78 kDa glucose-regulated protein	43.814	STMK(1)PVQK	0.74
P11021	HSPA5	81	78 kDa glucose-regulated protein	157.23	LIGDAAK(1)NQLTSNPENTVFD	0.85
P11021	HSPA5	154	78 kDa glucose-regulated protein	116.23	MK(1)ETAEAYLGK	0.86
P11021	HSPA5	547	78 kDa glucose-regulated protein	113.35	MVNDAEK(1)FAEEDKK	0.88
P11021	HSPA5	447	78 kDa glucose-regulated protein	156.08	K(1)SQIFSTASDNQPTVTIK	0.89
P11021	HSPA5	579	78 kDa glucose-regulated protein	67.169	NQIGDK(1)EK	0.89
P11021	HSPA5	370	78 kDa glucose-regulated protein	89.752	IPK(1)IQQLVK	0.91
P11021	HSPA5	617	78 kDa glucose-regulated protein	108.75	IEWLESHQDADIEDFK(1)AK	0.94

P11021	HSPA5	213	78 kDa glucose-regulated protein	134.98	IINEPTAAAIAYGLDK(1)R	0.95
P11021	HSPA5	553	78 kDa glucose-regulated protein	90.986	FAEEDK(1)K	0.95
P11021	HSPA5	96	78 kDa glucose-regulated protein	238.87	NQLTSNPENTVFDAK(1)R	0.97
P11021	HSPA5	352	78 kDa glucose-regulated protein	94.302	VLEDSDLK(1)K	0.99
P11021	HSPA5	353	78 kDa glucose-regulated protein	147.73	K(1)SDIDEIVLVGGSTR	1.15
P11021	HSPA5	326	78 kDa glucose-regulated protein	107.24	AK(1)FEELNMDLFR	1.24
P11021	HSPA5	516	78 kDa glucose-regulated protein	96.89	VTAEDK(1)GTGNK	1.25
P11021	HSPA5	344	78 kDa glucose-regulated protein	49.606	STMK(0.156)PVQK(0.844)VL	Tip60 OE only
P11021	HSPA5	573	78 kDa glucose-regulated protein	53.278	NELESYAYSLK(0.992)NQIGDK	Tip60 OE only
P11142	HSPA8	539	Heat shock cognate 71 kDa protein	89.548	VSSK(1)NSLESYAFNMK	0.74
P11142	HSPA8	524	Heat shock cognate 71 kDa protein	148.38	MVQEAEK(1)YK	0.82
P11142	HSPA8	56	Heat shock cognate 71 kDa protein	151.9	LIGDAAK(1)NQVAMNPTNTVF	0.92
P11142	HSPA8	589	Heat shock cognate 71 kDa protein	134.21	NQTAEK(1)EEFEHQQK	0.93
P11142	HSPA8	128	Heat shock cognate 71 kDa protein	112.36	MK(1)EIAEAYLGK	0.95
P11142	HSPA8	71	Heat shock cognate 71 kDa protein	281.99	NQVAMNPTNTVFDAK(1)R	1.01
P11142	HSPA8	512	Heat shock cognate 71 kDa protein	105.57	LSK(1)EDIER	1.02
P11142	HSPA8	319	Heat shock cognate 71 kDa protein	148.52	GTLDPVEK(1)ALR	1.02
P11142	HSPA8	126	Heat shock cognate 71 kDa protein	146.07	SFYPEEVSSMVLTK(1)MK	1.03
P11142	HSPA8	328	Heat shock cognate 71 kDa protein	215	LDK(1)SQIHDIVLVGGSTR	1.1
P11142	HSPA8	246	Heat shock cognate 71 kDa protein	78.488	MVNHFIAEFK(1)R	Tip60 OE only
P11142	HSPA8;HSPA2	507	Heat shock cognate 71 kDa protein;Heat shock-re	174.27	ITITNDK(1)GR	0.86
P11142	HSPA8;HSPA2	108	Heat shock cognate 71 kDa protein;Heat shock-re	98.233	VQVEYK(1)GETK	0.93
P11142	HSPA8;HSPA2	187	Heat shock cognate 71 kDa protein;Heat shock-re	136.99	IINEPTAAAIAYGLDK(1)K	0.94
P11142	HSPA8;HSPA2	451	Heat shock cognate 71 kDa protein;Heat shock-re	85.676	AMTK(1)DNNLLGK	0.95
P11142	HSPA8;HSPA2	112	Heat shock cognate 71 kDa protein;Heat shock-re	65.219	GETK(1)SFYPEEVSSMVLTK	1.02
P11142	HSPA8;HSPA2	102	Heat shock cognate 71 kDa protein;Heat shock-re	119.41	PK(1)VQVEYK	1.02
P38646	HSPA9	368	Stress-70 protein, mitochondrial	122.42	TIAPCQK(1)AMQDAEVSK	0.86
P38646	HSPA9	106	Stress-70 protein, mitochondrial	114.54	LVGMPAK(1)R	0.91
P38646	HSPA9	612	Stress-70 protein, mitochondrial	104.21	LK(1)EEISK	0.97
P38646	HSPA9	600	Stress-70 protein, mitochondrial	136.38	MEEFK(1)DQLPADECNK	0.97
P38646	HSPA9	625	Stress-70 protein, mitochondrial	148.5	K(1)DSETGENIR	1
P38646	HSPA9	175	Stress-70 protein, mitochondrial	136.83	MK(1)ETAENYLGHTAK	1.03
P38646	HSPA9	300	Stress-70 protein, mitochondrial	188.28	ETGVDLTK(1)DNMALQR	1.08
P38646	HSPA9	617	Stress-70 protein, mitochondrial	100.07	EEISK(1)MR	1.1

P38646	HSPA9	467	Stress-70 protein, mitochondrial	52.716	NTTIPTK(1)K	1.33
P38646	HSPA9	345	Stress-70 protein, mitochondrial	62.546	HLNMK(1)LTR	Tip60 OE only
P04792	HSPB1	198	Heat shock protein beta-1	99.531	AQLGGPEAAK(1)SDETAAK	1.03
P10809	HSPD1	301	60 kDa heat shock protein, mitochondrial	133.32	VGLQVVAVK(1)APGFGDNR	0.73
P10809	HSPD1	202	60 kDa heat shock protein, mitochondrial	140.78	GVITVK(1)DGK	0.73
P10809	HSPD1	359	60 kDa heat shock protein, mitochondrial	65.252	DDAMLLK(1)GK	0.74
P10809	HSPD1	396	60 kDa heat shock protein, mitochondrial	69.276	LAK(1)LSDGVAVLK	0.81
P10809	HSPD1	352	60 kDa heat shock protein, mitochondrial	116.87	VGEVIVTK(1)DDAMLLK	0.84
P10809	HSPD1	218	60 kDa heat shock protein, mitochondrial	170.95	TLNDELEIIEGMK(1)FDR	0.86
P10809	HSPD1	87	60 kDa heat shock protein, mitochondrial	60.76	SIDLK(1)DK	0.89
P10809	HSPD1	133	60 kDa heat shock protein, mitochondrial	102.52	ISK(1)GANPVEIR	0.89
P10809	HSPD1	130	60 kDa heat shock protein, mitochondrial	63.419	EGFEK(1)ISK	0.9
P10809	HSPD1	75	60 kDa heat shock protein, mitochondrial	103.55	VTK(1)DGVTVAK	0.9
P10809	HSPD1	473	60 kDa heat shock protein, mitochondrial	66.962	TLK(1)IPAMTIAK	0.92
P10809	HSPD1	481	60 kDa heat shock protein, mitochondrial	189.1	IPAMTIAK(1)NAGVEGSLIVEK	0.93
P10809	HSPD1	469	60 kDa heat shock protein, mitochondrial	148.07	IGIEIIK(1)R	0.94
P10809	HSPD1	125	60 kDa heat shock protein, mitochondrial	80.438	SIAK(1)EGFEK	0.94
P10809	HSPD1	417	60 kDa heat shock protein, mitochondrial	221.5	VGGTSDVEVNEK(1)K	0.96
P10809	HSPD1	91	60 kDa heat shock protein, mitochondrial	67.035	YK(1)NIGAK	0.97
P10809	HSPD1	196	60 kDa heat shock protein, mitochondrial	86.794	K(1)GVITVK	0.98
P10809	HSPD1	364	60 kDa heat shock protein, mitochondrial	197.21	GDK(1)AQIEK	0.98
P10809	HSPD1	523	60 kDa heat shock protein, mitochondrial	68.224	GIIDPTK(1)VVR	0.99
P10809	HSPD1	31	60 kDa heat shock protein, mitochondrial	61.602	DVK(1)FGADAR	1.05
P10809	HSPD1	236	60 kDa heat shock protein, mitochondrial	137.59	GQK(1)CEFQDAYVLLSEK	1.14
P10809	HSPD1	191	60 kDa heat shock protein, mitochondrial	60.102	EIGNIISDAMK(1)K	1.15
P10809	HSPD1	250	60 kDa heat shock protein, mitochondrial	135.09	K(1)ISSIQSIVPALEIANAHR	1.66
P10809	HSPD1	82	60 kDa heat shock protein, mitochondrial	87.298	DGVTVAK(1)SIDLK	Tip60 OE only
P10809	HSPD1	405	60 kDa heat shock protein, mitochondrial	91.207	LSDGVAVLK(1)VGGTSDVEVN	Tip60 OE only
P61604	HSPE1	28	10 kDa heat shock protein, mitochondrial	144.28	SAAETVTK(1)GGIMLPEK	0.94
P61604	HSPE1	36	10 kDa heat shock protein, mitochondrial	75.294	GGIMLPEK(1)SQGK	0.95
P61604	HSPE1	66	10 kDa heat shock protein, mitochondrial	110.66	GGEIQPVSVK(1)VGDK	1
P61604	HSPE1	56	10 kDa heat shock protein, mitochondrial	143.24	GK(1)GGEIQPVSVK	1
P61604	HSPE1	8	10 kDa heat shock protein, mitochondrial	107.66	K(1)FLPLFDR	1.03
P61604	HSPE1	70	10 kDa heat shock protein, mitochondrial	60.628	VGDK(1)VLLPEYGGTK	Tip60 OE only

P61604	HSPE1	80	10 kDa heat shock protein, mitochondrial	111.39	VLLPEYGGTK(1)VVLDDK	Tip60 OE only
Q92598	HSPH1	126	Heat shock protein 105 kDa	81.338	LK(1)ETAENSLK	0.93
Q92598	HSPH1	351	Heat shock protein 105 kDa	81.297	IPAVK(1)ER	0.97
Q92598	HSPH1	703	Heat shock protein 105 kDa	107.43	IGTPVK(1)VR	1.08
Q92598	HSPH1	648	Heat shock protein 105 kDa	102.51	DK(1)LCGPYEK	Tip60 OE only
Q92598	HSPH1	300	Heat shock protein 105 kDa	53.554	DVSGK(1)MNR	Tip60 OE only
O43719	HTATSF1	297	HIV Tat-specific factor 1	111.95	VECSK(1)FGQIR	Tip60 OE only
Q9Y4L1	HYOU1	797	Hypoxia up-regulated protein 1	66.799	EK(1)LAELR	1.31
Q9Y4L1	HYOU1	894	Hypoxia up-regulated protein 1	67.334	DIEAK(1)MMALDR	Tip60 OE only
P41252	IARS	382	Isoleucine--tRNA ligase, cytoplasmic	47.849	TLK(1)EQGR	1.02
P41252	IARS	132	Isoleucine--tRNA ligase, cytoplasmic	92.428	YSAEWK(1)STVSR	1.35
P41252	IARS	641	Isoleucine--tRNA ligase, cytoplasmic	63.148	FK(1)EEGVR	Tip60 OE only
P41252	IARS	844	Isoleucine--tRNA ligase, cytoplasmic	45.081	TIPIK(1)YPLK	Tip60 OE only
Q9NSE4	IARS2	810	Isoleucine--tRNA ligase, mitochondrial	46.158	LYCEK(1)ENDPK	0.84
Q9NSE4	IARS2	775	Isoleucine--tRNA ligase, mitochondrial	65.627	ITELYK(1)QYDFGK	Tip60 OE only
P50213	IDH3A	177	Isocitrate dehydrogenase [NAD] subunit alpha, m	114.28	LITEGASK(1)R	0.85
P50213	IDH3A	214	Isocitrate dehydrogenase [NAD] subunit alpha, m	144.09	MSDGLFLQK(1)CR	1.04
P50213	IDH3A	58	Isocitrate dehydrogenase [NAD] subunit alpha, m	79.82	IFDAAK(1)APIQWEER	Tip60 OE only
O43837	IDH3B	135	Isocitrate dehydrogenase [NAD] subunit beta, mi	62.694	K(1)LDLFANVVHVK	1
O43837	IDH3B	105	Isocitrate dehydrogenase [NAD] subunit beta, mi	90.913	LEQVLSSMK(1)ENK	Tip60 OE only
O43837	IDH3B;IDH3G	218	Isocitrate dehydrogenase [NAD] subunit beta, mi	117.7	VTAVHK(1)ANIMK	1.15
Q9NZI8	IGF2BP1	242	Insulin-like growth factor 2 mRNA-binding protei	73.036	ENAGAAEK(1)AISVHSTPEGCS	0.69
Q9NZI8	IGF2BP1	266	Insulin-like growth factor 2 mRNA-binding protei	62.303	MILEIMHK(1)EAK	1.02
Q9NZI8	IGF2BP1	272	Insulin-like growth factor 2 mRNA-binding protei	103.26	DTK(1)TADEVPLK	1.08
Q9NZI8	IGF2BP1	475	Insulin-like growth factor 2 mRNA-binding protei	57.859	LK(1)EENFFGPK	1.1
Q9NZI8	IGF2BP1	330	Insulin-like growth factor 2 mRNA-binding protei	52.579	TITVK(1)GAIENCCR	1.39
Q9NZI8	IGF2BP1;IGF2BP3;IG	465	Insulin-like growth factor 2 mRNA-binding protei	85.288	MVIITGPPEAQFK(1)AQGR	Tip60 OE only
Q9Y6M1	IGF2BP2	497	Insulin-like growth factor 2 mRNA-binding protei	61.679	LK(1)EENFFNPK	3.01
Q12905	ILF2	59	Interleukin enhancer-binding factor 2	48.883	VKPAPDETSFSEALLK(1)R	0.88
Q12906	ILF3	413	Interleukin enhancer-binding factor 3	113.94	LNQLK(1)PGLQYK	0.76
Q12906	ILF3	81	Interleukin enhancer-binding factor 3	140.07	EGAGEQK(1)TEHMTR	0.98
Q12906	ILF3	161	Interleukin enhancer-binding factor 3	64.723	NTK(1)EPPLSLTIHLTSPVVR	1
Q12906	ILF3	100	Interleukin enhancer-binding factor 3	96.711	VGLVAK(1)GLLLK	1.01
Q12906	ILF3	202	Interleukin enhancer-binding factor 3	88.681	QK(1)CLAALASLR	1.15

Q12906	ILF3	43	Interleukin enhancer-binding factor 3	93.345	ALK(1)AVSDWIDEQEK	1.27
Q12906	ILF3	577	Interleukin enhancer-binding factor 3	66.262	VAK(1)AYAALAALEK	Tip60 OE only
Q12906	ILF3	600	Interleukin enhancer-binding factor 3	91.937	LFPDTPLALDANK(1)K	Tip60 OE only
Q12906	ILF3;STRBP	553	Interleukin enhancer-binding factor 3;Spermatid	63.709	YELISETGGSHDK(1)R	0.95
Q12906	ILF3;STRBP	454	Interleukin enhancer-binding factor 3;Spermatid	65.465	TAK(1)LHVAVK	0.97
Q12906	ILF3;ZFR;STRBP	214	Interleukin enhancer-binding factor 3;Zinc finger	119.56	HAK(1)WFQAR	1.19
Q16891	IMMT	110	MICOS complex subunit MIC60	78.763	SIQSGPLK(1)ISSVSEVMK	0.68
Q16891	IMMT	299	MICOS complex subunit MIC60	76.774	AK(1)EELEK	0.87
Q16891	IMMT	726	MICOS complex subunit MIC60	72.607	VAQDWLK(1)EAR	0.92
Q16891	IMMT	423	MICOS complex subunit MIC60	129.42	ELAEQK(1)ATEK	0.95
Q16891	IMMT	297	MICOS complex subunit MIC60	107.83	AVDEAADALLK(1)AK	1.09
Q16891	IMMT	313	MICOS complex subunit MIC60	77.674	SVIENAK(1)K	1.25
Q16891	IMMT	269	MICOS complex subunit MIC60	87.789	AAMDNSEIAGEK(1)K	Tip60 OE only
Q16891	IMMT	211	MICOS complex subunit MIC60	141.02	LAQQEK(1)QEQVK	unquantifiable
P12268	IMPDH2	438	Inosine-5'-monophosphate dehydrogenase 2	110.08	IK(1)VAQGVSGAVQDK	0.56
P12268	IMPDH2	134	Inosine-5'-monophosphate dehydrogenase 2	77.674	DVFEAK(1)AR	0.85
P12268	IMPDH2	450	Inosine-5'-monophosphate dehydrogenase 2	74.147	VAQGVSGAVQDK(1)GSIHK	0.89
P12268	IMPDH2	349	Inosine-5'-monophosphate dehydrogenase 2	82.543	PQATAVYK(1)VSEYAR	1.1
O00410	IPO5	961	Importin-5	95.502	VIQSADSK(1)TK	0.7
O00410	IPO5	800	Importin-5	59.245	AK(1)LEEHFK	0.9
O00410	IPO5	1059	Importin-5	70.912	HEDPCAK(1)R	1.03
O00410	IPO5	693	Importin-5	84.169	ELK(1)EGFVEYTEQVVK	1.29
O95373	IPO7	774	Importin-7	44.395	EVK(1)TSELR	1.03
O95373	IPO7	314	Importin-7	47.849	EK(1)QYMAPR	Tip60 OE only
Q96P70	IPO9	773	Importin-9	96.89	LVSTLISK(1)AGR	0.6
P46940	IQGAP1	924	Ras GTPase-activating-like protein IQGAP1	83.087	NK(1)ITLQDVVSHSK	0.82
P46940	IQGAP1	806	Ras GTPase-activating-like protein IQGAP1	40.067	SHK(1)DEVVK	0.91
P46940	IQGAP1	1458	Ras GTPase-activating-like protein IQGAP1	65.535	K(0.035)EK(0.965)IQTGLK	0.97
P46940	IQGAP1	1455	Ras GTPase-activating-like protein IQGAP1	64.841	EDSNLTLQEK(1)K	Tip60 OE only
P46940	IQGAP1	1465	Ras GTPase-activating-like protein IQGAP1	64.103	K(1)LTELGTVDPK	unquantifiable
P46940	IQGAP1;IQGAP3	1035	Ras GTPase-activating-like protein IQGAP1;Ras G	89.247	TALQEEIK(1)SK	0.81
P46940	IQGAP1;IQGAP3	1037	Ras GTPase-activating-like protein IQGAP1;Ras G	143.56	SK(1)VDQIQEIVTGNPTVIK	0.84
P46940	IQGAP1;IQGAP3	1027	Ras GTPase-activating-like protein IQGAP1;Ras G	99.855	LFK(1)TALQEEIK	0.98
Q9NWZ3	IRAK4	34	Interleukin-1 receptor-associated kinase 4	66.994	K(1)LAVAIK	unquantifiable

Q9NPH2	ISYNA1	393	Inositol-3-phosphate synthase 1	56.563	YVPYVGDSK(1)R	0.9
Q9BY32	ITPA	8	Inosine triphosphate pyrophosphatase	81.297	AASLVGK(1)K	unquantifiable
P26440	IVD	401	Isovaleryl-CoA dehydrogenase, mitochondrial	85.288	DAK(1)LYEIGAGTSEVR	0.85
Q15046	KARS	479	Lysine--tRNA ligase	76.478	SK(1)EGLTER	1.2
Q92993	KAT5	451	Histone acetyltransferase KAT5	84.573	SESGERPQITINEISEITSIK(1)K	Tip60 OE only
Q92993	KAT5	41	Histone acetyltransferase KAT5	73.848	K(1)LFYVHYIDFNK	Tip60 OE only
Q07666	KHDRBS1	194	KH domain-containing, RNA-binding, signal trans	83.182	LQEETGAK(1)ISVLGK	0.72
Q07666	KHDRBS1	200	KH domain-containing, RNA-binding, signal trans	106.68	ISVLGK(1)GSMR	1.12
Q07666	KHDRBS1;KHDRBS2	165	KH domain-containing, RNA-binding, signal trans	75.38	VLIPVK(1)QYPK	0.91
Q92945	KHSRP	359	Far upstream element-binding protein 2	104.79	IQFK(1)QDDGTGPEK	0.97
Q96AE4	KHSRP;FUBP1	203	Far upstream element-binding protein 2;Far upst	57.836	AGLVIGK(1)GGETIK	0.88
Q96AE4	KHSRP;FUBP1	209	Far upstream element-binding protein 2;Far upst	105.4	GGETIK(1)QLQER	0.91
Q96AE4	KHSRP;FUBP1;FUBP3	300	Far upstream element-binding protein 2;Far upst	94.114	K(1)IQNDAGVR	1.01
Q9BY89	KIAA1671	568	Uncharacterized protein KIAA1671	48.561	DK(1)SRQTEQK	unquantifiable
O95239	KIF4A	536	Chromosome-associated kinesin KIF4A	56.729	ALALK(1)EALAR	unquantifiable
P33176	KIF5B	512	Kinesin-1 heavy chain	99.802	SQEVEDK(1)TK	0.76
P33176	KIF5B	690	Kinesin-1 heavy chain	140.3	EHLNK(1)VQTANEVK	0.81
P33176	KIF5B	514	Kinesin-1 heavy chain	138.75	TK(1)EYELLSDELNQK	0.89
P33176	KIF5B	726	Kinesin-1 heavy chain	53.166	DEVEAK(1)AK	0.92
P33176	KIF5B	829	Kinesin-1 heavy chain	134.08	SAEIDSDDTGGSAAQK(1)QK	0.99
P33176	KIF5B	450	Kinesin-1 heavy chain	187.44	LK(1)TQMLDQEELLASTR	1.04
P33176	KIF5B	485	Kinesin-1 heavy chain	91.937	LQAENDASK(1)EEVK	Tip60 OE only
P33176	KIF5B	753	Kinesin-1 heavy chain	52.268	VEHEK(1)LK	Tip60 OE only
P33176	KIF5B	656	Kinesin-1 heavy chain	131.28	SLTEYLQNVEQK(1)K	unquantifiable
P33176	KIF5B;KIF5C	642	Kinesin-1 heavy chain;Kinesin heavy chain isofori	54.066	ISQHEAK(1)IK	0.81
P33176	KIF5B;KIF5C	596	Kinesin-1 heavy chain;Kinesin heavy chain isofori	57.559	LYISK(1)MK	0.93
P33176	KIF5B;KIF5C	882	Kinesin-1 heavy chain;Kinesin heavy chain isofori	96.948	ALESALK(1)EAK	1.14
P33176	KIF5B;KIF5C;KIF5A	252	Kinesin-1 heavy chain;Kinesin heavy chain isofori	56.205	TGAEGAVLDEAK(1)NINK	0.87
P33176	KIF5B;KIF5C;KIF5A	240	Kinesin-1 heavy chain;Kinesin heavy chain isofori	81.565	VSK(1)TGAEGAVLDEAK	Tip60 OE only
Q07866	KLC1	27	Kinesin light chain 1	115.78	LTQDEIISK(1)TK	unquantifiable
Q9UMN6	KMT2B	2441	Histone-lysine N-methyltransferase 2B	42.864	TLIEK(1)VQEAR	unquantifiable
Q14974	KPNB1	73	Importin subunit beta-1	182.76	DPDIK(1)AQYQQR	0.84
Q14974	KPNB1	835	Importin subunit beta-1	82.75	DVLK(1)LVEAR	1
Q14974	KPNB1	211	Importin subunit beta-1	116.25	ANFDK(1)ESER	1.05

Q14974	KPNB1	23	Importin subunit beta-1	100.88	LELEAAQK(1)FLER	Tip60 OE only
Q14974	KPNB1	376	Importin subunit beta-1	65.305	EHIK(1)NPDWR	Tip60 OE only
Q13601	KRR1	168	KRR1 small subunit processome component hom	47.853	LIGPK(1)GSTLK	unquantifiable
P05783	KRT18	417	Keratin, type I cytoskeletal 18	64.121	IVDGK(1)VVSETNDTK	0.94
P05783	KRT18	426	Keratin, type I cytoskeletal 18	193.82	VVSETNDTK(1)VLR	0.97
P05783	KRT18	317	Keratin, type I cytoskeletal 18	147.2	NLK(1)ASLENSLR	1.01
P05783	KRT18	372	Keratin, type I cytoskeletal 18	132.01	VK(1)LEAEIATYR	1.04
P05783	KRT18	187	Keratin, type I cytoskeletal 18	116.05	K(1)VIDDTNITR	1.06
P05783	KRT18	247	Keratin, type I cytoskeletal 18	126.66	SQDLAK(1)IMADIR	1.06
P05783	KRT18	167	Keratin, type I cytoskeletal 18	131.22	VK(1)YETELAMR	1.07
Q86UP2	KTN1	678	Kinectin	96.89	MQQSVYVK(1)DDK	0.79
Q86UP2	KTN1	886	Kinectin	160.15	AVLEEK(1)EK	0.88
Q86UP2	KTN1	476	Kinectin	85.064	LQQEEVQK(1)K	0.98
Q86UP2	KTN1	872	Kinectin	117.89	GK(1)EEQMNTMK	1.01
Q86UP2	KTN1	1121	Kinectin	57.559	VLEHK(1)LK	1.08
Q86UP2	KTN1	653	Kinectin	55.228	AEVQK(1)LQALANEQAAAAHE	1.14
Q86UP2	KTN1	337	Kinectin	79.652	GELTTLIHQLQEK(1)DK	1.17
Q86UP2	KTN1	311	Kinectin	50.284	EK(1)SGVIQDALK	1.25
Q86UP2	KTN1	783	Kinectin	79.469	EVQDLK(1)AK	Tip60 OE only
P11279	LAMP1	137	Lysosome-associated membrane glycoprotein 1	73.781	EIK(1)TVESITDIR	Tip60 OE only
Q6PKG0	LARP1	964	La-related protein 1	69.812	YYSYGLEK(1)K	Tip60 OE only
Q9P2J5	LARS	647	Leucine--tRNA ligase, cytoplasmic	65.157	EAPFPK(1)TQIAK	1.07
Q9P2J5	LARS	486	Leucine--tRNA ligase, cytoplasmic	101.64	GQK(1)VQDVK	1.17
Q9P2J5	LARS	652	Leucine--tRNA ligase, cytoplasmic	41.025	TQIAK(1)EK	1.37
Q9P2J5	LARS	1047	Leucine--tRNA ligase, cytoplasmic	64.04	FASEAEDK(1)IR	Tip60 OE only
Q9P2J5	LARS	23	Leucine--tRNA ligase, cytoplasmic	99.815	EIQQK(1)WDTER	unquantifiable
Q14847	LASP1	87	LIM and SH3 domain protein 1	56.014	YK(1)EEFEK	1.14
Q14739	LBR	178	Lamin-B receptor	87.754	LK(1)EIDSK	unquantifiable
Q14739	LBR	190	Lamin-B receptor	71.501	YVAK(1)ELAVR	unquantifiable
P00338	LDHA	228	L-lactate dehydrogenase A chain	65.252	EQWK(1)EVHK	0.62
P00338	LDHA	222	L-lactate dehydrogenase A chain	107.24	TLHPDLGTDK(1)DK	0.76
P00338	LDHA	318	L-lactate dehydrogenase A chain	145.89	K(1)SADTLWGIQK	0.83
P00338	LDHA	224	L-lactate dehydrogenase A chain	132.84	TLHPDLGTDK(0.001)DK(0.9	0.93
P00338	LDHA	278	L-lactate dehydrogenase A chain	122.42	VHPVSTMIK(1)GLYGIK	0.97

P00338	LDHA	81	L-lactate dehydrogenase A chain	150.09	IVSGK(1)DYNVTANSK	0.98
P00338	LDHA	232	L-lactate dehydrogenase A chain	163.99	EVHK(1)QVVESAYEVIK	0.99
P00338	LDHA	76	L-lactate dehydrogenase A chain	78.192	TPK(1)IVSGK	1.04
P00338	LDHA	59	L-lactate dehydrogenase A chain	161.48	LK(1)GEMMDLQHGSLFLR	1.04
P00338	LDHA	5	L-lactate dehydrogenase A chain	180.67	ATLK(1)DQLIYNLLK	unquantifiable
P07195	LDHB	7	L-lactate dehydrogenase B chain	132.06	EK(1)LIAPVAEEEATVPNNK	0.75
P07195	LDHB	329	L-lactate dehydrogenase B chain	175.86	SADTLWDIQK(1)DLK	0.83
P07195	LDHB	308	L-lactate dehydrogenase B chain	315.73	GLTSVINQK(1)LKDDEVAQLK	0.88
P07195	LDHB	318	L-lactate dehydrogenase B chain	164.43	LKDDEVAQLK(1)K	0.88
P07195	LDHB	82	L-lactate dehydrogenase B chain	120.96	IVADK(1)DYSVTANSK	0.91
P07195	LDHB	310	L-lactate dehydrogenase B chain	140.45	LK(1)DDEVAQLK	0.98
P07195	LDHB	319	L-lactate dehydrogenase B chain	115.7	K(1)SADTLWDIQK	1
P07195	LDHB	156	L-lactate dehydrogenase B chain	105.39	LSGLPK(1)HR	1.02
P07195	LDHB	60	L-lactate dehydrogenase B chain	162.29	LK(1)GEMMDLQHGSLFLQTPI	1.03
P07195	LDHB	244	L-lactate dehydrogenase B chain	119.16	MVVESAYEVIK(1)LK	1.2
O95202	LETM1	463	LETM1 and EF-hand domain-containing protein 1	121.83	VAEVEGEQVDNK(1)AK	0.8
O95202	LETM1	597	LETM1 and EF-hand domain-containing protein 1	57.55	TGEEK(1)YVEESK	0.96
P18858	LIG1	422	DNA ligase 1	47.198	LTGSASTAK(1)K	2.44
P49257	LMAN1	288	Protein ERGIC-53	76.85	EK(1)YQEEFEHFQQELDK	0.79
P49257	LMAN1	87	Protein ERGIC-53	55.112	VAPSLK(1)SQR	0.97
P02545	LMNA	180	Prelamin-A/C;Lamin-A/C	63.216	LEAALGEAK(1)K	0.82
P02545	LMNA	201	Prelamin-A/C;Lamin-A/C	140.16	LQTMK(1)EELDFQK	0.95
P02545	LMNA	155	Prelamin-A/C;Lamin-A/C	166.34	EAALSTALSEK(1)R	1
P02545	LMNA	270	Prelamin-A/C;Lamin-A/C	96.948	TYSAK(1)LDNAR	1.05
P02545	LMNA	450	Prelamin-A/C;Lamin-A/C	99.522	VAVEEVDEEGK(1)FVR	1.08
P02545	LMNA	260	Prelamin-A/C;Lamin-A/C	91.937	AQHEDQVEQYK(1)K	1.15
P02545	LMNA	123	Prelamin-A/C;Lamin-A/C	127.4	K(1)EGDLIAAQAR	1.17
P02545	LMNA	208	Prelamin-A/C;Lamin-A/C	165.66	EELDFQK(1)NIYSEELR	1.44
P02545	LMNA	78	Prelamin-A/C;Lamin-A/C	79.639	EVSGIK(1)AAYEAELGDAR	Tip60 OE only
P20700	LMNA;LMNB1;LMNB	379	Prelamin-A/C;Lamin-A/C;Lamin-B1;Lamin-B2	63.419	K(1)LLEGEEER	unquantifiable
P20700	LMNB1	330	Lamin-B1	133.07	IQELEDLLAK(1)EK	0.91
P20700	LMNB1	271	Lamin-B1	53.828	EELEQTYHAK(1)LENAR	0.93
P20700	LMNB1	209	Lamin-B1	89.171	K(1)SMYEEEINETR	0.96
P20700	LMNB1	532	Lamin-B1	179.42	VILK(1)NSQGEEVAQR	0.96

P20700	LMNB1	123	Lamin-B1	176.8	AEHDQLLLNYAK(1)K	0.99
P20700	LMNB1	156	Lamin-B1	126.39	DAALATALGDK(1)K	1
P20700	LMNB1	145	Lamin-B1	173.44	LREYEAALNSK(1)DAALATALG	1.01
P20700	LMNB1	182	Lamin-B1	117.3	K(1)QLADETLLK	1.02
P20700	LMNB1	102	Lamin-B1	94.114	AK(1)LQIELGK	1.07
P20700	LMNB1	483	Lamin-B1	66.267	IGDTSVSYK(1)YTSR	1.08
P20700	LMNB1	261	Lamin-B1	111.12	LYK(1)EELEQTYHAK	1.08
P20700	LMNB1	109	Lamin-B1	72.485	LQIELGK(1)CK	1.15
P20700	LMNB1	342	Lamin-B1	121.29	MLTDK(1)ER	1.17
P20700	LMNB1	49	Lamin-B1	106.04	LAVYIDK(1)VR	Tip60 OE only
P20700	LMNB1;LMNB2	33	Lamin-B1;Lamin-B2	121.62	LQEK(1)EELR	1.07
Q03252	LMNB2	223	Lamin-B2	94.85	K(1)SVFEEEVR	0.81
Q03252	LMNB2	81	Lamin-B2	136.57	ISEK(1)EEVTTR	0.96
Q03252	LMNB2	186	Lamin-B2	63.185	AQLAK(1)AEDGHAVAK	1.01
Q03252	LMNB2	356	Lamin-B2	172.8	MLDAK(1)EQEMTEMR	1.02
Q03252	LMNB2	290	Lamin-B2	57.348	LDSAK(1)LSSDQNDK	1.1
Q03252	LMNB2	298	Lamin-B2	145.91	LSSDQNDK(1)AASAAR	1.11
P36776	LONP1	426	Lon protease homolog, mitochondrial	90.37	LK(1)ELVVPK	0.96
P36776	LONP1	374	Lon protease homolog, mitochondrial	82.426	EFELSK(1)LQQR	1.06
P36776	LONP1	529	Lon protease homolog, mitochondrial	74.326	ILCFYGPPGVGK(1)TSIAR	Tip60 OE only
P50851	LRBA	731	Lipopolysaccharide-responsive and beige-like an	58.433	LLASK(1)SEGIR	unquantifiable
P30533	LRPAP1	127	Alpha-2-macroglobulin receptor-associated prot	43.124	YGLDGK(1)K	unquantifiable
P42704	LRPPRC	1347	Leucine-rich PPR motif-containing protein, mito	108.47	ALYEHLTAK(1)NTK	0.66
P42704	LRPPRC	1332	Leucine-rich PPR motif-containing protein, mito	123.75	SYVSEK(1)DVTSAK	0.88
P42704	LRPPRC	868	Leucine-rich PPR motif-containing protein, mito	98.943	LVEK(1)GETDLIQK	0.92
P42704	LRPPRC	763	Leucine-rich PPR motif-containing protein, mito	194.17	HGK(1)LQDAINILK	0.95
P42704	LRPPRC	750	Leucine-rich PPR motif-containing protein, mito	174.84	LDSSAVLDTGK(1)YVGLVR	0.97
P42704	LRPPRC	777	Leucine-rich PPR motif-containing protein, mito	62.717	EK(1)DVLIK	1
P42704	LRPPRC	726	Leucine-rich PPR motif-containing protein, mito	167.6	HDK(1)VEDALNLK	1.02
P42704	LRPPRC	649	Leucine-rich PPR motif-containing protein, mito	118.48	DAHLLVESK(1)NLDFQK	1.03
P42704	LRPPRC	428	Leucine-rich PPR motif-containing protein, mito	43	ALMK(1)AVK	1.04
P42704	LRPPRC	395	Leucine-rich PPR motif-containing protein, mito	57.559	LTDYCK(1)K	1.04
P42704	LRPPRC	1338	Leucine-rich PPR motif-containing protein, mito	60.474	DVTSAK(1)ALYEHLTAK	1.09
P42704	LRPPRC	287	Leucine-rich PPR motif-containing protein, mito	129.84	GDIDHVK(1)QTLEK	1.17

P42704	LRPPRC	1357	Leucine-rich PPR motif-containing protein, mito	174.21	LDDLFLK(1)R	Tip60 OE only
P42704	LRPPRC	424	Leucine-rich PPR motif-containing protein, mito	50.607	TDLAK(1)ALMK	Tip60 OE only
P42704	LRPPRC;CFAP58	702	Leucine-rich PPR motif-containing protein, mito	73.039	ALELK(1)AK	0.97
Q8N1G4	LRRC47	369	Leucine-rich repeat-containing protein 47	49.715	LHEDLCEK(1)R	0.77
Q96AG4	LRRC59	73	Leucine-rich repeat-containing protein 59	202.2	NK(1)LQQLPADFGR	0.93
Q96AG4	LRRC59	135	Leucine-rich repeat-containing protein 59	138.11	VAGDCLDEK(1)QCK	1.04
Q96AG4	LRRC59	111	Leucine-rich repeat-containing protein 59	75.243	NLK(1)WLDLK	1.06
Q9Y333	LSM2	13	U6 snRNA-associated Sm-like protein LSm2	56.548	SLVGK(1)DVVVELK	unquantifiable
Q9UK45	LSM7	9	U6 snRNA-associated Sm-like protein LSm7	82.417	K(0.149)K(0.851)ESILDLSK	unquantifiable
Q9UK45	LSM7	26	U6 snRNA-associated Sm-like protein LSm7	103.28	VK(1)FQGGR	unquantifiable
P09960	LTA4H	337	Leukotriene A-4 hydrolase	107.74	LFGEK(1)FR	Tip60 OE only
P09960	LTA4H	225	Leukotriene A-4 hydrolase	90.05	TLVWSEK(1)EQVEK	Tip60 OE only
Q9NQ29	LUC7L	76	Putative RNA-binding protein Luc7-like 1	107.15	ADYEIASK(1)ER	unquantifiable
Q9Y383	LUC7L;LUC7L2	186	Putative RNA-binding protein Luc7-like 1;Putativ	94.616	NSMPASSFQQQK(1)LR	Tip60 OE only
Q9Y383	LUC7L2	61	Putative RNA-binding protein Luc7-like 2	70.98	MDLGECLK(1)VHDLALR	0.89
Q9Y383	LUC7L2	29	Putative RNA-binding protein Luc7-like 2	44.447	IK(1)FSDDR	1.05
Q9Y383	LUC7L2	224	Putative RNA-binding protein Luc7-like 2	60.59	EK(1)LEELKR	1.09
Q9Y383	LUC7L2	213	Putative RNA-binding protein Luc7-like 2	49.049	LADHFGGK(1)LHLGFIEIR	Tip60 OE only
Q9UNF1	MAGED2	494	Melanoma-associated antigen D2	112.75	AAAEAAAEAK(1)AR	Tip60 OE only
P61326	MAGOH;MAGOHB	61	Protein mago nashi homolog;Protein mago nashi	79.116	SVMEELK(1)R	unquantifiable
P55145	MANF	157	Mesencephalic astrocyte-derived neurotrophic fa	76.679	GCAEK(1)SDYIR	1.22
P36507	MAP2K2	40	Dual specificity mitogen-activated protein kinase	59.908	K(1)LEELELDEQQK	unquantifiable
P36507	MAP2K2	68	Dual specificity mitogen-activated protein kinase	41.399	VGELK(1)DDDFER	unquantifiable
P28482	MAPK1	344	Mitogen-activated protein kinase 1	81.865	LK(1)ELIFEETAR	0.97
Q15691	MAPRE1	83	Microtubule-associated protein RP/EB family me	111.28	ILQAGFK(1)R	1
Q15691	MAPRE1	212	Microtubule-associated protein RP/EB family me	147.51	LTVEDLEK(1)ER	Tip60 OE only
Q15691	MAPRE1	122	Microtubule-associated protein RP/EB family me	99.5	FFDANYDGK(1)DYDPVAAR	Tip60 OE only
Q15691	MAPRE1;MAPRE2	220	Microtubule-associated protein RP/EB family me	111.28	DFYFGK(1)LR	1.12
P56192	MARS	823	Methionine--tRNA ligase, cytoplasmic	42.395	FGGGQAK(1)TSPK	Tip60 OE only
P31153	MAT2A	234	S-adenosylmethionine synthase isoform type-2	83.453	AVVPAK(1)YLDEDTIYHLQPSG	Tip60 OE only
P43243	MATR3	532	Matrin-3	135.99	MK(1)SQAFIEMETR	1.07
P43243	MATR3	522	Matrin-3	75.854	LAEPYGK(1)IK	1.13
P43243	MATR3	524	Matrin-3	128.48	IK(1)NYILMR	1.17
P43243	MATR3	554	Matrin-3	92.538	EDAMAMVDHCLK(1)K	2.74

P43243	MATR3	491	Matrin-3	104.06	FDQK(1)QELGR	Tip60 OE only
P43243	MATR3	555	Matrin-3	73.637	K(1)ALWFQGR	Tip60 OE only
P49736	MCM2	469	DNA replication licensing factor MCM2	160.36	MITSLSK(1)DQQIGEK	0.74
P49736	MCM2	742	DNA replication licensing factor MCM2	133.57	LNQMDQDK(1)VAK	0.89
P49736	MCM2	224	DNA replication licensing factor MCM2	114.89	ISDMCK(1)ENR	1.17
P49736	MCM2	868	DNA replication licensing factor MCM2	44.692	DLVDK(1)AR	1.21
P49736	MCM2	745	DNA replication licensing factor MCM2	47.198	VAK(1)MYSDLR	1.27
P49736	MCM2	728	DNA replication licensing factor MCM2	102.72	YIIYAK(1)ER	1.32
P49736	MCM2	538	DNA replication licensing factor MCM2	72.485	YIEK(1)VSSR	1.38
P25205	MCM3	630	DNA replication licensing factor MCM3	75.738	LATAHAK(1)AR	1.13
P25205	MCM3	435	DNA replication licensing factor MCM3	76.679	VTIAK(1)AGIHAR	Tip60 OE only
P33991	MCM4	812	DNA replication licensing factor MCM4	60.788	LILSK(1)GK	0.8
P33991	MCM4	477	DNA replication licensing factor MCM4	61.962	LASALAPSIYEHEDIK(1)K	0.9
P33991	MCM4	746	DNA replication licensing factor MCM4	93.374	LAEAHAK(1)VR	0.95
P33991	MCM4	770	DNA replication licensing factor MCM4	41.724	EALK(1)QSATDPR	1.31
P33992	MCM5	141	DNA replication licensing factor MCM5	64.82	SLK(1)SDMMSHLVK	Tip60 OE only
Q14566	MCM6	744	DNA replication licensing factor MCM6	97.456	KVEEEEDESALK(1)R	0.76
Q14566	MCM6	25	DNA replication licensing factor MCM6	73.248	DEVAEK(1)CQK	0.92
Q14566	MCM6	599	DNA replication licensing factor MCM6	42.785	ESEDFIVEQYK(1)HLR	Tip60 OE only
P33993	MCM7	4	DNA replication licensing factor MCM7	106.6	ALK(1)DYALEK	1.01
P33993	MCM7	596	DNA replication licensing factor MCM7	119.45	EAWASK(1)DATYTSAR	1.16
P33993	MCM7	641	DNA replication licensing factor MCM7	100.72	LMEMSK(1)DSLLGDK	Tip60 OE only
Q9ULC4	MCTS1	18	Malignant T-cell-amplified sequence 1	92.925	ENVSNCIQLK(1)TSVIK	Tip60 OE only
P40925	MDH1	164	Malate dehydrogenase, cytoplasmic	89.123	AK(1)AQIALK	1.09
P40925	MDH1	110	Malate dehydrogenase, cytoplasmic	91.584	IFK(1)SQGAALDK	1.13
P40925	MDH1	248	Malate dehydrogenase, cytoplasmic	70.197	LSSAMSAAK(1)AICDHVR	1.21
P40925	MDH1	312	Malate dehydrogenase, cytoplasmic	66.073	EK(1)MDLTAK	1.24
P40925	MDH1	149	Malate dehydrogenase, cytoplasmic	90.653	SAPSIPK(1)ENFSCLTR	1.25
P40925	MDH1	318	Malate dehydrogenase, cytoplasmic	80.596	MDLTAK(1)ELTEEK	1.28
P40925	MDH1	236	Malate dehydrogenase, cytoplasmic	79.454	GAAVIK(1)AR	1.29
P40925	MDH1	73	Malate dehydrogenase, cytoplasmic	44.543	DVIATDK(1)EDVAFK	Tip60 OE only
P40925	MDH1	239	Malate dehydrogenase, cytoplasmic	70.908	K(1)LSSAMSAAK	unquantifiable
P40926	MDH2	78	Malate dehydrogenase, mitochondrial	113.99	AAVK(1)GYLGPEQLPDCLK	0.83
P40926	MDH2	329	Malate dehydrogenase, mitochondrial	60.682	K(1)GEDFVK	0.94

P40926	MDH2	241	Malate dehydrogenase, mitochondrial	172.99	AK(1)AGAGSATLSMAYAGAR	0.99
P40926	MDH2	239	Malate dehydrogenase, mitochondrial	60.342	IQEAGTEVVK(1)AK	0.99
P40926	MDH2	301	Malate dehydrogenase, mitochondrial	167.09	GIEK(1)NLGIGK	1.01
P40926	MDH2	307	Malate dehydrogenase, mitochondrial	97.214	NLGIGK(1)VSSFEEK	Tip60 OE only
P40926	MDH2	296	Malate dehydrogenase, mitochondrial	80.967	SQETECTYFSTPLLLGK(1)K	Tip60 OE only
P40926	MDH2	324	Malate dehydrogenase, mitochondrial	69.451	MISDAIPELK(1)ASIK	Tip60 OE only
P23368	ME2	224	NAD-dependent malic enzyme, mitochondrial	134.48	DPFYMGLYQK(1)R	1.37
P23368	ME2	340	NAD-dependent malic enzyme, mitochondrial	67.981	K(1)IWMFDK	Tip60 OE only
Q8N3F8	MICALL1	732	MICAL-like protein 1	60.518	LIHEK(1)HLLVR	unquantifiable
P52815	MRPL12	142	39S ribosomal protein L12, mitochondrial	100.48	LTEAK(0.003)PVDK(0.997)VI	0.8
P52815	MRPL12	162	39S ribosomal protein L12, mitochondrial	71.614	NYIQGINLVQAK(1)K	0.94
P52815	MRPL12	178	39S ribosomal protein L12, mitochondrial	73.985	ANVAK(1)AEAEK	0.96
P52815	MRPL12	173	39S ribosomal protein L12, mitochondrial	50.284	LVESLPQEIK(1)ANVAK	1.09
P52815	MRPL12	138	39S ribosomal protein L12, mitochondrial	91.069	LTEAK(1)PVDK	1.14
Q7Z2W9	MRPL21	151	39S ribosomal protein L21, mitochondrial	71.501	PLLGK(1)DLVR	unquantifiable
P82650	MRPS22	258	28S ribosomal protein S22, mitochondrial	47.067	GK(1)YDLLR	1.11
Q9Y3D9	MRPS23	102	28S ribosomal protein S23, mitochondrial	45.161	FVEK(1)YTELQK	Tip60 OE only
Q92552	MRPS27	274	28S ribosomal protein S27, mitochondrial	79.974	VAASPEDIK(1)LCR	unquantifiable
Q96E11	MRRF	223	Ribosome-recycling factor, mitochondrial	93.096	SK(1)DTVSEDTIR	1.54
Q9UKD2	MRTO4	69	mRNA turnover protein 4 homolog	54.525	MFFGK(1)NK	Tip60 OE only
P43246	MSH2	845	DNA mismatch repair protein Msh2	131.06	HVIECAK(1)QK	0.62
P52701	MSH6	1240	DNA mismatch repair protein Msh6	75.213	ELAETIK(1)CR	0.78
P52701	MSH6	852	DNA mismatch repair protein Msh6	95.573	AIMYEETTYSK(1)K	Tip60 OE only
P26038	MSN	79	Moesin	78.934	ESPLLFK(1)FR	0.79
P26038	MSN	501	Moesin	69.423	ADAMAK(1)DR	0.83
P26038	MSN	458	Moesin	143.89	AQMVQEDLEK(1)TR	0.84
P26038	MSN	514	Moesin	135.83	TTEAEK(1)NER	0.88
P26038	MSN	388	Moesin	74.464	AQSEAEK(1)LAK	0.9
P26038	MSN	165	Moesin	94.309	LNK(1)DQWEER	0.94
P26038	MSN	400	Moesin	53.554	ERQEAEEAK(1)EALLQASR	1.16
P26038	MSN	316	Moesin	40.103	HQK(1)QMER	1.31
P26038	MSN	352	Moesin	101.25	LK(1)QIEEQTK	unquantifiable
P26038	MSN;RDX	344	Moesin;Radixin	95.815	IEREK(1)EELMER	0.95
P11586	MTHFD1	832	C-1-tetrahydrofolate synthase, cytoplasmic;Meth	54.117	IIAQK(1)IYGADDIELLPEAQHK	0.73

P11586	MTHFD1	588	C-1-tetrahydrofolate synthase, cytoplasmic;Meth	70.1	LGK(1)MVVASSK	0.85
P11586	MTHFD1	333	C-1-tetrahydrofolate synthase, cytoplasmic;Meth	72.021	PIGK(1)LAR	1.07
P11586	MTHFD1	262	C-1-tetrahydrofolate synthase, cytoplasmic;Meth	92.866	VVGDVAYDEAK(1)ER	1.16
P11586	MTHFD1;MTHFD1L	684	C-1-tetrahydrofolate synthase, cytoplasmic;Meth	136.58	FFNIK(1)CR	1.04
Q9NVV4	MTPAP	205	Poly(A) RNA polymerase, mitochondrial	148.32	EFQLTEENTK(1)LR	0.77
P58546	MTPN	24	Myotrophin	99.815	DYVAK(1)GEDVNR	Tip60 OE only
P58546	MTPN	90	Myotrophin	45.161	LLLSK(1)GADK	unquantifiable
Q9BQG0	MYBBP1A	158	Myb-binding protein 1A	116.3	DQEALMK(1)SVK	1.03
P35580	MYH10	1809	Myosin-10	95.573	LQELEGAVK(1)SK	0.78
P35580	MYH10	1411	Myosin-10	88.681	LEEK(1)ALAYDK	0.8
P35580	MYH10	1800	Myosin-10	91.584	AK(1)LQELEGAVK	0.81
P35580	MYH10	1596	Myosin-10	80.377	LLIK(1)QVR	0.88
P35580	MYH10	1782	Myosin-10	59.245	SAAQK(1)SDNAR	0.89
P35580	MYH10	1520	Myosin-10	54.524	ADMEDLMSSK(1)DDVGK	0.98
P35580	MYH10	1281	Myosin-10	75.378	VELAEK(1)ASK	0.98
P35580	MYH10	1180	Myosin-10	47.198	EQEVAELK(1)K	1.29
P35580	MYH10	1399	Myosin-10	63.283	LLK(1)DAEALSQR	1.54
P35580	MYH10	1417	Myosin-10	41.448	ALAYDK(1)LEK	2.56
P35580	MYH10	1638	Myosin-10	84.17	DLEAQIEAANK(1)AR	Tip60 OE only
P35580	MYH10	1869	Myosin-10	57.59	HADQYK(1)EQMEK	Tip60 OE only
P35580	MYH10;MYH11	979	Myosin-10;Myosin-11	60.682	VTAEAK(1)IK	0.76
P35580	MYH10;MYH11	414	Myosin-10;Myosin-11	52.555	AQTK(1)EQADFAVEALAK	Tip60 OE only
	MYH10;MYH11;MYH14		Myosin-10;Myosin-11;Myosin-14	83.869	K(1)FDQLLAEEK	Tip60 OE only
Q7Z406	MYH14	1657	Myosin-14	73.781	AQMASAGQGK(1)EEAVK	unquantifiable
P35579	MYH9	1631	Myosin-9	84.352	DLEAHIDSANK(1)NR	0.93
P35579	MYH9	1392	Myosin-9	74.841	LQK(1)DLEGLSQR	0.95
P35579	MYH9	972	Myosin-9	60.682	VTTEAK(1)LK	1
P35579	MYH9	1404	Myosin-9	162.38	HEEK(1)VAAYDK	1.01
P35579	MYH9	1240	Myosin-9	50.484	VLLQGK(1)GDSEHK	1.04
P35579	MYH9	1918	Myosin-9	49.65	EVSSLK(1)NK	1.04
P35579	MYH9	1410	Myosin-9	67.897	VAAYDK(1)LEK	1.06
P35579	MYH9	1828	Myosin-9	206.58	IAQLEEQLDNETK(1)ER	1.09
P35579	MYH9	1724	Myosin-9	84.568	GALALEEK(1)R	1.09
P35579	MYH9	1793	Myosin-9	152.28	VK(1)LQEMEGTVK	1.1

P35579	MYH9	1332	Myosin-9	106.36	LK(1)QVEDEK	1.13
P35579	MYH9	1513	Myosin-9	127.52	TEMEDLMSSK(1)DDVGK	1.13
P35579	MYH9	1802	Myosin-9	125.48	LQEMEGTVK(1)SK	1.13
P35579	MYH9	1518	Myosin-9	125.23	DDVGK(1)SVHELEK	1.15
P35579	MYH9	860	Myosin-9	101.72	EK(1)QLAAENR	1.16
P35579	MYH9	1614	Myosin-9	79.469	K(1)LEMDLK	1.21
P35579	MYH9	1352	Myosin-9	157.38	EQLEEEEEAK(1)HNLEK	1.23
P35579	MYH9	1024	Myosin-9	67.115	NK(1)HEAMITDLEER	1.24
P35579	MYH9	1099	Myosin-9	148.96	VEEEAAQK(1)NMALK	1.27
P35579	MYH9	299	Myosin-9	143.7	TDLLLEPYNK(1)YR	1.31
P35579	MYH9	1862	Myosin-9	107.79	NAEQYK(1)DQADK	1.33
P35579	MYH9	1492	Myosin-9	153.95	ALEEAMEQK(1)AELER	1.42
P35579	MYH9	856	Myosin-9	94.407	EEELVK(1)VR	Tip60 OE only
P35579	MYH9	1454	Myosin-9	78.985	KFDQLLAEEK(1)TISAK	Tip60 OE only
P35579	MYH9	1445	Myosin-9	83.869	K(1)FDQLLAEEK	Tip60 OE only
P35579	MYH9	656	Myosin-9	72.006	EQLAK(1)LMATLR	Tip60 OE only
P35579	MYH9	1638	Myosin-9	70.912	DEAIK(1)QLR	Tip60 OE only
P35579	MYH9	1413	Myosin-9	72.006	VAAYDK(0.001)LEK(0.999)Tk	Tip60 OE only
P35579	MYH9	1648	Myosin-9	56.432	LQAQMK(1)DCMR	Tip60 OE only
P35579	MYH9;MYH10;MYH1	1477	Myosin-9;Myosin-10;Myosin-11	91.855	ETK(1)ALSLAR	1.22
P35579	MYH9;MYH10;MYH1	180	Myosin-9;Myosin-10;Myosin-11;Myosin-14	116.96	EDQSILCTGESGAGK(1)TENTK	1.38
P35579	MYH9;MYH10;MYH1	403	Myosin-9;Myosin-10;Myosin-14	126.52	DYVQK(1)AQTK	1.25
P35579	MYH9;MYH14	966	Myosin-9;Myosin-14	109.29	LQLEK(1)VTTEAK	1.09
P60660	MYL6	56	Myosin light polypeptide 6	95.81	VLGNPK(1)SDEMNVK	0.94
P60660	MYL6	81	Myosin light polypeptide 6	252.21	NK(1)DQGTYEDYVEGLR	1
O94832	MYO1D	524	Unconventional myosin-Id	46.001	NK(1)DTLFQDFK	unquantifiable
Q9UM54	MYO6	886	Unconventional myosin-VI	107.06	IK(1)STMMTQEQIQK	unquantifiable
Q9BSU3	NAA11;NAA10	148	N-alpha-acetyltransferase 11;N-alpha-acetyltrans	170.47	YYADGEDAYAMK(1)R	1.13
Q9BXJ9	NAA15	62	N-alpha-acetyltransferase 15, NatA auxiliary subu	97.635	K(1)EEAYELVR	0.88
Q9BXJ9	NAA15	419	N-alpha-acetyltransferase 15, NatA auxiliary subu	61.999	IYK(1)HAGNIK	0.91
Q9BXJ9	NAA15	454	N-alpha-acetyltransferase 15, NatA auxiliary subu	66.682	YMLK(1)ANLIK	1.86
Q9BXJ9	NAA15;NAA16	447	N-alpha-acetyltransferase 15, NatA auxiliary subu	58.917	FINSK(1)CAK	0.85
Q9BXJ9	NAA15;NAA16	467	N-alpha-acetyltransferase 15, NatA auxiliary subu	179.51	EAEEMCSK(1)FTR	1.08
E9PAV3	NACA	2005	Nascent polypeptide-associated complex subuni	168.76	IEDLSQQAQLAAAEK(1)FK	0.93

E9PAV3	NACA;NACAP1	1963	Nascent polypeptide-associated complex subuni	76.151	SK(1)NILFVITKPDVYK	0.88
Q4G0N4	NADK2	76	NAD kinase 2, mitochondrial	51.346	VVVVAK(1)TTR	unquantifiable
Q9NR45	NANS	52	Sialic acid synthase	61.679	ECGADCAK(1)FQK	0.79
Q9NR45	NANS	293	Sialic acid synthase	68.787	LGK(1)SVVAK	0.79
P55209	NAP1L1	82	Nucleosome assembly protein 1-like 1	79.713	VNALK(1)NLQVK	0.81
P55209	NAP1L1	87	Nucleosome assembly protein 1-like 1	151.83	NLQVK(1)CAQIEAK	0.94
P55209	NAP1L1	116	Nucleosome assembly protein 1-like 1	163.94	YAVLYQPLFDK(1)R	0.97
P55209	NAP1L1	105	Nucleosome assembly protein 1-like 1	138.71	K(1)YAVLYQPLFDK	0.98
Q99733	NAP1L4	192	Nucleosome assembly protein 1-like 4	93.267	HLQDIK(1)VK	0.92
Q99733	NAP1L4	94	Nucleosome assembly protein 1-like 4	125.74	K(1)YAALYQPLFDK	0.96
Q99733	NAP1L4	140	Nucleosome assembly protein 1-like 4	63.419	SK(1)VVVTEK	0.98
Q99733	NAP1L4	71	Nucleosome assembly protein 1-like 4	84.476	INALK(1)QLQVR	1.04
O43776	NARS	31	Asparagine--tRNA ligase, cytoplasmic	42.743	TGLK(1)ALMTVGK	Tip60 OE only
P49321	NASP	657	Nuclear autoantigenic sperm protein	163.99	IEDAK(1)ESQR	0.54
P49321	NASP	636	Nuclear autoantigenic sperm protein	69.954	EAEGSSAEYK(1)K	0.86
P49321	NASP	154	Nuclear autoantigenic sperm protein	69.451	EQVYDAMGEK(1)EEAK	0.87
P49321	NASP	44	Nuclear autoantigenic sperm protein	77.053	VESLDVDSEAK(1)K	0.89
P49321	NASP	626	Nuclear autoantigenic sperm protein	136.99	MAVLNEQVK(1)EAEGSSAEYK	0.98
P49321	NASP	45	Nuclear autoantigenic sperm protein	85.265	K(1)LLGLGQK	1.02
P49321	NASP	652	Nuclear autoantigenic sperm protein	50.632	EK(1)IEDAK	Tip60 OE only
P49321	NASP	405	Nuclear autoantigenic sperm protein	90.913	LTETK(1)DGSGLEEK	Tip60 OE only
P49321	NASP	450	Nuclear autoantigenic sperm protein	66.595	VAQGATEK(1)SPEDK	Tip60 OE only
Q9H0A0	NAT10	947	N-acetyltransferase 10	41.502	EFQEK(1)HK	0.65
Q9H0A0	NAT10	274	N-acetyltransferase 10	100.48	FIEGISEK(1)TLR	unquantifiable
Q15021	NCAPD2	395	Condensin complex subunit 1	90.108	IVQQK(1)ALPLTR	1.18
Q09161	NCBP1	698	Nuclear cap-binding protein subunit 1	126.19	LQEK(1)VESAQSEQK	1.08
Q09161	NCBP1	583	Nuclear cap-binding protein subunit 1	110.38	TLAESDEGK(1)LHVLR	1.18
Q9Y2A7	NCKAP1	905	Nck-associated protein 1	74.464	LSSVDSVLK(1)R	1.08
P19338	NCL	627	Nucleolin	138.94	AAK(1)EAMEDGEIDGNK	0.78
P19338	NCL	467	Nucleolin	53.528	SISLYYTGEK(1)GQNQDYR	0.79
P19338	NCL	348	Nucleolin	111.79	K(1)FGYVDFESAEDLEK	0.84
P19338	NCL	610	Nucleolin	97.797	ETGSSK(1)GFGFVDFNSEEDAK	0.85
P19338	NCL	624	Nucleolin	130.01	GFGFVDFNSEEDAK(1)AAK	0.86
P19338	NCL	577	Nucleolin	185.59	TLFVK(1)GLSEDTTEETLK	0.89

P19338	NCL	324	Nucleolin	155.99	SAPELK(1)TGISDVFAK	0.89
P19338	NCL	545	Nucleolin	124.08	EALNSCNK(1)R	0.92
P19338	NCL	377	Nucleolin	84.17	VFGNEIK(0.999)LEK(0.001)P	0.93
P19338	NCL	639	Nucleolin	106.13	EAMEDGEIDGNK(1)VTLDWA	0.94
P19338	NCL	429	Nucleolin	67.334	SK(1)GIAYIEFK	0.95
P19338	NCL	444	Nucleolin	175.41	TEADAEK(1)TFEEK	0.96
P19338	NCL	403	Nucleolin	127.76	NLPYK(1)VTQDELK	0.96
P19338	NCL	294	Nucleolin	64.776	AAPEAK(1)K	0.97
P19338	NCL	87	Nucleolin	50.127	AAVTPGK(1)K	0.98
P19338	NCL	477	Nucleolin	67.113	GGK(1)NSTWSGESK	0.98
P19338	NCL	449	Nucleolin	145.28	TFEEK(1)QGTEIDGR	1.01
P19338	NCL	398	Nucleolin	101.6	TLLAK(1)NLPYK	1.04
P19338	NCL	572	Nucleolin	54.722	SQPSK(1)TLFVK	1.07
P19338	NCL	223	Nucleolin	79.116	AAK(1)VVPVK	1.1
P19338	NCL	102	Nucleolin	101.93	TVTPAK(1)AVTTPGK	1.22
Q92597	NDRG1	280	Protein NDRG1	83.783	LDPTK(1)TTLLK	0.76
Q15843	NEDD8	48	NEDD8	111.06	LIYSGK(1)QMNDEK	unquantifiable
Q15843	NEDD8	6	NEDD8	64.567	VK(1)TLTGK	unquantifiable
P07196	NEFL	157	Neurofilament light polypeptide	236.61	LAAEDATNEK(1)QALQGER	unquantifiable
P55769	NHP2L1	86	NHP2-like protein 1;NHP2-like protein 1, N-term	81.381	SK(1)QALGR	0.66
Q9BYG3	NIFK	158	MKI67 FHA domain-interacting nucleolar phospl	103.29	TLTQK(1)LR	0.9
P15531	NME1	49	Nucleoside diphosphate kinase A	129.71	FMQASEDLLK(1)EHYVDLK	0.98
P15531	NME1	56	Nucleoside diphosphate kinase A	86.641	EHYVDLK(1)DRPFFAGLVK	1.02
P15531	NME1	39	Nucleoside diphosphate kinase A	98.353	LVGLK(1)FMQASEDLLK	1.04
P22392	NME1;NME2	12	Nucleoside diphosphate kinase A;Nucleoside dipl	185.6	TFIAIK(1)PDGVQR	0.96
P22392	NME1;NME2	26	Nucleoside diphosphate kinase A;Nucleoside dipl	120.62	GLVGEIIK(1)R	0.97
P22392	NME1;NME2;NME2P	100	Nucleoside diphosphate kinase A;Nucleoside dipl	191.26	VMLGETNPADSK(1)PGTIR	0.97
P22392	NME1;NME2;NME2P	31	Nucleoside diphosphate kinase A;Nucleoside dipl	125.86	FEQK(1)GFR	1.02
P22392	NME2	39	Nucleoside diphosphate kinase B	129.16	LVAMK(1)FLR	1.04
P22392	NME2;NME2P1	124	Nucleoside diphosphate kinase B;Putative nuclec	138.75	NIIHGSDSVK(1)SAEK	1.02
P22392	NME2;NME2P1	49	Nucleoside diphosphate kinase B;Putative nuclec	86.497	ASEEHLK(1)QHYIDLK	1.03
P30419	NMT1;NMT2	464	Glycylpeptide N-tetradecanoyltransferase 1;Glyc	49.358	TFLEK(1)LK	0.88
Q13423	NNT	1079	NAD(P) transhydrogenase, mitochondrial	161.02	TCDALQAK(1)VR	1.15
Q9Y3T9	NOC2L	226	Nucleolar complex protein 2 homolog	47.743	LLFGK(1)VAK	1.12

Q9Y3T9	NOC2L	516	Nucleolar complex protein 2 homolog	79.693	LSNVNLQEK(1)AYR	Tip60 OE only
Q15233	NONO	243	Non-POU domain-containing octamer-binding pı	114.78	LVIK(1)NQQFHK	0.91
Q15233	NONO	272	Non-POU domain-containing octamer-binding pı	110.87	WK(1)ALIEMEK	0.95
Q15233	NONO	279	Non-POU domain-containing octamer-binding pı	236.57	ALIEMEK(1)QQQDQVDR	0.99
Q15233	NONO	295	Non-POU domain-containing octamer-binding pı	111.06	EK(1)LEMEMEAAR	0.99
Q15233	NONO	336	Non-POU domain-containing octamer-binding pı	113.61	MEELHNQEVQK(1)R	1.02
Q15233	NONO	249	Non-POU domain-containing octamer-binding pı	96.253	NQQFHK(1)ER	1.04
Q15233	NONO	99	Non-POU domain-containing octamer-binding pı	56.225	YGK(1)AGEVFIHK	1.04
Q15233	NONO	107	Non-POU domain-containing octamer-binding pı	42.243	AGEVFIHK(1)DK	1.09
Q15233	NONO	109	Non-POU domain-containing octamer-binding pı	110.63	DK(1)GFGFIR	1.1
Q15233	NONO	68	Non-POU domain-containing octamer-binding pı	82.287	KPGEK(1)TFTQR	1.28
Q15233	NONO	96	Non-POU domain-containing octamer-binding pı	59.205	LFEK(1)YGK	1.52
Q15233	NONO	198	Non-POU domain-containing octamer-binding pı	120.12	GIVEFSGK(1)PAAR	unquantifiable
O00567	NOP56	240	Nucleolar protein 56	114.4	LEELTMDGAK(1)AK	0.92
O00567	NOP56	242	Nucleolar protein 56	66.692	AK(1)AILDASR	1.03
O00567	NOP56	375	Nucleolar protein 56	70.552	YLANK(1)CSIASR	1.17
O00567	NOP56	143	Nucleolar protein 56	97.271	GLTDLSACK(1)AQLGLGHSYSI	1.23
Q9Y2X3	NOP58	93	Nucleolar protein 58	91.657	EAHEPLAVADAK(1)LGGVIK	0.89
Q9Y2X3	NOP58	365	Nucleolar protein 58	78.934	MLAAK(1)TVLAIR	0.9
Q9Y2X3	NOP58	323	Nucleolar protein 58	124.2	HAASTVQILGAEK(1)ALFR	1.05
P55786	NPEPPS	869	Puromycin-sensitive aminopeptidase	42.64	MAGEVK(1)AFFESHPAPSAER	0.8
P55786	NPEPPS	768	Puromycin-sensitive aminopeptidase	49.418	LHK(1)QADMQEEK	1.04
P55786	NPEPPS	712	Puromycin-sensitive aminopeptidase	68.915	GLVLGK(1)LGK	1.05
P55786	NPEPPS;NPEPPSL1	274	Puromycin-sensitive aminopeptidase;Puromycir	107.79	VYTPVGK(1)AEQGK	0.66
P55786	NPEPPS;NPEPPSL1	279	Puromycin-sensitive aminopeptidase;Puromycir	57.019	AEQGK(1)FALEVAAK	Tip60 OE only
P06748	NPM1	141	Nucleophosmin	114.78	LLSISGK(1)R	0.74
P06748	NPM1	202	Nucleophosmin	87.284	DTPAK(1)NAQK	0.79
P06748	NPM1	223	Nucleophosmin	74.127	SK(1)GQESFK	0.84
P06748	NPM1	257	Nucleophosmin	80.979	MQASIEK(1)GGSLPK	0.89
P06748	NPM1	248	Nucleophosmin	147.51	GPSSVEDIK(1)AK	0.91
P06748	NPM1	267	Nucleophosmin	130.22	VEAK(1)FINYVK	0.94
P06748	NPM1	250	Nucleophosmin	104.44	AK(1)MQASIEK	0.96
P06748	NPM1	27	Nucleophosmin	95.909	ADK(1)DYHFK	0.99
P06748	NPM1	150	Nucleophosmin	77.662	SAPGGGSK(1)VPQK	1

P06748	NPM1	273	Nucleophosmin	139.68	FINYVK(1)NCFR	1.01
P06748	NPM1	32	Nucleophosmin	146.19	DYHFK(1)VDNDENEHQLSLR	1.09
P06748	NPM1	229	Nucleophosmin	67.035	GQESFK(1)K	Tip60 OE only
Q08J23	NSUN2	84	tRNA (cytosine(34)-C(5))-methyltransferase	48.283	ITGYK(1)SHAK	0.76
Q08J23	NSUN2	445	tRNA (cytosine(34)-C(5))-methyltransferase	109.71	LQGK(1)SAETR	1.09
Q08J23	NSUN2	710	tRNA (cytosine(34)-C(5))-methyltransferase	87.258	MMGLEVLGEK(1)K	unquantifiable
P80303	NUCB2	121	Nucleobindin-2;Nesfatin-1	45.221	AK(1)LDSLQDIGMDHQALLK	0.59
Q9Y266	NUDC	196	Nuclear migration protein nudC	77.677	GK(1)DMVVDIQR	0.63
Q9Y266	NUDC	39	Nuclear migration protein nudC	102.87	K(1)TDFFIGGEEGMAEK	0.83
Q9Y266	NUDC	96	Nuclear migration protein nudC	70.363	EAK(1)SETSGPQIK	0.88
Q9Y266	NUDC	122	Nuclear migration protein nudC	69.423	LQLEIDQK(1)K	0.97
Q9Y266	NUDC	247	Nuclear migration protein nudC	47.288	VVTVHLEK(1)INK	1.01
Q9Y266	NUDC	267	Nuclear migration protein nudC	62.303	LVSSDPEINTK(1)K	Tip60 OE only
O43809	NUDT21	122	Cleavage and polyadenylation specificity factor s	160.69	LPGGELNPGEDEVEGLK(1)R	0.85
Q14980	NUMA1	373	Nuclear mitotic apparatus protein 1	104.2	ELSAALQDK(1)K	0.51
Q14980	NUMA1	1210	Nuclear mitotic apparatus protein 1	96.331	AEDEWK(1)AQVAR	0.72
Q14980	NUMA1	908	Nuclear mitotic apparatus protein 1	75.102	LADDLSTLQEK(1)MAATSK	0.75
Q14980	NUMA1	1699	Nuclear mitotic apparatus protein 1	78.653	DLGK(1)FQVATDALK	0.78
Q14980	NUMA1	914	Nuclear mitotic apparatus protein 1	109.71	MAATSK(1)EVAR	0.82
Q14980	NUMA1	604	Nuclear mitotic apparatus protein 1	87.568	DAALK(1)QLEALEK	0.91
Q14980	NUMA1	358	Nuclear mitotic apparatus protein 1	93.959	ATQEWLEK(1)QAQLEK	0.92
Q14980	NUMA1	561	Nuclear mitotic apparatus protein 1	62.303	HQVEQLSSSLK(1)QK	1.08
Q14980	NUMA1	891	Nuclear mitotic apparatus protein 1	230.1	ALQQVQEK(1)EVR	1.11
Q14980	NUMA1	722	Nuclear mitotic apparatus protein 1	51.854	GSLEEEK(1)R	1.31
Q14980	NUMA1	1131	Nuclear mitotic apparatus protein 1	90.15	AEVSK(1)LEQQCQK	1.68
Q14980	NUMA1	1062	Nuclear mitotic apparatus protein 1	71.379	EGK(1)DQELAK	Tip60 OE only
Q14980	NUMA1	1566	Nuclear mitotic apparatus protein 1	79.148	LADSDQASK(1)VQQQK	Tip60 OE only
Q92621	NUP205	1978	Nuclear pore complex protein Nup205	96.89	K(1)LLDIEGLYSK	0.89
Q92621	NUP205	1742	Nuclear pore complex protein Nup205	72.652	FQDDNVEGDK(1)VSK	0.91
Q9BW27	NUP85	494	Nuclear pore complex protein Nup85	52.693	AK(1)DAAFATLVSDR	Tip60 OE only
Q9BW27	NUP85	540	Nuclear pore complex protein Nup85	69.176	LTFLGK(1)YR	unquantifiable
Q8N1F7	NUP93	619	Nuclear pore complex protein Nup93	94.191	VASVAENK(1)GLFEEAAK	0.87
Q9BVL2	NUPL1	414	Nucleoporin p58/p45	103.56	VLK(1)EQYLGYR	unquantifiable
Q9BVL2	NUPL1	312	Nucleoporin p58/p45	113.71	LK(1)IETAQELK	unquantifiable

P04181	OAT	155	Ornithine aminotransferase, mitochondrial;Orni	58.782	K(1)WGYTVK	0.68
P04181	OAT	421	Ornithine aminotransferase, mitochondrial;Orni	92.866	FAPPLVIK(1)EDELR	0.89
P04181	OAT	374	Ornithine aminotransferase, mitochondrial;Orni	76.605	GK(1)GLLNAIVIK	0.94
P04181	OAT	386	Ornithine aminotransferase, mitochondrial;Orni	52.482	ETK(1)DWDAWK	0.99
P04181	OAT	107	Ornithine aminotransferase, mitochondrial;Orni	114.89	SQVDK(1)LTLTSR	1.02
P04181	OAT	362	Ornithine aminotransferase, mitochondrial;Orni	93.345	NELMK(1)LPSDVVTAVR	1.42
P04181	OAT	66	Ornithine aminotransferase, mitochondrial;Orni	96.171	GK(1)GIYLWDVEGR	1.85
Q9NTK5	OLA1	190	Obg-like ATPase 1	105.52	VK(1)SWVIDQK	1.01
Q9NTK5	OLA1	197	Obg-like ATPase 1	46.844	SWVIDQK(0.836)K(0.164)PV	unquantifiable
Q8NGZ4	OR2G3	229	Olfactory receptor 2G3	40.268	IK(1)SVEAR	unquantifiable
Q9P0S3	ORMDL1;ORMDL3;O	79	ORM1-like protein 1;ORM1-like protein 3;ORM1-	56.359	GTPFETPDQGK(1)AR	unquantifiable
Q96FW1	OTUB1	71	Ubiquitin thioesterase OTUB1	194.06	EYAEDDNIYQQK(1)IK	0.84
Q96FW1	OTUB1	115	Ubiquitin thioesterase OTUB1	67.032	FK(1)AVSAK	0.85
Q96FW1	OTUB1	201	Ubiquitin thioesterase OTUB1	138.45	TVK(1)EFCQQEVEPMCK	0.88
P55809	OXCT1	473	Succinyl-CoA:3-ketoacid coenzyme A transferase	93.058	IITEK(1)AVFDVDK	1.24
P07237	P4HB	436	Protein disulfide-isomerase	50.358	MDSTANEVEAVK(1)VHSFPTLI	0.89
P07237	P4HB	276	Protein disulfide-isomerase	98.737	LSNFK(1)TAAESFK	0.92
P07237	P4HB	328	Protein disulfide-isomerase	109.42	YK(1)PESEELTAER	0.93
P07237	P4HB	81	Protein disulfide-isomerase	158.2	LAK(1)VDATEESDLAQQYGVR	0.94
P07237	P4HB	326	Protein disulfide-isomerase	148.88	LITLEEEMTK(0.999)YK(0.001	0.96
P07237	P4HB	283	Protein disulfide-isomerase	71.692	TAAESFK(1)GK	0.97
P07237	P4HB	130	Protein disulfide-isomerase	86.136	EADDIVNWLK(1)K	0.99
P07237	P4HB	71	Protein disulfide-isomerase	84.743	LK(1)AEGSEIR	0.99
P07237	P4HB	385	Protein disulfide-isomerase	113.1	NFEDVAFDEK(1)K	1
P07237	P4HB	31	Protein disulfide-isomerase	95.273	K(1)SNFAEALAAHK	1.02
P07237	P4HB	65	Protein disulfide-isomerase	90.15	ALAPEYAK(1)AAGK	1.02
P07237	P4HB	271	Protein disulfide-isomerase	114.89	SVSDYDGK(1)LSNFK	1.02
P07237	P4HB	103	Protein disulfide-isomerase	105.95	GYPTIK(1)FFR	1.09
P07237	P4HB	444	Protein disulfide-isomerase	95.067	VHSFPTLK(1)FFPASADR	1.3
P07237	P4HB	375	Protein disulfide-isomerase	87.363	VLVGK(1)NFEDVAFDEK	Tip60 OE only
P07237	P4HB	467	Protein disulfide-isomerase	46.37	TLDGFK(1)K	unquantifiable
Q9UQ80	PA2G4	210	Proliferation-associated protein 2G4	166.19	TIIQNPTDQQK(1)K	0.89
Q9UQ80	PA2G4	22	Proliferation-associated protein 2G4	131.06	YK(1)MGGDIANR	0.89
Q9UQ80	PA2G4	287	Proliferation-associated protein 2G4	70.912	AFEDEK(1)K	0.96

Q9UQ80	PA2G4	144	Proliferation-associated protein 2G4	97.32	ADVIK(1)AAHLCAEAALR	1.17
Q9UQ80	PA2G4	355	Proliferation-associated protein 2G4	169.04	SEMEVQDAELK(1)ALLQSSASF	1.24
Q9UQ80	PA2G4	101	Proliferation-associated protein 2G4	88.734	SDQDYILK(1)EGDLVK	Tip60 OE only
Q9UQ80	PA2G4	253	Proliferation-associated protein 2G4	96.253	DPSK(1)QYGLK	Tip60 OE only
P11940	PABPC1	188	Polyadenylate-binding protein 1	99.139	AK(1)EFTNVYIK	1.01
P11940	PABPC1;PABPC3	361	Polyadenylate-binding protein 1;Polyadenylate-l	87.298	IVATK(1)PLYVALAQR	1.02
P11940	PABPC1;PABPC3	229	Polyadenylate-binding protein 1;Polyadenylate-l	120.12	VMTDESGK(1)SK	1.03
P11940	PABPC1;PABPC3	221	Polyadenylate-binding protein 1;Polyadenylate-l	96.745	FGPALSVK(1)VMTDESGK	1.03
P11940	PABPC1;PABPC3;PAE	284	Polyadenylate-binding protein 1;Polyadenylate-l	148.38	FEQMK(1)QDR	1.13
P11940	PABPC1;PABPC3;PAE	78	Polyadenylate-binding protein 1;Polyadenylate-l	139.39	ALDTMNFDVIK(1)GK	0.92
Q13310	PABPC4	284	Polyadenylate-binding protein 4	58.23	FEQLK(1)QER	1.13
Q13310	PABPC4	221	Polyadenylate-binding protein 4	81.784	TLSVK(1)VMR	1.24
Q13310	PABPC4	188	Polyadenylate-binding protein 4	99.139	AK(1)EFTNVYIK	1.25
Q13310	PABPC4	186	Polyadenylate-binding protein 4	95.531	EAELGAK(1)AK	1.27
Q13310	PABPC4;PABPC4L	309	Polyadenylate-binding protein 4;Polyadenylate-l	97.456	NLDDTIDDEK(1)LR	1.23
Q9UKS6	PACSIN3	203	Protein kinase C and casein kinase substrate in ne	41.743	TK(1)AQYEQTLAELHR	Tip60 OE only
P22234	PAICS	17	Multifunctional protein ADE2;Phosphoribosylan	82.279	LYEGK(1)TK	0.7
P22234	PAICS	19	Multifunctional protein ADE2;Phosphoribosylan	99.215	TK(1)EVYELLDSPGK	0.8
P22234	PAICS	235	Multifunctional protein ADE2;Phosphoribosylan	87.713	DLK(1)EVTPEGLQMVK	0.9
P22234	PAICS	36	Multifunctional protein ADE2;Phosphoribosylan	221.55	VLLQSK(1)DQITAGNAAR	0.98
P22234	PAICS	53	Multifunctional protein ADE2;Phosphoribosylan	100.55	NHLEGK(1)AAISNK	1.01
P22234	PAICS	286	Multifunctional protein ADE2;Phosphoribosylan	76.927	K(1)ACGNFGIPCELR	1.08
P22234	PAICS	110	Multifunctional protein ADE2;Phosphoribosylan	81.548	IATGSFLK(1)R	1.3
Q9H074	PAIP1	335	Polyadenylate-binding protein-interacting prote	110.86	LTGSVLEDAWK(1)EK	0.88
Q9H074	PAIP1	258	Polyadenylate-binding protein-interacting prote	43.512	DQAAK(1)GDEVTR	1.07
Q9H074	PAIP1	253	Polyadenylate-binding protein-interacting prote	118.18	TEYEVK(1)DQAAK	Tip60 OE only
Q13177	PAK2	235	Serine/threonine-protein kinase PAK 2;PAK-2p27	85.377	MTDEEIMEK(1)LR	1.92
Q99497	PARK7	182	Protein deglycase DJ-1	116.74	EVAAQVK(1)APLVLK	0.85
Q99497	PARK7	62	Protein deglycase DJ-1	129.84	DVVICPDASLEDAK(1)K	1.04
Q99497	PARK7	32	Protein deglycase DJ-1	56.404	AGIK(1)VTVAGLAGK	1.09
Q99497	PARK7	130	Protein deglycase DJ-1	53.237	VTTHPLAK(1)DK	1.11
Q99497	PARK7	41	Protein deglycase DJ-1	136.27	VTVAGLAGK(1)DPVQCSR	1.13
Q99497	PARK7	148	Protein deglycase DJ-1	83.499	VEK(1)DGLILTSR	Tip60 OE only
O95453	PARN	326	Poly(A)-specific ribonuclease PARN	67.022	LLDTK(1)LMASTQPFK	unquantifiable

P09874	PARP1	433	Poly [ADP-ribose] polymerase 1	60.828	ASLCISTK(1)K	0.69
P09874	PARP1	683	Poly [ADP-ribose] polymerase 1	83.005	MIFDVESMK(1)K	0.71
P09874	PARP1	662	Poly [ADP-ribose] polymerase 1	81.972	LTVNPGTK(1)SK	0.81
P09874	PARP1	447	Poly [ADP-ribose] polymerase 1	157.91	MEEVK(1)EANIR	0.89
P09874	PARP1	512	Poly [ADP-ribose] polymerase 1	57.859	GQVK(1)EEGINK	1.01
P09874	PARP1	400	Poly [ADP-ribose] polymerase 1	111.95	ILTLGK(1)LSR	1.02
P09874	PARP1	518	Poly [ADP-ribose] polymerase 1	60.436	EEGINK(1)SEK	1.15
P09874	PARP1	579	Poly [ADP-ribose] polymerase 1	80.688	LQLLEDDK(1)ENR	1.35
P09874	PARP1	607	Poly [ADP-ribose] polymerase 1	47.548	LEQMPSK(1)EDAIEHFMK	1.82
P09874	PARP1	418	Poly [ADP-ribose] polymerase 1	59.908	LGGK(1)LTGTANK	Tip60 OE only
P09874	PARP1	15	Poly [ADP-ribose] polymerase 1	51.346	VEYAK(1)SGR	Tip60 OE only
P09874	PARP1	134	Poly [ADP-ribose] polymerase 1	67.344	IEK(1)GQVR	Tip60 OE only
P09874	PARP1	425	Poly [ADP-ribose] polymerase 1	113.65	LTGTANK(1)ASLCISTK	unquantifiable
Q15365	PCBP1	115	Poly(rC)-binding protein 1	112.43	LVVPATQCGSLIGK(1)GGCK	0.89
Q15366	PCBP1;PCBP3;PCBP2	23	Poly(rC)-binding protein 1;Poly(rC)-binding prot	176.51	LLMHGK(1)EVGSIIGK	0.92
Q15366	PCBP1;PCBP3;PCBP4	31	Poly(rC)-binding protein 1;Poly(rC)-binding prot	50.127	EVGSIIGK(1)K	1.13
Q15366	PCBP2	115	Poly(rC)-binding protein 2	152.14	LVVPASQCGSLIGK(1)GGCK	1.21
P22061	PCMT1	4	Protein-L-isoaspartate(D-aspartate) O-methyltrar	83.418	AWK(1)SGGASHSELIHNLR	0.99
P22061	PCMT1	105	Protein-L-isoaspartate(D-aspartate) O-methyltrar	46.89	MVGCTGK(1)VIGIDHIK	1.42
P12004	PCNA	248	Proliferating cell nuclear antigen	51.092	IADMGHLK(1)YYLAPK	0.93
P12004	PCNA	13	Proliferating cell nuclear antigen	83.265	LVQGSILK(1)K	0.99
P12004	PCNA	164	Proliferating cell nuclear antigen	59.281	DLSHIGDAVVISCAK(1)DGVK	1.11
P12004	PCNA	80	Proliferating cell nuclear antigen	102.4	ILK(1)CAGNEDIITLR	1.31
Q9UHG3	PCYOX1	415	Prenylcysteine oxidase 1	99.283	IFSQETLTK(1)AQILK	1.05
Q9UHG3	PCYOX1	205	Prenylcysteine oxidase 1	149.96	TLLETLQK(1)AGFSEK	Tip60 OE only
Q13442	PDAP1	137	28 kDa heat- and acid-stable phosphoprotein	102.07	TEQAK(1)ADLAR	1.16
Q13442	PDAP1	126	28 kDa heat- and acid-stable phosphoprotein	40.724	YMK(1)MHLAGK	1.32
Q13442	PDAP1	132	28 kDa heat- and acid-stable phosphoprotein	47.228	MHLAGK(1)TEQAK	Tip60 OE only
Q14690	PDCD11	790	Protein RRP5 homolog	102.61	VTNVDEEK(1)QR	0.97
Q53EL6	PDCD4	402	Programmed cell death protein 4	64.297	SSTITVDQMK(1)R	0.74
O14737	PDCD5	105	Programmed cell death protein 5	50.284	VSQQTEK(1)TTTVK	1.55
O14737	PDCD5	20	Programmed cell death protein 5	48.229	LAELQAK(1)HGDPGDAAQQE	Tip60 OE only
O75340	PDCD6	112	Programmed cell death protein 6	82.417	DNSGMIDK(1)NELK	unquantifiable
Q8WUM4	PDCD6IP	614	Programmed cell death 6-interacting protein	89.403	VYGGLTTK(1)VQESLK	0.79

Q8WUM4	PDCD6IP	303	Programmed cell death 6-interacting protein	54.549	DFSDK(1)INR	0.99
Q8WUM4	PDCD6IP	690	Programmed cell death 6-interacting protein	83.883	FQNK(1)CSDIVFAR	Tip60 OE only
P08559	PDHA1	63	Pyruvate dehydrogenase E1 component subunit	58.917	EDGLK(1)YYR	Tip60 OE only
P11177	PDHB	227	Pyruvate dehydrogenase E1 component subunit	50.607	DFLIPIGK(1)AK	0.95
P11177	PDHB	188	Pyruvate dehydrogenase E1 component subunit	124.89	GLIK(1)SAIR	1.07
O00330	PDHX	120	Pyruvate dehydrogenase protein X component,	95.358	IVVEEGSK(1)NIR	unquantifiable
P30101	PDIA3	129	Protein disulfide-isomerase A3	67.113	TADGIVSHLK(1)K	0.59
P30101	PDIA3	146	Protein disulfide-isomerase A3	72.234	TEEEFK(1)K	0.87
P30101	PDIA3	415	Protein disulfide-isomerase A3	80.462	NLEPK(1)YK	0.93
P30101	PDIA3	231	Protein disulfide-isomerase A3	57.811	MTSGK(1)IK	0.97
P30101	PDIA3	226	Protein disulfide-isomerase A3	146.11	TVAYTEQK(1)MTSGK	1
P30101	PDIA3	425	Protein disulfide-isomerase A3	56.043	LSK(1)DPNIVIAK	1.01
P30101	PDIA3	274	Protein disulfide-isomerase A3	129.47	NAK(1)GSNYWR	1.03
P30101	PDIA3	218	Protein disulfide-isomerase A3	164.64	FEDK(1)TVAYTEQK	1.05
P30101	PDIA3	296	Protein disulfide-isomerase A3	132.47	FLDAGHK(1)LNFAVASR	1.05
P30101	PDIA3	366	Protein disulfide-isomerase A3	97.813	YLK(1)SEPIPESNDGPVK	1.15
P30101	PDIA3	417	Protein disulfide-isomerase A3	70.69	YK(1)ELGEK	1.26
P30101	PDIA3	288	Protein disulfide-isomerase A3	56.51	VMMVAK(1)K	1.51
P30101	PDIA3	214	Protein disulfide-isomerase A3	111.63	PSHLTNK(1)FEDK	Tip60 OE only
P30101	PDIA3	335	Protein disulfide-isomerase A3	42.976	GEK(1)FVMQEEFSR	Tip60 OE only
P13667	PDIA4	218	Protein disulfide-isomerase A4	51.927	KLAPEYEK(1)AAK	0.86
P13667	PDIA4	145	Protein disulfide-isomerase A4	84.17	K(1)GQAVDYEGSR	1.06
P13667	PDIA4	637	Protein disulfide-isomerase A4	59.067	FIEEHATK(1)LSR	Tip60 OE only
Q15084	PDIA6	245	Protein disulfide-isomerase A6	166.14	IFQK(1)GESPVDYDGGR	0.96
Q15084	PDIA6	208	Protein disulfide-isomerase A6	103.21	NLEPEWAAAASEVK(1)EQTK	1.04
Q15084	PDIA6	150	Protein disulfide-isomerase A6	54.416	SGGYSSGK(1)QGR	1.07
Q15084	PDIA6	160	Protein disulfide-isomerase A6	54.259	K(1)DVIELTDDSFDK	Tip60 OE only
O00151	PDLIM1	22	PDZ and LIM domain protein 1	102.62	LVGGK(1)DFEQPLAISR	0.71
Q29RF7	PDS5A	292	Sister chromatid cohesion protein PDS5 homolo	57.59	LK(1)SNDGEER	0.99
P30086	PEBP1	47	Phosphatidylethanolamine-binding protein 1;Hi	92.309	VLTPTQVK(1)NRPTSISWDGLI	0.74
P30086	PEBP1	150	Phosphatidylethanolamine-binding protein 1;Hi	111.73	FK(1)VASFR	0.99
Q9UBV8	PEF1	260	Peflin	107.57	EK(1)DTAVQGNIR	unquantifiable
O00541	PES1	81	Pescadillo homolog	89.548	FLLHEPIVNK(1)FR	1.07
O60925	PFDN1	83	Prefoldin subunit 1	43.297	EAIHSQLLEK(1)QK	Tip60 OE only

O60925	PFDN1	26	Prefoldin subunit 1	80.229	VIDTQQK(1)VK	unquantifiable
Q9UHV9	PFDN2	111	Prefoldin subunit 2	182.64	IIETLTQQLQAK(1)GK	0.86
Q99471	PFDN5	124	Prefoldin subunit 5	65.563	IQPALQEK(1)HAMK	0.87
P17858	PFKL	8	ATP-dependent 6-phosphofructokinase, liver typ	60.436	AAVDLEK(1)LR	unquantifiable
Q01813	PFKP	700	ATP-dependent 6-phosphofructokinase, platelet	67.563	AMEWITAK(1)LK	0.62
Q01813	PFKP	10	ATP-dependent 6-phosphofructokinase, platelet	49.358	APK(1)GSLR	0.9
Q01813	PFKP	625	ATP-dependent 6-phosphofructokinase, platelet	67.153	DLQSNVEHLTEK(1)MK	1.16
Q01813	PFKP	736	ATP-dependent 6-phosphofructokinase, platelet	78.655	NVIFQPVAELK(1)K	unquantifiable
P07737	PFN1	108	Profilin-1	116.52	TDK(1)TLVLLMGK	0.82
P07737	PFN1	105	Profilin-1	112.44	STGGAPTFNVTVTK(1)TDK	0.9
P07737	PFN1	126	Profilin-1	176.6	EGVHGGLINK(1)K	0.95
P07737	PFN1	91	Profilin-1	127.71	TK(1)STGGAPTFNVTVTK	0.95
P07737	PFN1	127	Profilin-1	119.86	K(1)CYEMASHLR	1.01
P07737	PFN1	116	Profilin-1	140.31	TLVLLMGK(1)EGVHGGLINK	1.08
P18669	PGAM1	157	Phosphoglycerate mutase 1	197.14	YADLTEDQLPSCESLK(1)DTIAF	1.01
P18669	PGAM1;PGAM2	251	Phosphoglycerate mutase 1;Phosphoglycerate m	93.649	AMEAVAAQGK(1)AK	0.84
P18669	PGAM1;PGAM4	113	Phosphoglycerate mutase 1;Probable phosphogl	78.939	HGEAQVK(1)IWR	Tip60 OE only
P18669	PGAM1;PGAM4;PGA	100	Phosphoglycerate mutase 1;Probable phosphogl	147.39	HYGGLTGLNK(1)AETAAK	0.92
P18669	PGAM1;PGAM4;PGA	106	Phosphoglycerate mutase 1;Probable phosphogl	81.338	AETAAK(1)HGEAQVK	0.93
P52209	PGD	48	6-phosphogluconate dehydrogenase, decarboxyl	97.734	VDDFLANEAK(1)GTK	0.85
P52209	PGD	316	6-phosphogluconate dehydrogenase, decarboxyl	71.342	FQFDGDK(1)K	0.96
P52209	PGD	51	6-phosphogluconate dehydrogenase, decarboxyl	105.98	GTK(1)VVGAQSLK	1.06
P52209	PGD	38	6-phosphogluconate dehydrogenase, decarboxyl	86.911	TVSK(1)VDDFLANEAK	1.12
P52209	PGD	375	6-phosphogluconate dehydrogenase, decarboxyl	58.981	SVFLGK(1)IK	1.14
P52209	PGD	119	6-phosphogluconate dehydrogenase, decarboxyl	117.92	AK(1)GILFVGSGVSGGEEGAR	1.18
P52209	PGD	59	6-phosphogluconate dehydrogenase, decarboxyl	120.09	VVGAQSLK(1)EMVSK	1.25
P52209	PGD	253	6-phosphogluconate dehydrogenase, decarboxyl	105.42	HLLPK(1)IR	1.28
P00558	PGK1	131	Phosphoglycerate kinase 1	117.89	FHVEEEGK(1)GK	0.79
P00558	PGK1	97	Phosphoglycerate kinase 1	140.31	DVLFLK(1)DCVGPEVEK	0.92
P00558	PGK1	11	Phosphoglycerate kinase 1	66.27	LTLDK(1)LDVK	0.95
P00558	PGK1	323	Phosphoglycerate kinase 1	98.458	K(1)YAEAVTR	1
P00558	PGK1	353	Phosphoglycerate kinase 1	85.377	GTK(1)ALMDEVVK	1
P00558	PGK1	272	Phosphoglycerate kinase 1	69.825	DLMSK(1)AEK	1.02
P00558	PGK1	146	Phosphoglycerate kinase 1	92.838	AEPAK(1)IEAFR	Tip60 OE only

P00558	PGK1	41	Phosphoglycerate kinase 1	47.732	IK(1)AAVPSIK	Tip60 OE only
P00558	PGK1	220	Phosphoglycerate kinase 1	45.915	VADK(1)IQLINNMLDK	Tip60 OE only
P00558	PGK1	361	Phosphoglycerate kinase 1	69.979	ALMDEVVK(1)ATSR	Tip60 OE only
P00558	PGK1	6	Phosphoglycerate kinase 1	74.6	SLSNK(1)LTLDK	unquantifiable
P00558	PGK1	141	Phosphoglycerate kinase 1	82.671	VK(1)AEPAK	unquantifiable
P00558	PGK1;PGK2	156	Phosphoglycerate kinase 1;Phosphoglycerate kin	65.887	ASLSK(1)LGDVYVNDAFGTAH	1.34
P35232	PHB	186	Prohibitin	372.73	EFTEAVEAK(1)QVAQQEAER	0.92
P35232	PHB	83	Prohibitin	147.35	NVPVITGSK(1)DLQNVNITLR	1.07
P35232	PHB	202	Prohibitin	150.24	FVVEK(1)AEQQK	Tip60 OE only
P35232	PHB	208	Prohibitin	67.93	K(1)AAIISAEGDSK	Tip60 OE only
Q99623	PHB2	244	Prohibitin-2	128.03	MLGEALSK(1)NPGYIK	0.88
Q99623	PHB2	250	Prohibitin-2	84.244	NPGYIK(1)LR	0.96
Q99623	PHB2	216	Prohibitin-2	69.628	AQFLVEK(1)AK	0.97
Q99623	PHB2	147	Prohibitin-2	138.24	SVVAK(1)FNASQLITQR	0.97
Q99623	PHB2	262	Prohibitin-2	230.41	AAQNISK(1)TIATSQNR	1.02
Q99623	PHB2	200	Prohibitin-2	249.75	EYTAAVEAK(1)QVAQQEAQR	1.02
Q7RTV0	PHF5A	25	PHD finger-like domain-containing protein 5A	53.166	LCEK(1)CDGK	0.84
Q9UPP1	PHF8	1037	Histone lysine demethylase PHF8	59.641	K(1)GLATAK	unquantifiable
O43175	PHGDH	69	D-3-phosphoglycerate dehydrogenase	144.57	VTADVINAAEK(1)LQVVGR	0.89
O43175	PHGDH	33	D-3-phosphoglycerate dehydrogenase	132.31	ILQDGGLQVVEK(1)QNLSK	1.08
O43175	PHGDH	384	D-3-phosphoglycerate dehydrogenase	60.342	EASK(1)QADVNLVNAK	1.2
O43175	PHGDH	58	D-3-phosphoglycerate dehydrogenase	80.522	SATK(1)VTADVINAAEK	Tip60 OE only
Q9NRX4	PHPT1	41	14 kDa phosphohistidine phosphatase	150.1	SGPAPAAESK(1)EIVR	1.16
Q13492	PICALM	324	Phosphatidylinositol-binding clathrin assembly	121.9	EK(1)QAALEEEQAR	Tip60 OE only
Q9Y237	PIN4	57	Peptidyl-prolyl cis-trans isomerase NIMA-interac	42.599	IMEAMEK(1)LK	0.82
Q8TBX8	PIP4K2C	265	Phosphatidylinositol 5-phosphate 4-kinase type-	45.718	IFLEK(1)LK	unquantifiable
P48739	PITPNB	5	Phosphatidylinositol transfer protein beta isofor	74.415	VLIK(1)EFR	0.92
P14618	PKM	261	Pyruvate kinase PKM	57.811	VLGEK(1)GK	0.99
P14618	PKM	498	Pyruvate kinase PKM	171.73	VNFAMNVGK(1)AR	1
P14618	PKM	247	Pyruvate kinase PKM	126.07	K(1)ASDVHEVR	1.01
P14618	PKM	66	Pyruvate kinase PKM	146.81	EMIK(1)SGMNVAR	1.01
P14618	PKM	270	Pyruvate kinase PKM	173.32	IISK(1)IENHEGVR	1.02
P14618	PKM	166	Pyruvate kinase PKM	96.342	NICK(1)VVEVGSK	1.04
P14618	PKM	186	Pyruvate kinase PKM	134.13	IYVDDGLISLQVK(1)QK	1.06

P14618	PKM	141	Pyruvate kinase PKM	98.803	GATLK(1)ITLDNAYMEK	1.16
P14618	PKM	62	Pyruvate kinase PKM	107.57	SVETLK(1)EMIK	1.33
P14618	PKM	115	Pyruvate kinase PKM	182.28	TATESFASDPILYRPVAVALDTK	Tip60 OE only
P14618	PKM	207	Pyruvate kinase PKM	81.647	K(1)GVNLPGAAVDLPAVSEK	Tip60 OE only
P14618	PKM;PKLR	311	Pyruvate kinase PKM;Pyruvate kinase PKLR	121.62	VFLAQK(1)MMIGR	unquantifiable
Q16513	PKN2	247	Serine/threonine-protein kinase N2	74.173	LLGSGK(1)VTDR	Tip60 OE only
P19174	PLCG1	803	1-phosphatidylinositol 4,5-bisphosphate phospl	54.157	ALFDYK(1)AQR	Tip60 OE only
Q15149	PLEC	3237	Plectin	60.434	LLSAEK(1)AVTGYR	0.7
Q15149	PLEC	792	Plectin	47.198	K(1)ESYSALMR	0.91
Q15149	PLEC	4205	Plectin	59.245	LPVEVAYK(1)R	1.06
Q15149	PLEC	2350	Plectin	66.568	LK(1)AEATEAAR	1.23
Q15149	PLEC	567	Plectin	65.252	LLAAGK(1)VPQR	1.39
Q15149	PLEC	2452	Plectin	168.74	EK(1)MQAVQEATR	1.39
Q15149	PLEC	3505	Plectin	53.751	EK(1)VSIYEAMR	Tip60 OE only
Q15149	PLEC	2650	Plectin	71.342	FIEQEK(1)AK	Tip60 OE only
O60664	PLIN3	122	Perilipin-3	81.865	VLADTK(1)ELVSSK	0.71
O60664	PLIN3	164	Perilipin-3	84.213	GAVQSGVDK(1)TK	0.79
O60664	PLIN3	128	Perilipin-3	124.42	ELVSSK(1)VSGAQEMVSSAK	1.01
P13797	PLS3	312	Plastin-3	72.496	AYFHLLNQIAPK(1)GQK	0.52
P13797	PLS3	346	Plastin-3	155	AESMLQQADK(1)LGCR	0.79
P13797	PLS3	59	Plastin-3	123.96	EIIQK(1)LMLDGDR	0.84
P13797	PLS3	444	Plastin-3	62.378	VPVDWSK(0.997)VNK(0.003	0.86
P13797	PLS3	545	Plastin-3	141.48	STSIQSFK(1)DK	0.98
P13797	PLS3	300	Plastin-3	161.1	DSK(1)AYFHLLNQIAPK	1.01
P13797	PLS3	611	Plastin-3	103.34	VYALPEDLVEVK(1)PK	1.01
P13797	PLS3	335	Plastin-3	128.67	IDINMSGFNETDDLK(1)R	1.03
P13797	PLS3	582	Plastin-3	73.927	SGNLTEDDK(1)HNNAK	1.06
P13797	PLS3	52	Plastin-3	147.2	EANMPLPGYK(1)VR	1.06
P13797	PLS3	91	Plastin-3	109.11	SSDIAK(1)TFR	1.22
P13797	PLS3	100	Plastin-3	103.63	K(1)EGICALGGTSELSSEGTQH	1.34
P13797	PLS3	537	Plastin-3	255.3	TLSEAGK(1)STSIQSFK	Tip60 OE only
O75439	PMPCB	219	Mitochondrial-processing peptidase subunit bet	143.97	TILGPTENIK(1)SISR	1.08
Q9H307	PNN	14	Pinin	107.57	TLQEQLEK(1)AK	0.85
Q9NP80	PNPLA8	756	Calcium-independent phospholipase A2-gamma	76.064	ILSQEK(1)TTLQK	Tip60 OE only

P30876	POLR2B	644	DNA-directed RNA polymerase II subunit RPB2	60.589	HIDQLK(1)ER	unquantifiable
Q15181	PPA1	70	Inorganic pyrophosphatase	75.294	DPLNPIK(1)QDVK	0.87
Q15181	PPA1	213	Inorganic pyrophosphatase	70.552	DK(1)DFAIDIIK	0.89
Q15181	PPA1	63	Inorganic pyrophosphatase	128.81	MEIATK(1)DPLNPIK	0.95
Q9H2U2	PPA2	261	Inorganic pyrophosphatase 2, mitochondrial	54.608	NK(1)AFALEVIK	0.95
Q9NQ55	PPAN	217	Suppressor of SWI4 1 homolog	46.001	LLQEK(1)FPNMSR	unquantifiable
Q06203	PPAT	411	Amidophosphoribosyltransferase	65.465	ESGAK(1)EVHIR	Tip60 OE only
Q13136	PPFIA1	270	Liprin-alpha-1	41.029	EQSQMK(1)ER	unquantifiable
Q13136	PPFIA1;PPFIA4;PPFIA	177	Liprin-alpha-1;Liprin-alpha-4;Liprin-alpha-3;Lipr	64.776	ALDEK(1)VR	Tip60 OE only
P62937	PPIA	44	Peptidyl-prolyl cis-trans isomerase A;Peptidyl-pr	126.07	ALSTGEK(1)GFGYK	0.95
P62937	PPIA	125	Peptidyl-prolyl cis-trans isomerase A;Peptidyl-pr	143.79	TEWLDGK(1)HVVFGK	0.96
P62937	PPIA	133	Peptidyl-prolyl cis-trans isomerase A;Peptidyl-pr	118.48	VK(1)EGMNIVEAMER	1
P62937	PPIA	31	Peptidyl-prolyl cis-trans isomerase A;Peptidyl-pr	133.55	VPK(1)TAENFR	1.01
P62937	PPIA	82	Peptidyl-prolyl cis-trans isomerase A;Peptidyl-pr	116.54	SIYGEK(1)FEDENFILK	1.07
P62937	PPIA	49	Peptidyl-prolyl cis-trans isomerase A;Peptidyl-pr	127.76	GFGYK(1)GSCFHR	1.13
P23284	PPIB	131	Peptidyl-prolyl cis-trans isomerase B	45.723	LK(1)HYGPGWVSMANAGK	0.79
P23284	PPIB	215	Peptidyl-prolyl cis-trans isomerase B	78.342	IEVEKPFAIAK(1)E	0.9
P23284	PPIB	165	Peptidyl-prolyl cis-trans isomerase B	65.563	TAWLDGK(1)HVVFGK	0.96
P23284	PPIB	116	Peptidyl-prolyl cis-trans isomerase B	124.42	GDGTGGK(1)SIYGER	Tip60 OE only
P23284	PPIB	204	Peptidyl-prolyl cis-trans isomerase B	59.512	DVIIADCGK(0.999)IEVEK(0.0	Tip60 OE only
P23284	PPIB	171	Peptidyl-prolyl cis-trans isomerase B	98.353	HVVFGK(1)VLEGMEVVR	Tip60 OE only
P30405	PPIF	91	Peptidyl-prolyl cis-trans isomerase F, mitochond	65.5	GFGYK(1)GSTFHR	1.07
P30405	PPIF	67	Peptidyl-prolyl cis-trans isomerase F, mitochond	93.096	VVLELK(1)ADVVPK	1.08
P30405	PPIF	183	Peptidyl-prolyl cis-trans isomerase F, mitochond	71.877	K(1)IESFGSK	1.19
P30405	PPIF	86	Peptidyl-prolyl cis-trans isomerase F, mitochond	115.71	ALCTGEK(1)GFGYK	Tip60 OE only
O15355	PPM1G	519	Protein phosphatase 1G	137.98	NTAELQPESGK(1)R	0.79
Q9Y570	PPME1	293	Protein phosphatase methylesterase 1	63.565	IELAK(1)TEK	1.04
P36873	PPP1CC;PPP1CB;PPF	141	Serine/threonine-protein phosphatase PP1-gamr	101.25	IYGFYDECK(1)R	0.75
P36873	PPP1CC;PPP1CB;PPF	260	Serine/threonine-protein phosphatase PP1-gamr	122.7	AHQVVEDGYEFFAK(1)R	1.06
Q15435	PPP1R7	101	Protein phosphatase 1 regulatory subunit 7	90.706	VK(1)TLCLR	Tip60 OE only
P67775	PPP2CA;PPP2CB	41	Serine/threonine-protein phosphatase 2A catalyt	185.08	EILTK(1)ESNVQEVR	0.78
P30153	PPP2R1A	472	Serine/threonine-protein phosphatase 2A 65 kDa	50.966	LVEK(1)FGK	0.85
P30153	PPP2R1A	272	Serine/threonine-protein phosphatase 2A 65 kDa	83.137	FTELQK(1)AVGPEITK	0.88
P30153	PPP2R1A;PPP2R1B	188	Serine/threonine-protein phosphatase 2A 65 kDa	71.451	AAASK(1)LGEFAK	1.07

P30153	PPP2R1A;PPP2R1B	107	Serine/threonine-protein phosphatase 2A 65 kDa	123.69	DK(1)AVESLR	1.1
P60510	PPP4C	31	Serine/threonine-protein phosphatase 4 catalyti	98.03	ALCAK(1)AR	unquantifiable
P53041	PPP5C	93	Serine/threonine-protein phosphatase 5	69.383	AIELDK(1)K	unquantifiable
P53041	PPP5C	111	Serine/threonine-protein phosphatase 5	131.41	AASNMALGK(1)FR	unquantifiable
P53041	PPP5C;PPP5D1	135	Serine/threonine-protein phosphatase 5;Protein	89.296	MK(1)YQECNK	unquantifiable
Q06830	PRDX1	197	Peroxiredoxin-1	114.02	EYFSK(1)QK	0.91
Q06830	PRDX1	192	Peroxiredoxin-1	112.17	SK(1)EYFSK	0.93
Q06830	PRDX1	35	Peroxiredoxin-1	121.96	DISLSDYK(1)GK	0.97
Q06830	PRDX1	27	Peroxiredoxin-1	185.2	ATAVMPDGQFK(1)DISLSDYK	0.97
Q06830	PRDX1	7	Peroxiredoxin-1	127.83	SSGNAK(1)IGHPAPNFK	0.98
Q06830	PRDX1	120	Peroxiredoxin-1	148.41	TIAQDYGVLK(1)ADEGISFR	0.99
Q06830	PRDX1	16	Peroxiredoxin-1	86.8	IGHPAPNFK(1)ATAVMPDGQI	1.02
Q06830	PRDX1	109	Peroxiredoxin-1	40.844	K(0.001)QGGLGPMNIPLVSD	Tip60 OE only
P32119	PRDX2	16	Peroxiredoxin-2	86.803	PAPDFK(1)ATAVVDGAFK	0.84
P32119	PRDX2	26	Peroxiredoxin-2	136.02	ATAVVDGAFK(1)EVK	0.84
P32119	PRDX2	196	Peroxiredoxin-2	65.672	EYFSK(1)HN	0.93
P32119	PRDX2	34	Peroxiredoxin-2	53.554	LSDYK(1)GK	0.94
P32119	PRDX2	29	Peroxiredoxin-2	85.212	EVK(1)LSDYK	0.97
P32119	PRDX2	119	Peroxiredoxin-2	247.29	LSEDYGVLK(1)TDEGIAYR	1.05
P30048	PRDX3	91	Thioredoxin-dependent peroxide reductase, mito	107.47	DLSLDDFK(1)GK	0.89
Q13162	PRDX4	263	Peroxiredoxin-4	49.638	HGEVCPAGWKPGSETIIPDPA	0.94
Q13162	PRDX4	78	Peroxiredoxin-4	73.848	VSVADHSLHLSK(1)AK	1.01
Q13162	PRDX4	102	Peroxiredoxin-4	89.296	ELK(1)LTDYR	Tip60 OE only
P30044	PRDX5	116	Peroxiredoxin-5, mitochondrial	71.08	THLPGFVEQAEALK(1)AK	Tip60 OE only
P30041	PRDX6	63	Peroxiredoxin-6	101.69	LAPEFAK(1)R	0.77
P30041	PRDX6	209	Peroxiredoxin-6	102.06	GVFTK(1)ELPSGK	0.91
P30041	PRDX6	144	Peroxiredoxin-6	106.29	LK(1)LSILYPATTGR	1.04
P30041	PRDX6	141	Peroxiredoxin-6	114.89	VVFVFGPDK(1)K	1.21
P30041	PRDX6	56	Peroxiredoxin-6	72.879	AAK(1)LAPEFAK	Tip60 OE only
P22694	PRKACB;PRKACA	24	cAMP-dependent protein kinase catalytic subuni	87.323	AK(1)EDFLK	0.9
P14314	PRKCSH	180	Glucosidase 2 subunit beta	94.767	TVK(1)EEAEKPER	0.82
P14314	PRKCSH	166	Glucosidase 2 subunit beta	96.253	LIELQAGK(1)K	0.95
P14314	PRKCSH	196	Glucosidase 2 subunit beta	43.042	EQHQK(1)LWEEQLAAAK	0.97
P14314	PRKCSH	376	Glucosidase 2 subunit beta	70.525	NK(1)FEEAER	0.97

P14314	PRKCSH	158	Glucosidase 2 subunit beta	89.355	K(1)LIELQAGK	1.01
P14314	PRKCSH	185	Glucosidase 2 subunit beta	75.109	EEAEK(1)PER	Tip60 OE only
P14314	PRKCSH	167	Glucosidase 2 subunit beta	132.01	K(1)SLEDQVEMLR	Tip60 OE only
P78527	PRKDC	2259	DNA-dependent protein kinase catalytic subunit	52.255	LIFEK(1)FSGK	0.92
P78527	PRKDC	263	DNA-dependent protein kinase catalytic subunit	131.82	AIRPQIDLK(1)R	0.94
P78527	PRKDC	2715	DNA-dependent protein kinase catalytic subunit	46.844	LGLPGDEVDNK(1)VK	0.95
P78527	PRKDC	1627	DNA-dependent protein kinase catalytic subunit	62.303	LATTILQHWK(1)K	0.97
P78527	PRKDC	1857	DNA-dependent protein kinase catalytic subunit	83.047	FTK(1)LNESTFDTQITK	1.04
P78527	PRKDC	2829	DNA-dependent protein kinase catalytic subunit	138.66	TLSEK(1)NNITQK	1.04
P78527	PRKDC	3598	DNA-dependent protein kinase catalytic subunit	76.605	AELAK(1)TPVNK	1.06
P78527	PRKDC	2824	DNA-dependent protein kinase catalytic subunit	55.531	FK(1)TLSEK	1.09
P78527	PRKDC	2908	DNA-dependent protein kinase catalytic subunit	47.869	LLPAELPAK(1)R	1.13
P78527	PRKDC	117	DNA-dependent protein kinase catalytic subunit	117.7	NTCTSVYTK(1)DR	1.17
P78527	PRKDC	1892	DNA-dependent protein kinase catalytic subunit	124.92	DDVHAK(1)ESK	1.19
P78527	PRKDC	2764	DNA-dependent protein kinase catalytic subunit	87.476	MK(1)QDAQVVLYR	1.5
P78527	PRKDC	3840	DNA-dependent protein kinase catalytic subunit	83.862	APPCEYK(1)DWLTK	Tip60 OE only
P78527	PRKDC	2227	DNA-dependent protein kinase catalytic subunit	58.661	HVFHPK(1)R	unquantifiable
P78527	PRKDC	71	DNA-dependent protein kinase catalytic subunit	46.158	K(1)SLNSIEFR	unquantifiable
P78527	PRKDC	1057	DNA-dependent protein kinase catalytic subunit	110.76	SPVNTK(1)SLFK	unquantifiable
Q99873	PRMT1	233	Protein arginine N-methyltransferase 1	57.802	DVAIK(1)EPLVDVVDPK	0.74
Q99873	PRMT1	143	Protein arginine N-methyltransferase 1	117.7	LDHVVTIIK(1)GK	0.89
Q99873	PRMT1	134	Protein arginine N-methyltransferase 1	80.318	ANK(1)LDHVVTIIK	0.98
Q99873	PRMT1;PRMT8	145	Protein arginine N-methyltransferase 1;Protein a	113.69	GK(1)VEEVELPVEK	1
O14744	PRMT5	387	Protein arginine N-methyltransferase 5;Protein a	51.193	IK(1)LYAVEK	1.14
Q9UMS4	PRPF19	206	Pre-mRNA-processing factor 19	73.208	TVPEELVK(0.076)PEELSK(0.9	0.68
Q9UMS4	PRPF19	244	Pre-mRNA-processing factor 19	101.6	ILTGGADK(1)NVVVFDK	0.84
Q9UMS4	PRPF19	261	Pre-mRNA-processing factor 19	42.568	SSEQILATLK(1)GHTK	1.05
Q9UMS4	PRPF19	179	Pre-mRNA-processing factor 19	140.07	LQDK(1)ATVLTTER	1.06
Q9UMS4	PRPF19	122	Pre-mRNA-processing factor 19	73.665	LTK(1)EVTAAR	1.19
Q8WWY3	PRPF31	298	U4/U6 small nuclear ribonucleoprotein Prp31	103.55	LVAAK(1)CTLAAR	1.06
O75400	PRPF40A	751	Pre-mRNA-processing factor 40 homolog A	60.436	ESAFK(1)SMLK	Tip60 OE only
O94906	PRPF6	755	Pre-mRNA-processing factor 6	66.19	LEEK(1)IGQLTR	Tip60 OE only
Q6P2Q9	PRPF8	555	Pre-mRNA-processing-splicing factor 8	51.726	LTK(1)LVVDSHVQYR	0.51
Q6P2Q9	PRPF8	769	Pre-mRNA-processing-splicing factor 8	56.729	GATVDK(1)TVCK	0.9

Q6P2Q9	PRPF8	853	Pre-mRNA-processing-splicing factor 8	75.692	EAYSVK(1)SR	1.35
P11908	PRPS2;PRPS1	212	Ribose-phosphate pyrophosphokinase 2;Ribose-p	116.52	MVLVGDVK(1)DR	unquantifiable
P11908	PRPS2;PRPS1;PRPS1	29	Ribose-phosphate pyrophosphokinase 2;Ribose-p	45.908	LGLELGK(1)VVTK	Tip60 OE only
O60256	PRPSAP2;PRPSAP1	335	Phosphoribosyl pyrophosphate synthase-associa	44.611	LQCPK(1)IK	0.83
Q9Y617	PSAT1	311	Phosphoserine aminotransferase	69.672	IGNAK(1)GDDALEK	0.95
Q9Y617	PSAT1	318	Phosphoserine aminotransferase	98.299	GDDALEK(1)R	1.09
P25786	PSMA1	61	Proteasome subunit alpha type-1	107.57	AQSELAAHQK(1)K	0.86
P25786	PSMA1	41	Proteasome subunit alpha type-1	145.46	SK(1)THAVLVALK	0.93
P25786	PSMA1	208	Proteasome subunit alpha type-1	84.468	ETLPAEQDLTTK(1)NVSIGIVGK	1.15
P25786	PSMA1	115	Proteasome subunit alpha type-1	172.34	LVSLIGSK(1)TQIPTQR	Tip60 OE only
P25788	PSMA3	179	Proteasome subunit alpha type-3	87.616	TEIEK(1)LQMK	0.76
P25788	PSMA3	65	Proteasome subunit alpha type-3	186.13	LYEEGSNK(1)R	0.96
P25788	PSMA3	183	Proteasome subunit alpha type-3	167.24	LQMK(1)EMTCR	0.97
P25788	PSMA3	206	Proteasome subunit alpha type-3	60.434	IIYIVHDEVK(1)DK	0.98
P25789	PSMA4	199	Proteasome subunit alpha type-4	134.26	VLNK(1)TMDVSK	0.91
P25789	PSMA4	210	Proteasome subunit alpha type-4	85.729	LSAEK(1)VEIATLTR	1.08
P25789	PSMA4	205	Proteasome subunit alpha type-4	83.869	TMDVSK(1)LSAEK	1.09
P25789	PSMA4	246	Proteasome subunit alpha type-4	40.352	HEEEEAK(1)AER	2.34
P28066	PSMA5	187	Proteasome subunit alpha type-5	87.659	AIGSASEGAQSSLQEVYHK(1)S	0.75
P28066	PSMA5	192	Proteasome subunit alpha type-5	71.379	SMTLK(1)EAIK	Tip60 OE only
P60900	PSMA6	102	Proteasome subunit alpha type-6	87.616	YEAANWK(1)YK	1.08
P60900	PSMA6	171	Proteasome subunit alpha type-6	120.87	ATAAGVK(1)QTESTSFLEK	1.21
O14818	PSMA7	115	Proteasome subunit alpha type-7	107.43	YIASLK(1)QR	1.51
O14818	PSMA7;PSMA8	52	Proteasome subunit alpha type-7;Proteasome su	97.635	SVAK(1)LQDER	0.73
P20618	PSMB1	228	Proteasome subunit beta type-1	92.062	ICIVTK(1)EGIR	Tip60 OE only
P49720	PSMB3	77	Proteasome subunit beta type-3	102.07	LNLYELK(1)EGR	1
P28070	PSMB4	201	Proteasome subunit beta type-4	179.35	EVLEK(1)QPVLSQTEAR	0.83
Q99436	PSMB7	127	Proteasome subunit beta type-7	97.071	MLK(1)QMLFR	0.82
Q99436	PSMB7	72	Proteasome subunit beta type-7	140.39	ATEGMVVADK(1)NCSK	0.82
P62191	PSMC1	232	26S protease regulatory subunit 4	77.593	GVILYGPPGTGK(1)TLLAK	0.97
P62191	PSMC1	423	26S protease regulatory subunit 4	85.212	SK(1)ENVLYK	1.09
P62191	PSMC1	98	26S protease regulatory subunit 4	75.109	SK(1)VDDLR	1.16
P35998	PSMC2	116	26S protease regulatory subunit 7	61.999	YIINVK(1)QFAK	0.99
P35998	PSMC2	248	26S protease regulatory subunit 7	113.77	VIGSELVQK(1)YVGEGAR	Tip60 OE only

P17980	PSMC3	245	26S protease regulatory subunit 6A	140.75	ACAAQTK(1)ATFLK	0.94
P17980	PSMC3	56	26S protease regulatory subunit 6A	87.754	IMK(1)SEVLR	0.95
P17980	PSMC3	300	26S protease regulatory subunit 6A	90.657	FDSEK(1)AGDR	1.01
P17980	PSMC3	70	26S protease regulatory subunit 6A	48.568	VTHELQAMK(1)DK	1.13
P43686	PSMC4	238	26S protease regulatory subunit 6B	126.76	VVGSEFVQK(1)YLGEGPR	Tip60 OE only
P62195	PSMC5	196	26S protease regulatory subunit 8	86.772	GVLLYGPPGTGK(1)TLLAR	0.86
P62195	PSMC5	290	26S protease regulatory subunit 8	78.516	NIK(1)VIMATNR	0.93
P62195	PSMC5	38	26S protease regulatory subunit 8	132.47	IEELQLIVNDK(1)SQNLR	1.19
P62195	PSMC5	162	26S protease regulatory subunit 8	74.162	EIK(1)EVIELPVK	Tip60 OE only
P62333	PSMC6	20	26S protease regulatory subunit 10B	50.284	LLEHK(1)EIDGR	Tip60 OE only
Q99460	PSMD1	319	26S proteasome non-ATPase regulatory subunit 1	156.51	TPEASPEPK(1)DQTLK	0.74
Q99460	PSMD1	720	26S proteasome non-ATPase regulatory subunit 1	87.001	VINDK(1)HDDVMAK	0.79
Q99460	PSMD1	148	26S proteasome non-ATPase regulatory subunit 1	97.813	YK(1)QAIGIALETR	1.37
O75832	PSMD10	30	26S proteasome non-ATPase regulatory subunit 1	94.616	ESILADK(1)SLATR	unquantifiable
O00231	PSMD11	358	26S proteasome non-ATPase regulatory subunit 1	60.436	LSK(1)ADVER	0.96
O00231	PSMD11	417	26S proteasome non-ATPase regulatory subunit 1	61.679	VVDSLYNK(1)AK	Tip60 OE only
O00232	PSMD12	212	26S proteasome non-ATPase regulatory subunit 1	74.611	INTK(1)FFQEENTEK	0.81
O00232	PSMD12	98	26S proteasome non-ATPase regulatory subunit 1	64.265	SQLK(1)QAVAK	1
O00232	PSMD12	147	26S proteasome non-ATPase regulatory subunit 1	122.52	LTK(1)TLATIK	1.11
O00232	PSMD12	295	26S proteasome non-ATPase regulatory subunit 1	51.43	K(1)LEEIPK	unquantifiable
Q9UNM6	PSMD13	321	26S proteasome non-ATPase regulatory subunit 1	72.819	ALSVGLVK(1)GSIDEVDKR	0.94
Q9UNM6	PSMD13	105	26S proteasome non-ATPase regulatory subunit 1	83.862	VK(1)SSDEAVILCK	0.94
O00487	PSMD14	152	26S proteasome non-ATPase regulatory subunit 1	115.18	AVAVVVDPIQSVK(1)GK	1.04
Q13200	PSMD2	397	26S proteasome non-ATPase regulatory subunit 2	91.584	LLTDDGNK(1)WLYK	0.91
Q13200	PSMD2	551	26S proteasome non-ATPase regulatory subunit 2	156.92	SETELK(1)DTYAR	1.01
O43242	PSMD3	194	26S proteasome non-ATPase regulatory subunit 3	136.5	ISDDLMQK(1)ISTQNR	1.05
Q15008	PSMD6	165	26S proteasome non-ATPase regulatory subunit 6	127.42	AK(1)SLIEEGGDWDR	Tip60 OE only
Q15008	PSMD6	107	26S proteasome non-ATPase regulatory subunit 6	67.726	DAMMAK(1)AEYLCR	Tip60 OE only
P51665	PSMD7	180	26S proteasome non-ATPase regulatory subunit 7	186.87	DIK(1)DTTVGTLSQR	0.81
P51665	PSMD7	204	26S proteasome non-ATPase regulatory subunit 7	64.297	GLNSK(1)LLDIR	Tip60 OE only
Q9UL46	PSME2	156	Proteasome activator complex subunit 2	85.845	TK(1)VEAFQTTISK	unquantifiable
P61289	PSME3	132	Proteasome activator complex subunit 3	96.668	LLIEK(1)CNTVK	1.04
P61289	PSME3	14	Proteasome activator complex subunit 3	84.244	LK(1)VDSFR	1.09
P61289	PSME3	195	Proteasome activator complex subunit 3	50.786	IAK(1)YPHVEDYR	1.16

P61289	PSME3	6	Proteasome activator complex subunit 3	96.665	ASLLK(1)VDQEVK	Tip60 OE only
Q8WXF1	PSPC1	287	Paraspeckle component 1	179.21	ALDEMEK(1)QQR	0.9
Q8WXF1	PSPC1	303	Paraspeckle component 1	90.108	EK(1)LEAEMEAAR	1.07
Q8WXF1	PSPC1	143	Paraspeckle component 1	136.33	AELDGTILK(1)SR	1.24
P26599	PTBP1	402	Polypyrimidine tract-binding protein 1	40.686	LHGK(1)PIR	0.83
P26599	PTBP1	428	Polypyrimidine tract-binding protein 1	53.504	EGQEDQGLTK(1)DYGNSPLHF	0.94
P26599	PTBP1	137	Polypyrimidine tract-binding protein 1	116.3	ELK(1)TDSSPNQAR	1.03
P26599	PTBP1	485	Polypyrimidine tract-binding protein 1	82.939	GFK(1)FFQK	1.03
P26599	PTBP1	410	Polypyrimidine tract-binding protein 1	123.79	ITLSK(1)HQNVQLPR	1.26
P26599	PTBP1;PTBP3	259	Polypyrimidine tract-binding protein 1;Polypyri	118.4	IDFSK(1)LTSLNVK	1.03
Q96EY7	PTCD3	158	Pentatricopeptide repeat domain-containing pro	76.465	DISEAALK(1)ER	unquantifiable
Q9H7Z7	PTGES2	225	Prostaglandin E synthase 2;Prostaglandin E synth	96.015	EAQQVYGGK(1)EAR	0.62
Q15185	PTGES3	35	Prostaglandin E synthase 3	134.99	SK(1)LTFSCLGGSDNFK	0.99
Q15185	PTGES3	79	Prostaglandin E synthase 3	92.051	K(1)GESGQSWPR	1.14
Q15185	PTGES3	7	Prostaglandin E synthase 3	61.353	MQPASAK(1)WYDR	1.37
Q15185	PTGES3	33	Prostaglandin E synthase 3	77.192	DVNVNFEK(1)SK	Tip60 OE only
Q14914	PTGR1	178	Prostaglandin reductase 1	79.693	VVGAVGSDEK(1)VAYLQK	unquantifiable
P06454	PTMA	103	Prothymosin alpha;Prothymosin alpha, N-termir	149.23	AAEDDEDDDVDTK(1)K	1.04
P20962	PTMS	92	Parathymosin	144.77	AAEEEDEADPK(1)R	0.96
P20962	PTMS	27	Parathymosin	69.998	VEEK(1)ASR	1.22
P20962	PTMS	4	Parathymosin	54.343	SEK(1)SVEAAAELSAK	Tip60 OE only
Q9UHX1	PUF60	80	Poly(U)-binding-splicing factor PUF60	74.944	YAMEQSIK(1)SVLVK	1.05
Q96C36	PYCR2	283	Pyrroline-5-carboxylate reductase 2	40.268	ISPAALK(1)K	unquantifiable
P11216	PYGB	438	Glycogen phosphorylase, brain form	67.563	MSVIEEGDCK(1)R	Tip60 OE only
P06737	PYGL	438	Glycogen phosphorylase, liver form	75.378	MSLIEEEGSK(1)R	unquantifiable
P06737	PYGL;PYGM;PYGB	42	Glycogen phosphorylase, liver form;Glycogen ph	57.532	HLHFTLVK(1)DR	1.09
P06737	PYGL;PYGM;PYGB	290	Glycogen phosphorylase, liver form;Glycogen ph	102.4	VLYPNDNFFEGK(1)ELR	Tip60 OE only
Q96PU8	QKI	111	Protein quaking	78.908	GLTAK(1)QLEAETGCK	unquantifiable
P61026	RAB10	49	Ras-related protein Rab-10	91.069	IK(1)TVELQGK	unquantifiable
P62491	RAB11A	179	Ras-related protein Rab-11A	122.13	IVSQK(1)QMSDR	1.13
P62491	RAB11A;RAB11B	24	Ras-related protein Rab-11A;Ras-related protein I	143.86	VVLIGDSGVGK(1)SNLLSR	0.98
P62491	RAB11A;RAB11B	61	Ras-related protein Rab-11A;Ras-related protein I	128.85	TIK(1)AQIWDTAGQER	1.09
P51153	RAB13	138	Ras-related protein Rab-13	56.011	EQADK(1)LAR	unquantifiable
P61106	RAB14	59	Ras-related protein Rab-14	84.476	IIEVSGQK(1)IK	0.87

P62820	RAB1A	187	Ras-related protein Rab-1A	76.073	MGPGATAGGAEK(1)SNVK	0.87
P62820	RAB1A	125	Ras-related protein Rab-1A	114.4	LLVGNK(1)CDLTTK	0.96
P62820	RAB1A;RAB1B	119	Ras-related protein Rab-1A;Ras-related protein Ra	144.55	YASENVNK(1)LLVGNK	0.98
P62820	RAB1A;RAB1B;RAB1C	61	Ras-related protein Rab-1A;Ras-related protein Ra	128.85	TIK(1)LQIWDTAGQER	0.95
P62820	RAB1A;RAB1B;RAB1C	58	Ras-related protein Rab-1A;Ras-related protein Ra	117.89	TIELDGK(1)TIK	0.97
Q9H0U4	RAB1B;RAB1C	122	Ras-related protein Rab-1B;Putative Ras-related p	105.98	LLVGNK(1)SDLTTK	0.92
Q9H0U4	RAB1B;RAB1C	128	Ras-related protein Rab-1B;Putative Ras-related p	70.912	SDLTTK(1)K	1.02
P51148	RAB5C	141	Ras-related protein Rab-5C	94.262	ADLASK(1)R	0.93
P20340	RAB6A;RAB6B	26	Ras-related protein Rab-6A;Ras-related protein Ra	87.498	LVFLGEQSVGK(1)TSLITR	0.7
P20340	RAB6A;RAB6C	164	Ras-related protein Rab-6A;Ras-related protein Ra	111.01	AGYNVK(1)QLFR	1.15
P61006	RAB8A	55	Ras-related protein Rab-8A	63.48	TIELDGK(1)R	Tip60 OE only
P63000	RAC1	153	Ras-related C3 botulinum toxin substrate 1	149.83	EIGAVK(1)YLECSALTQR	0.99
P63000	RAC1;RAC2	123	Ras-related C3 botulinum toxin substrate 1;Ras-r	47.657	DDK(1)DTIEK	0.81
P63000	RAC1;RAC2;RAC3	166	Ras-related C3 botulinum toxin substrate 1;Ras-r	67.704	GLK(1)TVFDEAIR	Tip60 OE only
P54727	RAD23B	36	UV excision repair protein RAD23 homolog B	57.55	GK(1)DAFPVAGQK	0.84
P54727	RAD23B	24	UV excision repair protein RAD23 homolog B	112.15	IDIDPEETVK(1)ALK	1.06
P54727	RAD23B	60	UV excision repair protein RAD23 homolog B	69.349	ILNDDTALK(1)EYK	1.25
P54727	RAD23B;RAD23A	45	UV excision repair protein RAD23 homolog B;UV	80.763	DAFPVAGQK(1)LIYAGK	0.99
Q92878	RAD50	832	DNA repair protein RAD50	174.98	TVQQVNQEK(1)QEK	1
Q92878	RAD50	493	DNA repair protein RAD50	72.643	AEK(1)NSNVETLK	1.01
Q92878	RAD50	446	DNA repair protein RAD50	125.48	IIELK(1)SEILSK	1.05
Q92878	RAD50	421	DNA repair protein RAD50	83.087	TANQLMNDFAEK(1)ETLK	1.06
Q92878	RAD50	871	DNA repair protein RAD50	44.309	STTNELK(1)SEK	1.17
Q92878	RAD50	548	DNA repair protein RAD50	87.323	ADK(1)DEQIR	1.19
Q92878	RAD50	926	DNA repair protein RAD50	85.377	FQQEK(1)EELINK	unquantifiable
Q9P0K7	RAI14	594	Ankycorbin	55.549	EK(1)AFLFEK	unquantifiable
Q9UKM9	RALY	198	RNA-binding protein Raly	217.09	TELTQIK(1)SNIDALLSR	0.79
Q9UKM9	RALY	159	RNA-binding protein Raly	44.349	VK(1)TNVPVK	0.87
Q9UKM9	RALY	179	RNA-binding protein Raly	112.36	STAVTTSSAK(1)IK	0.88
Q9UKM9	RALY	183	RNA-binding protein Raly	53.517	LK(1)SSELQAIK	0.98
Q9UKM9	RALY	13	RNA-binding protein Raly	61.353	LQASNVTNK(1)NDPK	1
P62826	RAN	37	GTP-binding nuclear protein Ran	120.15	HLTGEFEK(1)K	0.86
P62826	RAN	142	GTP-binding nuclear protein Ran	170.62	K(0.079)K(0.921)NLQYYDISA	0.93
P62826	RAN	60	GTP-binding nuclear protein Ran	125.28	GPIK(1)FNVWDTAGQEK	0.94

P62826	RAN	123	GTP-binding nuclear protein Ran	90.412	VCENIPIVLCGNK(1)VDIK	0.95
P62826	RAN	99	GTP-binding nuclear protein Ran	94.692	VTYK(1)NVPNWHR	0.99
P62826	RAN	71	GTP-binding nuclear protein Ran	206.58	FNVWDTAGQEK(1)FGGLR	1.03
P62826	RAN	23	GTP-binding nuclear protein Ran	150.06	LVLVGDGGTGK(1)TTFVK	1.04
P62826	RAN	134	GTP-binding nuclear protein Ran	81.297	AK(1)SIVFHR	1.08
P43487	RANBP1	183	Ran-specific GTPase-activating protein	68.49	VAEK(1)LEALSVK	0.88
P43487	RANBP1	200	Ran-specific GTPase-activating protein	41.448	EETK(0.007)EDAEEK(0.993)C	0.95
P43487	RANBP1	68	Ran-specific GTPase-activating protein	187.42	FASENDLPEWK(1)ER	0.99
P43487	RANBP1	154	Ran-specific GTPase-activating protein	107.59	TK(1)FEECR	1.09
P49792	RANBP2	2951	E3 SUMO-protein ligase RanBP2	101.64	DVSQWK(1)ER	Tip60 OE only
P49792	RANBP2;RGPD3	2053	E3 SUMO-protein ligase RanBP2;RanBP2-like and	104.22	FDAEVSQWK(1)ER	unquantifiable
P49792	RANBP2;RGPD3;RGP	365	E3 SUMO-protein ligase RanBP2;RanBP2-like and	42.229	GK(1)QDFLK	0.54
P46060	RANGAP1	26	Ran GTPase-activating protein 1	78.653	TQVAGGQLSFK(1)GK	0.75
P46060	RANGAP1	306	Ran GTPase-activating protein 1	96.342	ELNLSFCEIK(1)R	0.97
P46060	RANGAP1	279	Ran GTPase-activating protein 1	93.258	SK(1)GAVAIADAIR	1.1
P46060	RANGAP1	481	Ran GTPase-activating protein 1	108.47	VSSVFK(1)DEATVR	Tip60 OE only
A6NIZ1	RAP1B	104	Ras-related protein Rap-1b-like protein;Ras-relat	130.56	VK(1)DTDDVPMILVGNK	0.89
A6NIZ1	RAP1B;RAP1A	128	Ras-related protein Rap-1b-like protein;Ras-relat	113.71	VVGK(1)EQGQNLAR	1
P54136	RARS	287	Arginine--tRNA ligase, cytoplasmic	103.56	FDTEEEFK(1)K	0.92
P54136	RARS	557	Arginine--tRNA ligase, cytoplasmic	215.89	LANIDEEMLQK(1)AAR	1
P54136	RARS	570	Arginine--tRNA ligase, cytoplasmic	93.096	ILLDHEK(1)EWK	1.05
P54136	RARS	471	Arginine--tRNA ligase, cytoplasmic	91.584	LMDLLGEGLK(1)R	1.17
P54136	RARS	393	Arginine--tRNA ligase, cytoplasmic	104.24	SDGGYTYDTSDLAAIK(1)QR	1.37
Q09028	RBBP4	4	Histone-binding protein RBBP4	101.6	ADK(1)EAAFDDAVEER	0.81
P49756	RBM25	837	RNA-binding protein 25	71.223	LLIYETEAK(1)K	0.62
Q9NW13	RBM28	465	RNA-binding protein 28	104.52	AAEGVSAADMAK(1)R	1.25
Q14498	RBM39	322	RNA-binding protein 39	40.154	PMK(1)VGHVTER	1.13
Q9BQ04	RBM4B;RBM4	139	RNA-binding protein 4B;RNA-binding protein 4	67.563	GLDNTEFQGK(1)R	Tip60 OE only
P38159	RBMX	30	RNA-binding motif protein, X chromosome;RNA-	112.84	ALEAVFGK(1)YGR	1.3
P38159	RBMX;RBMXL1	80	RNA-binding motif protein, X chromosome;RNA-	64.42	AIK(1)VEQATK	0.93
P38159	RBMX;RBMXL1	9	RNA-binding motif protein, X chromosome;RNA-	110.55	VEADRPGK(1)LFIGGLNTETNE	unquantifiable
P18754	RCC1	227	Regulator of chromosome condensation	56.721	LLVPK(1)CVMLK	0.75
Q9P258	RCC2	318	Protein RCC2	53.775	VAIFIEK(1)TK	0.75
Q9P258	RCC2	77	Protein RCC2	71.735	PATAGK(1)AGGAAVVITEPEHT	1.12

Q9P258	RCC2	320	Protein RCC2	60.434	TK(1)DGQILPVPNVVVR	2.01
Q15293	RCN1	81	Reticulocalbin-1	192.5	TFDQLTPDESK(1)ER	0.78
Q15293	RCN1	134	Reticulocalbin-1	109.24	DKDDK(1)ISWEEYK	0.82
Q15293	RCN1	125	Reticulocalbin-1	99.919	VWK(1)DYDR	0.96
Q15293	RCN1	86	Reticulocalbin-1	55.676	LGK(1)IVDR	1.16
Q15293	RCN1	266	Reticulocalbin-1	120.65	LDK(1)DEIR	1.21
Q15293	RCN1	296	Reticulocalbin-1	81.565	HLVYESDK(0.049)NK(0.951)	1.55
Q15293	RCN1	70	Reticulocalbin-1	64.64	EDSK(1)TFDQLTPDESK	Tip60 OE only
Q14257	RCN2	71	Reticulocalbin-2	67.035	LQAIIK(1)K	0.97
P35241	RDX	435	Radixin	81.548	IALLEEAK(1)K	0.76
P35241	RDX	400	Radixin	147.51	RAAEEAK(1)SAIAK	0.76
P35241	RDX	405	Radixin	123.79	SAIAK(1)QAADQMK	0.92
P35241	RDX	162	Radixin	90.657	VLEQHK(1)LTK	Tip60 OE only
P35241	RDX	327	Radixin	74.127	AQLENEK(1)K	unquantifiable
P46063	RECQL	193	ATP-dependent DNA helicase Q1	58.676	LIYVTPEK(1)IAK	0.79
P46063	RECQL	206	ATP-dependent DNA helicase Q1	107.9	LEK(1)AYEAR	1.13
P46063	RECQL	180	ATP-dependent DNA helicase Q1	46.797	WVHAEMVNK(1)NSELK	1.18
P46063	RECQL	78	ATP-dependent DNA helicase Q1	47.198	EDFPWSGK(1)VK	Tip60 OE only
Q00765	REEP5	29	Receptor expression-enhancing protein 5	71.379	LEAK(1)TGVNR	unquantifiable
P35251	RFC1	568	Replication factor C subunit 1	60.434	EQVAEETSGDSK(1)AR	Tip60 OE only
P35250	RFC2	192	Replication factor C subunit 2	79.659	YTK(1)LTDAQILTR	Tip60 OE only
P61586	RHOA	135	Transforming protein RhoA	105.65	MK(1)QEPVKPEEGR	1
P61586	RHOA	162	Transforming protein RhoA	112.15	IGAFGYMECSAK(1)TK	unquantifiable
P08134	RHOC	162	Rho-related GTP-binding protein RhoC	64.55	ISAFGYLECSAK(1)TK	Tip60 OE only
P61586	RHOC;RHOA	118	Rho-related GTP-binding protein RhoC;Transforn	79.974	HFCPNVPIILVGNK(1)K	1.2
Q06587	RING1;RNF2	62	E3 ubiquitin-protein ligase RING1;E3 ubiquitin-p	51.268	NTMTTK(1)ECLHR	Tip60 OE only
O75792	RNASEH2A	167	Ribonuclease H2 subunit A	65.043	AK(1)ADALYPVVSAASICAK	unquantifiable
Q9NTX7	RNF146	176	E3 ubiquitin-protein ligase RNF146	58.661	K(1)GVAGLR	unquantifiable
Q5VTR2	RNF20	256	E3 ubiquitin-protein ligase BRE1A	54.416	LQSK(1)VETAESR	Tip60 OE only
Q5VTR2	RNF20	198	E3 ubiquitin-protein ligase BRE1A	53.683	LQEK(1)VELLSR	Tip60 OE only
P13489	RNH1	287	Ribonuclease inhibitor	94.717	ESLK(1)ELSLAGNELGDEGAR	1.37
P13489	RNH1	226	Ribonuclease inhibitor	81.625	DLCGIVASK(1)ASLR	Tip60 OE only
Q9H4A4	RNPEP	446	Aminopeptidase B	80.239	AYVHEFK(1)FR	1.1
Q13464	ROCK1;ROCK2	999	Rho-associated protein kinase 1;Rho-associated p	62.107	TLK(1)TQAVNK	unquantifiable

O75695	RP2	273	Protein XRP2	107.15	EVSMK(1)AEDAQR	unquantifiable
P27694	RPA1	206	Replication protein A 70 kDa DNA-binding subun	87.323	VTNK(1)SQIR	1.17
Q96AT9	RPE	223	Ribulose-phosphate 3-epimerase	58.676	NVCSEAAQK(1)R	unquantifiable
P27635	RPL10	188	60S ribosomal protein L10	144.65	FNADEFEDMVAEK(1)R	0.92
P27635	RPL10	145	60S ribosomal protein L10	168.85	LQNK(1)EHVIEALR	0.95
P27635	RPL10	82	60S ribosomal protein L10	84.476	SCGK(1)DGFHIR	1.67
P27635	RPL10;RPL10L	208	60S ribosomal protein L10;60S ribosomal protei	94.163	GPLDK(1)WR	1.04
P27635	RPL10;RPL10L	30	60S ribosomal protein L10;60S ribosomal protei	97.602	GVPDAK(1)IR	1.08
P27635	RPL10;RPL10L	101	60S ribosomal protein L10;60S ribosomal protei	119.53	INK(1)MLSCAGADR	1.12
P27635	RPL10;RPL10L	74	60S ribosomal protein L10;60S ribosomal protei	78.496	ICANK(1)YMVK	1.23
P62906	RPL10A	156	60S ribosomal protein L10a	57.559	STIK(1)FQMK	0.85
P62906	RPL10A	212	60S ribosomal protein L10a	55.604	STMGK(1)PQR	0.96
P62906	RPL10A	152	60S ribosomal protein L10a	87.639	VDEVK(1)STIK	0.98
P62906	RPL10A	207	60S ribosomal protein L10a	46.462	ALYIK(1)STMGK	Tip60 OE only
P62906	RPL10A	196	60S ribosomal protein L10a	69.383	K(1)NWQNVR	Tip60 OE only
P62913	RPL11	85	60S ribosomal protein L11	87.308	AEEILEK(1)GLK	0.92
P62913	RPL11	169	60S ribosomal protein L11	129.7	WFQQK(1)YDGIILPGK	0.97
P62913	RPL11	159	60S ribosomal protein L11	123.69	ISK(1)EEAMR	1.04
P62913	RPL11	38	60S ribosomal protein L11	156.8	AAK(1)VLEQLTGQTPVFSK	1.05
P62913	RPL11	78	60S ribosomal protein L11	57.55	GAK(1)AEEILEK	1.09
P62913	RPL11	52	60S ribosomal protein L11	183.79	VLEQLTGQTPVFSK(1)AR	1.09
P62913	RPL11	67	60S ribosomal protein L11	75.509	NEK(1)IAVHCTVR	1.12
P62913	RPL11	154	60S ribosomal protein L11	73.665	TGCIGAK(1)HR	1.3
P62913	RPL11	8	60S ribosomal protein L11	61.353	AQDQGEK(1)ENPMR	1.41
P30050	RPL12	40	60S ribosomal protein L12	78.113	IGPLGLSPK(1)K	1.07
P30050	RPL12	54	60S ribosomal protein L12	113.5	ATGDWK(1)GLR	1.5
P30050	RPL12	48	60S ribosomal protein L12	110.08	VGDDIAK(1)ATGDWK	Tip60 OE only
P30050	RPL12	99	60S ribosomal protein L12	73.11	NIK(1)HSGNITFDEIVNIAR	Tip60 OE only
P26373	RPL13	174	60S ribosomal protein L13	153.78	VITEEEK(1)NFK	0.73
P26373	RPL13	88	60S ribosomal protein L13	66.994	VAGIHK(1)K	0.76
P26373	RPL13	123	60S ribosomal protein L13	127.84	SK(1)LILFPR	0.89
P26373	RPL13	105	60S ribosomal protein L13	119.16	NK(1)STESLQANVQR	0.9
P26373	RPL13	200	60S ribosomal protein L13	73.067	AK(1)EAAEQDVEK	Tip60 OE only
P40429	RPL13A	191	60S ribosomal protein L13a	85.533	K(0.006)IDK(0.994)YTEVLK	0.83

P40429	RPL13A	197	60S ribosomal protein L13a	123.86	YTEVLK(1)THGLLV	0.96
P40429	RPL13A	134	60S ribosomal protein L13a	106.76	K(1)FAYLGR	0.99
P40429	RPL13A	148	60S ribosomal protein L13a	128.76	LAHEVGWK(1)YQAVTATLEEK	1.05
P40429	RPL13A;RPL13AP3	159	60S ribosomal protein L13a;Putative 60S ribosor	173.32	YQAVTATLEEK(1)RK	0.99
P40429	RPL13A;RPL13AP3	125	60S ribosomal protein L13a;Putative 60S ribosor	94.114	MVVPAALK(1)VVR	Tip60 OE only
P50914	RPL14	124	60S ribosomal protein L14	90.706	IIK(1)NEVK	0.89
P50914	RPL14	137	60S ribosomal protein L14	119.27	AALLK(1)ASPK	0.93
P50914	RPL14	103	60S ribosomal protein L14	125.25	AK(1)MTDFDR	1.01
P50914	RPL14	132	60S ribosomal protein L14	134.81	KLQK(1)AALLK	1.03
P50914	RPL14	85	60S ribosomal protein L14	132.22	ADINTK(1)WAATR	1.03
P50914	RPL14	182	60S ribosomal protein L14	88.948	VPAQK(1)ATGQK	1.04
P61313	RPL15	56	60S ribosomal protein L15	132.29	AK(1)QGYVIYR	0.99
P61313	RPL15	47	60S ribosomal protein L15	44.82	PTRPDK(1)AR	unquantifiable
P18621	RPL17	49	60S ribosomal protein L17	106.91	YLK(1)DVTLQK	0.89
P18621	RPL17	27	60S ribosomal protein L17	52.683	VHFK(1)NTR	0.96
P18621	RPL17	167	60S ribosomal protein L17	89.466	EQIVPKPEEEVAQK(1)K	0.96
P18621	RPL17	37	60S ribosomal protein L17	124.74	ETAQAIK(1)GMHIR	0.96
Q07020	RPL18	19	60S ribosomal protein L18	107.47	EPK(1)SQDIYLR	0.68
Q07020	RPL18	78	60S ribosomal protein L18	135.81	ENK(1)TAVVVGTITDDVR	0.91
Q07020	RPL18	97	60S ribosomal protein L18	58.917	VQEVPK(1)LK	1.05
Q07020	RPL18	99	60S ribosomal protein L18	74.517	LK(1)VCALR	1.07
Q07020	RPL18	30	60S ribosomal protein L18	94.669	LLVK(1)LYR	1.1
Q07020	RPL18	49	60S ribosomal protein L18	129.38	TNSTFNQVVLK(1)R	Tip60 OE only
Q02543	RPL18A	11	60S ribosomal protein L18a	69.432	EYK(1)VVGR	0.8
Q02543	RPL18A	76	60S ribosomal protein L18a	128.57	VK(1)NFGIWLR	0.93
Q02543	RPL18A	70	60S ribosomal protein L18a	121.04	SSGEIVYCGQVFEK(1)SPLR	0.98
Q02543	RPL18A	41	60S ribosomal protein L18a	94.191	IFAPNHVVAK(1)SR	1.02
Q02543	RPL18A	136	60S ribosomal protein L18a	137.14	VEEIAASK(1)CR	1.2
Q02543	RPL18A	128	60S ribosomal protein L18a	70.783	AHSIQIMK(1)VEEIAASK	Tip60 OE only
P84098	RPL19	128	60S ribosomal protein L19	83.377	VK(1)GNVFK	0.84
P84098	RPL19	144	60S ribosomal protein L19	78.655	ILMEHIHK(1)LK	0.87
P84098	RPL19	46	60S ribosomal protein L19	123.98	LIK(1)DGLIIR	0.94
P84098	RPL19	190	60S ribosomal protein L19	95.815	TLSK(1)EEETK	0.95
P84098	RPL19	92	60S ribosomal protein L19	51.949	MPEK(1)VTWMR	1.39

P84098	RPL19	153	60S ribosomal protein L19	95.502	K(1)LLADQAEAR	Tip60 OE only
P84098	RPL19	80	60S ribosomal protein L19	94.95	HMGIGK(1)R	unquantifiable
P46778	RPL21	129	60S ribosomal protein L21	68.422	GTWVQLK(1)R	0.73
P35268	RPL22	80	60S ribosomal protein L22	151.22	ITVTSEVPFSK(1)R	0.86
P35268	RPL22	69	60S ribosomal protein L22	105.17	SK(1)ITVTSEVPFSK	0.98
P35268	RPL22	107	60S ribosomal protein L22	192.12	VVANSK(1)ESYELR	1.01
P35268	RPL22;RPL22L1	84	60S ribosomal protein L22;60S ribosomal protei	82.305	YLK(1)YLTK	Tip60 OE only
P62829	RPL23	113	60S ribosomal protein L23	88.155	GEMK(1)GSAITGPVAK	0.93
P62829	RPL23	43	60S ribosomal protein L23	64.297	NLYIISVK(1)GIK	1.22
P62829	RPL23	123	60S ribosomal protein L23	123.64	GSAITGPVAK(1)ECADLWPR	unquantifiable
P62750	RPL23A	123	60S ribosomal protein L23a	117.67	LYDIDVAK(1)VNTLIRPDGEK	0.83
P62750	RPL23A	14	60S ribosomal protein L23a	54.066	EAPAPPK(1)AEAK	0.86
P62750	RPL23A	134	60S ribosomal protein L23a	149.38	VNTLIRPDGEK(1)K	0.88
P62750	RPL23A	110	60S ribosomal protein L23a	139.28	HQIK(1)QAVK	1.06
P62750	RPL23A	88	60S ribosomal protein L23a	94.692	FPLTTESAMK(1)K	1.08
P62750	RPL23A	115	60S ribosomal protein L23a	71.451	K(1)LYDIDVAK	1.57
P83731	RPL24	69	60S ribosomal protein L24	61.962	GQSEEIQK(1)K	0.91
P83731	RPL24	93	60S ribosomal protein L24	179.57	AITGASLADIMAK(1)R	1.13
P83731	RPL24	2	60S ribosomal protein L24	79.693	MK(1)VELCSFSGYK	1.18
P61254	RPL26	136	60S ribosomal protein L26	61.679	YK(1)EETIEK	0.96
P61254	RPL26;RPL26L1	69	60S ribosomal protein L26;60S ribosomal protei	157.91	GQQIGK(1)VVQVYR	1.1
P61254	RPL26;RPL26L1	36	60S ribosomal protein L26;60S ribosomal protei	93.111	IMSSPLSK(1)ELR	1.12
P61254	RPL26;RPL26L1	51	60S ribosomal protein L26;60S ribosomal protei	68.371	K(1)DDEVQVVR	1.16
P61353	RPL27	128	60S ribosomal protein L27	75.915	NK(1)WFFQK	0.83
P61353	RPL27	133	60S ribosomal protein L27	85.306	WFFQK(1)LR	0.83
P61353	RPL27	93	60S ribosomal protein L27	51.727	YSVDIPLDK(1)TVVNK	0.91
P61353	RPL27	59	60S ribosomal protein L27	84.097	VTAAMGK(1)K	0.95
P61353	RPL27	98	60S ribosomal protein L27	96.253	TVVNK(1)DVFR	0.96
P46776	RPL27A	55	60S ribosomal protein L27a	68.224	YHPGYFGK(1)VGMK	Tip60 OE only
P46779	RPL28	84	60S ribosomal protein L28	73.039	TTINK(1)NAR	0.81
P46779	RPL28	47	60S ribosomal protein L28	108.7	K(1)TVGVEPAADGK	0.82
P46779	RPL28	58	60S ribosomal protein L28	95.018	TVGVEPAADGK(1)GVVVVIK	0.84
P46779	RPL28	65	60S ribosomal protein L28	116.35	GVVVVIK(1)R	0.89
P46779	RPL28	101	60S ribosomal protein L28	70.912	NK(1)YRPDLR	1

P47914	RPL29	73	60S ribosomal protein L29	77.677	AEAIK(1)ALVKPK	0.67
P47914	RPL29	134	60S ribosomal protein L29	97.203	DQTK(1)AQAAAPASVPAQAPK	0.98
P39023	RPL3	362	60S ribosomal protein L3	75.109	ALEK(1)IDLK	0.62
P39023	RPL3	300	60S ribosomal protein L3	81.431	LIK(1)NNASTDYDLSDK	0.73
P39023	RPL3	366	60S ribosomal protein L3	112.13	IDLK(1)FIDTTSK	0.83
P39023	RPL3	294	60S ribosomal protein L3	103.26	IGQGYLIK(1)DGK	0.85
P39023	RPL3	143	60S ribosomal protein L3	89.142	WQDEDGK(1)K	0.87
P39023	RPL3	385	60S ribosomal protein L3	89.296	FQTMEEK(1)K	0.88
P39023	RPL3	393	60S ribosomal protein L3	48.504	AFMGPLK(1)K	0.91
P39023	RPL3	155	60S ribosomal protein L3	126.24	K(1)YCQVIR	0.92
P39023	RPL3	349	60S ribosomal protein L3	91.069	K(1)SLLVQTK	0.96
P39023	RPL3	103	60S ribosomal protein L3	98.694	TFK(1)TVFAEHISDECK	0.96
P39023	RPL3	115	60S ribosomal protein L3	113.63	TVFAEHISDECK(1)R	1.11
P39023	RPL3	136	60S ribosomal protein L3	75.109	K(1)WQDEDGKK	Tip60 OE only
P39023	RPL3;RPL3L	373	60S ribosomal protein L3;60S ribosomal protein	96.89	FIDTTSK(1)FGHGR	0.99
P39023	RPL3;RPL3L	250	60S ribosomal protein L3;60S ribosomal protein	51.727	K(1)VACIGAWHPAR	Tip60 OE only
P62888	RPL30	9	60S ribosomal protein L30	51.066	K(1)SLESINSR	0.84
P62888	RPL30	23	60S ribosomal protein L30	71.692	LQLVMK(1)SGK	0.86
P62888	RPL30	32	60S ribosomal protein L30	111.74	YVLGYK(1)QTLK	0.98
P62888	RPL30	44	60S ribosomal protein L30	132.01	AK(1)LVILANNCPALR	Tip60 OE only
P62899	RPL31	70	60S ribosomal protein L31	106.42	LNK(1)AVWAK	0.88
P62899	RPL31	39	60S ribosomal protein L31	68.485	IHGVGFK(1)K	0.91
P62899	RPL31	55	60S ribosomal protein L31	110.38	FAMK(1)EMGTPDVR	1.01
P62899	RPL31	75	60S ribosomal protein L31	118.06	AVWAK(1)GIR	1.04
P49207	RPL34	85	60S ribosomal protein L34	144.12	AYGGSMCAK(1)CVR	0.94
P49207	RPL34	43	60S ribosomal protein L34	98.048	APK(1)SACGVCPGR	1.05
P49207	RPL34	105	60S ribosomal protein L34	52.683	IVVK(1)VLK	1.1
P49207	RPL34	62	60S ribosomal protein L34	78.113	AVRPK(1)VLMR	Tip60 OE only
P49207	RPL34	19	60S ribosomal protein L34	91.584	LSYNTASNK(1)TR	unquantifiable
P42766	RPL35	14	60S ribosomal protein L35	52.716	K(0.147)K(0.853)EELLK	0.74
P42766	RPL35	35	60S ribosomal protein L35	88.681	VAK(1)VTGGAASK	0.97
P18077	RPL35A	15	60S ribosomal protein L35a	132.72	AIFAGYK(1)R	0.96
P18077	RPL35A	95	60S ribosomal protein L35a	92.062	SNLPAK(1)AIGHR	1.03
P18077	RPL35A	45	60S ribosomal protein L35a	145.63	DETEFYLGK(1)R	1.03

P18077	RPL35A	8	60S ribosomal protein L35a	78.934	LWSK(1)AIFAGYK	1.34
Q9Y3U8	RPL36	13	60S ribosomal protein L36	80.239	YPMAVGLNK(1)GHK	0.85
Q9Y3U8	RPL36	62	60S ribosomal protein L36	67.897	AMELLK(1)VSK	1.15
P83881	RPL36A	30	60S ribosomal protein L36a	89.355	GK(1)DSLYAQGK	1.12
P83881	RPL36A;RPL36AL	6	60S ribosomal protein L36a;60S ribosomal prote	94.262	VNVPK(1)TR	0.9
P61927	RPL37	31	60S ribosomal protein L37	100.25	AYHLQK(1)STCGK	unquantifiable
P61513	RPL37A	7	60S ribosomal protein L37a	66.138	K(1)VGIVGK	1
P61513	RPL37A;RPL37AP8	44	60S ribosomal protein L37a;Putative 60S ribosor	126.07	YTCSFCGK(1)TK	1.01
P63173	RPL38	57	60S ribosomal protein L38	65.347	LK(1)QSLPPGLAVK	0.95
P36578	RPL4	14	60S ribosomal protein L4	119.45	PLISVYSEK(1)GESSGK	0.89
P36578	RPL4	333	60S ribosomal protein L4	84.498	LNPYAK(1)TMR	0.93
P36578	RPL4	353	60S ribosomal protein L4	73.951	VDK(1)AAAAAAALQAK	0.93
P36578	RPL4	165	60S ribosomal protein L4	126.24	TK(1)EAVLLLK	0.97
P36578	RPL4	364	60S ribosomal protein L4	218.18	AAAAAAALQAK(1)SDEK	1
P36578	RPL4	368	60S ribosomal protein L4	97.431	SDEK(1)AAVAGK	1.02
P36578	RPL4	259	60S ribosomal protein L4	115.86	K(1)LDELYGTWR	1.06
P36578	RPL4	239	60S ribosomal protein L4	85.672	LNILK(1)LAPGGHVGR	1.07
P36578	RPL4	20	60S ribosomal protein L4	56.225	GESSGK(1)NVTLPAVFK	1.1
P36578	RPL4	172	60S ribosomal protein L4	81.297	EAVLLLK(1)K	1.1
P36578	RPL4	175	60S ribosomal protein L4	63.419	LK(1)AWNDIK	1.11
P36578	RPL4	274	60S ribosomal protein L4	125.97	AASLK(1)SNYNLPMHK	1.13
P36578	RPL4	182	60S ribosomal protein L4	107.43	K(1)VYASQR	Tip60 OE only
P46777	RPL5	276	60S ribosomal protein L5	62.714	MSLAQK(1)K	0.8
P46777	RPL5	158	60S ribosomal protein L5	155.42	TTTGNK(1)VFGALK	0.86
P46777	RPL5	5	60S ribosomal protein L5	54.525	GFVK(1)VVK	0.91
P46777	RPL5	85	60S ribosomal protein L5	79.568	IEGDMIVCAAYAHELPK(1)YGV	0.91
P46777	RPL5	48	60S ribosomal protein L5	143.37	YNTPK(1)YR	0.91
P46777	RPL5	164	60S ribosomal protein L5	122.57	VFGALK(1)GAVDGGLSIPHSTK	0.92
P46777	RPL5	220	60S ribosomal protein L5	152.28	YLMEEDEDAYK(1)K	0.95
P46777	RPL5	197	60S ribosomal protein L5	94.616	K(1)HIMGQNVADYMR	1.02
P46777	RPL5	221	60S ribosomal protein L5	64.207	K(1)QFSQYIK	1.06
P46777	RPL5	27	60S ribosomal protein L5	113.71	EGK(1)TDYYAR	1.07
Q02878	RPL6	87	60S ribosomal protein L6	74.772	EK(1)VLATVTKPVGGDK	0.71
Q02878	RPL6	77	60S ribosomal protein L6	45.081	YSAAK(1)SK	0.76

Q02878	RPL6	210	60S ribosomal protein L6	55.452	IPK(1)HLTDAYFK	0.87
Q02878	RPL6	207	60S ribosomal protein L6	78.334	IDISNVK(1)IPK	0.89
Q02878	RPL6	251	60S ribosomal protein L6	94.122	IDQK(1)AVDSQILPK	0.9
Q02878	RPL6	192	60S ribosomal protein L6	123.84	THQK(1)FVIATSTK	0.92
Q02878	RPL6	239	60S ribosomal protein L6	164.53	EK(1)YEITEQR	0.94
Q02878	RPL6	237	60S ribosomal protein L6	158.42	HQEGEIFDTEK(1)EK	1.02
Q02878	RPL6	262	60S ribosomal protein L6	60.828	IK(1)AIPQLQGYLR	1.16
Q02878	RPL6	260	60S ribosomal protein L6	99.688	AVDSQILPK(1)IK	Tip60 OE only
Q02878	RPL6	94	60S ribosomal protein L6	67.704	VLATVTK(1)PVGGDK	Tip60 OE only
P18124	RPL7	19	60S ribosomal protein L7	90.108	EVPAVPETLK(1)K	0.77
P18124	RPL7	77	60S ribosomal protein L7	79.16	K(1)AGNFYVPAEPK	0.87
P18124	RPL7	7	60S ribosomal protein L7	101.64	MEGVEEK(0.926)K(0.074)K	0.89
P18124	RPL7	53	60S ribosomal protein L7	53.554	LIYEK(1)AK	0.9
P18124	RPL7	29	60S ribosomal protein L7	87.754	NFAELK(1)IK	0.91
P18124	RPL7	107	60S ribosomal protein L7	137.5	K(1)VLQLLR	0.96
P18124	RPL7	161	60S ribosomal protein L7	75.915	GYGK(1)INK	1.01
P18124	RPL7	40	60S ribosomal protein L7	93.593	FAQK(1)MLR	1.11
P18124	RPL7	223	60S ribosomal protein L7	59.709	K(1)TTHFVEGGDAGNR	unquantifiable
P62424	RPL7A	37	60S ribosomal protein L7a	50.088	RPK(1)NFGIGQDIQPK	0.4
P62424	RPL7A	125	60S ribosomal protein L7a	49.444	AAGK(1)GDVPTK	0.83
P62424	RPL7A	107	60S ribosomal protein L7a	45.68	YRPETK(1)QEK	0.87
P62424	RPL7A	20	60S ribosomal protein L7a	77.282	VAPAPAVVK(1)K	0.93
P62424	RPL7A	48	60S ribosomal protein L7a	148.96	NFGIGQDIQPK(1)R	1
P62424	RPL7A	212	60S ribosomal protein L7a	120.14	TCTTVAFTQVNSEDK(1)GALAK	Tip60 OE only
P62424	RPL7A	245	60S ribosomal protein L7a	94.297	HWGGNVLGPK(1)SVAR	Tip60 OE only
P62424	RPL7A	34	60S ribosomal protein L7a	69.423	VVNPLFEK(1)R	Tip60 OE only
P62917	RPL8	181	60S ribosomal protein L8	60.342	IDKPILK(1)AGR	0.86
P62917	RPL8	250	60S ribosomal protein L8	96.492	GTK(1)TVQEK	0.89
P62917	RPL8	42	60S ribosomal protein L8	116.88	HGYIK(1)GIVK	0.97
P62917	RPL8	60	60S ribosomal protein L8	87.754	GAPLAK(1)VVFR	1
P62917	RPL8	46	60S ribosomal protein L8	61.375	GIVK(1)DIIHDPGR	1.22
P32969	RPL9	28	60S ribosomal protein L9	97.473	TVIVK(1)GPR	0.99
P32969	RPL9	59	60S ribosomal protein L9	143.85	VDK(1)WWGNR	1.01
P32969	RPL9	65	60S ribosomal protein L9	66.073	K(1)ELATVR	1.02

P32969	RPL9	121	60S ribosomal protein L9	122.13	NFLGEK(1)YIR	1.05
P05388	RPLP0	77	60S acidic ribosomal protein P0	114.59	GHLENNPALEK(1)LLPHIR	0.75
P05388	RPLP0	50	60S acidic ribosomal protein P0	75.109	GK(1)AVVLMGK	1.02
P05388	RPLP0	57	60S acidic ribosomal protein P0	61.56	AVVLMGK(1)NTMMR	1.54
P05388	RPLP0;RPLP0P6	106	60S acidic ribosomal protein P0;60S acidic ribos	114.4	DMLLANK(1)VPAAAR	1.31
P05388	RPLP0;RPLP0P6	10	60S acidic ribosomal protein P0;60S acidic ribos	74.301	ATWK(1)SNYFLK	Tip60 OE only
P05387	RPLP2	25	60S acidic ribosomal protein P2	119.76	K(1)ILDSVGIEADDDR	0.99
P05387	RPLP2	94	60S acidic ribosomal protein P2	54.514	LASVPAGGAVAVSAAPGSAAP.	1.18
P04843	RPN1	559	Dolichyl-diphosphooligosaccharide--protein gly	132.88	LDAQVK(1)ELVLK	0.98
P04843	RPN1	576	Dolichyl-diphosphooligosaccharide--protein gly	54.525	LVAGK(1)LK	1.07
P04843	RPN1	524	Dolichyl-diphosphooligosaccharide--protein gly	62.362	SLETEHK(1)ALTSEIALLQSR	1.12
P04843	RPN1	587	Dolichyl-diphosphooligosaccharide--protein gly	104.43	DTYIENEK(1)LISGK	1.15
P04843	RPN1	538	Dolichyl-diphosphooligosaccharide--protein gly	72.325	LK(1)TEGSDLCDR	Tip60 OE only
P04844	RPN2	311	Dolichyl-diphosphooligosaccharide--protein gly	161.63	LEHAK(1)SVASR	1.12
P46783	RPS10	59	40S ribosomal protein S10	119.29	GYVK(1)EQFAWR	0.92
P46783	RPS10;RPS10P5	53	40S ribosomal protein S10;Putative 40S ribosom	104.21	AMQSLK(1)SR	0.86
P62280	RPS11	48	40S ribosomal protein S11	130.56	TPK(1)EAIEGTYIDK	0.74
P62280	RPS11	58	40S ribosomal protein S11	129.38	EAIEGTYIDK(1)K	0.92
P62280	RPS11	79	40S ribosomal protein S11	75.378	ILSGVVTK(1)MK	0.92
P62280	RPS11	136	40S ribosomal protein S11	90.706	PLSK(1)TVR	0.94
P62280	RPS11	144	40S ribosomal protein S11	73.208	FNVLK(1)VTK	0.99
P62280	RPS11	30	40S ribosomal protein S11	96.331	VLLGETGK(1)EK	1.2
P62280	RPS11	38	40S ribosomal protein S11	96.253	YYK(1)NIGLGFK	Tip60 OE only
P25398	RPS12	116	40S ribosomal protein S12	52.867	DYGK(1)ESQAK	1.01
P25398	RPS12	84	40S ribosomal protein S12	77.662	K(1)LGEWVGLCK	1.08
P25398	RPS12	78	40S ribosomal protein S12	44.089	LVEALCAEHQINLIK(0.995)VD	Tip60 OE only
P62277	RPS13	70	40S ribosomal protein S13	141.78	FVTGNK(1)ILR	0.84
P62277	RPS13	34	40S ribosomal protein S13	160.53	LTSDDVK(1)EQIYK	1.06
P62277	RPS13	27	40S ribosomal protein S13	76.522	SVPTWLK(1)LTSDDVK	Tip60 OE only
P62263	RPS14	61	40S ribosomal protein S14	50.127	VTGGMK(1)VK	0.89
P62263	RPS14	106	40S ribosomal protein S14	64.64	TK(1)TPGPGAQSALR	1.11
P62263	RPS14	125	40S ribosomal protein S14	41.502	SGMK(1)IGR	1.26
P62263	RPS14	96	40S ribosomal protein S14	86.624	ELGITALHIK(1)LR	1.39
P62263	RPS14	63	40S ribosomal protein S14	76.421	VK(1)ADRDESSPYAAMLAAQD	Tip60 OE only

P62841	RPS15	77	40S ribosomal protein S15	107.07	EAPPMEKPEVVK(1)THLR	1.14
P62841	RPS15	52	40S ribosomal protein S15	98.418	K(1)QHSLLK	1.19
P62841	RPS15	7	40S ribosomal protein S15	93.262	AEVEQK(1)K	Tip60 OE only
P62244	RPS15A	19	40S ribosomal protein S15a	75.213	SINNAEK(1)R	0.87
P62244	RPS15A	84	40S ribosomal protein S15a	78.655	FDVQLK(1)DLEK	Tip60 OE only
P62249	RPS16	98	40S ribosomal protein S16	132.76	ALVAYYQK(1)YVDEASK	1.06
P62249	RPS16	105	40S ribosomal protein S16	89.046	YVDEASK(1)K	1.08
P62249	RPS16	109	40S ribosomal protein S16	131.12	EIK(1)DILIQYDR	1.41
P08708	RPS17	49	40S ribosomal protein S17	65.234	NK(1)IAGYVTHLMK	0.9
P08708	RPS17	72	40S ribosomal protein S17	172.59	GISIK(1)LQEEER	0.93
P08708	RPS17	19	40S ribosomal protein S17	127.56	VIIEK(1)YYTR	0.94
P62269	RPS18	34	40S ribosomal protein S18	98.249	IAFAITAIK(1)GVGR	Tip60 OE only
P39019	RPS19	77	40S ribosomal protein S19	82.417	GGAGVGSMTK(1)IYGGR	0.78
P39019	RPS19	43	40S ribosomal protein S19	128.67	HK(1)ELAPYDENWFYTR	0.88
P39019	RPS19	38	40S ribosomal protein S19	108.47	VPEWVDTVK(1)LAK	0.88
P39019	RPS19	97	40S ribosomal protein S19	55.604	GSK(1)SVAR	0.89
P39019	RPS19	122	40S ribosomal protein S19	77.058	K(1)LTPQGQR	0.89
P39019	RPS19	115	40S ribosomal protein S19	69.954	MVEK(1)DQDGGR	0.89
P39019	RPS19	29	40S ribosomal protein S19	103.7	LK(1)VPEWVDTVK	0.92
P39019	RPS19	7	40S ribosomal protein S19	270.74	PGVTVK(1)DVNQQEFVR	0.94
P39019	RPS19	143	40S ribosomal protein S19	99.139	IAGQVAAANK(1)K	1.01
P39019	RPS19	111	40S ribosomal protein S19	93.561	VLQALEGLK(1)MVEK	Tip60 OE only
P15880	RPS2	58	40S ribosomal protein S2	115.78	AEDK(1)EWMPVTK	0.87
P15880	RPS2	238	40S ribosomal protein S2	93.206	GCTATLGNFAK(1)ATFDAISK	0.88
P15880	RPS2	211	40S ribosomal protein S2	79.16	GTGIVSAPVPK(1)K	0.99
P15880	RPS2	114	40S ribosomal protein S2	78.334	IMPVQK(1)QTR	1.02
P15880	RPS2	65	40S ribosomal protein S2	77.633	EWMPVTK(1)LGR	1.07
P15880	RPS2	263	40S ribosomal protein S2	48.848	ETVFTK(1)SPYQEFTDHLVK	1.12
P15880	RPS2	275	40S ribosomal protein S2	74.786	SPYQEFTDHLVK(1)THTR	1.15
P15880	RPS2	108	40S ribosomal protein S2	88.921	DEVLK(1)IMPVQK	1.19
P60866	RPS20	75	40S ribosomal protein S20	79.693	TPCGEGSK(1)TWDR	0.98
P60866	RPS20	34	40S ribosomal protein S20	96.89	SLEK(1)VCADLIR	1.09
P60866	RPS20	30	40S ribosomal protein S20	41.892	NVK(1)SLEK	1.18
P60866	RPS20	8	40S ribosomal protein S20	42.314	DTGK(1)TPVEPEVAIHR	1.23

P63220	RPS21	74	40S ribosomal protein S21	62.823	LAK(1)ADGIVSK	0.88
P62266	RPS23	48	40S ribosomal protein S23	76.01	ANPFGGASHAK(1)GIVLEK	0.86
P62266	RPS23	54	40S ribosomal protein S23	122.26	GIVLEK(1)VGVEAK	0.91
P62266	RPS23	37	40S ribosomal protein S23	40.968	AHLGTALK(1)ANPFGGASHAK	1.9
P62847	RPS24	122	40S ribosomal protein S24	76.827	GTAK(1)ANVGAGK	1.04
P62847	RPS24	37	40S ribosomal protein S24	81.548	ATVPK(1)TEIR	1.07
P62851	RPS25	57	40S ribosomal protein S25	68.915	ATYDK(1)LCK	1.02
P62851	RPS25	98	40S ribosomal protein S25	86.953	GLIK(1)LVSK	1.12
P62851	RPS25	43	40S ribosomal protein S25	104.79	DK(1)LNNLVLFDK	1.15
P62851	RPS25	66	40S ribosomal protein S25	67.019	EVPNYK(1)LITPAVVSER	1.17
P62851	RPS25	60	40S ribosomal protein S25	40.002	LCK(1)EVPNYK	1.27
P62851	RPS25	52	40S ribosomal protein S25	86.313	LNNLVLFDK(1)ATYDK	unquantifiable
P42677	RPS27	5	40S ribosomal protein S27	97.904	PLAK(1)DLLHPSPEEEK	0.85
P62979	RPS27A	99	Ubiquitin-40S ribosomal protein S27a;Ubiquitin	90.986	VK(1)LAVLK	0.88
P62979	RPS27A	104	Ubiquitin-40S ribosomal protein S27a;Ubiquitin	70.268	LAVLK(1)YYK	1.34
P62979	RPS27A;UBA52;UBB;	33	Ubiquitin-40S ribosomal protein S27a;Ubiquitin	122.7	IQDK(1)EGIPPDQQR	0.91
P62979	RPS27A;UBA52;UBB;	48	Ubiquitin-40S ribosomal protein S27a;Ubiquitin	180.8	LIFAGK(1)QLEDGR	0.92
P62979	RPS27A;UBA52;UBB;	11	Ubiquitin-40S ribosomal protein S27a;Ubiquitin	242.41	TLTGK(1)TITLEVEPSDTIENVK	0.93
P62979	RPS27A;UBA52;UBB;	27	Ubiquitin-40S ribosomal protein S27a;Ubiquitin	231.52	TITLEVEPSDTIENVK(1)AK	0.94
P62979	RPS27A;UBA52;UBB;	6	Ubiquitin-40S ribosomal protein S27a;Ubiquitin	150.08	MQIFVK(1)TLTGK	0.95
P62979	RPS27A;UBA52;UBB;	63	Ubiquitin-40S ribosomal protein S27a;Ubiquitin	144.33	TLSDYNIQK(1)ESTLHLVLR	1.05
P62857	RPS28	47	40S ribosomal protein S28	80.555	NVK(1)GPVR	unquantifiable
P62857	RPS28	16	40S ribosomal protein S28	128.82	VTK(1)VLGR	unquantifiable
P62273	RPS29	33	40S ribosomal protein S29	74.423	K(1)YGLNMCR	Tip60 OE only
P23396	RPS3	75	40S ribosomal protein S3	150.84	ELTAVVQK(1)R	0.81
P23396	RPS3	10	40S ribosomal protein S3	58.981	K(1)FVADGIFK	0.86
P23396	RPS3	141	40S ribosomal protein S3	74.162	GCEVVVSGK(1)LR	1.08
P23396	RPS3	132	40S ribosomal protein S3	100.55	FIMESGAK(1)GCEVVVSGK	Tip60 OE only
P61247	RPS3A	195	40S ribosomal protein S3a	50.284	LIPDSIGK(1)DIEK	0.78
P61247	RPS3A	182	40S ribosomal protein S3a	137.98	EVQTNDLK(1)EVVNK	0.79
P61247	RPS3A	240	40S ribosomal protein S3a	55.588	LMELHGEGSSSGK(1)ATGDET	0.88
P61247	RPS3A	199	40S ribosomal protein S3a	133.86	DIEK(1)ACQSIYPLHDVFVR	0.9
P61247	RPS3A	85	40S ribosomal protein S3a	128.36	FK(1)LITEDVQGK	0.93
P61247	RPS3A	109	40S ribosomal protein S3a	98.009	DK(1)MCSMVK	0.93

P61247	RPS3A	144	40S ribosomal protein S3a	105.57	LFCVGFTK(1)K	0.95
P61247	RPS3A	56	40S ribosomal protein S3a	112.85	TQGTK(1)IASDGLK	1.02
P61247	RPS3A	46	40S ribosomal protein S3a	52.255	NIGK(1)TLVTR	1.03
P61247	RPS3A	63	40S ribosomal protein S3a	166.17	IASDGLK(1)GR	1.04
P61247	RPS3A	249	40S ribosomal protein S3a	140.49	ATGDETGAK(1)VER	1.07
P61247	RPS3A	27	40S ribosomal protein S3a	98.299	VVDPFSK(1)K	1.12
P61247	RPS3A	167	40S ribosomal protein S3a	118.74	K(1)MMEIMTR	1.13
P61247	RPS3A	227	40S ribosomal protein S3a	51.566	FELGK(1)LMELHGEGSSSGK	1.15
P61247	RPS3A	152	40S ribosomal protein S3a	141.48	K(1)TSYAQHQQVR	1.19
P61247	RPS3A	94	40S ribosomal protein S3a	40.012	LITEDVQGK(1)NCLTNFHGMD	Tip60 OE only
P62701	RPS4X	120	40S ribosomal protein S4, X isoform	77.923	ITPEEAK(1)YK	0.96
P62701	RPS4X	128	40S ribosomal protein S4, X isoform	75.189	K(1)IFVGTK	0.98
P62701	RPS4X	106	40S ribosomal protein S4, X isoform	131.04	LIYDTK(1)GR	1.06
P62701	RPS4X	134	40S ribosomal protein S4, X isoform	60.317	IFVGTK(1)GIPHLVTHDAR	1.14
P62701	RPS4X;RPS4Y2;RPS4Y	53	40S ribosomal protein S4, X isoform;40S riboson	114.4	LK(1)YALTGDEVK	0.86
P62701	RPS4X;RPS4Y2;RPS4Y	62	40S ribosomal protein S4, X isoform;40S riboson	102.61	YALTGDEVK(1)K	1.08
P62701	RPS4X;RPS4Y2;RPS4Y	16	40S ribosomal protein S4, X isoform;40S riboson	69.451	VAAPK(1)HWMLDK	1.16
P62753	RPS6	149	40S ribosomal protein S6	142.92	LFNLSK(1)EDDVR	0.81
P62081	RPS7	178	40S ribosomal protein S7	99.802	VETFSGVYK(1)K	0.75
P62081	RPS7	155	40S ribosomal protein S7	53.493	LIK(1)VHLDK	1.13
P62081	RPS7	90	40S ribosomal protein S7	62.088	FSGK(1)HVVFIAQR	1.18
P62081	RPS7	160	40S ribosomal protein S7	43.761	VHLDK(1)AQQNNVEHK	Tip60 OE only
P62241	RPS8	139	40S ribosomal protein S8	123.63	LTPEEEEILNK(1)K	0.88
P62241	RPS8	128	40S ribosomal protein S8	100.55	GAK(1)LTPEEEEILNK	1
P62241	RPS8	157	40S ribosomal protein S8	81.619	NAK(1)ISSLLEEQFQQGK	unquantifiable
P46781	RPS9	91	40S ribosomal protein S9	109.71	IGVLDEGK(1)MK	0.89
P46781	RPS9	22	40S ribosomal protein S9	78.516	RPFEK(1)SR	0.97
P46781	RPS9	155	40S ribosomal protein S9	123.95	LDSQK(1)HIDFSLR	0.99
P46781	RPS9	116	40S ribosomal protein S9	82.008	LQTQVFK(1)LGLAK	1
P46781	RPS9	121	40S ribosomal protein S9	86.624	LGLAK(1)SIHHAR	1.03
P46781	RPS9	52	40S ribosomal protein S9	92.692	FTLAK(1)IR	1.03
P46781	RPS9	93	40S ribosomal protein S9	58.676	MK(1)LDYILGLK	1.05
P46781	RPS9	139	40S ribosomal protein S9	65.627	K(1)QVVNIPSFIVR	1.1
P46781	RPS9	47	40S ribosomal protein S9	84.097	VK(1)FTLAK	1.22

P46781	RPS9	66	40S ribosomal protein S9	131.44	ELLTLDEK(1)DPR	Tip60 OE only
P08865	RPSA	220	40S ribosomal protein SA	100.88	EEQAAAEK(1)AVTK	0.8
P08865	RPSA	212	40S ribosomal protein SA	123.64	DPEEIEK(1)EEQAAAEK	0.9
P08865	RPSA	89	40S ribosomal protein SA	101.6	AVLK(1)FAAATGATPIAGR	0.96
P08865	RPSA	52	40S ribosomal protein SA	131.22	SDGIYIINLK(1)R	1.09
P08865	RPSA	11	40S ribosomal protein SA	134.98	SGALDVLQMK(1)EEDVLK	Tip60 OE only
P08865	RPSA	42	40S ribosomal protein SA	143.39	K(1)SDGIYIINLK	Tip60 OE only
Q9P2E9	RRBP1	720	Ribosome-binding protein 1	149.38	LLATEQEDAAVAK(1)SK	1.17
P23921	RRM1	376	Ribonucleoside-diphosphate reductase large sub	194.2	LYASYEK(1)QGR	0.81
P23921	RRM1	471	Ribonucleoside-diphosphate reductase large sub	76.064	LAEVTK(1)VVVR	0.84
P23921	RRM1	128	Ribonucleoside-diphosphate reductase large sub	88.056	STLDIVLANK(1)DR	Tip60 OE only
P31350	RRM2	217	Ribonucleoside-diphosphate reductase subunit I	42.001	WIGDK(1)EATYGER	Tip60 OE only
Q5JTH9	RRP12	599	RRP12-like protein	116.51	SK(1)AMDLAQAGSTVESK	0.98
Q5JTH9	RRP12	671	RRP12-like protein	124.1	TLITK(1)GCQAEADR	1.19
Q15050	RRS1	20	Ribosome biogenesis regulatory protein homolo	55.064	AEQDEAEK(1)LQR	Tip60 OE only
Q15050	RRS1	265	Ribosome biogenesis regulatory protein homolo	57.492	FQPLFGDFAAEK(1)K	unquantifiable
O76021	RSL1D1	240	Ribosomal L1 domain-containing protein 1	72.879	GLSEK(1)LPEK	0.93
O76021	RSL1D1	244	Ribosomal L1 domain-containing protein 1	117.53	LPEK(1)WESVK	0.93
O76021	RSL1D1	136	Ribosomal L1 domain-containing protein 1	127.42	TVSQIISLQTLK(1)K	1
O76021	RSL1D1	48	Ribosomal L1 domain-containing protein 1	74.944	AVDALLTHCK(1)SR	1.01
O76021	RSL1D1	76	Ribosomal L1 domain-containing protein 1	50.632	IPSK(1)ELR	1.03
O76021	RSL1D1	140	Ribosomal L1 domain-containing protein 1	72.492	EYK(1)SYEAK	1.15
O76021	RSL1D1	385	Ribosomal L1 domain-containing protein 1	72.434	VEIQK(1)HATGK	1.2
O76021	RSL1D1	124	Ribosomal L1 domain-containing protein 1	73.466	HGIK(1)TVSQIISLQTLK	2.11
O76021	RSL1D1	109	Ribosomal L1 domain-containing protein 1	77.062	DEPNSTPEK(1)TEQFYR	Tip60 OE only
Q9Y3I0	RTCB	366	tRNA-splicing ligase RtcB homolog	88.338	VEQHVVDGK(1)ER	1.12
O95197	RTN3	1018	Reticulon-3	69.198	SIVEK(1)IQAK	unquantifiable
O95197	RTN3	1022	Reticulon-3	73.248	IQAK(1)LPGIAK	unquantifiable
O95197	RTN3	905	Reticulon-3	81.992	SVIQAVQK(1)SEEGHPFK	unquantifiable
O95197	RTN3	998	Reticulon-3	142.1	YK(1)TQIDHYVGIAR	unquantifiable
Q9NQC3	RTN4	1179	Reticulon-4	50.703	DAMAK(1)IQAK	Tip60 OE only
Q9Y265	RUVBL1	274	RuvB-like 1	49.85	KTEITDK(1)LR	0.49
Q9Y265	RUVBL1	162	RuvB-like 1	107.06	TISHVIIGLK(1)TAK	0.94
Q9Y265	RUVBL1	445	RuvB-like 1	74.944	SSAK(1)ILADQQDK	0.98

Q9Y265	RUVBL1	182	RuvB-like 1	136.48	LDPSIFESLQK(1)ER	Tip60 OE only
Q9Y230	RUVBL2	197	RuvB-like 2	94.122	VQAGDVITIDK(1)ATGK	0.59
Q9Y230	RUVBL2	365	RuvB-like 2	124.12	LLIVSTTPYSEK(1)DTK	0.91
P60903	S100A10	28	Protein S100-A10	133.12	GYLTK(1)EDLR	0.85
P60903	S100A10	23	Protein S100-A10	107.15	FAGDK(1)GYLTK	0.85
P60903	S100A10	54	Protein S100-A10	73.233	DPLAVDK(1)IMK	0.91
P60903	S100A10	57	Protein S100-A10	136.75	IMK(1)DLDQCR	1.1
P31949	S100A11	27	Protein S100-A11;Protein S100-A11, N-terminall	46.73	YAGK(1)DGYNYTLSK	Tip60 OE only
Q9UBE0	SAE1	183	SUMO-activating enzyme subunit 1;SUMO-activa	138.45	VAK(1)VSQGVEDGPDTK	Tip60 OE only
Q9UBE0	SAE1	141	SUMO-activating enzyme subunit 1;SUMO-activa	60.49	DVIVK(1)VDQICHK	Tip60 OE only
Q9UBE0	SAE1	32	SUMO-activating enzyme subunit 1;SUMO-activa	86.833	LWGLEAQK(1)R	unquantifiable
Q15424	SAFB;SAFB2	436	Scaffold attachment factor B1;Scaffold attachme	51.445	VVGAK(1)VVTNAR	1.04
Q9Y512	SAMM50	74	Sorting and assembly machinery component 50 I	71.692	AK(1)NLIEVMR	0.74
Q9NR31	SAR1A	146	GTP-binding protein SAR1a	118.26	TDAISEEK(1)LR	0.94
Q9NR31	SAR1A	27	GTP-binding protein SAR1a	43.68	SGK(1)LVFLGLDNAGK	Tip60 OE only
P82979	SARNP	12	SAP domain-containing ribonucleoprotein	77.741	LK(1)LAELK	0.76
P82979	SARNP	17	SAP domain-containing ribonucleoprotein	191.85	LAELK(1)QECLAR	0.79
P82979	SARNP	31	SAP domain-containing ribonucleoprotein	116.05	GIK(1)QDLIHR	0.87
Q14108	SCARB2	129	Lysosome membrane protein 2	101.93	DQSVGDPK(1)IDLIR	unquantifiable
O00560	SDCBP	119	Syntenin-1	87.806	EVILCK(1)DQDGK	1.11
P31040	SDHA	538	Succinate dehydrogenase [ubiquinone] flavoprot	103.34	VGSVLQEGCGK(1)ISK	0.93
P31040	SDHA	250	Succinate dehydrogenase [ubiquinone] flavoprot	94.297	AK(1)NTVVATGGYGR	1.15
P31040	SDHA	550	Succinate dehydrogenase [ubiquinone] flavoprot	48.504	HLK(1)TFDR	1.38
P55735	SEC13	38	Protein SEC13 homolog	67.169	SVK(1)IFDVR	1.32
Q9H9S3	SEC61A2;SEC61A1	107	Protein transport protein Sec61 subunit alpha is	120.68	IIEVGDTPK(1)DR	0.81
Q99442	SEC62	60	Translocation protein SEC62	92.538	AVDCLLDSK(1)WAK	unquantifiable
Q9UGP8	SEC63	631	Translocation protein SEC63 homolog	63.48	ALLETK(1)SK	0.9
Q9UGP8	SEC63	416	Translocation protein SEC63 homolog	190.15	TIQDLVSLK(1)ESDR	Tip60 OE only
Q9NVA2	SEPT11;SEPT6;SEPT8	175	Septin-11;Septin-6;Septin-8	70.598	K(0.069)LDSK(0.931)VNIIPII	Tip60 OE only
Q9NVA2	SEPT11;SEPT6;SEPT8	170	Septin-11;Septin-6;Septin-8	57.96	SLDLVTMK(1)K	unquantifiable
Q9NVA2	SEPTIN11	190	Septin-11	54.608	ADTIAK(1)NELHK	0.9
Q9NVA2	SEPTIN11	366	Septin-11	99.257	ELHEK(1)FDLLK	Tip60 OE only
Q15019	SEPTIN2	190	Septin-2	124.23	ADTLTLK(1)ER	0.94
Q15019	SEPTIN2	74	Septin-2	81.338	VIPGAAEK(1)IER	0.98

Q15019	SEPTIN2	174	Septin-2	122.26	AIHNK(1)VNIVPVIAK	1
Q16181	SEPTIN2	219	Septin-7	60.76	EIQEHK(1)IK	0.99
Q16181	SEPTIN2	208	Septin-7	143.89	ADTLTPEECQQFK(1)K	1.12
Q16181	SEPTIN2	373	Septin-7	95.608	LK(1)DSEAELQR	1.17
Q9UHD8	SEPTIN9	490	Septin-9	100.07	LVNEK(1)FR	0.92
Q8NC51	SERBP1	102	Plasminogen activator inhibitor 1 RNA-binding p	51.193	EETQPPVALK(1)K	0.84
Q8NC51	SERBP1	303	Plasminogen activator inhibitor 1 RNA-binding p	111.65	AK(1)VEFNIR	unquantifiable
P35237	SERPINB6	246	Serpin B6	53.683	TVEK(1)ELTYEK	unquantifiable
P50454	SERPINH1	332	Serpin H1	135.66	HLAGLGLTEAIDK(1)NK	0.6
P50454	SERPINH1	250	Serpin H1	84.658	TGLYNYYDDEK(1)EK	0.89
P50454	SERPINH1	291	Serpin H1	63.486	EQLK(1)IWMGK	0.92
P50454	SERPINH1	300	Serpin H1	76.064	K(1)AVAISLPK	0.95
P50454	SERPINH1	39	Serpin H1	58.699	LSPK(1)AATLAER	1
P50454	SERPINH1	252	Serpin H1	86.699	EK(1)LQIVEMPLAHK	1.03
P50454	SERPINH1	94	Serpin H1	96.464	ATTASQAK(1)AVLSAEQLR	1.03
P50454	SERPINH1	334	Serpin H1	170.5	NK(1)ADLSR	1.05
Q01105	SET	167	Protein SET	84.895	EFHLNESGDPSSK(1)STEIK	0.94
Q01105	SET	172	Protein SET	77.748	STEIK(1)WK	1.04
Q01105	SET	154	Protein SET	148.72	VLSK(1)EFHLNESGDPSSK	1.05
Q01105	SET;SETSIP	72	Protein SET;Protein SETSIP	88.35	VEQK(1)YNK	0.86
Q01105	SET;SETSIP	132	Protein SET;Protein SETSIP	232	VEVTEFEDIK(1)SGYR	0.95
Q15637	SF1	306	Splicing factor 1	46.41	FQRPGDPQSAQDK(1)AR	1.08
Q15459	SF3A1	743	Splicing factor 3A subunit 1	53.001	VK(1)IHEATGMPAGK	1
Q15459	SF3A1	251	Splicing factor 3A subunit 1	90.629	VEWAK(1)FQER	Tip60 OE only
Q15459	SF3A1	55	Splicing factor 3A subunit 1	69.01	NIVDK(1)TASFVAR	Tip60 OE only
Q15428	SF3A2	91	Splicing factor 3A subunit 2	73.067	EAK(1)EAPAQPAPEK	Tip60 OE only
Q12874	SF3A3	64	Splicing factor 3A subunit 3	57.859	DLYDDK(1)DGLR	0.7
Q12874	SF3A3	153	Splicing factor 3A subunit 3	61.679	YINLK(1)ASEK	0.87
Q12874	SF3A3	264	Splicing factor 3A subunit 3	110.76	LK(1)SALLALGLK	0.96
O75533	SF3B1	562	Splicing factor 3B subunit 1	77.677	ILYK(1)LDDLVR	0.76
O75533	SF3B1	963	Splicing factor 3B subunit 1	143.89	TAVVMK(1)TCQEEK	1.15
O75533	SF3B1	858	Splicing factor 3B subunit 1	110.39	IVDDLK(1)DEAEQYR	1.2
Q15393	SF3B3	965	Splicing factor 3B subunit 3	86.794	VLIGVGK(1)LLR	1.01
Q15393	SF3B3	126	Splicing factor 3B subunit 3	78.342	IVPGQFLAVDPK(1)GR	1.07

Q15393	SF3B3	137	Splicing factor 3B subunit 3	151.97	AVMISAIEK(1)QK	1.18
Q15393	SF3B3	109	Splicing factor 3B subunit 3	85.377	IHQETFGK(1)SGCR	1.32
Q15393	SF3B3	546	Splicing factor 3B subunit 3	114.93	TIVK(1)CAVNQR	unquantifiable
Q15393	SF3B3	974	Splicing factor 3B subunit 3	50.966	VYDLGK(1)K	unquantifiable
P23246	SFPQ	421	Splicing factor, proline- and glutamine-rich	105.4	GIVEFASK(1)PAAR	0.33
P23246	SFPQ	510	Splicing factor, proline- and glutamine-rich	79.597	EQVEK(1)NMK	0.86
P23246	SFPQ	279	Splicing factor, proline- and glutamine-rich	126.1	ISDSEGFK(1)ANLSLLR	0.88
P23246	SFPQ	466	Splicing factor, proline- and glutamine-rich	62.464	LAQK(1)NPMYQK	0.89
P23246	SFPQ	332	Splicing factor, proline- and glutamine-rich	60.648	GK(1)GFGFIK	1
P23246	SFPQ	502	Splicing factor, proline- and glutamine-rich	205.7	SLDEMEK(1)QQR	1
P23246	SFPQ	559	Splicing factor, proline- and glutamine-rich	167.3	MEELHNQEMQK(1)R	1.02
P23246	SFPQ	413	Splicing factor, proline- and glutamine-rich	51.286	STGK(1)GIVEFASK	1.03
P23246	SFPQ	518	Splicing factor, proline- and glutamine-rich	54.225	DK(1)LESEMEDAYHEHQANLL	1.07
P23246	SFPQ	516	Splicing factor, proline- and glutamine-rich	65.438	DAK(0.934)DK(0.066)LESEM	1.08
P23246	SFPQ	472	Splicing factor, proline- and glutamine-rich	149.4	NPMYQK(1)ER	1.09
P23246	SFPQ	338	Splicing factor, proline- and glutamine-rich	126.41	GFGFIK(1)LESR	1.4
P23246	SFPQ	319	Splicing factor, proline- and glutamine-rich	89.561	LFAK(1)YGEPGEVFINK	Tip60 OE only
Q9H9B4	SFXN1	223	Sideroflexin-1	112.15	LGESANAAK(1)QAITQVVVSR	unquantifiable
Q9H299	SH3BGRL3	18	SH3 domain-binding glutamic acid-rich-like prot	76.465	EIK(1)SQQSEVTR	unquantifiable
Q99961	SH3GL1	20	Endophilin-A2	109.22	ASQLVSEK(1)VGGAEGTK	1.15
Q9NR46	SH3GLB2	123	Endophilin-B2	46.796	VAEAEK(1)QLGAAER	1.37
P34897	SHMT2	297	Serine hydroxymethyltransferase, mitochondrial	47.443	GVK(1)AVDPK	0.86
P34897	SHMT2	200	Serine hydroxymethyltransferase, mitochondrial	109.84	LNPK(1)TGLIDYNQLALTAR	0.91
P34897	SHMT2	474	Serine hydroxymethyltransferase, mitochondrial	125.75	SFLLK(1)DSETSQR	0.93
P34897	SHMT2	280	Serine hydroxymethyltransferase, mitochondrial	77.192	HADIVTTTTHK(1)TLR	0.95
P34897	SHMT2	464	Serine hydroxymethyltransferase, mitochondrial	94.302	TAK(1)LQDFK	0.96
P34897	SHMT2	302	Serine hydroxymethyltransferase, mitochondrial	54.157	AVDPK(1)TGR	1.08
P34897	SHMT2	103	Serine hydroxymethyltransferase, mitochondrial	98.997	YSEGYPGK(1)R	1.13
P42285	SKIV2L2	1029	Superkiller viralicidic activity 2-like 2	69.423	FAEGITK(1)IK	0.7
P63208	SKP1	130	S-phase kinase-associated protein 1	126.67	GK(1)TPEEIR	1.17
P55011	SLC12A2	1061	Solute carrier family 12 member 2	47.849	VFIGGK(1)INR	unquantifiable
P53985	SLC16A1	450	Monocarboxylate transporter 1	58.981	LLAK(1)EQK	unquantifiable
Q02978	SLC25A11	57	Mitochondrial 2-oxoglutarate/malate carrier prc	117.01	MQLSGEGAK(1)TR	0.78
Q6NUK1	SLC25A24	285	Calcium-binding mitochondrial carrier protein S	181.31	LLTEEGQK(1)IGTFER	unquantifiable

Q00325	SLC25A3	313	Phosphate carrier protein, mitochondrial	79.597	GVWK(1)GLFAR	0.87
Q00325	SLC25A3	295	Phosphate carrier protein, mitochondrial	95.573	EK(1)GSSASLVLK	0.91
Q00325	SLC25A3	206	Phosphate carrier protein, mitochondrial	50.632	DAAPK(1)MYK	0.95
Q00325	SLC25A3	209	Phosphate carrier protein, mitochondrial	100.19	MYK(1)EEGLK	1.04
Q00325	SLC25A3	304	Phosphate carrier protein, mitochondrial	89.355	GSSASLVLK(1)R	1.1
Q00325	SLC25A3	99	Phosphate carrier protein, mitochondrial	60.973	MQVDPQK(1)YK	1.12
Q00325	SLC25A3	214	Phosphate carrier protein, mitochondrial	43.246	EEGLK(1)AFYK	Tip60 OE only
P12235	SLC25A4	263	ADP/ATP translocase 1	52.403	IAK(1)DEGAK	0.8
P12235	SLC25A4	147	ADP/ATP translocase 1	118.18	LAADVGK(1)GAAQR	0.8
P12235	SLC25A4	166	ADP/ATP translocase 1	89.142	IFK(1)SDGLR	0.82
P05141	SLC25A5	163	ADP/ATP translocase 2;ADP/ATP translocase 2, N	87.639	GLGDCLVK(1)IYK	0.8
P05141	SLC25A5	166	ADP/ATP translocase 2;ADP/ATP translocase 2, N	98.523	IYK(1)SDGIK	0.8
P05141	SLC25A5	147	ADP/ATP translocase 2;ADP/ATP translocase 2, N	157.75	LAADVGK(1)AGAER	0.8
P05141	SLC25A5	10	ADP/ATP translocase 2;ADP/ATP translocase 2, N	180.74	TDAAVSFAK(1)DFLAGGVAAA	0.93
P05141	SLC25A5	43	ADP/ATP translocase 2;ADP/ATP translocase 2, N	231.06	LLLQVQHASK(1)QITADK	0.96
P05141	SLC25A5;SLC25A4;Sl	33	ADP/ATP translocase 2;ADP/ATP translocase 2, N	156.16	VK(1)LLLQVQHASK	0.89
P05141	SLC25A5;SLC25A6	96	ADP/ATP translocase 2;ADP/ATP translocase 2, N	136.63	YK(1)QIFLGGVDK	0.91
P05141	SLC25A5;SLC25A6	268	ADP/ATP translocase 2;ADP/ATP translocase 2, N	77.058	DEGGK(1)AFFK	0.95
P05141	SLC25A5;SLC25A6	63	ADP/ATP translocase 2;ADP/ATP translocase 2, N	234.67	IPK(1)EQGVLSFWR	0.99
P12236	SLC25A6	147	ADP/ATP translocase 3;ADP/ATP translocase 3, N	133.23	LAADVGK(1)SGTER	0.91
P12236	SLC25A6	43	ADP/ATP translocase 3;ADP/ATP translocase 3, N	144.29	LLLQVQHASK(1)QIAADK	1.02
P08195	SLC3A2	147	4F2 cell-surface antigen heavy chain	95.018	IK(1)VAEDEAEAAAAAK	0.83
P08195	SLC3A2	160	4F2 cell-surface antigen heavy chain	91.076	VAEDEAEAAAAAK(1)FTGLSK	0.85
P08195	SLC3A2	257	4F2 cell-surface antigen heavy chain	47.559	VK(1)GLVLGPIHK	0.88
P08195	SLC3A2	245	4F2 cell-surface antigen heavy chain	112.74	IGDLQAFQGHGAGNLAGLK(1	0.96
P08195	SLC3A2	166	4F2 cell-surface antigen heavy chain	96.665	FTGLSK(1)EELLK	1.01
P08195	SLC3A2	298	4F2 cell-surface antigen heavy chain	108.67	EDFDSLLQSAK(1)K	1.05
P08195	SLC3A2	171	4F2 cell-surface antigen heavy chain	90.685	EELLK(1)VAGSPGWVR	1.33
P08195	SLC3A2	255	4F2 cell-surface antigen heavy chain	77.677	LDYLSSLK(1)VK	Tip60 OE only
Q01650	SLC7A5	19	Large neutral amino acids transporter small subu	115.18	ALAAPAAEEK(1)EEAR	0.88
Q9H2G2	SLK	1106	STE20-like serine/threonine-protein kinase	48.283	IK(1)QFAAQEEK	1.16
O00193	SMAP	62	Small acidic protein	110.31	LVIGDHK(1)STSHFR	0.96
P51532	SMARCA4	399	Transcription activator BRG1	74.301	TK(1)ATIELK	0.91
O60264	SMARCA5	600	SWI/SNF-related matrix-associated actin-depend	74.127	IGQTK(1)TVR	0.81

O60264	SMARCA5	834	SWI/SNF-related matrix-associated actin-depend	172.99	IDEAESLNDEELEEK(1)EK	1.01
O60264	SMARCA5	966	SWI/SNF-related matrix-associated actin-depend	104.79	LGFDK(1)ENVYDELR	1.05
O60264	SMARCA5	924	SWI/SNF-related matrix-associated actin-depend	77.058	ALDTK(1)IGR	1.29
O60264	SMARCA5;SMARCA1	319	SWI/SNF-related matrix-associated actin-depend	61.999	SK(1)LSEIVR	1.06
Q92922	SMARCC1;SMARCC2	910	SWI/SNF complex subunit SMARCC1;SWI/SNF co	60.76	KLEIK(1)LR	1.11
Q92922	SMARCC1;SMARCC2	882	SWI/SNF complex subunit SMARCC1;SWI/SNF co	102.06	AK(1)HLAAVEER	Tip60 OE only
Q14683	SMC1A	287	Structural maintenance of chromosomes proteir	55.531	EK(1)DSELNQK	0.84
Q14683	SMC1A	1037	Structural maintenance of chromosomes proteir	111.63	DK(1)FQETSDEFEAAR	0.84
Q14683	SMC1A	723	Structural maintenance of chromosomes proteir	152.54	YSQSDLEQTK(1)TR	0.89
Q14683	SMC1A	1030	Structural maintenance of chromosomes proteir	50.04	AMEK(1)LESVR	0.94
Q14683	SMC1A	129	Structural maintenance of chromosomes proteir	73.039	LGILIK(1)AR	1
Q14683	SMC1A	245	Structural maintenance of chromosomes proteir	50.966	ELASK(1)NK	1.08
Q14683	SMC1A	395	Structural maintenance of chromosomes proteir	110.73	AATLAQELEK(1)FNR	Tip60 OE only
Q14683	SMC1A	446	Structural maintenance of chromosomes proteir	102.66	LEEYITTSK(1)QSLEEQK	Tip60 OE only
O95347	SMC2	890	Structural maintenance of chromosomes proteir	142.83	EVITAQDTVIK(1)AK	0.78
O95347	SMC2	930	Structural maintenance of chromosomes proteir	53.683	EAEDGAAK(1)VSK	0.88
O95347	SMC2	1014	Structural maintenance of chromosomes proteir	75.78	SK(1)ILTTIEDLDQK	0.94
O95347	SMC2	691	Structural maintenance of chromosomes proteir	111.04	IK(1)ENELR	0.95
O95347	SMC2	877	Structural maintenance of chromosomes proteir	124.98	AQEEVTK(1)QK	0.96
O95347	SMC2	348	Structural maintenance of chromosomes proteir	67.032	TLAAK(1)EK	0.99
O95347	SMC2	275	Structural maintenance of chromosomes proteir	48.532	IK(1)ALNHEIEELEK	1
O95347	SMC2	1026	Structural maintenance of chromosomes proteir	157.91	K(1)NQALNIAWQK	1.08
O95347	SMC2	732	Structural maintenance of chromosomes proteir	113.47	TEEADLLQTK(1)LQQSSYHK	1.13
O95347	SMC2	1012	Structural maintenance of chromosomes proteir	72.485	IVENDK(1)SK	1.38
O95347	SMC2	209	Structural maintenance of chromosomes proteir	96.103	TILEEEITPTIQK(1)LK	1.82
O95347	SMC2	892	Structural maintenance of chromosomes proteir	58.286	AK(1)YAEVAK	Tip60 OE only
O95347	SMC2	433	Structural maintenance of chromosomes proteir	46.37	NK(1)QAEVK	Tip60 OE only
O95347	SMC2;MCPH1;CCDC:	976	Structural maintenance of chromosomes proteir	47.849	LQEMK(1)EK	1.32
Q9UQE7	SMC3	266	Structural maintenance of chromosomes proteir	53.775	DK(1)MEDIER	0.93
Q9UQE7	SMC3	245	Structural maintenance of chromosomes proteir	126.67	LDELSAK(1)R	1.1
Q9UQE7	SMC3	486	Structural maintenance of chromosomes proteir	232.79	EENAEQQALAAK(1)R	1.14
Q9UQE7	SMC3	288	Structural maintenance of chromosomes proteir	133.12	EEK(1)EQLSAER	1.2
Q9UQE7	SMC3	445	Structural maintenance of chromosomes proteir	86.882	LDQDLNEVK(1)AR	1.22
Q9UQE7	SMC3	997	Structural maintenance of chromosomes proteir	113.22	ALDQFVNFSEQK(1)EK	1.26

Q9UQE7	SMC3	629	Structural maintenance of chromosomes proteir	70.256	HVFGK(1)TLICR	1.4
Q9UQE7	SMC3	756	Structural maintenance of chromosomes proteir	62.978	TFMPK(1)QR	Tip60 OE only
Q9UQE7	SMC3	378	Structural maintenance of chromosomes proteir	64.567	TDLYAK(1)QGR	Tip60 OE only
Q9NTJ3	SMC4	1099	Structural maintenance of chromosomes proteir	71.501	K(0.139)K(0.861)EELYLQR	0.96
Q9NTJ3	SMC4	968	Structural maintenance of chromosomes proteir	63.283	SLEDK(1)AAEVVK	1.07
Q9NTJ3	SMC4	522	Structural maintenance of chromosomes proteir	154.66	HNTAVSQLTK(1)AK	1.08
Q9NTJ3	SMC4	1015	Structural maintenance of chromosomes proteir	79.469	DALSIK(1)LK	1.27
Q9NTJ3	SMC4	341	Structural maintenance of chromosomes proteir	85.744	IAEMETQK(1)EK	1.34
Q9NTJ3	SMC4	354	Structural maintenance of chromosomes proteir	107.85	EINEK(1)SNILSNEMK	Tip60 OE only
Q9NTJ3	SMC4	607	Structural maintenance of chromosomes proteir	85.731	VLDAIIQEK(1)K	unquantifiable
Q6IN85	SMEK1;SMEK2	566	Serine/threonine-protein phosphatase 4 regulatc	68.735	IIGLK(1)DEFYNR	unquantifiable
P52788	SMS	98	Spermine synthase	71.349	MK(1)ELSQDSTGR	0.81
P52788	SMS	307	Spermine synthase	70.912	VLK(1)QDGK	0.96
P52788	SMS	186	Spermine synthase	116.23	AIMGSGK(1)EDYTGK	1.02
Q2TAY7	SMU1	287	WD40 repeat-containing protein SMU1;WD40 re	76.655	DTEMLATGAQDGK(1)IK	0.6
Q2TAY7	SMU1	214	WD40 repeat-containing protein SMU1;WD40 re	67.08	FGQK(1)SHVECAR	0.96
P37840	SNCA	23	Alpha-synuclein	44.863	TK(1)QGVAEAAGK	unquantifiable
P37840	SNCA	12	Alpha-synuclein	72.652	AK(1)EGVVAAAEK	unquantifiable
Q7KZF4	SND1	157	Staphylococcal nuclease domain-containing pro	63.076	LSECEEQAK(1)AAK	0.92
Q7KZF4	SND1	513	Staphylococcal nuclease domain-containing pro	134.68	VADISGDTQK(1)AK	1.02
Q7KZF4	SND1	641	Staphylococcal nuclease domain-containing pro	89.301	SSYYK(1)SLLSAEEAAK	1.15
Q7KZF4	SND1	341	Staphylococcal nuclease domain-containing pro	76.847	DK(1)QFVAK	Tip60 OE only
Q7KZF4	SND1	339	Staphylococcal nuclease domain-containing pro	112.15	DYVAPTANLDQK(1)DK	Tip60 OE only
O75643	SNRNP200	770	U5 small nuclear ribonucleoprotein 200 kDa heli	123.63	TEAEQCK(1)NLELK	0.9
O75643	SNRNP200	1421	U5 small nuclear ribonucleoprotein 200 kDa heli	100.55	LLGK(1)GNIIISTPEK	1.04
Q96DI7	SNRNP40	270	U5 small nuclear ribonucleoprotein 40 kDa prote	60.961	PFAPK(1)ER	unquantifiable
P08621	SNRNP70	118	U1 small nuclear ribonucleoprotein 70 kDa	155.42	VNYDTTESK(1)LR	0.98
P09012	SNRPA	88	U1 small nuclear ribonucleoprotein A	82.171	IQYAK(1)TDSDIIAK	1.03
P09012	SNRPA	98	U1 small nuclear ribonucleoprotein A	81.548	MK(1)GTFVER	1.13
P09661	SNRPA1	149	U2 small nuclear ribonucleoprotein A'	54.549	VLDFQK(1)VK	0.58
P08579	SNRPB2	85	U2 small nuclear ribonucleoprotein B''	100.55	IQYAK(1)TDSDIISK	Tip60 OE only
P62314	SNRPD1	44	Small nuclear ribonucleoprotein Sm D1	63.306	AVK(1)MTLK	1.08
P62316	SNRPD2	8	Small nuclear ribonucleoprotein Sm D2	125.28	SLLNKPK(1)SEMTPEELQK	0.68
P62306	SNRPF	22	Small nuclear ribonucleoprotein F	62.338	PVMVK(1)LK	unquantifiable

Q13573	SNW1	153	SNW domain-containing protein 1	61.65	VALEK(1)SVSQK	unquantifiable
Q9UMY4	SNX12;SNX3	62	Sorting nexin-12;Sorting nexin-3	41.448	TNLPIFK(1)LK	unquantifiable
Q9UMY4	SNX12;SNX3	64	Sorting nexin-12;Sorting nexin-3	49.59	LK(1)ESCVR	unquantifiable
O60749	SNX2	447	Sorting nexin-2	102.51	IQQAK(1)NEIR	1.19
O60749	SNX2	223	Sorting nexin-2	71.153	VGK(1)EDSSSTEFVEK	Tip60 OE only
O60749	SNX2	287	Sorting nexin-2	42.19	MVNK(1)AADAVNK	unquantifiable
Q9UNH7	SNX6	307	Sorting nexin-6;Sorting nexin-6, N-terminally pro	71.614	ESQAAK(1)DLLYR	Tip60 OE only
Q9UNH6	SNX7	155	Sorting nexin-7	70.1	IFLTAQAWELSSHK(1)K	unquantifiable
Q9Y5X1	SNX9	534	Sorting nexin-9	92.062	ITLQDK(1)QNMVK	Tip60 OE only
P00441	SOD1	123	Superoxide dismutase [Cu-Zn]	142.07	TLVVHEK(1)ADDLGK	0.81
P00441	SOD1	129	Superoxide dismutase [Cu-Zn]	108.35	ADDLGK(1)GGNEESTK	0.91
P00441	SOD1	4	Superoxide dismutase [Cu-Zn]	82.452	ATK(1)AVCVLK	0.95
P00441	SOD1	137	Superoxide dismutase [Cu-Zn]	67.153	GGNEESTK(1)TGNAGSR	0.96
Q15005	SPCS2	50	Signal peptidase complex subunit 2	42.19	IDK(1)WDGSAVK	unquantifiable
Q15005	SPCS2	71	Signal peptidase complex subunit 2	70.268	VLLEK(1)YK	unquantifiable
Q69YQ0	SPECC1L	999	Cytospin-A	57.177	K(1)DPLSALAR	Tip60 OE only
Q96T58	SPEN	581	Msx2-interacting protein	65.897	IGGNK(1)IK	Tip60 OE only
Q13813	SPTAN1	274	Spectrin alpha chain, non-erythrocytic 1	110.08	EK(1)EQLMASDDFGR	0.86
Q13813	SPTAN1	2000	Spectrin alpha chain, non-erythrocytic 1	90.108	ENSLK(1)TDDYGR	0.96
Q13813	SPTAN1	864	Spectrin alpha chain, non-erythrocytic 1	63.184	AK(1)LHELNQK	1
Q13813	SPTAN1	646	Spectrin alpha chain, non-erythrocytic 1	62.378	LIDVNHYAK(1)DEVAAR	1.01
Q13813	SPTAN1	335	Spectrin alpha chain, non-erythrocytic 1	80.488	LQQSHPLSATQIQVK(1)R	1.1
Q13813	SPTAN1	1630	Spectrin alpha chain, non-erythrocytic 1	110.14	GACAGSEDAVK(1)AR	1.14
Q13813	SPTAN1	229	Spectrin alpha chain, non-erythrocytic 1	155.42	TK(1)QDEVNAAWQR	1.18
Q13813	SPTAN1	1288	Spectrin alpha chain, non-erythrocytic 1	193.16	DLAALGDK(1)VNSLGETAER	1.18
Q13813	SPTAN1	846	Spectrin alpha chain, non-erythrocytic 1	80.98	AVTQK(1)GNAMVEEGHFAAE[	1.23
Q13813	SPTAN1	1838	Spectrin alpha chain, non-erythrocytic 1	151.98	LSDDNTIGK(1)EEIQQR	1.5
Q13813	SPTAN1	441	Spectrin alpha chain, non-erythrocytic 1	89.047	EK(1)LTVLSEER	Tip60 OE only
Q13813	SPTAN1	1784	Spectrin alpha chain, non-erythrocytic 1	81.338	K(1)LLVGSEDYGR	Tip60 OE only
Q13813	SPTAN1	2047	Spectrin alpha chain, non-erythrocytic 1	84.06	DQLLAAK(1)HVQSK	Tip60 OE only
Q13813	SPTAN1	1963	Spectrin alpha chain, non-erythrocytic 1	99.653	VSDLEK(1)AAAQR	Tip60 OE only
Q13813	SPTAN1	2052	Spectrin alpha chain, non-erythrocytic 1	72.547	HVQSK(1)AIEAR	Tip60 OE only
Q01082	SPTBN1	668	Spectrin beta chain, non-erythrocytic 1	113.65	ILSSDDYGK(1)DLTSVMR	0.84
Q01082	SPTBN1	1878	Spectrin beta chain, non-erythrocytic 1	125.28	LQAAYAGDK(1)ADDIQK	1.04

Q01082	SPTBN1	1354	Spectrin beta chain, non-erythrocytic 1	72.789	EK(1)LTGLHK	1.12
Q01082	SPTBN1	1344	Spectrin beta chain, non-erythrocytic 1	83.087	EGMQLISEK(1)PETEAVVK	1.21
Q01082	SPTBN1	1421	Spectrin beta chain, non-erythrocytic 1	194.54	K(1)QQMLENQMEVR	1.45
Q01082	SPTBN1;SPTBN2;SPTI	288	Spectrin beta chain, non-erythrocytic 1;Spectrin	51.787	ALAVEGK(1)R	0.99
P61011	SRP54	9	Signal recognition particle 54 kDa protein	69.176	K(1)ITSALR	0.94
Q9UHB9	SRP68	288	Signal recognition particle subunit SRP68	57.348	AK(1)QAATMSEVEWR	Tip60 OE only
Q9UHB9	SRP68	462	Signal recognition particle subunit SRP68	43.814	TLVFK(1)AYR	unquantifiable
Q9UHB9	SRP68	213	Signal recognition particle subunit SRP68	50.607	AAIEAFNK(1)CK	unquantifiable
O76094	SRP72	391	Signal recognition particle subunit SRP72	80.014	ISQGNISK(1)ACLILR	unquantifiable
Q07955	SRSF1	48	Serine/arginine-rich splicing factor 1	89.903	DIDLK(1)NR	Tip60 OE only
Q13247	SRSF6	160	Serine/arginine-rich splicing factor 6	62.714	SYSDMK(1)R	unquantifiable
Q16629	SRSF7	24	Serine/arginine-rich splicing factor 7	141.38	VYVGNLGTGAGK(1)GELER	1.06
Q16629	SRSF7	70	Serine/arginine-rich splicing factor 7	70.028	GLDGK(1)VICGSR	1.07
P05455	SSB	317	Lupus La protein	137.98	K(1)IIEDQQESLNK	0.79
P05455	SSB	299	Lupus La protein	120.53	NK(1)EVTWEVLEGEVEK	0.93
P05455	SSB	37	Lupus La protein	88.643	FLK(1)EQIK	0.93
P05455	SSB	285	Lupus La protein	62.715	EALGK(1)AK	1.04
P05455	SSB	76	Lupus La protein	90.306	SK(1)AELMEISEDK	1.14
P05455	SSB	235	Lupus La protein	124.86	IGCLLK(1)FSGDLDDQTCR	Tip60 OE only
P05455	SSB	229	Lupus La protein	53.493	SLEEK(1)IGCLLK	Tip60 OE only
Q04837	SSBP1	122	Single-stranded DNA-binding protein, mitochon	94.465	IDYGEYMDK(1)NNVR	Tip60 OE only
P43308	SSR2	27	Translocon-associated protein subunit beta	84.743	LLASK(1)SLLNR	Tip60 OE only
P50502	ST13;ST13P5	186	Hsc70-interacting protein;Putative protein FAM	95.81	AIEINPDSAQPYK(1)WR	1.04
P50502	ST13;ST13P5;ST13P4	153	Hsc70-interacting protein;Putative protein FAM	129.96	LAILYAK(1)R	0.79
Q8N3U4	STAG2	339	Cohesin subunit SA-2	65.092	YVGWTMHDK(1)QGEVR	0.72
Q8N3U4	STAG2	346	Cohesin subunit SA-2	94.616	LK(1)CLTALQGLYYNK	0.9
Q8N3U4	STAG2	755	Cohesin subunit SA-2	94.616	ITESSSTK(1)EDLLR	Tip60 OE only
Q8N3U4	STAG2	551	Cohesin subunit SA-2	50.127	VLTAK(1)EK	Tip60 OE only
Q8N3U4	STAG2;STAG1	270	Cohesin subunit SA-2;Cohesin subunit SA-1	132.29	LELLLQK(1)R	0.92
P31948	STIP1	364	Stress-induced-phosphoprotein 1	94.767	LAYINPDLALEEK(1)NK	0.95
P31948	STIP1	100	Stress-induced-phosphoprotein 1	103.6	TYEEGLK(1)HEANNPQLK	0.95
P31948	STIP1	246	Stress-induced-phosphoprotein 1	107.83	DFDTALK(1)HYDK	0.96
P31948	STIP1	434	Stress-induced-phosphoprotein 1	80.219	K(1)AAALEAMK	0.96
P31948	STIP1	486	Stress-induced-phosphoprotein 1	116.37	HDSPEDVK(1)R	0.99

P31948	STIP1	530	Stress-induced-phosphoprotein 1	195.81	NPVIAQK(1)IQK	1.02
P31948	STIP1	78	Stress-induced-phosphoprotein 1	140.14	K(1)AAALEFLNR	1.03
P31948	STIP1	446	Stress-induced-phosphoprotein 1	143.86	DYTK(1)AMDVYQK	1.03
P31948	STIP1	237	Stress-induced-phosphoprotein 1	89.296	ELGNDAYK(1)K	1.04
P31948	STIP1	56	Stress-induced-phosphoprotein 1	103.01	GDYQK(1)AYEDGCK	1.05
P31948	STIP1	317	Stress-induced-phosphoprotein 1	67.214	YK(1)DAIHFYNK	1.05
P31948	STIP1	395	Stress-induced-phosphoprotein 1	130.04	DAK(1)LYSNR	1.06
P31948	STIP1	388	Stress-induced-phosphoprotein 1	120.65	HYTEAIK(1)R	1.06
P31948	STIP1	453	Stress-induced-phosphoprotein 1	243.7	AMDVYQK(1)ALDLDSSCK	1.11
P31948	STIP1	63	Stress-induced-phosphoprotein 1	58.09	AYEDGCK(1)TVDLKPDWGK	1.13
P31948	STIP1	347	Stress-induced-phosphoprotein 1	94.262	ILK(1)EQER	1.16
P31948	STIP1	462	Stress-induced-phosphoprotein 1	307.95	ALDLDSSCK(1)EAADGYQR	1.21
P31948	STIP1	373	Stress-induced-phosphoprotein 1	119.45	GNECFQK(1)GDYPQAMK	1.32
P31948	STIP1	442	Stress-induced-phosphoprotein 1	115.78	AAALEAMK(1)DYTK	Tip60 OE only
P31948	STIP1	366	Stress-induced-phosphoprotein 1	106.38	NK(1)GNECFQK	Tip60 OE only
P31948	STIP1	523	Stress-induced-phosphoprotein 1	53.754	DPQALSEHLK(1)NPVIAQK	Tip60 OE only
P31948	STIP1	50	Stress-induced-phosphoprotein 1	41.502	SAAYAK(1)K	unquantifiable
P16949	STMN1	80	Stathmin	77.072	EHEK(1)EVLQK	0.71
P16949	STMN1	100	Stathmin	90.657	MAEEK(1)LTHK	0.79
P16949	STMN1	41	Stathmin	55.401	ESVPEFPLSPPK(1)K	0.8
P16949	STMN1	109	Stathmin	124.08	MEANK(1)ENR	1.03
P16949	STMN1	119	Stathmin	71.349	EAQMAAK(1)LER	1.03
P16949	STMN1	128	Stathmin	62.823	DK(1)HIEEVR	Tip60 OE only
P16949	STMN1;STMN2	43	Stathmin;Stathmin-2	209.8	K(0.23)K(0.77)DLSLEEIQK	0.73
P16949	STMN1;STMN2	52	Stathmin;Stathmin-2	121.96	DLSLEEIQK(1)K	0.83
P16949	STMN1;STMN2;STMN	53	Stathmin;Stathmin-2;Stathmin-4	67.993	K(1)LEAAEER	0.76
Q9UJZ1	STOML2	222	Stomatin-like protein 2, mitochondrial	132.25	K(1)QAQILASEAEK	0.93
Q9UJZ1	STOML2	221	Stomatin-like protein 2, mitochondrial	93.111	ESAINVAEGK(1)K	0.97
Q9UJZ1	STOML2	254	Stomatin-like protein 2, mitochondrial	74.423	AK(1)AEAIR	1.13
Q9UJZ1	STOML2	250	Stomatin-like protein 2, mitochondrial	111.35	AEQINQAAGEASAVLAK(1)AK	1.17
Q9UJZ1	STOML2	140	Stomatin-like protein 2, mitochondrial	62.823	SELGK(1)LSLDK	1.31
Q9Y3F4	STRAP	73	Serine-threonine kinase receptor-associated prot	88.021	DATK(1)AATAAADFTAK	Tip60 OE only
Q9Y3F4	STRAP	156	Serine-threonine kinase receptor-associated prot	81.431	ALWCSEDK(1)QILSADDK	Tip60 OE only
P46977	STT3A	13	Dolichyl-diphosphooligosaccharide--protein gly	97.565	LSYEK(1)QDTLLK	1.54

P46977	STT3A	695	Dolichyl-diphosphooligosaccharide--protein gly	68.915	VK(1)DLDNR	Tip60 OE only
Q9UNE7	STUB1	72	E3 ubiquitin-protein ligase CHIP	70.235	ALCYLK(1)MQQHEQALADCR	1.17
Q9UNE7	STUB1	125	E3 ubiquitin-protein ligase CHIP	83.313	AYSLAK(1)EQR	Tip60 OE only
Q13190	STX5	120	Syntaxin-5	76.833	LTILAK(1)R	Tip60 OE only
P53999	SUB1	68	Activated RNA polymerase II transcriptional coac	93.649	DDNMFQIGK(1)MR	0.93
P53999	SUB1	80	Activated RNA polymerase II transcriptional coac	91.9	GK(1)VLIDIR	0.97
P53999	SUB1	53	Activated RNA polymerase II transcriptional coac	140.16	ALSSSK(1)QSSSSR	1.09
Q9P2R7	SUCLA2	417	Succinyl-CoA ligase [ADP-forming] subunit beta,	71.241	VDDAK(1)ALIADSGLK	0.89
P61956	SUMO2	7	Small ubiquitin-related modifier 2	84.213	ADEK(0.002)PK(0.998)EGVK	0.87
P61956	SUMO2;SUMO3	45	Small ubiquitin-related modifier 2;Small ubiquit	110.81	LMK(1)AYCER	0.98
Q9Y5B9	SUPT16H	216	FACT complex subunit SPT16	176.95	HSK(1)LAESVEK	0.95
Q9Y5B9	SUPT16H	211	FACT complex subunit SPT16	95.573	VMEIVDADEK(1)VR	Tip60 OE only
O15260	SURF4	139	Surfeit locus protein 4	68.44	SEGK(1)SMFAGVPTMR	unquantifiable
O15260	SURF4	22	Surfeit locus protein 4	67.726	VTK(1)QYLPHVAR	unquantifiable
O75683	SURF6	267	Surfeit locus protein 6	76.478	AQELEAK(1)MK	Tip60 OE only
O75683	SURF6	260	Surfeit locus protein 6	88.596	GQDEGK(1)AQELEAK	unquantifiable
O60506	SYNCRIP	100	Heterogeneous nuclear ribonucleoprotein Q	55.426	SAFLCGVMK(1)TYR	0.21
O60506	SYNCRIP	363	Heterogeneous nuclear ribonucleoprotein Q	171.36	AFSQFGK(1)LER	0.94
O60506	SYNCRIP	256	Heterogeneous nuclear ribonucleoprotein Q	158.79	TK(1)EQILEEFSK	0.94
O60506	SYNCRIP	91	Heterogeneous nuclear ribonucleoprotein Q	205.61	DSDLSHVQNK(1)SAFLCGVM	1
O60506	SYNCRIP	371	Heterogeneous nuclear ribonucleoprotein Q	98.337	LK(1)DYAFIHFDER	1.01
P37802	TAGLN2	79	Transgelin-2	68.846	K(1)IQASTMAFK	0.68
P37802	TAGLN2	17	Transgelin-2	113.7	EVQQK(1)IEK	0.96
P37837	TALDO1	277	Transaldolase	126.95	LVPVLSAK(1)AAQASDLEK	0.74
P37837	TALDO1	286	Transaldolase	123.95	AAQASDLEK(1)IHLDEK	0.95
P37837	TALDO1	115	Transaldolase	126.07	LSFDK(1)DAMVAR	0.98
P37837	TALDO1	292	Transaldolase	119.27	IHLDEK(1)SFR	1.04
P37837	TALDO1	81	Transaldolase	89.403	LGGSQEDQIK(1)NAIDK	1.27
P37837	TALDO1	307	Transaldolase	87.16	WLHNEDQMAVEK(1)LSDGIR	1.61
P37837	TALDO1	219	Transaldolase	72.547	SVTK(1)IYNYYK	Tip60 OE only
P37837	TALDO1	130	Transaldolase	81.865	LIELYK(1)EAGISK	Tip60 OE only
P37837	TALDO1	215	Transaldolase	80.69	SYEPLEDPGVK(1)SVTK	unquantifiable
Q13148	TARDBP	145	TAR DNA-binding protein 43	53.567	TGHSK(1)GFGFVR	0.84
Q13148	TARDBP	95	TAR DNA-binding protein 43	84.658	MDETDASSAVK(1)VK	unquantifiable

P26639	TARS	91	Threonine--tRNA ligase, cytoplasmic	133.05	VTLPDGK(1)QVDAESWK	0.9
P26639	TARS	243	Threonine--tRNA ligase, cytoplasmic	205.46	ILNEK(1)VNTPTTTVYR	0.94
P26639	TARS	213	Threonine--tRNA ligase, cytoplasmic	66.682	EK(1)QAFER	1.19
P26639	TARS	75	Threonine--tRNA ligase, cytoplasmic	158.3	AEHDSILAEK(1)AEK	Tip60 OE only
P26639	TARS;TARSL2	319	Threonine--tRNA ligase, cytoplasmic;Probable th	94.407	FQEEAK(1)NR	1.09
O75347	TBCA	12	Tubulin-specific chaperone A	85.212	IK(1)TGVVK	0.99
O75347	TBCA	23	Tubulin-specific chaperone A	64.567	EK(1)VMYEK	1.03
O75347	TBCA	95	Tubulin-specific chaperone A	130.94	DLEEAEEYK(1)EAR	1.14
Q99426	TBCB	153	Tubulin-folding cofactor B	141.58	LAEEK(1)AQASSIPVGSR	unquantifiable
Q15370	TCEB2	11	Transcription elongation factor B polypeptide 2	204.52	HK(1)TTIFTDAK	1.01
Q15370	TCEB2	28	Transcription elongation factor B polypeptide 2	85.666	ESSTVFELK(1)R	1.15
Q13428	TCOF1	820	Treacle protein	67.993	VVTAAAQAK(1)QR	1.05
P17987	TCP1	510	T-complex protein 1 subunit alpha	48.004	QAGVFEPTIVK(1)VK	0.83
P17987	TCP1	109	T-complex protein 1 subunit alpha	63.565	NADELVK(1)QK	0.88
P17987	TCP1	365	T-complex protein 1 subunit alpha	102.4	ICDDELILIK(1)NTK	0.88
P17987	TCP1	126	T-complex protein 1 subunit alpha	102.72	LACK(1)EAVR	1
P17987	TCP1	272	T-complex protein 1 subunit alpha	120.46	ESDITK(1)ER	1.06
P17987	TCP1	243	T-complex protein 1 subunit alpha	102.52	IACLDFSLQK(1)TK	1.11
P17987	TCP1	499	T-complex protein 1 subunit alpha	50.275	DNK(1)QAGVFEPTIVK	Tip60 OE only
P17987	TCP1	532	T-complex protein 1 subunit alpha	73.067	IDDLIK(1)LHPESK	Tip60 OE only
Q9NZ01	TECR	16	Very-long-chain enoyl-CoA reductase	56.81	EK(1)LCFLDK	0.87
Q9NZ01	TECR	12	Very-long-chain enoyl-CoA reductase	64.121	HYEVEILDAK(1)TR	0.97
Q9NZ01	TECR	60	Very-long-chain enoyl-CoA reductase	68.735	SLK(1)DEDVLQK	Tip60 OE only
Q00059	TFAM	186	Transcription factor A, mitochondrial	50.025	TVK(1)ENWK	0.98
Q00059	TFAM	174	Transcription factor A, mitochondrial	62.303	FQEAK(1)GDSPQEK	Tip60 OE only
Q96FV9	THOC1	275	THO complex subunit 1	51.268	SYK(1)LDDTQASR	1.41
Q8NI27	THOC2	542	THO complex subunit 2	69.628	VK(1)AQTIDR	unquantifiable
P52888	THOP1	317	Thimet oligopeptidase	93.262	AVILELK(1)R	1.17
Q9NQ88	TIGAR	98	Fructose-2,6-bisphosphatase TIGAR	85.958	YGVVEGK(1)ALSELR	Tip60 OE only
O43615	TIMM44	157	Mitochondrial import inner membrane transloca	100.48	TAK(1)QSAESVSK	0.94
O43615	TIMM44	262	Mitochondrial import inner membrane transloca	50.966	FFEMK(1)MK	Tip60 OE only
P29401	TKT	543	Transketolase	96.253	K(1)LILDSAR	0.77
P29401	TKT	16	Transketolase	86.67	LQALK(1)DTANR	0.92
P29401	TKT	144	Transketolase	143.85	YFDK(1)ASYR	0.99

P29401	TKT	232	Transketolase	129.84	AFGQAK(1)HQPTAIIAK	0.99
P29401	TKT	11	Transketolase	78.191	MESYHKPDQQK(1)LQALK	1.04
P29401	TKT	241	Transketolase	72.006	HQPTAIIAK(1)TFK	1.14
P29401	TKT	327	Transketolase	51.695	AYGQALAK(1)LGHASDR	1.21
P29401	TKT	310	Transketolase	117.67	MPSLPSYK(1)VGDK	Tip60 OE only
P29401	TKT	319	Transketolase	68.214	K(1)AYGQALAK	Tip60 OE only
P29401	TKT	59	Transketolase	64.776	YK(1)SQDPR	Tip60 OE only
P29401	TKT	352	Transketolase	55.567	NSTFSEIFK(1)K	unquantifiable
P29401	TKT;TKTL1	314	Transketolase;Transketolase-like protein 1	79.474	VGDK(1)IATR	1.06
Q9Y490	TLN1	869	Talin-1	60.49	ILADATAK(1)MVEAAK	0.76
Q9Y490	TLN1	2445	Talin-1	81.972	VK(1)ADQDSEAMK	1.2
Q9Y490	TLN1	687	Talin-1	93.593	AK(1)SVAQR	1.32
Q9Y490	TLN1	1947	Talin-1	91.937	AGALQCSPSDAYTK(1)K	1.39
Q9Y490	TLN1	334	Talin-1	118.33	LLGITK(1)ECVMR	Tip60 OE only
Q9Y490	TLN1	137	Talin-1	53.554	ELMEEK(1)K	Tip60 OE only
Q9Y490	TLN1	2099	Talin-1	80.245	ALGDLISATK(1)AAAGK	unquantifiable
Q99805	TM9SF2	423	Transmembrane 9 superfamily member 2	57.59	FYK(1)SFGGEK	Tip60 OE only
Q9HD45	TM9SF3	257	Transmembrane 9 superfamily member 3	86.898	YSK(1)EEEMDDMDR	1.27
Q9UM00	TMCO1	105	Transmembrane and coiled-coil domain-containi	53.683	K(0.104)K(0.896)ETITESAGR	unquantifiable
P49755	TMED10	143	Transmembrane emp24 domain-containing prot	87.498	VEK(0.994)LK(0.006)PLEVEL	0.79
P49755	TMED10	127	Transmembrane emp24 domain-containing prot	69.935	IPDQLVILDMK(1)HGVEAK	1.17
Q15363	TMED2	71	Transmembrane emp24 domain-containing prot	41.502	GIYK(1)GDR	unquantifiable
Q7Z7H5	TMED4	152	Transmembrane emp24 domain-containing prot	84.743	DK(1)LTELQLR	Tip60 OE only
Q9BVK6	TMED4;TMED9	226	Transmembrane emp24 domain-containing prot	91.265	HLK(1)SFFEAK	Tip60 OE only
Q9BVC6	TMEM109	215	Transmembrane protein 109	127.4	ASGAQLEAK(1)VR	0.69
P42167	TMPO	17	Lamina-associated polypeptide 2, isoforms beta/	106.13	LK(1)SELVANNVTLPAGEQR	Tip60 OE only
P42167	TMPO	401	Lamina-associated polypeptide 2, isoforms beta/	68.557	YVPLADVK(1)SEK	unquantifiable
P42167	TMPO	213	Lamina-associated polypeptide 2, isoforms beta/	92.47	TPVTLK(1)QR	unquantifiable
Q6ZXV5	TMTC3	583	Transmembrane and TPR repeat-containing prote	64.757	AK(1)EAYLK	1.01
Q15388	TOMM20	56	Mitochondrial import receptor subunit TOM20 h	40.058	AGLSK(1)LPDLK	unquantifiable
O94826	TOMM70A	563	Mitochondrial import receptor subunit TOM70	41.399	GNMEK(1)AIDMFNK	1.03
O94826	TOMM70A	302	Mitochondrial import receptor subunit TOM70	65.627	AK(1)QYMEEENYDK	1.19
O94826	TOMM70A	364	Mitochondrial import receptor subunit TOM70	76.847	EANVK(1)LR	1.45
O94826	TOMM70A	319	Mitochondrial import receptor subunit TOM70	82.171	IISECSK(1)EIDAEGK	Tip60 OE only

P11387	TOP1	746	DNA topoisomerase 1	83.614	IYNK(1)TQR	0.9
P55327	TPD52	108	Tumor protein D52	76.847	HLAEIK(1)R	1.04
Q16890	TPD52L1	68	Tumor protein D53	85.064	HLVEIK(1)QK	unquantifiable
P60174	TPI1	186	Triosephosphate isomerase	138.25	VVFEQTK(1)VIADNVK	0.8
P60174	TPI1	96	Triosephosphate isomerase	164.33	LDPK(1)IAVAAQNCYK	0.88
P60174	TPI1	179	Triosephosphate isomerase	165.18	EAGITEK(1)VVFEQTK	0.9
P60174	TPI1	225	Triosephosphate isomerase	174.24	TATPQQAQEVHEK(1)LR	0.99
P60174	TPI1	212	Triosephosphate isomerase	112.8	VVLAYEPVWAIGTGK(1)TATP(	1.04
P60174	TPI1	231	Triosephosphate isomerase	179.67	GWLK(1)SNVSDAVAQSTR	1.1
P60174	TPI1	193	Triosephosphate isomerase	84.17	VIADNVK(1)DWSK	unquantifiable
P67936	TPM4	223	Tropomyosin alpha-4 chain	113.93	TIDDLEEK(1)LAQAK	0.92
P67936	TPM4	215	Tropomyosin alpha-4 chain	132.88	LEK(1)TIDDLEEK	Tip60 OE only
P12270	TPR	204	Nucleoprotein TPR	67.704	TK(1)TDELLALGR	0.94
P12270	TPR	1081	Nucleoprotein TPR	157.96	IAVEAQNK(1)YER	0.95
P12270	TPR	1361	Nucleoprotein TPR	99.802	LLSEK(1)EVHTK	1.06
P12270	TPR	1507	Nucleoprotein TPR	161.79	TLSEK(1)ETEAR	1.08
P12270	TPR	1561	Nucleoprotein TPR	65.252	AIVAAK(1)SK	1.13
P12270	TPR	905	Nucleoprotein TPR	75.797	EIATLK(1)QHLSNMEVQVASQ!	1.15
P12270	TPR	834	Nucleoprotein TPR	65.038	SETETK(1)QR	1.16
P12270	TPR	1435	Nucleoprotein TPR	53.163	TITQVK(1)K	1.25
P12270	TPR	1248	Nucleoprotein TPR	58.815	EK(1)VQVTAK	1.31
P12270	TPR	299	Nucleoprotein TPR	207.88	SAADDSEAK(1)SNELTR	1.34
P12270	TPR	265	Nucleoprotein TPR	59.426	EAK(1)EQQASMEEK	1.44
P12270	TPR	290	Nucleoprotein TPR	50.145	LSNLYK(1)SAADDSEAK	1.51
P12270	TPR	1603	Nucleoprotein TPR	83	ITALK(1)SQYEGR	2.09
P12270	TPR	1427	Nucleoprotein TPR	79.474	IIDIQEK(1)VK	Tip60 OE only
P12270	TPR	1420	Nucleoprotein TPR	64.439	DLDAK(1)IIDIQEK	Tip60 OE only
P12270	TPR	215	Nucleoprotein TPR	57.708	EK(1)GNEILELK	Tip60 OE only
P12270	TPR	1015	Nucleoprotein TPR	80.165	EK(1)QELQDDK	Tip60 OE only
P13693	TPT1	102	Translationally-controlled tumor protein	87.308	GK(1)LEEQRPER	1.03
Q12931	TRAP1	95	Heat shock protein 75 kDa, mitochondrial	140.45	HEFQAETK(1)K	0.78
Q12931	TRAP1	126	Heat shock protein 75 kDa, mitochondrial	208.46	ELISNASDALEK(1)LR	0.87
Q12931	TRAP1	258	Heat shock protein 75 kDa, mitochondrial	105.52	IIIHLK(1)SDCK	0.9
Q12931	TRAP1	181	Heat shock protein 75 kDa, mitochondrial	126.48	SGSK(1)AFLDALQNQAEASSK	0.97

Q12931	TRAP1	375	Heat shock protein 75 kDa, mitochondrial	132.91	VLIQTK(1)ATDILPK	1.04
Q12931	TRAP1	262	Heat shock protein 75 kDa, mitochondrial	112.75	SDCK(1)EFSSEAR	1.05
Q12931	TRAP1	598	Heat shock protein 75 kDa, mitochondrial	78.516	VTNVK(1)VTLR	1.13
Q12931	TRAP1	109	Heat shock protein 75 kDa, mitochondrial	133.57	SLYSEK(1)EVFIR	1.13
Q12931	TRAP1	96	Heat shock protein 75 kDa, mitochondrial	75.738	K(1)LLDIVAR	1.22
Q12931	TRAP1	431	Heat shock protein 75 kDa, mitochondrial	91.041	FFIDQSK(1)K	Tip60 OE only
Q12931	TRAP1	332	Heat shock protein 75 kDa, mitochondrial	58.487	YTLHYK(1)TDAPLNIR	Tip60 OE only
Q13263	TRIM28	272	Transcription intermediary factor 1-beta	189.7	HATLQK(1)STK	0.93
Q13263	TRIM28	319	Transcription intermediary factor 1-beta	208.95	VLVNDAQK(1)VTEGQQER	0.94
Q13263	TRIM28	266	Transcription intermediary factor 1-beta	161.17	LGDK(1)HATLQK	0.98
Q13263	TRIM28	779	Transcription intermediary factor 1-beta	196.38	LTEDK(1)ADVQSIIGLQR	0.99
Q13263	TRIM28	199	Transcription intermediary factor 1-beta	63.565	STGPAK(1)SR	1.05
Q13263	TRIM28	261	Transcription intermediary factor 1-beta	138.37	LLASLVK(1)R	1.07
Q13263	TRIM28	366	Transcription intermediary factor 1-beta	117.04	K(1)LIYFQLHR	1.11
Q13263	TRIM28	31	Transcription intermediary factor 1-beta	89.181	AASAAAASAAAASAASGSPGP(	Tip60 OE only
Q7L0Y3	TRMT10C	173	Mitochondrial ribonuclease P protein 1	101.11	LLETTEEDK(1)QK	unquantifiable
P10155	TROVE2	369	60 kDa SS-A/Ro ribonucleoprotein	53.166	TVEPTGK(1)R	unquantifiable
P10155	TROVE2	224	60 kDa SS-A/Ro ribonucleoprotein	47.706	ALSVETEK(1)LLK	unquantifiable
Q8WWH5	TRUB1	58	Probable tRNA pseudouridine synthase 1	60.828	VSK(1)AALATK	unquantifiable
Q15631	TSN	187	Translin	129.1	LLNLK(1)NDSLR	0.88
Q15631	TSN	219	Translin	91.937	GFNK(1)ETAAACVEK	1.05
Q15631	TSN	76	Translin	74.941	THLTSLK(1)TK	Tip60 OE only
O60637	TSPAN3	110	Tetraspanin-3	101.54	AK(1)VENEVDR	unquantifiable
Q8N4P2	TTC30B	392	Tetratricopeptide repeat protein 30B	71.692	K(1)LTIQVQEAR	unquantifiable
Q8N5M4	TTC9C	109	Tetratricopeptide repeat protein 9C	41.979	QPDNAK(1)ALYR	unquantifiable
Q8WZ42	TTN	19231	Titin	60.59	NLK(1)IVDVSSDR	Tip60 OE only
Q8WZ42	TTN	32879	Titin	46.351	TVK(1)GEFR	unquantifiable
P68363	TUBA1B;TUBA1A;TUE	112	Tubulin alpha-1B chain;Tubulin alpha-1A chain;T	176.32	GHYTIGK(1)EIIDLVLDR	0.98
P68363	TUBA1B;TUBA1A;TUE	336	Tubulin alpha-1B chain;Tubulin alpha-1A chain;T	118.9	DVNAAIATIK(1)TK	0.88
P68363	TUBA1B;TUBA1A;TUE	60	Tubulin alpha-1B chain;Tubulin alpha-1A chain;T	174.2	TIGGGDDSFNTFFSETGAGK(1)	0.99
P68363	TUBA1B;TUBA1A;TUE	326	Tubulin alpha-1B chain;Tubulin alpha-1A chain;T	94.122	GDVVPK(1)DVNAAIATIK	0.99
P68363	TUBA1B;TUBA1A;TUE	163	Tubulin alpha-1B chain;Tubulin alpha-1A chain;T	50.703	LSVDYGK(1)K	unquantifiable
P68363	TUBA1B;TUBA1A;TUE	370	Tubulin alpha-1B chain;Tubulin alpha-1A chain;T	122.78	VGINYQPPTVVPGGDLAK(1)V	0.97
P68363	TUBA1B;TUBA1A;TUE	96	Tubulin alpha-1B chain;Tubulin alpha-1A chain;T	102.98	QLFHPEQLITGK(1)EDAANNY/	1.36

P68363	TUBA1B;TUBA1A;TUE	394	Tubulin alpha-1B chain;Tubulin alpha-1A chain;T	124.67	LDHK(1)FDLMYAK	1.02
P68363	TUBA1B;TUBA1A;TUE	401	Tubulin alpha-1B chain;Tubulin alpha-1A chain;T	135.35	FDLMYAK(1)R	1.11
P68371	TUBB;TUBB4B;TUBB2	324	Tubulin beta chain;Tubulin beta-4B chain;Tubuli	238.01	MSMK(1)EVDEQMLNVQNK	0.99
P68371	TUBB4B	58	Tubulin beta-4B chain	206.36	INVYYNEATGGK(1)YVPR	0.96
P49411	TUFM	88	Elongation factor Tu, mitochondrial	99.802	ILAEGGGAK(1)FK	0.76
P49411	TUFM	91	Elongation factor Tu, mitochondrial	131.82	K(1)YEEIDNAPEER	1.12
P49411	TUFM	234	Elongation factor Tu, mitochondrial	70.399	DPELGLK(1)SVQK	1.87
P40222	TXLNA	330	Alpha-taxilin	117.09	HK(1)DLQQQLVDAK	unquantifiable
P40222	TXLNA	256	Alpha-taxilin	81.548	SLK(1)EEGVQR	unquantifiable
P40222	TXLNA;TXLNG	417	Alpha-taxilin;Gamma-taxilin	41.979	QEMEK(1)MTK(1)K	unquantifiable
P40222	TXLNA;TXLNG	420	Alpha-taxilin;Gamma-taxilin	41.979	QEMEK(1)MTK(1)K	unquantifiable
P10599	TXN	94	Thioredoxin	133.99	VGEFSGANK(1)EK	0.81
P10599	TXN	3	Thioredoxin	89.123	VK(1)QIESK	0.82
P10599	TXN	39	Thioredoxin	87.216	MIK(1)PFFHSLSEK	0.82
P10599	TXN	85	Thioredoxin	145.9	GQK(1)VGEFSGANK	0.84
Q8NBS9	TXNDC5	118	Thioredoxin domain-containing protein 5	98.132	VYVAK(1)VDCTAHSDVCSAQG	1.09
Q16881	TXNRD1	580	Thioredoxin reductase 1, cytoplasmic	78.334	IICNTK(1)DNER	Tip60 OE only
P04818	TYMS	99	Thymidylate synthase	42.718	GSTNAK(1)ELSSK	unquantifiable
P0DN76	U2AF1L4;U2AF1	15	Splicing factor U2AF 26 kDa subunit;Splicing fact	52.867	DK(1)VNCSFYFK	Tip60 OE only
P26368	U2AF2	413	Splicing factor U2AF 65 kDa subunit	49.089	YGLVK(1)SIEIPRPVDGVEVPG	1.1
P26368	U2AF2	462	Splicing factor U2AF 65 kDa subunit	97.86	VVVTK(1)YCDPDSYHR	1.11
O15042	U2SURP	840	U2 snRNP-associated SURP motif-containing pro	138.54	YSEMSEEK(1)R	unquantifiable
O15042	U2SURP	647	U2 snRNP-associated SURP motif-containing pro	82.908	TIQGHLQSENFK(1)QR	unquantifiable
Q16222	UAP1;UAP1L1	278	UDP-N-acetylhexosamine pyrophosphorylase;UD	51.276	GADCGAK(1)VVEK	Tip60 OE only
P22314	UBA1	296	Ubiquitin-like modifier-activating enzyme 1	78.334	GGIVSQVK(1)VPK	0.95
P22314	UBA1	528	Ubiquitin-like modifier-activating enzyme 1	101.65	LK(1)SDTAAAAVR	1
P22314	UBA1	526	Ubiquitin-like modifier-activating enzyme 1	60.436	PWDVTK(1)LK	1.02
P22314	UBA1	443	Ubiquitin-like modifier-activating enzyme 1	179.68	EVLTEDK(1)CLQR	1.03
P22314	UBA1	884	Ubiquitin-like modifier-activating enzyme 1	78.616	SK(1)LIAGK	1.07
P22314	UBA1	465	Ubiquitin-like modifier-activating enzyme 1	118.52	YDGQVAVFGSDLQEK(1)LGK	1.18
Q9UBT2	UBA2	420	SUMO-activating enzyme subunit 2	69.954	TIFLNK(1)QPNPR	0.91
Q9UBT2	UBA2	241	SUMO-activating enzyme subunit 2	40.002	ISTK(1)EWAK	1.13
Q9UBT2	UBA2	409	SUMO-activating enzyme subunit 2	146.81	ILSGK(1)IDQCR	1.16
Q9UBT2	UBA2	236	SUMO-activating enzyme subunit 2	79.906	ASNEDGDIK(1)R	1.17

Q9UBT2	UBA2	316	SUMO-activating enzyme subunit 2	104.43	DQQVLDVK(1)SYAR	1.18
Q9UBT2	UBA2	72	SUMO-activating enzyme subunit 2	99.139	SK(1)AQVAK	2.1
Q9UBT2	UBA2	260	SUMO-activating enzyme subunit 2	68.422	LFK(1)DDIR	Tip60 OE only
Q9UBT2	UBA2	617	SUMO-activating enzyme subunit 2	79.693	KLDEK(1)ENLSAK	unquantifiable
P63279	UBE2I	74	SUMO-conjugating enzyme UBC9	45.161	DDYPSSPPK(1)CK	1.04
P63279	UBE2I	65	SUMO-conjugating enzyme UBC9	81.865	MLFK(1)DDYPSSPPK	Tip60 OE only
P61086	UBE2K	18	Ubiquitin-conjugating enzyme E2 K	44.309	EVLK(1)SEETSK	0.87
P61086	UBE2K	24	Ubiquitin-conjugating enzyme E2 K	103.56	SEETSK(1)NQIK	0.91
P61081	UBE2M	72	NEDD8-conjugating enzyme Ubc12	112.13	LVICPDEGFYK(1)SGK	0.99
P61081	UBE2M	8	NEDD8-conjugating enzyme Ubc12	89.369	LFSLK(1)QQK	1.23
P61088	UBE2N	92	Ubiquitin-conjugating enzyme E2 N	83.005	ICLDILK(1)DK	0.83
P61088	UBE2N	82	Ubiquitin-conjugating enzyme E2 N	87.184	IYHPNVDK(1)LGR	1.04
P61088	UBE2N;UBE2NL	94	Ubiquitin-conjugating enzyme E2 N;Putative ubi	133.23	DK(1)WSPALQIR	1.02
Q13404	UBE2V1	10	Ubiquitin-conjugating enzyme E2 variant 1	83.783	AATTGSGVK(1)VPR	0.89
Q13404	UBE2V1;UBE2V2	131	Ubiquitin-conjugating enzyme E2 variant 1;Ubiq	94.114	LMMSK(1)ENMK	Tip60 OE only
Q5T4S7	UBR4	4820	E3 ubiquitin-protein ligase UBR4	41.399	GQVVTK(1)TALLK	unquantifiable
P09936	UCHL1	78	Ubiquitin carboxyl-terminal hydrolase isozyme L	111.65	GQEVSPK(1)VYFMK	1.03
P09936	UCHL1	199	Ubiquitin carboxyl-terminal hydrolase isozyme L	66.994	DAAK(1)VCR	1.12
P09936	UCHL1	71	Ubiquitin carboxyl-terminal hydrolase isozyme L	137.89	QIEELK(1)GQEVSPK	1.41
Q9NYU2	UGGT1	585	UDP-glucose:glycoprotein glucosyltransferase 1	69.327	VK(1)VEHVVSVLEK	0.78
Q92900	UPF1	604	Regulator of nonsense transcripts 1	96.015	DETGELSSADEK(1)R	1.52
P22695	UQCRC2	92	Cytochrome b-c1 complex subunit 2, mitochonc	85.288	LTSSLTTK(1)GASSFK	Tip60 OE only
O60763	USO1	815	General vesicular transport factor p115	100.25	LQTEK(1)QELLQK	unquantifiable
P54578	USP14	336	Ubiquitin carboxyl-terminal hydrolase 14	55.676	EK(1)ESVNAK	0.82
P54578	USP14	130	Ubiquitin carboxyl-terminal hydrolase 14	54.982	SVPELK(1)DALK	1.87
P45974	USP5	468	Ubiquitin carboxyl-terminal hydrolase 5	63.48	FLVEEK(1)IK	1.05
Q15836	VAMP3;VAMP2;VAM	42	Vesicle-associated membrane protein 3;Vesicle-a	43.297	DQK(1)LSELDDR	Tip60 OE only
Q9P0L0	VAPA	188	Vesicle-associated membrane protein-associated	65.574	LQGEMMK(1)LSEENR	Tip60 OE only
Q9P0L0	VAPA;VAPB	52	Vesicle-associated membrane protein-associated	86.866	VK(1)TTAPR	0.92
P50552	VASP	348	Vasodilator-stimulated phosphoprotein	50.354	VK(1)QELLEEVKK	Tip60 OE only
P61758	VBP1	59	Prefoldin subunit 3	56.258	KLDEQYQK(1)YK	0.76
P18206	VCL	646	Vinculin	149.49	LGATAEK(1)AAAVGTANK	0.79
P18206	VCL	666	Vinculin	88.187	STVEGIQASVK(1)TAR	0.84
P18206	VCL	59	Vinculin	108.56	VGK(1)ETVQTTEDQILK	0.84

P18206	VCL	496	Vinculin	85.554	AAVHLEGK(1)IEQAQR	0.88
P18206	VCL	316	Vinculin	70.908	VGELCAGK(1)ER	0.93
P18206	VCL	830	Vinculin	72.138	ILGAVAK(1)VR	0.97
P18206	VCL	768	Vinculin	90.939	ILLVAK(1)R	1.02
P18206	VCL	1070	Vinculin	144.65	ILSTVK(1)ATMLGR	1.26
P18206	VCL	219	Vinculin	103.26	NSK(1)NQGIEEALK	Tip60 OE only
P18206	VCL	426	Vinculin	78.655	IAELCDDPK(1)ER	Tip60 OE only
P18206	VCL	992	Vinculin	58.433	GNDIIAAAK(1)R	Tip60 OE only
P55072	VCP	524	Transitional endoplasmic reticulum ATPase	92.19	GVLFYGPPGCGK(1)TLLAK	0.72
P55072	VCP	231	Transitional endoplasmic reticulum ATPase	60.157	HPALFK(1)AIGVKPPR	0.83
P55072	VCP	190	Transitional endoplasmic reticulum ATPase	100.88	VVETDPSPYCIVAPDTVIHCEGI	0.94
P55072	VCP	754	Transitional endoplasmic reticulum ATPase	202.16	K(1)YEMFAQTLQQSR	0.95
P55072	VCP	565	Transitional endoplasmic reticulum ATPase	92.692	EIFDK(1)AR	0.98
P55072	VCP	251	Transitional endoplasmic reticulum ATPase	140.24	GILLYGPPGTGK(1)TLIAR	0.99
P55072	VCP	658	Transitional endoplasmic reticulum ATPase	135.87	VAILK(1)ANLR	1.07
P55072	VCP	8	Transitional endoplasmic reticulum ATPase	139.89	ASGADSK(1)GDDLSTAILK	1.14
P21796	VDAC1	12	Voltage-dependent anion-selective channel prote	100.55	AVPPTYADLGK(1)SAR	0.9
P21796	VDAC1	109	Voltage-dependent anion-selective channel prote	137.89	LTFDSSFSPNTGK(1)K	0.97
P21796	VDAC1	61	Voltage-dependent anion-selective channel prote	129.1	VTGSLETK(1)YR	Tip60 OE only
P45880	VDAC2	277	Voltage-dependent anion-selective channel prote	72.819	LTLSALVDGK(1)SINAGGHK	0.79
P45880	VDAC2	72	Voltage-dependent anion-selective channel prote	110.87	VTGTLETK(1)YK	1.06
P45880	VDAC2	120	Voltage-dependent anion-selective channel prote	134.2	LTFDTTFSPNTGK(1)K	Tip60 OE only
Q9Y277	VDAC3	15	Voltage-dependent anion-selective channel prote	53.891	AAK(1)DVFNK	0.66
Q9Y277	VDAC3	12	Voltage-dependent anion-selective channel prote	77.64	CNTPTYCDLGK(1)AAK	1.26
P08670	VIM	235	Vimentin	118.51	KVESLQEEIAFLK(1)K	0.84
P08670	VIM	445	Vimentin	102.61	TLLIK(1)TVETR	0.98
P08670	VIM	313	Vimentin	47.823	QAK(1)QESTEYR	1.03
P08670	VIM	168	Vimentin	206.43	QVDQLTNDK(1)AR	1.05
P08670	VIM	139	Vimentin	125.75	ILLAELEQLK(1)GQGK	1.07
P08670	VIM	120	Vimentin	115.49	FANYIDK(1)VR	1.07
P08670	VIM	439	Vimentin	61.692	ETNLDSLPLVDTHSK(1)R	1.12
P08670	VIM	373	Vimentin	271.14	LQDEIQNMK(1)EEMAR	1.2
P08670	VIM	223	Vimentin	145.55	K(1)VESLQEEIAFLK	1.2
P08670	VIM;DES	104	Vimentin;Desmin	156.71	TNEK(1)VELQELNDR	1.07

P08670	VIM;PRPH	292	Vimentin;Peripherin	146.11	NLQEAEEWYK(1)SK	0.95
P08670	VIM;PRPH	294	Vimentin;Peripherin	101.38	SK(1)FADLSEAANR	1.12
Q96QK1	VPS35	38	Vacuolar protein sorting-associated protein 35	43.808	NK(1)LMDALK	Tip60 OE only
Q9NP79	VTA1	52	Vacuolar protein sorting-associated protein VTA1	116.73	IDSK(1)TPECR	0.79
P23381	WARS	33	Tryptophan--tRNA ligase, cytoplasmic;T1-TrpRS;T	52.555	AGNASK(1)DEIDSAVK	0.67
P23381	WARS	111	Tryptophan--tRNA ligase, cytoplasmic;T1-TrpRS;T	60.59	FGSSK(1)IDK	0.9
Q9Y2W2	WBP11	46	WW domain-binding protein 11	54.525	AAVLK(1)MK	0.94
Q969T9	WBP2	62	WW domain-binding protein 2	47.082	VIFLSK(1)GK	unquantifiable
Q9UNX4	WDR3	360	WD repeat-containing protein 3	89.08	VTNIK(1)TSAK	0.73
Q5MNZ6	WDR45B	206	WD repeat domain phosphoinositide-interacting	90.827	IATASEK(1)GTLIR	unquantifiable
Q9Y4E6	WDR7	862	WD repeat-containing protein 7	50.127	LSHGK(1)TEVGR	Tip60 OE only
O14980	XPO1	531	Exportin-1	66.435	DLLGLCEQK(1)R	1.05
O14980	XPO1	122	Exportin-1	121.6	TSSDPTCVEK(1)EK	1.23
O14980	XPO1	680	Exportin-1	78.908	NVDILK(1)DPETVK	1.58
P13010	XRCC5	543	X-ray repair cross-complementing protein 5	78.934	TLFPLIEAK(1)K	0.8
P13010	XRCC5	155	X-ray repair cross-complementing protein 5	81.431	SQLDIIIHSLK(1)K	0.88
P13010	XRCC5	265	X-ray repair cross-complementing protein 5	107.21	IAAYK(1)SILQER	0.93
P13010	XRCC5	648	X-ray repair cross-complementing protein 5	136.57	EEAIK(1)FSEEQR	1.02
P13010	XRCC5	338	X-ray repair cross-complementing protein 5	81.475	SEGK(1)CFSVLGFCK	1.04
P13010	XRCC5	702	X-ray repair cross-complementing protein 5	130.56	EEASGSSVTAEEAK(1)K	1.05
P13010	XRCC5	202	X-ray repair cross-complementing protein 5	196.76	GITEQQK(1)EGLEIVK	1.17
P13010	XRCC5	274	X-ray repair cross-complementing protein 5	88.181	K(1)TWTVVDAK	1.54
P13010	XRCC5	466	X-ray repair cross-complementing protein 5	71.548	K(0.929)DEK(0.071)TDTLEDI	Tip60 OE only
P12956	XRCC6	565	X-ray repair cross-complementing protein 6	90.146	VEYSEEELK(1)THISK	0.87
P12956	XRCC6	357	X-ray repair cross-complementing protein 6	66.994	PLVLLK(1)K	0.88
P12956	XRCC6	238	X-ray repair cross-complementing protein 6	189.13	VHFEESSK(1)LEDLLR	0.95
P12956	XRCC6	461	X-ray repair cross-complementing protein 6	123.88	IMATPEQVGK(1)MK	0.98
P12956	XRCC6	189	X-ray repair cross-complementing protein 6	73.616	TK(1)AGDLR	0.98
P12956	XRCC6	317	X-ray repair cross-complementing protein 6	162.97	TFNTSTGGLLLPSDTK(1)R	0.99
P12956	XRCC6	570	X-ray repair cross-complementing protein 6	105.4	THISK(1)GTLGK	1.02
P12956	XRCC6	287	X-ray repair cross-complementing protein 6	72.434	ALKPPPIK(1)LYR	1.02
P12956	XRCC6	338	X-ray repair cross-complementing protein 6	117.93	EETEELK(1)R	1.03
P12956	XRCC6	123	X-ray repair cross-complementing protein 6	187.97	ILELDQFK(1)GQQGQK	1.1
P12956	XRCC6	468	X-ray repair cross-complementing protein 6	110.54	AIVEK(1)LR	1.1

P12956	XRCC6	596	X-ray repair cross-complementing protein 6	105.2	K(1)QELLEALTK	1.17
P12956	XRCC6	591	X-ray repair cross-complementing protein 6	94.302	AYGLK(1)SGLK	1.25
P12956	XRCC6	463	X-ray repair cross-complementing protein 6	83.753	MK(1)AIVEK	Tip60 OE only
Q9H0D6	XRN2	123	5'-3' exoribonuclease 2	61.815	ASK(1)EGMEAAVEK	Tip60 OE only
P54577	YARS	10	Tyrosine--tRNA ligase, cytoplasmic;Tyrosine--tRN	80.522	GDAPSPEEK(1)LHLITR	1.09
P54577	YARS	119	Tyrosine--tRNA ligase, cytoplasmic;Tyrosine--tRN	116.52	FIK(1)GTDYQLSK	1.13
P67809	YBX1;YBX3	64	Nuclease-sensitive element-binding protein 1;Y-b	143.01	VLGTVK(1)WFNVR	1.33
Q9BYJ9	YTHDF1;YTHDF2;YTH	515	YTH domain-containing family protein 1;YTH dor	88.496	DTQEVPLEK(1)AK	Tip60 OE only
P31946	YWHAB	11	14-3-3 protein beta/alpha;14-3-3 protein beta/al	71.715	SELVQK(1)AK	0.9
P31946	YWHAB	82	14-3-3 protein beta/alpha;14-3-3 protein beta/al	71.379	KQQMGK(1)EYR	1.15
P31946	YWHAB	70	14-3-3 protein beta/alpha;14-3-3 protein beta/al	155.42	VISSIEQK(1)TER	Tip60 OE only
P63104	YWHAB;YWHAG;YWH	120	14-3-3 protein beta/alpha;14-3-3 protein beta/al	89.903	VFYLK(1)MK	0.98
P62258	YWHAE	12	14-3-3 protein epsilon	140.24	MDDREDLVYQAK(1)LAEQAER	0.91
P62258	YWHAE	142	14-3-3 protein epsilon	98.754	K(1)EAAENSLVAYK	0.92
P62258	YWHAE	118	14-3-3 protein epsilon	115.5	HLIPAANTGESK(1)VFYYK	0.93
P62258	YWHAE	28	14-3-3 protein epsilon	129.1	YDEMVESMK(1)K	0.94
P62258	YWHAE	69	14-3-3 protein epsilon	51.268	IISSIEQK(1)EENK	0.95
P62258	YWHAE	78	14-3-3 protein epsilon	67.035	GGEDK(1)LK	0.97
P62258	YWHAE	123	14-3-3 protein epsilon	62.842	VFYYK(1)MK	1.08
P62258	YWHAE	125	14-3-3 protein epsilon	84.244	MK(1)GDYHR	unquantifiable
P61981	YWHAG	69	14-3-3 protein gamma;14-3-3 protein gamma, N-	121.08	VISSIEQK(1)TSADGNEK	0.9
P61981	YWHAG	10	14-3-3 protein gamma;14-3-3 protein gamma, N-	105.39	EQLVQK(1)AR	1.04
P61981	YWHAG	142	14-3-3 protein gamma;14-3-3 protein gamma, N-	168.8	YLAEVATGEK(1)R	Tip60 OE only
P63104	YWHAG;YWHAZ;YWH	122	14-3-3 protein gamma;14-3-3 protein gamma, N-	124.89	MK(1)GDYYR	1.01
P27348	YWHAQ	139	14-3-3 protein theta	71.879	K(1)QTIDNSQGAYQEAFDISK	0.8
P27348	YWHAQ	115	14-3-3 protein theta	83.314	YLIANATNPESK(1)VFYLK	0.91
P27348	YWHAQ	68	14-3-3 protein theta	174.4	VISSIEQK(1)TDTSDK	0.95
P27348	YWHAQ	80	14-3-3 protein theta	170.32	LQLIK(1)DYR	0.95
P27348	YWHAQ	120	14-3-3 protein theta	89.903	VFYLK(1)MK	0.96
P27348	YWHAQ	85	14-3-3 protein theta	130.22	EK(1)VESELR	0.98
P27348	YWHAQ	9	14-3-3 protein theta	91.041	TELIQK(1)AK	0.98
P27348	YWHAQ	3	14-3-3 protein theta	136.57	MEK(1)TELIQK	1.02
P27348	YWHAQ;YWHAB	122	14-3-3 protein theta;14-3-3 protein beta/alpha;1	114.59	MK(1)GDYFR	1.06
	YWHAQ;YWHAB		14-3-3 protein theta;14-3-3 protein beta/alpha;1	69.825	K(1)EMQPTHPIR	unquantifiable

P63104	YWHAZ	68	14-3-3 protein zeta/delta	179.67	VVSSIEQK(1)TEGAEK	0.87
P63104	YWHAZ	9	14-3-3 protein zeta/delta	208.75	NELVQK(1)AK	0.92
P63104	YWHAZ	138	14-3-3 protein zeta/delta	135.86	YLAEVAAGDDK(1)K	0.94
P63104	YWHAZ	158	14-3-3 protein zeta/delta	69.825	K(1)EMQPTHPIR	0.95
P63104	YWHAZ	3	14-3-3 protein zeta/delta	128	MDK(1)NELVQK	0.95
P63104	YWHAZ	157	14-3-3 protein zeta/delta	126.09	GIVDQSQQAYQEAFEISK(1)K	1.02
P63104	YWHAZ	85	14-3-3 protein zeta/delta	109.01	EK(1)IETELR	1.06
P63104	YWHAZ	139	14-3-3 protein zeta/delta	157.75	K(1)GIVDQSQQAYQEAFEISK	1.11
O95159	ZFPL1	121	Zinc finger protein-like 1	90.709	EK(1)LATVNWAR	unquantifiable
Q96KR1	ZFR	796	Zinc finger RNA-binding protein	92.439	VGVLAK(1)GLLLR	1.19
O43670	ZNF207	100	BUB3-interacting and GLEBS motif-containing pr	155.07	LLEQK(1)TQESQK	Tip60 OE only
P0DP25		31		105.65	DGDGTITTK(1)ELGTVMR	0.76
