# Supplementary material for: Quantitative Proteomics Reveals the Role of Lysine 2-Hydroxyisobutyrylation Pathway Mediated by Tip60
Source: Oxid Med Cell Longev. 2022 Feb 8;2022:4571319. doi: 10.1155/2022/4571319 (PMC8847014; doi:10.1155/2022/4571319)
Supplement: Supplementary Materials — Tables S1 and S2 are the complete lists of identified Khib and Kac sites in WT and Tip60 OE cells. Supplementary Materials Figure S1 is the representative MS2 spectra of the Tip60-targeted Khib peptides. Table S1: complete list of identified Khib sites in WT and Tip60 OE cells. Table S2: complete list of identified Kac sites in WT and Tip60 OE cells. Figure S1: the representative MS2 spectra of the Tip60-targeted Khib peptides, including K147hib of EF1G, K115hib of KPYM, K416hib of SEC63, and K624hib of HS90B. [file 4571319.f1.zip › SI_Table S2.pdf]

Table S2. Complete list of identified Kac sites in WT and Tip60 OE cells.

Column describtion

**Protein:**	Identifiers of proteins this site is associated with
**Gene names:**	NCBI Gene names of leading proteins
**Position:**	For each protein identifier in the 'Proteins' column you find here the psoition of the site in the respective protein sequence
**Protein names:**	NCBI Gene identifiers of leading proteins
**Score:**	Andromeda score of the identified MS/MS spectra
**Acetyl (K) Probabilities:**	Sequence representation of the peptide including PTM positioning probabilities ([0..1], where 1 is best match) for 'Hib (K)'.
**Ratio WT/OE:**	Abundance ratio of the site, WT/OE

Protein	Gene names	Position	Protein.names	Score	Acetyl (K) Probabilities	Ratio WT/OE
Q9NRN7	AASDHPPT	91	L-aminoadipate-semialdehyde dehy	41.502	GK(1)PVLAK	0.6
Q9NY61	AATF	145	Protein AATF	168.5	SHSAK(1)TPGFSVQSISDFEK	Tip60 OE only
P61221	ABCE1	181	ATP-binding cassette sub-family E m	54.198	AAK(1)GTVGSILDR	0.03
P61221	ABCE1	419	ATP-binding cassette sub-family E m	52.725	ISPK(1)STGSVR	0.95
Q9NUJ1	ABHD10	101	Mycophenolic acid acyl-glucuronid	87.618	ALAIEEFCK(1)SLGHACIR	1.08
P42765	ACAA2	137	3-ketoacyl-CoA thiolase, mitochonc	44.203	FGTK(1)LGSDIK	0.88
P42765	ACAA2	209	3-ketoacyl-CoA thiolase, mitochonc	46.964	AANDAGYFNDEMAPIEVK(1)	Tip60 OE only
P11310	ACADM	279	Medium-chain specific acyl-CoA deh	60.518	VAMGAFDK(1)TR	0.96
P49748	ACADVL	276	Very long-chain specific acyl-CoA de	69.451	TPVTDPATGAVK(1)EK	Tip60 OE only
P24752	ACAT1	263	Acetyl-CoA acetyltransferase, mitocl	57.96	VDFSK(1)VPK	0.86
P24752	ACAT1	190	Acetyl-CoA acetyltransferase, mitocl	43.116	DGLTDVYNK(1)IHMGSCAEN	0.95
P24752	ACAT1	174	Acetyl-CoA acetyltransferase, mitocl	53.967	GSTPYGGVK(1)LEDLIVK	Tip60 OE only
Q9UKV3	ACIN1	861	Apoptotic chromatin condensation	121.02	K(1)PSISITTESLK	Tip60 OE only
P53396	ACLY	630	ATP-citrate synthase	91.914	GVTIIGPATVGGIK(1)PGCFK	0.91
O00154	ACOT7	286	Cytosolic acyl coenzyme A thioester	70.1	K(1)GCVITISGR	0.74
Q15067	ACOX1	512	Peroxisomal acyl-coenzyme A oxida	41.052	SK(1)EVAWNLTSVDLVR	1.06
Q15067	ACOX1	500	Peroxisomal acyl-coenzyme A oxida	54.416	LVEIAAK(1)NLQK	Tip60 OE only
P24666	ACP1	113	Low molecular weight phosphotyro	62.303	AK(1)IELLGSYDPQK	0.75
Q9NR19	ACSS2	418	Acetyl-coenzyme A synthetase, cyto	41.283	LLMK(1)FGDEPVTK	unquantifiable
P63261	ACTB;ACTBL2;ACTG1;A	328	Actin, cytoplasmic 1;Actin, cytoplas	126.41	IK(1)IIAPPER	0.74
P63261	ACTB;ACTG1	113	Actin, cytoplasmic 1;Actin, cytoplas	102.73	VAPEEHPVLLTEAPLNPK(1)A	0.66
P63261	ACTB;ACTG1;ACTA1;A(	326	Actin, cytoplasmic 1;Actin, cytoplas	73.781	EITALAPSTMK(1)IK	0.92

O43707	ACTN4	592	Alpha-actinin-4	49.35	EAILAIHK(1)EAQR	Tip60 OE only
O43707	ACTN4;ACTN1	417	Alpha-actinin-4;Alpha-actinin-1	49.418	LDHLAEK(1)FR	1.1
P23526	AHCY	408	Adenosylhomocysteinase	60.478	LTK(1)LTEK	0.89
P23526	AHCY	426	Adenosylhomocysteinase	61.641	QAQYLGMSCDGPFK(1)PDH	Tip60 OE only
O43865	AHCYL1	40	Putative adenosylhomocysteinase 2	62.408	K(1)QIQFADDMQEFTK	0.95
O95433	AHSA1	203	Activator of 90 kDa heat shock prote	61.593	PVGVK(1)IPTCK	Tip60 OE only
P15121	AKR1B1	179	Aldose reductase	50.387	YK(1)PAVNQIECHPYLTQEK	0.82
P54886	ALDH18A1	767	Delta-1-pyrroline-5-carboxylate syn	68.129	GK(1)DHVVSDFSEHGSLK	1.11
P54886	ALDH18A1	408	Delta-1-pyrroline-5-carboxylate syn	74.127	DEILLANK(1)K	1.19
P04075	ALDOA	230	Fructose-bisphosphate aldolase A	82.342	ALSDHHIYLEGTLLK(1)PNM\	0.71
P04075	ALDOA	108	Fructose-bisphosphate aldolase A	44.203	GGVVGIK(1)VDK	0.84
P04075	ALDOA	312	Fructose-bisphosphate aldolase A	74.162	ALQASALK(1)AWGGK	0.92
P04075	ALDOA	42	Fructose-bisphosphate aldolase A	44.299	GILAADESTGSIAK(1)R	1.01
P04075	ALDOA;ALDOC	147	Fructose-bisphosphate aldolase A;Fr	63.565	DGADFAK(1)WR	0.92
Q6P6C2	ALKBH5	321	RNA demethylase ALKBH5	51.092	LSGNNRDPALK(1)PK	0.09
Q6P6C2	ALKBH5	132	RNA demethylase ALKBH5	108.37	NK(1)YFFGEGYTYGAQLQK	1.06
Q4VCS5	AMOT	1051	Angiomotin	41.208	LSIPSLTCNPDK(1)TDGPVFH	0.61
Q9UM13	ANAPC10	169	Anaphase-promoting complex subu	49.5	IYTPVEESSIGK(1)FPR	unquantifiable
Q9UJX3	ANAPC7	263	Anaphase-promoting complex subu	44.318	AISTICSLEK(1)K	0.91
Q9NQW6	ANLN	482	Actin-binding protein anillin	59.542	HQGVSK(1)TQSLPVTEK	Tip60 OE only
P50995	ANXA11	255	Annexin A11	51.092	DLK(1)SELSGNFEK	0.86
O95782	AP2A1	177	AP-2 complex subunit alpha-1	72.958	LYK(1)ASPDLVPMGEWTAR	0.76
O00203	AP3B1	337	AP-3 complex subunit beta-1	41.283	SEAGIISK(1)SLVR	1.37
P27695	APEX1	7	DNA-(apurinic or apyrimidinic site) l	51.998	K(1)GAVAEDGDELR	1.12
P53365	ARFIP2	139	Arfaptin-2	59.8	K(1)YESVLQLGR	Tip60 OE only
P52565	ARHGDIA	141	Rho GDP-dissociation inhibitor 1	66.262	IDK(1)TDYMVGSYGPR	0.82
P52565	ARHGDIA	178	Rho GDP-dissociation inhibitor 1	61.962	GSYSIK(1)SR	1.1
Q9H993	ARMT1	40	Protein-glutamate O-methyltransfe	66.692	HK(1)SEFFEK	1.33
O15145	ARPC3	61	Actin-related protein 2/3 complex s	76.465	NYEIK(1)NEADR	Tip60 OE only
O15511	ARPC5	3	Actin-related protein 2/3 complex s	40.352	SK(1)NTVSSAR	Tip60 OE only
Q8N3C0	ASCC3	572	Activating signal cointegrator 1 cor	76.522	ELTGDMQLSK(1)SEILR	1.01
Q9UBL3	ASH2L	294	Set1/Ash2 histone methyltransferas	116.35	QSSAVSTSGNLNGGIAAGSSG	unquantifiable
P08243	ASNS	203	Asparagine synthetase [glutamine-h	62.14	VASVEMVK(1)YHHCR	1.06
Q6PL18	ATAD2	772	ATPase family AAA domain-containi	75.294	AK(1)DNFNFLHLNR	0.07

P54259	ATN1	66	Atrophin-1	57.598	VEEASTPK(1)VNK	Tip60 OE only
O00244	ATOX1	57	Copper transport protein ATOX1	152.54	K(0.966)TGK(0.034)TVSYLG	unquantifiable
P25705	ATP5A1	539	ATP synthase subunit alpha, mitoch	121.73	ISEQSDAK(1)LK	1.11
P06576	ATP5B	264	ATP synthase subunit beta, mitocho	55.841	DATSK(1)VALVYGQMNEPPG	0.7
P36542	ATP5C1	90	ATP synthase subunit gamma, mitoc	44.309	K(1)HLLIGVSSDR	Tip60 OE only
P24539	ATP5F1	162	ATP synthase F(0) complex subunit E	59.245	SQQALVQK(1)R	0.92
O75947	ATP5H	63	ATP synthase subunit d, mitochondr	76.655	ANVAK(1)AGLVDDFEK	0.8
O75947	ATP5H	99	ATP synthase subunit d, mitochondr	48.004	EDVK(1)SCAEWVSLSK	0.91
P18859	ATP5J	79	ATP synthase-coupling factor 6, mit	63.727	LK(1)QMFGNADMNTFPTFK	0.71
P48047	ATP5O	162	ATP synthase subunit O, mitochond	70.942	TVLK(1)SFLSQGQVLK	0.99
Q9UII2	ATPIF1	71	ATPase inhibitor, mitochondrial	54.549	EQLAALK(1)K	unquantifiable
Q9UII2	ATPIF1	82	ATPase inhibitor, mitochondrial	202.05	HHEEEIVHHK(1)K	unquantifiable
Q8WWM7	ATXN2L	348	Ataxin-2-like protein	50.607	EGK(1)YIPLPQR	Tip60 OE only
O15265	ATXN7	310	Ataxin-7	53.779	PTLPSPGQILNGK(1)GLPAPI	Tip60 OE only
Q92934	BAD	36	Bcl2-associated agonist of cell death	42.913	GLGPSPAGDGPSGSGK(1)HI	Tip60 OE only
Q9NRL2	BAZ1A	1383	Bromodomain adjacent to zinc finge	111.61	VIATK(1)SSEQSR	0.87
Q9UIG0	BAZ1B	1335	Tyrosine-protein kinase BAZ1B	159.02	APPVDDAEVDELVLQTK(1)R	0.5
Q9UIG0	BAZ1B	588	Tyrosine-protein kinase BAZ1B	59.542	YEDQELTGK(1)NLPAFR	0.82
Q9UIG0	BAZ1B	817	Tyrosine-protein kinase BAZ1B	145.25	VENGLGK(1)TDR	Tip60 OE only
Q9NYF8	BCLAF1	475	Bcl-2-associated transcription facto	43.382	ETGYVVERPSTTK(1)DK	0.04
Q9NYF8	BCLAF1	332	Bcl-2-associated transcription facto	145.29	SSFYPDGGDQETAK(1)TGK(1	0.04
Q9NYF8	BCLAF1	421	Bcl-2-associated transcription facto	61.375	SVLADQGK(1)SFATASHR	0.09
Q9NYF8	BCLAF1	593	Bcl-2-associated transcription facto	48.741	SIFDHIK(1)LPQASK	1.18
Q9NYF8	BCLAF1	335	Bcl-2-associated transcription facto	65.092	SSFYPDGGDQETAK(1)TGK(1	Tip60 OE only
Q9Y2W1	BCLAF1;THRAP3	876	Bcl-2-associated transcription facto	59.418	EEEWDPEYTPK(1)SK	Tip60 OE only
Q6W2J9	BCOR	618	BCL-6 corepressor	61.353	GAK(1)ASNPEPSFK	Tip60 OE only
P54132	BLM	1356	Bloom syndrome protein	52.555	TASSGSK(1)AK(1)GGSATCR	Tip60 OE only
P54132	BLM	1358	Bloom syndrome protein	52.555	TASSGSK(1)AK(1)GGSATCR	Tip60 OE only
P53004	BLVRA	269	Biliverdin reductase A	56.225	LLGQFSEK(1)ELAAEK	0.88
Q14692	BMS1	548	Ribosome biogenesis protein BMS1	41.092	AAGEGSK(1)AGLSPANCQSD	0.02
Q96IK1	BOD1;BOD1L1	79	Biorientation of chromosomes in ce	54.143	DCLADVDTK(1)PAYQNLR	0.4
O95861	BPNT1	49	3'(2'),5'-bisphosphate nucleotidase :	53.569	TCATDLQTK(1)ADR	Tip60 OE only
Q12830	BPTF	2743	Nucleosome-remodeling factor subu	49.418	LSALLFK(1)HK	0.96
P38398	BRCA1	450	Breast cancer type 1 susceptibility p	134.13	VHSK(1)SVESNIEDK	Tip60 OE only

P51587	BRCA2	268	Breast cancer type 2 susceptibility p	72.496	EAASHGFGK(1)TSGNSFK	Tip60 OE only
O95696	BRD1	333	Bromodomain-containing protein 1	45.997	QK(1)GVGACIQCHK	Tip60 OE only
O95696	BRD1	903	Bromodomain-containing protein 1	143.17	SELISCIENGNYAK(1)AAR	unquantifiable
O95696	BRD1	23	Bromodomain-containing protein 1	113.25	HPSSPCSVK(1)HSPTR	unquantifiable
O95696	BRD1	877	Bromodomain-containing protein 1	82.263	GK(1)PALVR	unquantifiable
P25440	BRD2	544	Bromodomain-containing protein 2	51.875	AVHEQLAALSQGPISK(1)PK	Tip60 OE only
O60885	BRD4	1218	Bromodomain-containing protein 4	49.929	HPTTPSSTAK(1)SSSDSFEQFF	Tip60 OE only
Q8TDN6	BRIX1	276	Ribosome biogenesis protein BRX1 h	134.44	SITAAK(1)YR	Tip60 OE only
P55201	BRPF1	1027	Peregrin	88.029	GK(1)PSFSR	unquantifiable
P55201	BRPF1	896	Peregrin	71.501	TSVLFSK(1)K	unquantifiable
Q9ULD4	BRPF3	91	Bromodomain and PHD finger-conta	52.555	ENSEQPQFPGK(1)SK(1)K	unquantifiable
Q9ULD4	BRPF3	105	Bromodomain and PHD finger-conta	144.82	ESCSK(1)HASGTSFHLPQPSF	unquantifiable
Q9ULD4	BRPF3	89	Bromodomain and PHD finger-conta	105.2	ENSEQPQFPGK(1)SK(1)K	unquantifiable
Q9BRD0	BUD13	427	BUD13 homolog	138.77	AAHMYSGAK(1)TGLVLTDIQ	Tip60 OE only
Q7L1Q6	BZW1	390	Basic leucine zipper and W2 domain	61.78	GK(1)SVFLEQMK	0.39
Q8N5I9	C12orf45	172	Uncharacterized protein C12orf45	72.652	GK(1)IEVLDSPASK	Tip60 OE only
Q9Y224	C14orf166	20	UPF0568 protein C14orf166	89.028	LTALDYHNPAGFNCK(1)DET	0.84
Q32NC0	C18orf21	157	UPF0711 protein C18orf21	48.794	GK(1)SPASVFR	unquantifiable
Q9BQ61	C19orf43	170	Uncharacterized protein C19orf43	57.136	AHQCGDDDK(1)TRPLVK	1.22
Q9BQ61	C19orf43	146	Uncharacterized protein C19orf43	79.82	TEDEVLTSK(1)GDAWAK	1.8
Q8N6N3	C1orf52	43	UPF0690 protein C1orf52	76.143	TPDPAK(1)SAGGCR	Tip60 OE only
Q07021	C1QBP	91	Complement component 1 Q subco	62.924	AFVDFLSDEIK(1)EER	Tip60 OE only
Q6NW34	C3orf17	486	Uncharacterized protein C3orf17	88.948	SATDTSAK(1)WR	Tip60 OE only
Q7Z6I8	C5orf24	17	UPF0461 protein C5orf24	77.19	MMHPVASSNPAFCGPGK(1)	unquantifiable
Q8N0T1	C8orf59	21	Uncharacterized protein C8orf59	62.303	NVFHIASQK(1)NFK	unquantifiable
P00918	CA2	18	Carbonic anhydrase 2	62.378	HNGPEHWHK(1)DFPIAK	0.77
Q9Y2D0	CA5B	249	Carbonic anhydrase 5B, mitochond	57.836	K(1)QPVEVDHDQLEQFR	unquantifiable
Q9Y376	CAB39	10	Calcium-binding protein 39	43.308	SHK(1)SPADIVK	0.97
Q9HB71	CACYBP	178	Calcyclin-binding protein	53.001	EK(1)PSYDTETDPSEGLMNVI	0.64
Q9HB71	CACYBP	146	Calcyclin-binding protein	66.435	VK(1)TDTVLILCR	1.08
P27708	CAD	2036	CAD protein;Glutamine-dependent	96.673	HPQPGAVELAAK(1)HCR	0.81
P27708	CAD	1411	CAD protein;Glutamine-dependent	52.579	LSSFVTK(1)GYR	1.09
P27708	CAD	2082	CAD protein;Glutamine-dependent	57.989	EELGTVNGMTITMVGDLK(1)	1.1
P27797	CALR	143	Calreticulin	45.68	K(1)VHVIFNYK	0.89

Q86VP6	CAND1	971	Cullin-associated NEDD8-dissociate	69.979	LK(1)GYLISGSSYAR	1.09
P27824	CANX	170	Calnexin	56.122	LLSK(1)TPELNLDQFHDK	1.05
Q9Y6Q1	CAPN6	616	Calpain-6	41.227	K(1)GGPTAK	unquantifiable
P04632	CAPNS1	172	Calpain small subunit 1	60.788	YLWNNIK(1)R	2.34
Q8NG31	CASC5	1221	Protein CASC5	63.816	GK(1)NLGVSFPK	Tip60 OE only
Q9UKL3	CASP8AP2	1059	CASP8-associated protein 2	56.563	AK(1)FSLIQFHR	Tip60 OE only
Q13185	CBX3	5	Chromobox protein homolog 3	56.729	ASNK(1)TTLQK	0.98
Q8N163	CCAR2	215	Cell cycle and apoptosis regulator p	74.162	AGGEPWGAK(0.924)K(0.07	0.03
Q96CT7	CCDC124	11	Coiled-coil domain-containing prot	44.863	FQGENTK(1)SAAAR	0.72
Q9H6F5	CCDC86	212	Coiled-coil domain-containing prot	59.356	GQHEPSKPPPAGETVTGGFG	0.16
P78371	CCT2	284	T-complex protein 1 subunit beta	78.653	ILK(1)HGINCFINR	0.83
P78371	CCT2	181	T-complex protein 1 subunit beta	43.084	DHFTK(1)LAVEAVLR	0.94
P78371	CCT2	154	T-complex protein 1 subunit beta	72.184	EALLSSAVDHGSDEVK(1)FR	0.95
P78371	CCT2	272	T-complex protein 1 subunit beta	82.417	VAEIEHAEK(1)EK	Tip60 OE only
P50991	CCT4	292	T-complex protein 1 subunit delta	96.342	K(1)TGCNVLLIQK	1.01
P50991	CCT4	288	T-complex protein 1 subunit delta	71.379	AYILNLVK(1)QIK	1.11
P50991	CCT4	375	T-complex protein 1 subunit delta	47.302	LLK(1)ITGCASPGK	Tip60 OE only
P40227	CCT6A	5	T-complex protein 1 subunit zeta	41.448	AAVK(1)TLNPK	0.61
P40227	CCT6A	199	T-complex protein 1 subunit zeta	78.653	HK(1)SETDTSLIR	0.64
P40227	CCT6A	129	T-complex protein 1 subunit zeta	87.298	EK(1)ALQFLEEVK	0.84
P40227	CCT6A	377	T-complex protein 1 subunit zeta	76.827	SVTLLIK(1)GPNK	0.9
P40227	CCT6A	251	T-complex protein 1 subunit zeta	55.452	TEVNSGFFYK(1)SAEER	0.91
P40227	CCT6A	365	T-complex protein 1 subunit zeta	55.567	FTFIEK(1)CNNPR	Tip60 OE only
Q99832	CCT7	366	T-complex protein 1 subunit eta	44.309	YNFFTGCPK(1)AK	0.91
Q99832	CCT7	77	T-complex protein 1 subunit eta	54.343	LLDVVHPAAK(1)TLVDIAK	1.05
Q99832	CCT7	109	T-complex protein 1 subunit eta	49.595	QVK(1)PYVEEGLHPQIIIR	Tip60 OE only
P50990	CCT8	7	T-complex protein 1 subunit theta	42.743	ALHVPK(1)APGFAQMLK	0.72
P50990	CCT8	326	T-complex protein 1 subunit theta	93.096	LCK(1)TVGATALPR	0.87
P50990	CCT8	400	T-complex protein 1 subunit theta	113.77	AVDDGVNTFK(1)VLTR	0.9
P50990	CCT8	466	T-complex protein 1 subunit theta	70.412	ANEVISK(1)LYAVHQEGNK	1.03
P50990	CCT8	152	T-complex protein 1 subunit theta	64.878	AHEILPNLVCCSAK(1)NLR	1.23
P50990	CCT8	16	T-complex protein 1 subunit theta	63.408	APGFAQMLK(1)EGAK	Tip60 OE only
Q16543	CDC37	78	Hsp90 co-chaperone Cdc37;Hsp90 c	97.214	ELEVAEGGK(1)AELER	0.81
Q16543	CDC37	154	Hsp90 co-chaperone Cdc37;Hsp90 c	49.418	TFVEK(1)YEK	0.96

O60508	CDC40	47	Pre-mRNA-processing factor 17	63.709	SPSSK(1)PSLAVAVDSAPEVA	Tip60 OE only
Q99459	CDC5L	294	Cell division cycle 5-like protein	57.159	SK(1)LVLPAPQISDAELQEVVI	Tip60 OE only
P06493	CDK1	6	Cyclin-dependent kinase 1	55.567	MEDYTK(1)IEK	1.06
P06493	CDK1	56	Cyclin-dependent kinase 1	47.198	EISLLK(1)ELR	1.11
Q14004	CDK13	677	Cyclin-dependent kinase 13	48.423	KTATQLHSK(1)R	0.05
Q03701	CEBPZ	13	CCAAT/enhancer-binding protein ze	40.002	EPLEFHAK(1)R	Tip60 OE only
Q9BXW7	CECR5	69	Cat eye syndrome critical region prc	42.599	VIPAALK(1)AFR	0.69
Q5JTW2	CEP78	317	Centrosomal protein of 78 kDa	43.544	SAK(1)SEYQWITSPSVK	Tip60 OE only
P23528	CFL1	132	Cofilin-1	61.375	LTGIK(1)HELQANCYEEVK	1.03
P23528	CFL1;CFL2	19	Cofilin-1;Cofilin-2	42.109	VFNDMK(1)VR	1.01
Q9UFW8	CGGBP1	16	CGG triplet repeat-binding protein :	45.077	SK(1)TALYVTPLDR	Tip60 OE only
Q14839	CHD4	969	Chromodomain-helicase-DNA-bindi	44.203	NMPSK(1)TELIVR	0.59
Q14839	CHD4	1206	Chromodomain-helicase-DNA-bindi	45.077	PGLGSK(1)TGSMSK	Tip60 OE only
Q9P2D1	CHD7	617	Chromodomain-helicase-DNA-bindi	62.14	GFGK(1)DDFPGGVDNQELN	unquantifiable
Q9H444	CHMP4B	14	Charged multivesicular body protei	66.621	LFGAGGGK(1)AGK	0.14
Q9Y3Y2	CHTOP	81	Chromatin target of PRMT1 protein	121.05	LGK(1)SNIQAR	Tip60 OE only
Q9Y3Y2	CHTOP	30	Chromatin target of PRMT1 protein	95.741	NK(1)QPTPVNIR	unquantifiable
Q96RK0	CIC	689	Protein capicua homolog	127.63	PVSSTPVPIASK(1)PFPTSGR	unquantifiable
Q8WWK9	CKAP2	187	Cytoskeleton-associated protein 2	60.434	GQIVQSK(1)INSFR	1.03
Q14008	CKAP5	1761	Cytoskeleton-associated protein 5	73.067	TLK(1)TLLHTLCK	1.36
Q14008	CKAP5	572	Cytoskeleton-associated protein 5	96.745	PAAPGGAGNTGTK(1)NK	Tip60 OE only
P12277	CKB	307	Creatine kinase B-type	49.418	HEK(1)FSEVLK	1.25
P12277	CKB	242	Creatine kinase B-type	87.639	VISMQK(1)GGNMK	1.31
Q9HAW4	CLSPN	891	Claspin	86.953	NQYQALK(1)PR	Tip60 OE only
Q9HAW4	CLSPN	925	Claspin	55.401	FTSQAEK(1)HLPR	unquantifiable
P09496	CLTA	242	Clathrin light chain A	49.418	SVLISLK(1)QAPLVH	1.05
Q00610	CLTC	367	Clathrin heavy chain 1	48.004	K(1)FNALFAQGNYSEAAK	1.19
Q00610	CLTC	1501	Clathrin heavy chain 1	57.348	LEK(1)HELIEFR	1.3
Q00610	CLTC;CLTCL1	456	Clathrin heavy chain 1;Clathrin heav	75.78	EDK(1)LECSEELGDLVK	1.59
O75153	CLUH	1144	Clustered mitochondria protein hor	56.482	ALK(1)VALSHHLVAR	1.21
Q8N1G2	CMTR1	108	Cap-specific mRNA (nucleoside-2'-O-	112.13	EGEGLGK(1)YSQGR	0.13
Q8N1G2	CMTR1	123	Cap-specific mRNA (nucleoside-2'-O-	54.608	DIVEASSQK(1)GR	Tip60 OE only
P62633	CNBP	103	Cellular nucleic acid-binding protei	46.408	EQCCYNCGK(1)PGHLAR	0.88
Q15417	CNN3	256	Calponin-3	45.359	VASQK(1)GMSVYGLGR	1.02

Q9H9A5	CNOT10	215	CCR4-NOT transcription complex su	64.121	AESGALIEAAK(1)SK	0.82
P53621	COPA	46	Coatomer subunit alpha;Xenin;Prox	96.64	MCTLIDK(1)FDEHDGPVR	0.92
P13073	COX4I1	53	Cytochrome c oxidase subunit 4 isof	103.85	DHPLPEVAHVK(1)HLSASQK	0.8
P13073	COX4I1	67	Cytochrome c oxidase subunit 4 isof	69.721	EK(1)ASWSSLSMDEK	0.99
P13073	COX4I1	87	Cytochrome c oxidase subunit 4 isof	42.336	FK(1)ESFAEMNR	1.12
Q16630	CPSF6	79	Cleavage and polyadenylation speci	47.288	GAAPNVVYTYTGK(1)R	0.03
Q8N684	CPSF7	397	Cleavage and polyadenylation speci	55.452	VLISSLK(1)DCLHGIEAK	Tip60 OE only
Q92793	CREBBP	1744	CREB-binding protein	88.974	MVK(1)WGLGLDDEGSSQGE	1.2
Q92793	CREBBP	1586	CREB-binding protein	80.534	EESTAASETTEGSQGDSK(1)N	1.4
Q92793	CREBBP	1595	CREB-binding protein	94.767	TNK(1)NK(1)SSISR	1.41
Q92793	CREBBP	1597	CREB-binding protein	94.767	TNK(1)NK(1)SSISR	1.41
Q92793	CREBBP	1583	CREB-binding protein	274.24	EESTAASETTEGSQGDSK(1)N	1.56
Q92793	CREBBP	1937	CREB-binding protein	42.582	TQPPTTVSTGK(1)PTSQVPAF	2.2
Q92793	CREBBP	1014	CREB-binding protein	90.334	TETQAEDTEPDPGESK(1)GEF	Tip60 OE only
O75390	CS	76	Citrate synthase, mitochondrial	136.51	GMK(1)GLVYETSVLDPDEGII	0.79
O75390	CS	450	Citrate synthase, mitochondrial	81.017	PK(1)SMSTEGLMK	1.09
P48729	CSNK1A1	8	Casein kinase I isoform alpha	72.34	ASSSGSK(1)AEFIVGGK	0.25
P68400	CSNK2A1	247	Casein kinase II subunit alpha	62.891	IAK(1)VLGTEDLYDYIDK	0.96
P68400	CSNK2A1;CSNK2A3	49	Casein kinase II subunit alpha;Casein	51.774	GK(1)YSEVFEAINITNNEK	unquantifiable
Q9H8E8	CSRP2BP	276	Cysteine-rich protein 2-binding prot	73.067	AQK(1)EAAGFLDR	Tip60 OE only
P04080	CSTB	91	Cystatin-B	50.04	AK(1)HDELTYF	0.98
P04080	CSTB	44	Cystatin-B	42.809	AVSFK(1)SQVVAGTNYFIK	Tip60 OE only
P56545	CTBP2	6	C-terminal-binding protein 2	47.849	ALVDK(1)HK	1.37
O60716	CTNND1	633	Catenin delta-1	51.013	GK(0.971)K(0.029)PIEDPAI	Tip60 OE only
Q6PD62	CTR9	637	RNA polymerase-associated protein	40.045	ALAIYK(1)QVLR	unquantifiable
Q14247	CTTN	198	Src substrate cortactin	68.893	GFGGK(1)YGIDK	Tip60 OE only
Q14247	CTTN	161	Src substrate cortactin	64.55	DYSSGFGGK(1)YGVQADR	Tip60 OE only
Q14247	CTTN	124	Src substrate cortactin	48.004	GFGGK(1)FGVQMDR	Tip60 OE only
Q13617	CUL2	393	Cullin-2	62.408	APELLAK(1)YCDNLLK	0.88
Q13618	CUL3	459	Cullin-3	116.77	LK(1)TECGCQFTSK	unquantifiable
Q13620	CUL4B	715	Cullin-4B	55.567	TFYLGK(1)HSGR	Tip60 OE only
Q9P013	CWC15	18	Spliceosome-associated protein CW	138.25	GK(1)GEGDLSQLSK	0.02
Q6UX04	CWC27	230	Peptidyl-prolyl cis-trans isomerase C	67.704	SK(1)SSHDLLK	Tip60 OE only
Q2TBE0	CWF19L2	382	CWF19-like protein 2	40.085	FLRPSDDEELSFHSK(1)GR	unquantifiable

Q9H5V9	CXorf56	128	UPF0428 protein CXorf56	45.077	FGQGFGK(1)TNIYTQK	Tip60 OE only
Q6NSI4	CXorf57	671	Uncharacterized protein CXorf57	172.23	ANINANLQGK(1)AR	Tip60 OE only
P00387	CYB5R3	42	NADH-cytochrome b5 reductase 3;N	86.814	STPAITLESPDIK(1)YPLR	0.82
P51397	DAP	29	Death-associated protein 1	94.297	IVQK(1)HPHTGDTK	unquantifiable
P14868	DARS	74	Aspartate--tRNA ligase, cytoplasmic	54.157	GK(1)QCFLVLR	Tip60 OE only
Q96EP5	DAZAP1	59	DAZ-associated protein 1	44.765	FK(1)DPNCVGTVLASR	0.7
P07108	DBI	19	Acyl-CoA-binding protein	113.83	TK(1)PSDEEMLFIYGHYK	0.96
P07108	DBI	77	Acyl-CoA-binding protein	99.802	AYINK(1)VEELK	1.07
P07108	DBI	55	Acyl-CoA-binding protein	102.06	AK(1)WDAWNELK	1.08
P11182	DBT	440	Lipoamide acyltransferase compone	50.563	GEVYK(1)AQIMNVSWSADH	unquantifiable
Q9NV06	DCAF13	426	DDB1- and CUL4-associated factor 1	50.149	HSK(1)PGSVPLVSEK	unquantifiable
Q92466	DDB2	278	DNA damage-binding protein 2	49.5	GK(1)ASFLYSLPHR	Tip60 OE only
Q9NVP1	DDX18	458	ATP-dependent RNA helicase DDX18	77.39	YHYELLNYIDLPVLAIHGK(1)(	0.58
Q9NR30	DDX21	668	Nucleolar RNA helicase 2	59.542	EQLGEEIDSK(1)VK	1.18
Q9NR30	DDX21	54	Nucleolar RNA helicase 2	44.188	TEEIAEEEETVFPK(1)AK	Tip60 OE only
Q13838	DDX39A;DDX39B	334	ATP-dependent RNA helicase DDX39	59.8	YQQFK(1)DFQR	1.41
Q13838	DDX39B	36	Spliceosome RNA helicase DDX39B	78.486	DVK(1)GSYVSIHSSGFR	0.41
O00571	DDX3X;DDX3Y	208	ATP-dependent RNA helicase DDX3X	53.995	YTRPTPVQK(1)HAIPIIK	1.28
Q86XP3	DDX42	50	ATP-dependent RNA helicase DDX42	40.254	LPQQSHSAFGATSSSSGFGK(	Tip60 OE only
Q7L014	DDX46	776	Probable ATP-dependent RNA helica	98.033	SSGFSGK(1)GFK	0.15
Q7L014	DDX46	1025	Probable ATP-dependent RNA helica	43.512	LQNSYQPTNK(1)GR	0.8
P17844	DDX5;DDX17	197	Probable ATP-dependent RNA helica	44.309	LK(1)STCIYGGAPK	1.07
P17844	DDX5;DDX17	207	Probable ATP-dependent RNA helica	46.606	STCIYGGAPK(1)GPQIR	Tip60 OE only
Q8N8A6	DDX51	601	ATP-dependent RNA helicase DDX51	42.001	AGK(1)TGQAFTLLLK	unquantifiable
Q96DF8	DGCR14	123	Protein DGCR14	47.919	EPPPPYVTPATFETPEVHAGT(	Tip60 OE only
Q96HY7	DHTKD1	818	Probable 2-oxoglutarate dehydroge	40.045	HFYSLVK(1)QR	1.24
Q08211	DHX9	14	ATP-dependent RNA helicase A	62.2	NFLYAWCGK(1)R	Tip60 OE only
Q9Y2L1	DIS3	815	Exosome complex exonuclease RRP4	57.598	LADICK(1)NLNFR	Tip60 OE only
Q9Y2L1	DIS3	281	Exosome complex exonuclease RRP4	46.891	EIILQGLK(1)HLNR	Tip60 OE only
O60832	DKC1	480	H/ACA ribonucleoprotein complex s	58.752	AK(1)AGLESGAEPGDGDSDT	Tip60 OE only
P10515	DLAT	466	Dihydrolipoyllysine-residue acetyltr	47.198	ELNK(1)ILEGR	0.81
P10515	DLAT	473	Dihydrolipoyllysine-residue acetyltr	51.268	SK(1)ISVNDFIIK	0.82
P09622	DLD	430	Dihydrolipoyl dehydrogenase, mito	61.353	AK(1)TNADTDGMVK	0.88
P09622	DLD	166	Dihydrolipoyl dehydrogenase, mito	78.486	NQVTATK(1)ADGGTQVIDTK	0.96

P09622	DLD	267	Dihydrolipoyl dehydrogenase, mito	70.268	ILQK(1)QGFK	0.99
P09622	DLD	143	Dihydrolipoyl dehydrogenase, mito	91.855	ALTGGIAHLFK(1)QNK	1.11
O60884	DNAJA2	152	DnaJ homolog subfamily A member	112.15	NVLCSACSGQGGK(1)SGAV(	Tip60 OE only
O75937	DNAJC8	146	DnaJ homolog subfamily C member	55.452	EGK(1)PTIVEEDDPELFK	0.48
P26358	DNMT1	341	DNA (cytosine-5)-methyltransferase	83.397	AK(1)TVMNSK(1)THPPK	Tip60 OE only
P26358	DNMT1	347	DNA (cytosine-5)-methyltransferase	83.397	AK(1)TVMNSK(1)THPPK	Tip60 OE only
Q5QJE6	DNTTIP2	10	Deoxynucleotidyltransferase termir	58.674	AK(1)ASIQAASAESSGQK	unquantifiable
Q9NRR4	DROSHA	380	Ribonuclease 3	49.35	WSDNQSSGK(1)DK	Tip60 OE only
P15924	DSP	940	Desmoplakin	42.599	NLHSEISGK(1)R	Tip60 OE only
P33316	DUT	179	Deoxyuridine 5'-triphosphate nucle	110.44	SGLAAK(1)HFIDVGAGVIDEI	0.29
Q14204	DYNC1H1	4283	Cytoplasmic dynein 1 heavy chain 1	78.934	SFDSEFK(1)LACK	1.1
P63167	DYNLL1	9	Dynein light chain 1, cytoplasmic	60.621	AVIK(1)NADMSEEMQQDSVI	0.88
Q56P03	EAPP	93	E2F-associated phosphoprotein	78.285	LSSLGTGSSSGNGK(1)VATAF	Tip60 OE only
Q13011	ECH1	65	Delta(3,5)-Delta(2,4)-dienoyl-CoA is	132.59	VTSAQK(1)HVLHVQLNRPNK	0.98
P30084	ECHS1	43	Enoyl-CoA hydratase, mitochondria	41.621	GK(1)NNTVGLIQLNRPK	0.58
O75521	ECI2	92	Enoyl-CoA delta isomerase 2, mitocl	66.262	AK(1)WDAWNALGSLPK	0.81
O75521	ECI2	161	Enoyl-CoA delta isomerase 2, mitocl	72.652	K(1)NAINTEMYHEIMR	0.87
O75521	ECI2	90	Enoyl-CoA delta isomerase 2, mitocl	55.567	PGVFDLINK(1)AK	Tip60 OE only
Q96F86	EDC3	346	Enhancer of mRNA-decapping prote	112.72	PTVALLCGPHVK(1)GAQGIS(	0.94
O60869	EDF1	25	Endothelial differentiation-related f	49.06	SK(1)QAILAAQR	unquantifiable
P68104	EEF1A1;EEF1A1P5	392	Elongation factor 1-alpha 1;Putative	52.79	KLEDGPK(1)FLK	0.9
P68104	EEF1A1;EEF1A1P5	439	Elongation factor 1-alpha 1;Putative	46.069	QTVAVGVIK(1)AVDK	1.35
P68104	EEF1A1;EEF1A1P5	408	Elongation factor 1-alpha 1;Putative	72.035	SGDAAIVDMVPGK(1)PMCV	1.72
P68104	EEF1A1;EEF1A1P5;EEF	255	Elongation factor 1-alpha 1;Putative	127.71	LPLQDVYK(1)IGGIGTVPVGF	1
P68104	EEF1A1;EEF1A1P5;EEF	44	Elongation factor 1-alpha 1;Putative	53.124	FEK(1)EAAEMGK	1.17
P68104	EEF1A1;EEF1A1P5;EEF	5	Elongation factor 1-alpha 1;Putative	54.355	EK(1)THINIVVIGHVDSGK	1.66
P24534	EEF1B2	78	Elongation factor 1-beta	99.451	ALGK(1)YGPADVEDTTGSGA	0.84
P26641	EEF1G	147	Elongation factor 1-gamma	161.17	ILGLLDAYLK(1)TR	0.97
P26641	EEF1G	434	Elongation factor 1-gamma	100.45	AFNQGK(1)IFK	1.11
P13639	EEF2	239	Elongation factor 2	75.739	FAAK(1)GEGQLGPAER	0.67
P13639	EEF2	337	Elongation factor 2	99.688	EGKPLLK(1)AVMR	0.8
P13639	EEF2	445	Elongation factor 2	84.213	KEDLYLK(1)PIQR	1.04
P13639	EEF2	275	Elongation factor 2	115.7	FSK(1)SATSPEGK	1.07
P13639	EEF2	42	Elongation factor 2	42.209	STLTDSLVCK(1)AGIIASAR	1.13

P13639	EEF2	426	Elongation factor 2	84.17	VFSGLVSTGLK(1)VR	1.22
P13639	EEF2	308	Elongation factor 2	59.8	VFDAIMNFK(1)K	1.3
P13639	EEF2	235	Elongation factor 2	44.309	QFAEMYVAK(1)FAAK	1.36
P13639	EEF2	439	Elongation factor 2	74.162	K(1)EDLYLKPIQR	Tip60 OE only
P13639	EEF2	594	Elongation factor 2	83.769	ETVSEESNVLCLSK(1)SPNK	Tip60 OE only
P13639	EEF2	272	Elongation factor 2	45.137	YFDPANGK(1)FSK	unquantifiable
Q15029	EFTUD2	646	116 kDa U5 small nuclear ribonucle	40.002	K(1)MYSEIDIK	1.22
P47813	EIF1AX;EIF1AY	88	Eukaryotic translation initiation fac	88.681	DYQDNK(1)ADVILK	0.92
Q99613	EIF3C;EIF3CL	643	Eukaryotic translation initiation fac	71.349	AK(1)ELLGQGLLLR	1.18
P60228	EIF3E	120	Eukaryotic translation initiation fac	70.17	MLFDYLADK(1)HGFR	1.02
Q9Y262	EIF3L	494	Eukaryotic translation initiation fac	65.252	IQLLVFK(1)HK	0.88
P60842	EIF4A1	146	Eukaryotic initiation factor 4A-I	132.44	AEVQK(1)LQMEAPHIIVGTP(	0.68
P60842	EIF4A1	309	Eukaryotic initiation factor 4A-I	76.655	DFTVSAMHGDMDQK(1)ER	0.94
P60842	EIF4A3;EIF4A1;EIF4A2	54	Eukaryotic initiation factor 4A-III;Eu	75.695	GIYAYGFEK(1)PSAIQQR	1.08
P06730	EIF4E	162	Eukaryotic translation initiation fac	68.44	GDK(1)IAIWTTECENR	Tip60 OE only
Q04637	EIF4G1	1074	Eukaryotic translation initiation fac	51.218	ITK(1)PGSIDSNNQLFAPGGF	Tip60 OE only
Q9GZV4	EIF5A2;EIF5A	47	Eukaryotic translation initiation fac	125.48	IVEMSTSK(1)TGK	4.39
Q9GZV4	EIF5A2;EIF5A;EIF5AL1	68	Eukaryotic translation initiation fac	81.431	K(1)YEDICPSTHNMDVPNIK	1.07
Q9GZV4	EIF5A2;EIF5A;EIF5AL1	67	Eukaryotic translation initiation fac	72.2	VHLVGIDIFTGK(1)K	2.64
Q8N8S7	ENAH	574	Protein enabled homolog	71.349	LK(1)EELIDAIR	1.33
Q8N8S7	ENAH	559	Protein enabled homolog	54.982	LK(1)QDILDEMR	Tip60 OE only
P06733	ENO1	202	Alpha-enolase	70.98	YGK(1)DATNVGDEGGFAPNI	0.74
P06733	ENO1	228	Alpha-enolase	72.006	EGLELLK(1)TAIGK	0.79
P06733	ENO1	60	Alpha-enolase	50.04	YMGK(1)GVSK	0.79
P06733	ENO1	89	Alpha-enolase	88.681	LNVTEQEK(1)IDK	0.91
P06733	ENO1	5	Alpha-enolase	129.4	SILK(1)IHAR	1
O43768	ENSA	47	Alpha-endosulfine	45.915	YPSLGQK(1)PGGSDFLMK	Tip60 OE only
Q09472	EP300	1570	Histone acetyltransferase p300	72.29	K(1)PGMPNVSNDLSQK	0.73
Q09472	EP300	1340	Histone acetyltransferase p300	66.27	TVEVK(1)PGMK(1)AR	0.93
Q09472	EP300	1542	Histone acetyltransferase p300	249.3	REENTSNESTDVTK(1)GDSK	1.14
Q09472	EP300	1336	Histone acetyltransferase p300	44.309	TVEVK(1)PGMK(1)AR	1.18
Q09472	EP300	1546	Histone acetyltransferase p300	249.3	EENTSNESTDVTK(1)GDSK(1	1.19
Q09472	EP300	1558	Histone acetyltransferase p300	123.79	TSK(1)NK(1)SSLSR	1.22
Q09472	EP300	1560	Histone acetyltransferase p300	123.79	TSK(1)NK(1)SSLSR	1.22

Q09472	EP300	1180	Histone acetyltransferase p300	74.364	LEFSPQTLCCYGK(1)QLCTIPI	1.39
Q09472	EP300;CREBBP	1674	Histone acetyltransferase p300;CREI	101.39	FVYTCNECK(1)HHVETR	1.02
Q09472	EP300;CREBBP	1590	Histone acetyltransferase p300;CREI	62.823	LYATMEK(1)HK	1.05
Q9Y2J2	EPB41L2;EPB41L3	405	Band 4.1-like protein 2;Band 4.1-lik	42.109	FLTLGSK(1)FR	0.7
Q52LR7	EPC2	23	Enhancer of polycomb homolog 2	74.46	GK(1)DMPDLNDCVSINR	unquantifiable
P07814	EPRS	497	Bifunctional glutamate/proline--tRI	60.961	IWAFNK(1)K	1.18
P07814	EPRS	788	Bifunctional glutamate/proline--tRI	63.426	QLLSLK(1)AEYK	1.32
P07814	EPRS	435	Bifunctional glutamate/proline--tRI	59.426	LNLNNTVLSK(1)R	1.39
P07814	EPRS	417	Bifunctional glutamate/proline--tRI	45.997	K(1)PYIWEYSR	1.59
Q96A65	EXOC4	18	Exocyst complex component 4	56.225	SK(1)DPSGLLISVIR	0.37
Q01780	EXOSC10	873	Exosome component 10	54.416	SMSFPTGK(1)SDR	Tip60 OE only
Q01780	EXOSC10	835	Exosome component 10	63.283	SK(1)VSSQFDPNK	unquantifiable
Q01469	FABP5	61	Fatty acid-binding protein, epiderm	43.991	TESTLK(1)TTQFSCTLGEK	0.81
Q9UK61	FAM208A	832	Protein FAM208A	54.7	DYEQPTCAK(1)VENAQFK	Tip60 OE only
Q5VWN6	FAM208B	2022	Protein FAM208B	46.66	ESPTQISIGAFPSTK(1)ISEAPI	Tip60 OE only
Q14320	FAM50A	275	Protein FAM50A	113.35	GK(1)SGPLFNFDVHDDVR	Tip60 OE only
Q14320	FAM50A;FAM50B	158	Protein FAM50A;Protein FAM50B	93.959	LGK(1)NPDVDTSFLPDR	Tip60 OE only
Q9NVI1	FANCI	1232	Fanconi anemia group I protein	77.379	EK(1)PAAVATAMAR	Tip60 OE only
P49327	FASN	2391	Fatty acid synthase;[Acyl-carrier-prc	41.283	VLEALLPLK(1)GLEER	0.68
P49327	FASN	2406	Fatty acid synthase;[Acyl-carrier-prc	72.958	VAAAVDLIIK(1)SHQGLDR	0.78
P49327	FASN	1239	Fatty acid synthase;[Acyl-carrier-prc	75.695	ACLDTAVENMPSLK(1)MK	0.78
P49327	FASN	1847	Fatty acid synthase;[Acyl-carrier-prc	81.548	YMAQGK(1)HIGK	0.8
P49327	FASN	436	Fatty acid synthase;[Acyl-carrier-prc	91.937	TPEAVQK(1)LLEQGLR	0.83
P49327	FASN	1072	Fatty acid synthase;[Acyl-carrier-prc	122.45	LYTLQDK(1)AQVADVVVSR	0.92
P49327	FASN	1704	Fatty acid synthase;[Acyl-carrier-prc	51.445	VFTTVGSAEK(1)R	0.99
P49327	FASN	1393	Fatty acid synthase;[Acyl-carrier-prc	58.37	K(1)SFYGSTLFLCR	1.01
P49327	FASN	41	Fatty acid synthase;[Acyl-carrier-prc	41.448	WK(1)AGLYGLPR	1.1
P49327	FASN	1151	Fatty acid synthase;[Acyl-carrier-prc	43.084	GLVQALQTK(1)VTQQGLK	1.57
P62861	FAU	51	40S ribosomal protein S30	125.48	FVNVVPTFGK(1)K	0.81
P22087	FBL	84	rRNA 2'-O-methyltransferase fibrilla	67.153	GNQSGK(1)NVMVEPHR	Tip60 OE only
Q86XK2	FBXO11	142	F-box only protein 11	60.918	VSGK(1)SQDLSAAPAEQYLQI	unquantifiable
P07954	FH	66	Fumarate hydratase, mitochondrial	54.143	VPNDK(1)YYGAQTVR	0.91
P07954	FH	61	Fumarate hydratase, mitochondrial	48.004	IEYDTFGELK(1)VPNDK	Tip60 OE only
Q13642	FHL1	144	Four and a half LIM domains proteir	58.457	QVIGTGSFFPK(1)GEDFYCVT	0.89

Q13642	FHL1	22	Four and a half LIM domains proteir	87.913	YVQK(1)DGHHCCLK	1.01
Q13642	FHL1	157	Four and a half LIM domains proteir	74.46	GEDFYCVTCHETK(1)FAK	1.02
Q13642	FHL1	4	Four and a half LIM domains proteir	47.823	AEK(1)FDCHYCR	Tip60 OE only
Q6UN15	FIP1L1	135	Pre-mRNA 3'-end-processing factor I	98.033	VYGTTGTK(1)VK	0.01
Q6UN15	FIP1L1	294	Pre-mRNA 3'-end-processing factor I	98.353	ANSSVGK(1)WQDR	Tip60 OE only
Q6UN15	FIP1L1	254	Pre-mRNA 3'-end-processing factor I	40.552	ETALPSTK(1)AEFTSPPSLFK	Tip60 OE only
Q02790	FKBP4	250	Peptidyl-prolyl cis-trans isomerase F	46.158	YELHLK(1)SFEK	0.98
Q02790	FKBP4	287	Peptidyl-prolyl cis-trans isomerase F	60.59	YK(1)QALLQYK	1.28
Q02790	FKBP4	390	Peptidyl-prolyl cis-trans isomerase F	45.137	AAK(1)TQLAVCQQR	unquantifiable
P21333	FLNA	578	Filamin-A	55.567	VGTECGNQK(1)VR	1.28
Q08050	FOXM1	614	Forkhead box protein M1	41.54	ETLPISSTPSK(1)SVLPR	Tip60 OE only
Q8IY81	FTSJ3	643	pre-rRNA processing protein FTSJ3	41.092	GPK(1)SDDDGFEIVPIEDPAK	Tip60 OE only
Q96I24	FUBP3	32	Far upstream element-binding prote	44.555	QIAAK(1)IDSIPHLNNSTPLVI	0.26
Q96QD9	FYTTD1	247	UAP56-interacting factor	41.188	TSTTNGGILTVSIDNPGAVQC	Tip60 OE only
P04406	GAPDH	194	Glyceraldehyde-3-phosphate dehyd	72.643	TVDGPSGK(1)LWR	0.78
P04406	GAPDH	219	Glyceraldehyde-3-phosphate dehyd	132.01	AVGK(1)VIPELNGK	0.87
P04406	GAPDH	263	Glyceraldehyde-3-phosphate dehyd	53.327	VVK(1)QASEGPLK	0.92
Q9NY12	GAR1	112	H/ACA ribonucleoprotein complex s	79.693	EQIGK(1)VDEIFGQLR	Tip60 OE only
Q92616	GCN1L1	436	Translational activator GCN1	43.282	AFSLK(1)TSTSAVR	1.24
P50395	GDI2	112	Rab GDP dissociation inhibitor beta	73.067	VTEGSFVYK(1)GGK	0.94
Q8TEQ6	GEMIN5	860	Gem-associated protein 5	79.466	SK(1)EELHQDCLVLATAK	1.2
Q96RP9	GFM1	710	Elongation factor G, mitochondrial	54.7	SCTEGK(1)GEYTMEYSR	1.18
Q969S9	GFM2	233	Ribosome-releasing factor 2, mitoch	43.512	TFK(1)GVVDVVMK	unquantifiable
O75223	GGCT	118	Gamma-glutamylcyclotransferase	49.5	VATQEGK(1)EITCR	Tip60 OE only
O95749	GGPS1	25	Geranylgeranyl pyrophosphate synt	49.813	YLLQLPGK(1)QVR	1.01
Q9NZM5	GLTSCR2	94	Glioma tumor suppressor candidate	68.893	LFFVDTGSK(1)EK	Tip60 OE only
P00367	GLUD1	480	Glutamate dehydrogenase 1, mitoch	43.696	FGK(1)HGGTIPIVPTAEFQDR	0.7
O75496	GMNN	50	Geminin	40.715	ENELSAGLSK(1)R	Tip60 OE only
Q9P2T1	GMPR2	291	GMP reductase 2	51.092	ASEGK(1)TVEVPFK	0.59
P49915	GMPS	389	GMP synthase [glutamine-hydrolyzi	55.656	AELIK(1)THHNDTELIR	1.22
P49915	GMPS	9	GMP synthase [glutamine-hydrolyzi	40.577	ALCNGDSK(1)LENAGGDLK	1.27
P63244	GNB2L1	130	Guanine nucleotide-binding proteir	41.283	TIK(1)LWNTLGVCK	1.08
P63244	GNB2L1	175	Guanine nucleotide-binding proteir	59.35	LVK(1)VWNLANCK	Tip60 OE only
Q13823	GNL2	55	Nucleolar GTP-binding protein 2	50.145	GK(1)IIKPLQYQSTVASGTVAI	Tip60 OE only

O15228	GNPAT	507	Dihydroxyacetone phosphate acyltr	44.309	K(1)EDVYSCFR	unquantifiable
P00505	GOT2	309	Aspartate aminotransferase, mitoch	71.223	VESQLK(1)ILIR	0.81
P00505	GOT2	296	Aspartate aminotransferase, mitoch	51.841	VGAFTMVCK(1)DADEAK	0.92
P00505	GOT2	404	Aspartate aminotransferase, mitoch	72.643	EFSIYMTK(1)DGR	Tip60 OE only
P00505	GOT2	159	Aspartate aminotransferase, mitoch	41.54	DVFLPK(1)PTWGNHTPIFR	Tip60 OE only
P00505	GOT2	185	Aspartate aminotransferase, mitoch	64.675	YYDPK(1)TCGFDFTGAVEDIS	Tip60 OE only
Q8N954	GPATCH11	116	G patch domain-containing protein	74.789	SGQALGK(1)SGGGIVEPIPLN	Tip60 OE only
Q5T3I0	GPATCH4	101	G patch domain-containing protein	78.934	YNHPK(1)PNLLYQK	0.09
Q9UKJ3	GPATCH8	61	G patch domain-containing protein	101.38	LGQGLGK(1)SLQGR	Tip60 OE only
P06744	GPI	252	Glucose-6-phosphate isomerase	95.573	HFVALSTNTTK(1)VK	1.13
Q92917	GPKOW	192	G patch domain and KOW motifs-co	94.302	TFNQVVK(1)PR	Tip60 OE only
Q8IYD1	GSPT1;GSPT2	337	Eukaryotic peptide chain release fac	49.189	EHAMLAK(1)TAGVK	unquantifiable
Q8IYD1	GSPT1;GSPT2	342	Eukaryotic peptide chain release fac	49.3	TAGVK(1)HLIVLINK	unquantifiable
P21266	GSTM3	128	Glutathione S-transferase Mu 3	48.741	LK(1)PQYLEELPGQLK	0.87
P78417	GSTO1	160	Glutathione S-transferase omega-1	76.827	LEEVLTNK(1)K	1.02
P35269	GTF2F1	435	General transcription factor IIF subu	200.55	LDTGPQSLSGK(1)STPQPPS(	0.18
P35269	GTF2F1	169	General transcription factor IIF subu	44.765	NK(1)VLNHFSIMQQR	Tip60 OE only
P13984	GTF2F2	71	General transcription factor IIF subu	71.86	TEVSFTLNEDLANIHDIGGK(1	0.05
P13984	GTF2F2	137	General transcription factor IIF subu	54.608	LQIEESSK(1)PVR	0.07
P78347	GTF2I	450	General transcription factor II-I	47.894	EDLQLDK(1)PASGVK	0.38
P78347	GTF2I	891	General transcription factor II-I	69.01	FGEAIGMGFPVK(1)VPYR	Tip60 OE only
P78347	GTF2I	221	General transcription factor II-I	51.546	VK(1)TEPTEDSGISLEMAAVT	Tip60 OE only
Q12789	GTF3C1	517	General transcription factor 3C poly	52.527	TQPHHSTPTK(1)GGWK	Tip60 OE only
Q12789	GTF3C1	507	General transcription factor 3C poly	65.47	ASANLRPK(1)TQPHHSTPTK	Tip60 OE only
Q12789	GTF3C1	724	General transcription factor 3C poly	42.317	VK(1)TSQPPVPQGEAEEDSQ	Tip60 OE only
Q9UKN8	GTF3C4	225	General transcription factor 3C poly	57.679	LSK(1)NEAPEGNLGDFAEFQI	Tip60 OE only
Q9Y5Q8	GTF3C5	462	General transcription factor 3C poly	98.105	SK(1)RPALFSSSAK	unquantifiable
Q9NYZ3	GTSE1	333	G2 and S phase-expressed protein 1	85.288	AK(1)SSEFASIPANSSR	Tip60 OE only
P07305	H1F0	12	Histone H1.0;Histone H1.0, N-termi	56.404	TENSTSAPAAK(1)PK	unquantifiable
Q71UI9	H2AFV;H2AFZ	116	Histone H2A.V;Histone H2A.Z	72.089	ATIAGGGVIPHIHK(1)SLIGK	1.25
Q71UI9	H2AFV;H2AFZ	12	Histone H2A.V;Histone H2A.Z	62.823	AGK(1)DSGK(1)AK	1.33
Q71UI9	H2AFV;H2AFZ	5	Histone H2A.V;Histone H2A.Z	73.781	AGGK(1)AGK(1)DSGK	1.35
Q71UI9	H2AFV;H2AFZ	8	Histone H2A.V;Histone H2A.Z	73.781	AGK(1)DSGK(1)AK	1.42
P16104	H2AFX	6	Histone H2AX	58.981	GK(1)TGGK(1)AR	unquantifiable

P16104	H2AFX	10	Histone H2AX	58.981	GK(1)TGGK(1)AR	unquantifiable
O75367	H2AFY	307	Core histone macro-H2A.1	51.092	LK(1)SIAFPSIGSGR	Tip60 OE only
P84243	H3F3A	28	Histone H3.3	47.706	K(1)SAPSTGGVK	1.11
P40939	HADHA	60	Trifunctional enzyme subunit alpha,	107.85	INSPNSK(1)VNTLSK	0.64
P40939	HADHA	406	Trifunctional enzyme subunit alpha,	113.44	GQQQVFK(1)GLNDK	0.91
P40939	HADHA	386	Trifunctional enzyme subunit alpha,	73.26	GLK(1)TILK	1.1
P40939	HADHA	644	Trifunctional enzyme subunit alpha,	55.567	GFYIYQEGVK(1)R	Tip60 OE only
P55084	HADHB	188	Trifunctional enzyme subunit beta,	43.808	LMLDLNK(1)AK	0.93
P51610	HCFC1	288	Host cell factor 1;HCF N-terminal ch	79.974	VATHEK(1)EWK	1
P51610	HCFC1	1163	Host cell factor 1;HCF N-terminal ch	56.482	ISVATGALEAAQGSK(1)SQCC	Tip60 OE only
P51610	HCFC1	129	Host cell factor 1;HCF N-terminal ch	47.823	TPK(1)NGPPPCPR	unquantifiable
Q13547	HDAC1	220	Histone deacetylase 1	69.954	GK(1)YYAVNYPLR	0.12
Q7Z4V5	HDGFRP2	483	Hepatoma-derived growth factor-re	47.198	LHSEIK(1)FALK	1.17
Q9BSH5	HDHD3	15	Haloacid dehalogenase-like hydrola	56.783	LLTWDVK(1)DTLLR	unquantifiable
Q9H583	HEATR1	1583	HEAT repeat-containing protein 1;H	41.283	ALLSK(1)AYDLLDK	1.16
Q6NVY1	HIBCH	353	3-hydroxyisobutyryl-CoA hydrolase,	42.001	AVLIDK(1)DQSPK	0.72
P49773	HINT1	21	Histidine triad nucleotide-binding p	123.63	PGGDTIFGK(1)IIR	0.73
P49773	HINT1	30	Histidine triad nucleotide-binding p	56.359	EIPAK(1)IIFEDDR	unquantifiable
P16402	HIST1H1D;HIST1H1C;H	47	Histone H1.3;Histone H1.2;Histone	40.025	ASGPPVSELITK(1)AVAASK	unquantifiable
P16402	HIST1H1D;HIST1H1C;H	91	Histone H1.3;Histone H1.2;Histone	125.74	SLVSK(1)GTLVQTK	unquantifiable
P16402	HIST1H1D;HIST1H1C;H	65	Histone H1.3;Histone H1.2;Histone	51.927	K(1)ALAAAGYDVEK	unquantifiable
P16402	HIST1H1D;HIST1H1C;H	76	Histone H1.3;Histone H1.2;Histone	52.928	ALAAAGYDVEK(1)NNSR	unquantifiable
Q93077	HIST1H2AC;HIST3H2A;	96	Histone H2A type 1-C;Histone H2A t	75.294	NDEELNK(1)LLGR	1.74
Q99878	HIST1H2AC;HIST3H2A;	6	Histone H2A type 1-C;Histone H2A t	166.17	GK(1)QGGK(1)AR	1.38
Q99878	HIST1H2AC;HIST3H2A;	10	Histone H2A type 1-C;Histone H2A t	166.17	GK(1)QGGK(1)AR	1.38
Q99878	HIST1H2AJ;HIST1H2AH	96	Histone H2A type 1-J;Histone H2A ty	85.377	NDEELNK(1)LLGK	1.62
Q16778	HIST1H2BC;H2BFS;HIS	109	Histone H2B type 1-C/E/F/G/I;Histor	123.02	LLLPGELAK(1)HAVSEGTK	0.9
Q16778	HIST1H2BC;H2BFS;HIS	117	Histone H2B type 1-C/E/F/G/I;Histor	103.91	HAVSEGTK(1)AVTK	1.32
Q16778	HIST1H2BC;H2BFS;HIS	47	Histone H2B type 1-C/E/F/G/I;Histor	63.408	VLK(1)QVHPDTGISSK	Tip60 OE only
P62807	HIST1H2BC;H2BFS;HIS	35	Histone H2B type 1-C/E/F/G/I;Histor	46.462	K(1)ESYSVYVYK	1.02
Q16778	HIST1H2BC;H2BFS;HIS	6	Histone H2B type 1-C/E/F/G/I;Histor	115.33	PEPAK(1)SAPAPK(1)K	0.59
Q16778	HIST1H2BC;H2BFS;HIS	12	Histone H2B type 1-C/E/F/G/I;Histor	115.33	SAPAPK(1)K(1)GSK	0.67
Q16778	HIST1H2BC;H2BFS;HIS	13	Histone H2B type 1-C/E/F/G/I;Histor	43.282	SAPAPK(1)K(1)GSK	0.72
Q16778	HIST1H2BC;H2BFS;HIS	17	Histone H2B type 1-C/E/F/G/I;Histor	143.45	K(1)AVTK(1)AQK	0.83

Q16778	HIST1H2BC;H2BFS;HIS	21	Histone H2B type 1-C/E/F/G/I;Histor	143.45	K(1)AVTK(1)AQK	1.03
P58876	HIST1H2BD	12	Histone H2B type 1-D	43.282	SAPAPK(1)K(1)GSK	0.67
P58876	HIST1H2BD	6	Histone H2B type 1-D	100.88	PEPTK(1)SAPAPK(1)K	0.69
P58876	HIST1H2BD	13	Histone H2B type 1-D	43.282	SAPAPK(1)K(1)GSK	0.73
Q99880	HIST1H2BL	6	Histone H2B type 1-L	173.71	PELAK(1)SAPAPK	0.65
Q99880	HIST1H2BL	12	Histone H2B type 1-L	43.282	SAPAPK(1)K(1)GSK	0.73
Q99877	HIST1H2BN	6	Histone H2B type 1-N	70.028	PEPSK(1)SAPAPK	0.7
Q99877	HIST1H2BN	12	Histone H2B type 1-N	43.282	SAPAPK(1)K(1)GSK	0.73
	HIST1H2BN;HIST1H2BL		Histone H2B type 1-N;Histone H2B t	43.282	SAPAPK(1)K(1)GSK	unquantifiable
P23527	HIST1H2BO;HIST2H2B	12	Histone H2B type 1-O;Histone H2B t	56.936	SAPAPK(1)K(1)GSK	0.72
P23527	HIST1H2BO;HIST2H2B	13	Histone H2B type 1-O;Histone H2B t	43.282	SAPAPK(1)K(1)GSK	0.72
P23527	HIST1H2BO;HIST2H2B	6	Histone H2B type 1-O;Histone H2B t	93.258	PDPAK(1)SAPAPK(1)K	0.72
P68431	HIST1H3A;HIST2H3A;H	80	Histone H3.1;Histone H3.2;Histone	78.655	EIAQDFK(1)TDLR	1.24
P68431	HIST1H3A;HIST2H3A;H	24	Histone H3.1;Histone H3.2;Histone	209.6	QLATK(1)AAR	1.13
P68431	HIST1H3A;HIST2H3A;H	19	Histone H3.1;Histone H3.2;Histone	209.6	K(1)QLATK(1)AAR	1.17
P68431	HIST1H3A;HIST2H3A;H	123	Histone H3.1;Histone H3.2;Histone	64.297	VTIMPK(1)DIQLAR	0.98
P68431	HIST1H3A;HIST2H3A;H	15	Histone H3.1;Histone H3.2;Histone	133.58	STGGK(1)APR	0.98
P68431	HIST1H3A;HIST2H3A;H	10	Histone H3.1;Histone H3.2;Histone	133.58	K(1)STGGK(1)APR	1.12
P68431	HIST1H3A;HIST2H3A;H	57	Histone H3.1;Histone H3.2;Histone	122.13	YQK(1)STELLIR	1.31
P68431	HIST1H3A;HIST2H3A;H	28	Histone H3.1;Histone H3.2;Histone	80.688	K(1)SAPATGGVK	1.5
P62805	HIST1H4A	78	Histone H4	65.347	DAVTYTEHAK(1)R	0.42
P62805	HIST1H4A	32	Histone H4	51.927	DNIQGITK(1)PAIR	0.82
P62805	HIST1H4A	17	Histone H4	127.56	GLGK(1)GGAK(1)R	0.88
P62805	HIST1H4A	9	Histone H4	99.442	GK(1)GGK(1)GLGK	0.92
P62805	HIST1H4A	6	Histone H4	99.442	GK(1)GGK(1)GLGK	0.94
P62805	HIST1H4A	13	Histone H4	127.56	GLGK(1)GGAK(1)R	1.02
P62805	HIST1H4A	80	Histone H4	43.308	K(1)TVTAMDVVYALK	1.13
P62805	HIST1H4A	92	Histone H4	117.67	TVTAMDVVYALK(1)R	1.14
Q99525	HIST1H4G	6	Histone H4-like protein type G	60.436	GK(1)AGK(1)GLGK	unquantifiable
Q99525	HIST1H4G	9	Histone H4-like protein type G	60.436	GK(1)AGK(1)GLGK	unquantifiable
Q5QNW6	HIST2H2BF	17	Histone H2B type 2-F	161.79	K(1)AVTK(1)VQK	0.95
Q5QNW6	HIST2H2BF	21	Histone H2B type 2-F	161.79	K(1)AVTK(1)VQK	1.12
Q16695	HIST3H3	24	Histone H3.1t	101.64	QLATK(1)VAR	1.14
Q16695	HIST3H3	19	Histone H3.1t	101.64	K(1)QLATK(1)VAR	1.54

Q8NCD3	HJURP	495	Holliday junction recognition prote	108.7	AK(1)SLSEAFENLGK	Tip60 OE only
Q8NCD3	HJURP	332	Holliday junction recognition prote	41.052	ENFIPCSEPVK(1)GTGALR	Tip60 OE only
P08397	HMBS	70	Porphobilinogen deaminase	41.283	ILDTALSK(1)IGEK	0.54
P17096	HMGA1	7	High mobility group protein HMG-I/	51.286	SESSSK(1)SSQPLASK	1.02
P35914	HMGCL	137	Hydroxymethylglutaryl-CoA lyase, m	41.283	K(1)NINCSIEESFQR	unquantifiable
P05114	HMGN1	61	Non-histone chromosomal protein I	175.15	GK(1)QAEVANQETK	0.33
P05114	HMGN1	14	Non-histone chromosomal protein I	88.441	VSSAEGAAK(1)EEPK	Tip60 OE only
P82970	HMGN5	48	High mobility group nucleosome-bi	62.378	TK(1)SDMMEENIDTSAQAVA	0.58
Q9UK76	HN1	148	Hematological and neurological exp	108.3	RNPPGGK(1)SSLVLG	0.34
Q9H910	HN1L	67	Hematological and neurological exp	116.43	TNPPGGK(1)GSGIFDESTPV(	0.4
Q9H910	HN1L	124	Hematological and neurological exp	53.569	DHVFLCEGEEPK(1)SDLK	Tip60 OE only
Q13151	HNRNPA0	96	Heterogeneous nuclear ribonucleop	72.138	PGAHAK(1)VK	0.02
P09651	HNRNPA1	3	Heterogeneous nuclear ribonucleop	197.07	SK(1)SESPKEPEQLR	0.03
P09651	HNRNPA1	350	Heterogeneous nuclear ribonucleop	99.522	SSGPYGGGGQYFAK(1)PR	0.06
P09651	HNRNPA1	166	Heterogeneous nuclear ribonucleop	42.913	IVIQK(1)YHTVNGHNCEVR	1.54
P22626	HNRNPA2B1	173	Heterogeneous nuclear ribonucleop	50.653	IVLQK(1)YHTINGHNAEVR	2.73
P07910	HNRNPC	176	Heterogeneous nuclear ribonucleop	91.937	SGFNSK(1)SGQR	0.02
P07910	HNRNPC	8	Heterogeneous nuclear ribonucleop	96.171	ASNVTNK(1)TDPR	Tip60 OE only
P07910	HNRNPC	170	Heterogeneous nuclear ribonucleop	91.937	GK(1)SGFNSK(1)SGQR	Tip60 OE only
P07910	HNRNPC	89	Heterogeneous nuclear ribonucleop	105	MIAGQVLDINLAAEPK(1)VNI	Tip60 OE only
P07910	HNRNPC;HNRNPCL4;H	39	Heterogeneous nuclear ribonucleop	77.379	SDVEAIFSK(1)YGK	0.81
P07910	HNRNPC;HNRNPCL4;H	29	Heterogeneous nuclear ribonucleop	54.608	VFIGNLNTLVVK(1)K	1.83
P07910	HNRNPC;HNRNPCL4;H	42	Heterogeneous nuclear ribonucleop	50.452	YGK(1)IVGCSVHK	unquantifiable
P07910	HNRNPC;RALYL	50	Heterogeneous nuclear ribonucleop	57.989	IVGCSVHK(1)GFAFVQYVNEI	0.66
Q14103	HNRNPD	231	Heterogeneous nuclear ribonucleop	64.199	GFCFITFK(0.992)EEEPVK(0.	1.02
Q14103	HNRNPD	243	Heterogeneous nuclear ribonucleop	48.794	K(1)YHNVGLSK	1.28
Q14103	HNRNPD	197	Heterogeneous nuclear ribonucleop	45.257	IFVGGLSPDTPEEK(1)IR	Tip60 OE only
P52597	HNRNPF	224	Heterogeneous nuclear ribonucleop	58.37	YIGIVK(1)QAGLER	Tip60 OE only
P31943	HNRNPH1	98	Heterogeneous nuclear ribonucleop	110.31	SNNVEMDWVLK(1)HTGPNS	1.07
P31942	HNRNPH3	76	Heterogeneous nuclear ribonucleop	47.853	EIAENALGK(1)HK	Tip60 OE only
P61978	HNRNPK	405	Heterogeneous nuclear ribonucleop	157.66	DLAGSIIGK(1)GGQR	0.04
P61978	HNRNPK	163	Heterogeneous nuclear ribonucleop	207.39	LLIHQSLAGGIIGVK(1)GAK	0.07
P61978	HNRNPK	461	Heterogeneous nuclear ribonucleop	124.59	QYSGK(1)FF	0.4
P61978	HNRNPK	219	Heterogeneous nuclear ribonucleop	72.652	IILDLISESPIK(1)GR	0.68

P61978	HNRNPK	52	Heterogeneous nuclear ribonucleop	84.17	ILLQSK(1)NAGAVIGK	1.46
P14866	HNRNPL	418	Heterogeneous nuclear ribonucleop	75.462	SK(1)PGAAMVEMADGYAVD	0.79
P14866	HNRNPL	248	Heterogeneous nuclear ribonucleop	86.291	AK(1)ASLNGADIYSGCCTLK	1.1
P52272	HNRNPM	698	Heterogeneous nuclear ribonucleop	61.435	GCGVVK(1)FESPEVAER	0.86
P52272	HNRNPM	651	Heterogeneous nuclear ribonucleop	79.116	K(1)ACQIFVR	1.46
O60506	HNRNPR;SYNCRIP	100	Heterogeneous nuclear ribonucleop	46.606	SAFLCGVMK(1)TYR	1.15
O60506	HNRNPR;SYNCRIP	576	Heterogeneous nuclear ribonucleop	45.137	ADGYNQPDSK(1)R	Tip60 OE only
Q00839	HNRNPU	186	Heterogeneous nuclear ribonucleop	116.43	EAAGK(1)SSGPTSLFAVTVAP	0.09
Q00839	HNRNPU	352	Heterogeneous nuclear ribonucleop	136.3	HLYTK(1)DIDIHEVR	0.27
Q00839	HNRNPU	635	Heterogeneous nuclear ribonucleop	104.52	DLPEHAVLK(1)MK	0.93
Q00839	HNRNPU	609	Heterogeneous nuclear ribonucleop	43.282	AVVVCPK(1)DEDYK	0.98
Q00839	HNRNPU	265	Heterogeneous nuclear ribonucleop	61.353	GYFEYIEENK(1)YSR	1.03
Q00839	HNRNPU	565	Heterogeneous nuclear ribonucleop	48.004	APQCLGK(1)FIEIAAR	1.14
Q00839	HNRNPU	387	Heterogeneous nuclear ribonucleop	86.814	GIK(1)TCNCETEDYGEK	1.16
Q00839	HNRNPU	516	Heterogeneous nuclear ribonucleop	148.62	TTWVTK(1)HAAENPGK	1.19
Q00839	HNRNPU	670	Heterogeneous nuclear ribonucleop	67.563	LLEQYK(1)EESK	1.22
Q00839	HNRNPU	592	Heterogeneous nuclear ribonucleop	59.8	K(1)MCLFAGFQR	1.41
Q00839	HNRNPU	270	Heterogeneous nuclear ribonucleop	48.697	AK(1)SPQPPVEEEDEHFDDT	Tip60 OE only
Q00839	HNRNPU	234	Heterogeneous nuclear ribonucleop	43.382	PGAPAAGDGK(1)TEQK	Tip60 OE only
P31274	HOXC9	119	Homeobox protein Hox-C9	59.908	HYALK(1)PDAYPGR	unquantifiable
Q5SSJ5	HP1BP3	160	Heterochromatin protein 1-binding	71.153	PK(1)MDAILTEAIK	Tip60 OE only
P51659	HSD17B4	565	Peroxisomal multifunctional enzym	52.555	FAK(1)PVYPGQTLQTEMWK	0.82
P51659	HSD17B4	184	Peroxisomal multifunctional enzym	79.92	K(1)SNIHCNTIAPNAGSR	0.89
P51659	HSD17B4	46	Peroxisomal multifunctional enzym	46.88	GALVVVNDLGGDFK(1)GVG	1.09
P07900	HSP90AA1	84	Heat shock protein HSP 90-alpha	72.891	ELHINLIPNK(1)QDR	0.8
P07900	HSP90AA1	478	Heat shock protein HSP 90-alpha	140.08	YYTSASGDEMVSLK(1)DYCT	1.14
P07900	HSP90AA1	489	Heat shock protein HSP 90-alpha	55.186	ENQK(1)HIYYITGETK	1.15
P07900	HSP90AA1	632	Heat shock protein HSP 90-alpha	178.12	K(1)HLEINPDHSIIETLR	Tip60 OE only
P07900	HSP90AA1;HSP90AA2	292	Heat shock protein HSP 90-alpha;He	51.927	YIDQEELNK(1)TK	1.03
P07900	HSP90AA1;HSP90AA2	185	Heat shock protein HSP 90-alpha;He	72.879	GTK(1)VILHLK	1.83
P07900	HSP90AA1;HSP90AA2	191	Heat shock protein HSP 90-alpha;He	67.456	VILHLK(1)EDQTEYLEER	2.2
P07900	HSP90AA1;HSP90AA4	539	Heat shock protein HSP 90-alpha;Pu	41.704	EFEGK(1)TLVSVTK	0.97
P07900	HSP90AA1;HSP90AA5	567	Heat shock protein HSP 90-alpha;Pu	69.423	TK(1)FENLCK	1.05
P07900	HSP90AA1;HSP90AA5	209	Heat shock protein HSP 90-alpha;Pu	50.088	K(1)HSQFIGYPITLFVEK	1.47

P07900	HSP90AA1;HSP90AB1;	407	Heat shock protein HSP 90-alpha;He	107.47	EMLQQSK(1)ILK	1.18
P07900	HSP90AA1;HSP90AB1;	362	Heat shock protein HSP 90-alpha;He	45.718	NNIK(1)LYVR	1.46
P08238	HSP90AB1	624	Heat shock protein HSP 90-beta	132.86	K(1)HLEINPDHPIVETLR	0.96
P08238	HSP90AB1;HSP90AB2I	559	Heat shock protein HSP 90-beta;Put	88.942	AK(1)FENLCK	1.34
P08238	HSP90AB1;HSP90AB3I	347	Heat shock protein HSP 90-beta;Put	46.158	APFDLFENK(1)K	1.03
P08238	HSP90AB1;HSP90AB3I	72	Heat shock protein HSP 90-beta;Put	50.284	ELK(1)IDIIPNPQER	1.18
P08238	HSP90AB1;HSP90AB3I	204	Heat shock protein HSP 90-beta;Put	56.959	K(1)HSQFIGYPITLYLEK	1.45
P08238	HSP90AB1;HSP90AB3I	531	Heat shock protein HSP 90-beta;Put	50.284	EFDGK(1)SLVSVTK	1.15
Q58FF6	HSP90AB4P	161	Putative heat shock protein HSP 90-	67.456	VILHLK(1)EDQTEYLEER	2
P14625	HSP90B1	142	Endoplasmin	42.041	EK(1)NLLHVTDTGVGMTR	1.18
P14625	HSP90B1	97	Endoplasmin	40.573	NK(1)EIFLR	1.68
P0DMV9	HSPA1B;HSPA1A	56	Heat shock 70 kDa protein 1B;Heat s	118.33	LIGDAAK(1)NQVALNPQNTV	0.53
P0DMV9	HSPA1B;HSPA1A	328	Heat shock 70 kDa protein 1B;Heat s	77.078	LDK(1)AQIHDLVLVGGSTR	1.31
P0DMV9	HSPA1B;HSPA1A	597	Heat shock 70 kDa protein 1B;Heat s	112.99	RK(1)ELEQVCNPIISGLYQGA	Tip60 OE only
P0DMV9	HSPA1B;HSPA1A;HSPA	507	Heat shock 70 kDa protein 1B;Heat s	46.158	ITITNDK(1)GR	1.65
P0DMV9	HSPA1B;HSPA1A;HSPA	500	Heat shock 70 kDa protein 1B;Heat s	108.98	ANK(1)ITITNDK	Tip60 OE only
P34932	HSPA4	557	Heat shock 70 kDa protein 4	72.937	AESEEMETSQAGSK(1)DK	0.35
P34932	HSPA4	221	Heat shock 70 kDa protein 4	54.143	LK(1)VLATAFDTTLGGR	1.03
P34932	HSPA4	53	Heat shock 70 kDa protein 4	60.157	SIGAAAK(1)SQVISNAK	1.03
P34932	HSPA4	135	Heat shock 70 kDa protein 4	76.262	K(1)PVVDCVVSVPCFYTDAEF	Tip60 OE only
P11021	HSPA5	326	78 kDa glucose-regulated protein	45.359	AK(1)FEELNMDLFR	1.36
P11021	HSPA5	81	78 kDa glucose-regulated protein	109.81	LIGDAAK(1)NQLTSNPENTVF	Tip60 OE only
P11142	HSPA8	56	Heat shock cognate 71 kDa protein	79.022	LIGDAAK(1)NQVAMNPTNT\	0.77
P11142	HSPA8	328	Heat shock cognate 71 kDa protein	105.38	LDK(1)SQIHDIVLVGGSTR	1.07
P11142	HSPA8;HSPA2	507	Heat shock cognate 71 kDa protein;I	46.158	ITITNDK(1)GR	0.79
P38646	HSPA9	187	Stress-70 protein, mitochondrial	54.658	ETAENYLGHTAK(1)NAVITVP	0.75
P38646	HSPA9	567	Stress-70 protein, mitochondrial	55.353	NAEK(1)YAEEDR	Tip60 OE only
P10809	HSPD1	72	60 kDa heat shock protein, mitocho	43.084	TVIIEQSWGSPK(1)VTK	0.73
P10809	HSPD1	301	60 kDa heat shock protein, mitocho	84.718	VGLQVVAVK(1)APGFGDNRI	0.76
P10809	HSPD1	359	60 kDa heat shock protein, mitocho	43.808	DDAMLLK(1)GK	0.88
P10809	HSPD1	125	60 kDa heat shock protein, mitocho	48.794	SIAK(1)EGFEK	1.12
P10809	HSPD1	82	60 kDa heat shock protein, mitocho	63.624	DGVTVAK(1)SIDLK	1.22
P61604	HSPE1	8	10 kDa heat shock protein, mitocho	92.439	K(1)FLPLFDR	0.66
P61604	HSPE1	56	10 kDa heat shock protein, mitocho	127.83	GK(1)GGEIQPVSVK	0.79

P61604	HSPE1	40	10 kDa heat shock protein, mitocho	80.706	SQGK(1)VLQATVVAVGSGSK	0.87
P61604	HSPE1	70	10 kDa heat shock protein, mitocho	43.544	VGDK(1)VLLPEYGGTK	Tip60 OE only
Q92598	HSPH1	95	Heat shock protein 105 kDa	40.577	ENLSYDLVPLK(1)NGGVGIK	Tip60 OE only
P42858	HTT	1512	Huntingtin	128.03	YHSK(1)QIIGIPK	0.04
Q7Z6Z7	HUWE1	2267	E3 ubiquitin-protein ligase HUWE1	97.551	IVNQPSSLFGSK(1)SASSK	0.07
Q7Z6Z7	HUWE1	1037	E3 ubiquitin-protein ligase HUWE1	43.282	SK(1)ITPAMAAR	Tip60 OE only
Q9NX55	HYPK	35	Huntingtin-interacting protein K	110.66	K(1)HDSGAADLER	1.06
P41252	IARS	1115	Isoleucine--tRNA ligase, cytoplasmic	51.927	LK(1)SVVTSIFGVK	1.26
Q9NSE4	IARS2	241	Isoleucine--tRNA ligase, mitochondr	87.298	SYK(1)PVFWSPSSR	0.89
P50213	IDH3A	77	Isocitrate dehydrogenase [NAD] sub	49.929	NVTAIQGPGGK(1)WMIPSEA	0.42
Q9NZI8	IGF2BP1	508	Insulin-like growth factor 2 mRNA-b	148.72	GGK(1)TVNELQNLTAAEVVV	Tip60 OE only
Q12906	ILF3	413	Interleukin enhancer-binding factor	53.493	LNQLK(1)PGLQYK	0.62
Q12906	ILF3	460	Interleukin enhancer-binding factor	69.148	LHVAVK(1)VLQDMGLPTGAI	0.86
Q12906	ILF3	332	Interleukin enhancer-binding factor	80.522	LAAFGQLHK(1)VLGMDPLPS	Tip60 OE only
P12268	IMPDH2	438	Inosine-5'-monophosphate dehydro	57.836	IK(1)VAQGVSGAVQDK	1.02
P12268	IMPDH2	257	Inosine-5'-monophosphate dehydro	89.013	QLLCGAAIGTHEDDK(1)YR	1.03
Q9NXR8	ING3	264	Inhibitor of growth protein 3	91.961	NNDFQLGK(1)EFSMAR	unquantifiable
Q9UNL4	ING4	129	Inhibitor of growth protein 4	94.122	QIESSDYDSSSSK(1)GK(1)K	unquantifiable
Q9UNL4	ING4	236	Inhibitor of growth protein 4	88.734	GK(1)WFCPR	unquantifiable
Q9UNL4	ING4	127	Inhibitor of growth protein 4	94.122	QIESSDYDSSSSK(1)GK(1)K	unquantifiable
Q9UNL4	ING4	146	Inhibitor of growth protein 4	137.4	SK(1)GK(1)NSDEEAPK	unquantifiable
Q9UNL4	ING4	148	Inhibitor of growth protein 4	137.4	SK(1)GK(1)NSDEEAPK	unquantifiable
Q8WYH8	ING5	224	Inhibitor of growth protein 5	48.796	PK(1)GK(1)WFCPR	unquantifiable
Q8WYH8	ING5	226	Inhibitor of growth protein 5	88.734	PK(1)GK(1)WFCPR	unquantifiable
P14616	INSRR;IGF1R;INSR	1151	Insulin receptor-related protein;Ins	51.854	GGK(1)GLLPVR	Tip60 OE only
O95373	IPO7	119	Importin-7	62.94	VQLTTCIHHIIK(1)HDYPSR	0.73
Q96P70	IPO9	277	Importin-9	65.305	AVTALVK(1)NFPK	1.05
P46940	IQGAP1	1027	Ras GTPase-activating-like protein I	52.278	LFK(1)TALQEEIK	Tip60 OE only
Q13576	IQGAP2	1524	Ras GTPase-activating-like protein I	83.265	SK(1)FLGVEMEK	unquantifiable
Q8IU81	IRF2BP1	73	Interferon regulatory factor 2-bindi	57.019	SPGPPALK(1)HPATK	Tip60 OE only
Q7Z5L9	IRF2BP2	289	Interferon regulatory factor 2-bindi	106.19	GPADSLSTAAGAAELSAEGA	Tip60 OE only
O14654	IRS4	586	Insulin receptor substrate 4	163	PGGGHGSGGGQGPGDGH	Tip60 OE only
O14654	IRS4	592	Insulin receptor substrate 4	163	PGGGHGSGGGQGPGDGH	Tip60 OE only
Q96CN7	ISOC1	286	Isochorismatase domain-containing	86.467	EIQNLIK(1)ASAPESGLLSK	0.73

Q6IE81	JADE1	302	Protein Jade-1	40.025	MEPITK(1)VSHIPSSR	unquantifiable
Q9NQC1	JADE2	32	Protein Jade-2	57.836	CSK(1)LPSSTK(1)SGWPR	unquantifiable
Q9NQC1	JADE2	637	Protein Jade-2	77.912	TWGQDAGSGK(1)GGQGPP[illegible]	unquantifiable
Q9NQC1	JADE2	38	Protein Jade-2	77.662	LPSSTK(1)SGWPR	unquantifiable
Q92613	JADE3	299	Protein Jade-3	44.299	MEPITK(1)ISHIPPSR	Tip60 OE only
Q92613	JADE3	38	Protein Jade-3	62.823	SK(1)IPNEHK(1)K	unquantifiable
Q92613	JADE3	812	Protein Jade-3	59.542	SK(1)THPLSHSSMQR	unquantifiable
Q92613	JADE3	638	Protein Jade-3	68.434	TPSSECYHGQSLGK(1)PLVLC	unquantifiable
Q92613	JADE3	601	Protein Jade-3	133.55	YPLESK(1)NNR	unquantifiable
Q92613	JADE3	30	Protein Jade-3	139.43	IK(1)SK(1)IPNEHK	unquantifiable
Q92613	JADE3	32	Protein Jade-3	139.43	SK(1)IPNEHK(1)K	unquantifiable
Q15652	JMJD1C	659	Probable JmjC domain-containing h	47.288	SK(1)ATYVNSQATGER	Tip60 OE only
Q15652	JMJD1C	676	Probable JmjC domain-containing h	49.5	LANK(1)IEHELSR	Tip60 OE only
Q92993	KAT5	80	Histone acetyltransferase KAT5	148.56	TPTK(1)NGLPGSR	Tip60 OE only
Q92993	KAT5	310	Histone acetyltransferase KAT5	161.63	NK(1)SYSQNLCLLAK	Tip60 OE only
Q92993	KAT5	282	Histone acetyltransferase KAT5	83.265	HLTK(1)CDLRHPPGNEIYR	Tip60 OE only
Q92993	KAT5	296	Histone acetyltransferase KAT5	91.937	K(1)GTISFFEIDGR	Tip60 OE only
Q92993	KAT5	230	Histone acetyltransferase KAT5	134.59	MK(1)NIECIELGR	Tip60 OE only
Q92993	KAT5	451	Histone acetyltransferase KAT5	44.415	SESGERPQITINEISEITSIK(1)K	Tip60 OE only
Q92993	KAT5	398	Histone acetyltransferase KAT5	143.02	VEGK(1)TGTPEKPLSDLGLLS	Tip60 OE only
Q92993	KAT5	52	Histone acetyltransferase KAT5	106.01	LFYVHYIDFNK(1)R	Tip60 OE only
Q92993	KAT5	498	Histone acetyltransferase KAT5	112.13	IDSK(1)CLHFTPK	Tip60 OE only
Q92993	KAT5	404	Histone acetyltransferase KAT5	45.115	TGTPEK(1)PLSDLGLLSYR	Tip60 OE only
Q92993	KAT5	321	Histone acetyltransferase KAT5	137.62	SYSQNLCLLAK(1)CFLDHK	Tip60 OE only
Q92993	KAT5	41	Histone acetyltransferase KAT5	44.203	K(1)LFYVHYIDFNK	Tip60 OE only
Q92993	KAT5	104	Histone acetyltransferase KAT5	79.346	EVPASAQASGK(1)TLPIPVQI	unquantifiable
Q92794	KAT6A	350	Histone acetyltransferase KAT6A	62.303	QNTVSK(1)GPFSK	unquantifiable
Q92794	KAT6A	815	Histone acetyltransferase KAT6A	96.64	ELEISVGK(1)SVSHENK	unquantifiable
Q8WYB5	KAT6B	223	Histone acetyltransferase KAT6B	59.306	ADPIPICSFCLGTK(1)ESNR	unquantifiable
Q8WYB5	KAT6B	379	Histone acetyltransferase KAT6B	61.703	TK(1)VCTTPSSGHAASGK(1)[illegible]	unquantifiable
Q8WYB5	KAT6B	650	Histone acetyltransferase KAT6B	113.52	VTPQMGTPSPGK(1)GSLTD[illegible]	unquantifiable
Q8WYB5	KAT6B	393	Histone acetyltransferase KAT6B	69.148	VCTTPSSGHAASGK(1)DSSS[illegible]	unquantifiable
Q8WYB5	KAT6B	590	Histone acetyltransferase KAT6B	53.327	SK(1)AHFFGK(1)R	unquantifiable
Q8WYB5	KAT6B	1928	Histone acetyltransferase KAT6B	51.819	TK(1)SASLSPAAATHQSQIYG[illegible]	unquantifiable

Q8WYB5	KAT6B	412	Histone acetyltransferase KAT6B	58.349	LAVTDPTRPGATTK(1)ITTTST	unquantifiable
Q8WYB5	KAT6B	584	Histone acetyltransferase KAT6B	58.981	SK(1)AHFFGK(1)R	unquantifiable
Q8WYB5	KAT6B	1044	Histone acetyltransferase KAT6B	66.246	VQSK(1)NK(1)YLHSPESR	unquantifiable
Q8WYB5	KAT6B	1038	Histone acetyltransferase KAT6B	172.8	QSPAK(1)VQSK(1)NK	unquantifiable
Q8WYB5	KAT6B	1115	Histone acetyltransferase KAT6B	54.539	LTK(1)PQSVAIK	unquantifiable
Q8WYB5	KAT6B	1042	Histone acetyltransferase KAT6B	172.8	VQSK(1)NK(1)YLHSPESR	unquantifiable
Q8WYB5	KAT6B;KAT6A	432	Histone acetyltransferase KAT6B;His	56.359	TK(1)GLIDGLTK	unquantifiable
Q9UGL1	KDM5B	832	Lysine-specific demethylase 5B	41.54	SGGGK(1)SQNQLTVNELR	Tip60 OE only
O15550	KDM6A	29	Lysine-specific demethylase 6A	79.771	MAAGK(1)ASGESEEASPSLTA	Tip60 OE only
Q14145	KEAP1	131	Kelch-like ECH-associated protein 1	101.3	EQGMEVVSIEGIHPK(1)VMEI	unquantifiable
Q15397	KIAA0020	33	Pumilio domain-containing protein	96.103	NSDSGSSK(1)TFPTR	0.21
Q7Z7F0	KIAA0907	91	UPF0469 protein KIAA0907	50.292	LQAPGK(1)GLTSNK	Tip60 OE only
Q69YN4	KIAA1429	1605	Protein virilizer homolog	67.456	HETFITSSGK(1)SEYIEPAK	Tip60 OE only
Q96Q89	KIF20B	497	Kinesin-like protein KIF20B	66.285	LFGPVK(1)SSQDVSLDSNSNS	Tip60 OE only
O00139	KIF2A	102	Kinesin-like protein KIF2A	69.979	TVASIK(1)NDPPSR	Tip60 OE only
Q9UMN6	KMT2B	1263	Histone-lysine N-methyltransferase 2	95.483	GSK(1)HLLECER	Tip60 OE only
Q14974	KPNB1	867	Importin subunit beta-1	88.681	TLATWATK(1)ELR	0.96
Q9P2J5	LARS	719	Leucine--tRNA ligase, cytoplasmic	43.382	MSK(1)STGNFLTLTQAIDK	1
Q15031	LARS2	600	Probable leucine--tRNA ligase, mito	45.359	LLAQGLIK(1)GQTFR	Tip60 OE only
Q9Y4W2	LAS1L	31	Ribosomal biogenesis protein LAS1L	81.016	GK(1)GSLPLSAHGIVVAWLSI	unquantifiable
Q14739	LBR	601	Lamin-B receptor	46.158	YGVAWEK(1)YCQR	1.28
Q14739	LBR	594	Lamin-B receptor	68.224	K(1)YGVAWEK	Tip60 OE only
P00338	LDHA	126	L-lactate dehydrogenase A chain	48.741	FIIPNVVK(1)YSPNCK	Tip60 OE only
P07195	LDHB	156	L-lactate dehydrogenase B chain	114.78	LSGLPK(1)HR	Tip60 OE only
Q9Y2U8	LEMD3	300	Inner nuclear membrane protein Ma	97.502	PLPPLTAK(1)SAGGR	Tip60 OE only
P30533	LRPAP1	287	Alpha-2-macroglobulin receptor-ass	57.859	HFEAK(1)IEK	unquantifiable
P42704	LRPPRC	750	Leucine-rich PPR motif-containing p	53.252	LDSSAVLDTGK(1)YVGLVR	0.99
P42704	LRPPRC	1347	Leucine-rich PPR motif-containing p	172.21	ALYEHLTAK(1)NTK	1.17
P42704	LRPPRC	649	Leucine-rich PPR motif-containing p	41.283	DAHLLVESK(1)NLDFQK	1.18
Q9NX58	LYAR	61	Cell growth-regulating nucleolar pro	74.92	YGGK(1)GYEGK	Tip60 OE only
P61326	MAGOH;MAGOHB	41	Protein mago nashi homolog;Protei	44.765	YANNSNYK(1)NDVMIR	unquantifiable
P61326	MAGOH;MAGOHB	14	Protein mago nashi homolog;Protei	57.559	YYVGHK(1)GK	unquantifiable
Q9BXY0	MAK16	296	Protein MAK16 homolog	84.289	AYVEIEYEQETEPVAK(1)AK	Tip60 OE only
Q92585	MAML1	822	Mastermind-like protein 1	40.589	GTLNPGLTK(1)PPVPR	unquantifiable

Q92585	MAML1	405	Mastermind-like protein 1	51.963	ALAGVVLPSQGPGGASELSSA	unquantifiable
Q15691	MAPRE1	148	Microtubule-associated protein RP/	58.487	QGQETAVAPSLVAPALNK(1)	Tip60 OE only
P31153	MAT2A	88	S-adenosylmethionine synthase isof	56.122	EAVK(1)HIGYDDSSK	1.2
P43243	MATR3	555	Matrin-3	56.011	K(1)ALWFQGR	0.96
P43243	MATR3	524	Matrin-3	47.849	IK(1)NYILMR	1.23
P43243	MATR3	3	Matrin-3	188.04	SK(1)SFQQSSLSR	Tip60 OE only
P43243	MATR3	473	Matrin-3	75.854	VHLSQK(1)YK	unquantifiable
Q05BQ5	MBTD1	131	MBT domain-containing protein 1	61.375	LAAYAQYQATLQNQAK(1)TK	unquantifiable
Q7L590	MCM10	313	Protein MCM10 homolog	43.991	VTPQSVNSGK(1)TFSIWK	Tip60 OE only
P33991	MCM4	858	DNA replication licensing factor MC	80.522	ALADDDFLTVTGK(1)TVR	0.05
P33992	MCM5	272	DNA replication licensing factor MC	63.283	VTIMGIYSIK(1)K	Tip60 OE only
Q14566	MCM6	422	DNA replication licensing factor MC	67.115	AVYTSGK(1)ASSAAGLTAAVV	0.78
Q14566	MCM6	643	DNA replication licensing factor MC	45.257	MHCCDEVQPK(1)HVK	Tip60 OE only
P40926	MDH2	185	Malate dehydrogenase, mitochondr	84.289	ANTFVAELK(1)GLDPAR	0.67
P40926	MDH2	78	Malate dehydrogenase, mitochondr	52.555	AAVK(1)GYLGPEQLPDCLK	0.7
P40926	MDH2	301	Malate dehydrogenase, mitochondr	74.941	GIEK(1)NLGIGK	0.74
P40926	MDH2	329	Malate dehydrogenase, mitochondr	58.981	K(1)GEDFVK	0.78
P40926	MDH2	335	Malate dehydrogenase, mitochondr	93.258	KGEDFVK(1)TLK	0.79
P40926	MDH2	307	Malate dehydrogenase, mitochondr	131.83	NLGIGK(1)VSSFEEK	0.86
P40926	MDH2	239	Malate dehydrogenase, mitochondr	81.865	IQEAGTEVVK(1)AK	1.09
P40926	MDH2	296	Malate dehydrogenase, mitochondr	76.262	SQETECTYFSTPLLLGK(1)K	Tip60 OE only
P40926	MDH2	157	Malate dehydrogenase, mitochondr	56.721	K(1)HGVYNPNK	unquantifiable
P23368	ME2	24	NAD-dependent malic enzyme, mitc	48.091	EK(0.998)GK(0.002)PLMLN	unquantifiable
Q9HAF1	MEAF6	6	Chromatin modification-related prc	86.313	AMHNK(1)AAPPQIPDTR	unquantifiable
Q9HAF1	MEAF6	74	Chromatin modification-related prc	78.324	YLTNQK(1)NSNSK(1)NDR	unquantifiable
Q9HAF1	MEAF6	69	Chromatin modification-related prc	178.22	YLTNQK(1)NSNSK(1)NDR	unquantifiable
Q15648	MED1	1095	Mediator of RNA polymerase II trans	111.23	PSSHSQYTSSGSVSSSGSK(1)S	Tip60 OE only
Q15648	MED1	1076	Mediator of RNA polymerase II trans	83.701	GTVMVGK(1)PSSHSQYTSSG	unquantifiable
Q15648	MED1	1152	Mediator of RNA polymerase II trans	41.092	NSSQSGGK(1)PGSSPITK	unquantifiable
Q02078	MEF2A	249	Myocyte-specific enhancer factor 2A	56.599	ASPNLIGATGANSLGK(1)VM	unquantifiable
P55081	MFAP1	381	Microfibrillar-associated protein 1	44.447	VMQVK(1)NFGR	0.46
P55081	MFAP1	390	Microfibrillar-associated protein 1	73.109	TK(1)YTHLVDQDTTSFDSAW	Tip60 OE only
Q8IWI9	MGA	1448	MAX gene-associated protein	63.727	LHGGK(1)GLPFYAGLSPAGK	Tip60 OE only
Q8IWI9	MGA	1207	MAX gene-associated protein	66.621	LLTGIK(1)SPR	unquantifiable

P14174	MIF	78	Macrophage migration inhibitory fa	57.019	SYSK(1)LLCGLLAER	1.09
P46013	MKI67	2187	Antigen KI-67	42.743	GK(1)AQPLEDLAGLK	Tip60 OE only
P46013	MKI67	993	Antigen KI-67	131.37	GQNLLQTQDHAK(1)APK	Tip60 OE only
P46013	MKI67	404	Antigen KI-67	80.534	TPAK(1)VEDAADSATKPENLS	Tip60 OE only
P46013	MKI67	1208	Antigen KI-67	51.927	LDLAGTLPGSK(1)R	Tip60 OE only
P46013	MKI67	1938	Antigen KI-67	50.284	LDLLGNLPGSK(1)R	Tip60 OE only
P46013	MKI67	1694	Antigen KI-67	40.589	QILDSAASLTGSK(1)R	Tip60 OE only
P46013	MKI67	1178	Antigen KI-67	52.79	AMLTPK(1)PAGGDEK	Tip60 OE only
P46013	MKI67	2024	Antigen KI-67	49.5	LTQTSGK(1)TTQTHR	Tip60 OE only
P46013	MKI67	1367	Antigen KI-67	48.112	ELFQTPGHTEEAVAAGK(1)TT	Tip60 OE only
P46013	MKI67	2236	Antigen KI-67	59.185	SPQPDPVGTPTIFK(1)PQSK	Tip60 OE only
P46013	MKI67	1245	Antigen KI-67	55.885	ELFQTPGHTEELVAAGK(1)TT	Tip60 OE only
P52815	MRPL12	150	39S ribosomal protein L12, mitoch	41.54	EIK(1)NYIQGINLVQAK	1.25
Q9BZE1	MRPL37	127	39S ribosomal protein L37, mitoch	44.203	TK(1)LIEGLPEK	1.19
Q9NYK5	MRPL39	72	39S ribosomal protein L39, mitoch	73.848	HVGK(1)TDPGTVFVMNK	0.93
Q9HD33	MRPL47	144	39S ribosomal protein L47, mitoch	133.71	VVDSMDALDK(1)VVQER	0.99
Q9Y3D9	MRPS23	93	28S ribosomal protein S23, mitoch	55.426	AFDLFNPNFK(1)STCQR	0.69
P82933	MRPS9	287	28S ribosomal protein S9, mitochor	72.652	AEAIVYK(1)HGSGR	0.87
P43246	MSH2	73	DNA mismatch repair protein Msh2	89.507	YMGPAGAK(1)NLQSVVLSK	0.06
Q96DY7	MTBP	739	Mdm2-binding protein	56.563	SK(1)DLNCLYPR	Tip60 OE only
P11586	MTHFD1	10	C-1-tetrahydrofolate synthase, cyto	72.958	APAEILNGK(1)EISAQIR	1.01
P11586	MTHFD1	553	C-1-tetrahydrofolate synthase, cyto	64.55	ITIGQAPTEK(1)GHTR	unquantifiable
Q6UB35	MTHFD1L	189	Monofunctional C1-tetrahydrofolat	69.979	DVDGVTDINLGK(1)LVR	0.77
P13995	MTHFD2	286	Bifunctional methylenetetrahydrof	66.27	VHDPVTAK(1)PK	0.95
P58546	MTPN	66	Myotrophin	62.7	GADINAPDK(1)HHITPLLSA\	0.73
P58546	MTPN	24	Myotrophin	64.297	DYVAK(1)GEDVNR	Tip60 OE only
Q9BQG0	MYBBP1A	1289	Myb-binding protein 1A	56.404	GVLGK(1)SPLSALAR	0.14
Q9BQG0	MYBBP1A	1219	Myb-binding protein 1A	63.091	AK(1)VPAQANGTPTTK	Tip60 OE only
O75592	MYCBP2	2731	E3 ubiquitin-protein ligase MYCBP2	50.207	PASTSGK(1)SELSSK	Tip60 OE only
P35580	MYH10;MYH11	1532	Myosin-10;Myosin-11	45.161	NVHELEK(1)SK	1.03
P35579	MYH9	1024	Myosin-9	80.979	NK(1)HEAMITDLEER	0.61
P35579	MYH9	1441	Myosin-9	65.535	QSACNLEK(1)K	0.93
P35579	MYH9	1525	Myosin-9	75.378	SVHELEK(1)SK	1.15
P60660	MYL6	81	Myosin light polypeptide 6	87.001	NK(1)DQGTYEDYVEGLR	0.91

Q147X3	NAA30	104	N-alpha-acetyltransferase 30	98.694	SK(1)VLSVAEVAATTATPDGG	unquantifiable
Q147X3	NAA30	233	N-alpha-acetyltransferase 30	133.32	LITK(1)DLSEPYSIYTYR	unquantifiable
Q9GZZ1	NAA50	34	N-alpha-acetyltransferase 50	176.18	LNQVIFPVSYNDK(1)FYK	0.86
Q9GZZ1	NAA50	37	N-alpha-acetyltransferase 50	111.63	FYK(1)DVLEVGELAK	0.96
E9PAV3	NACA	2005	Nascent polypeptide-associated con	135.09	IEDLSQQAQLAAAEK(1)FK	0.64
E9PAV3	NACA;NACAP1	1963	Nascent polypeptide-associated con	97.953	SK(1)NILFVITKPDVYK	0.7
E9PAV3	NACA;NACAP1	1971	Nascent polypeptide-associated con	79.693	NILFVITK(1)PDVYK	0.9
Q96RE7	NACC1	167	Nucleus accumbens-associated prot	45.257	VK(1)TEQQESDSVQCMPVAK	Tip60 OE only
P55209	NAP1L1	194	Nucleosome assembly protein 1-like	64.723	NVDLLSDMVQEHDEPILK(1)	1.17
P55209	NAP1L1	82	Nucleosome assembly protein 1-like	78.334	VNALK(1)NLQVK	1.35
P49321	NASP	698	Nuclear autoantigenic sperm protei	49.089	K(1)PTDGASSSNCVTDISHLV	0.59
Q9H0A0	NAT10	426	N-acetyltransferase 10	66.435	SLSLK(1)LIQQLR	1.09
O60934	NBN	334	Nibrin	48.694	NYCDPQGHPSTGLK(1)TTTP	Tip60 OE only
Q6IBW4	NCAPH2	358	Condensin-2 complex subunit H2	66.871	GAAK(1)LQDFHQWYLAAYAI	Tip60 OE only
P19338	NCL	429	Nucleolin	81.338	SK(1)GIAYIEFK	0.1
P19338	NCL	610	Nucleolin	102.28	ETGSSK(1)GFGFVDFNSEEDA	0.25
P19338	NCL	116	Nucleolin	78.692	GATPGK(1)ALVATPGK	0.31
P19338	NCL	398	Nucleolin	47.823	TLLAK(1)NLPYK	0.44
P19338	NCL	577	Nucleolin	79.466	TLFVK(1)GLSEDTTEETLK	0.55
P19338	NCL	403	Nucleolin	42.336	NLPYK(1)VTQDELK	0.81
P19338	NCL	545	Nucleolin	69.825	EALNSCNK(1)R	1.16
P19338	NCL	477	Nucleolin	132.88	GGK(1)NSTWSGESK	Tip60 OE only
P19338	NCL	646	Nucleolin	57.96	VTLDWAK(1)PK	Tip60 OE only
P19338	NCL	333	Nucleolin	68.83	TGISDVFAK(1)NDLAVVDVR	Tip60 OE only
Q15596	NCOA2	785	Nuclear receptor coactivator 2	48.283	LIAMK(1)TEK	unquantifiable
O75376	NCOR1	1336	Nuclear receptor corepressor 1	51.276	GK(1)PYDGITTIK	unquantifiable
Q9Y618	NCOR2	1535	Nuclear receptor corepressor 2	43.282	GAPVIVPELGK(1)PR	Tip60 OE only
Q9Y618	NCOR2	2399	Nuclear receptor corepressor 2	61.353	SDHTLTSPGGGGK(1)AK	Tip60 OE only
Q8NCF5	NFATC2IP	129	NFATC2-interacting protein	68.034	LVLDPGEAPLVPVYSGK(1)VK	Tip60 OE only
Q6P4R8	NFRKB	671	Nuclear factor related to kappa-B-bi	69.954	IHQAQAAAAK(1)AR	unquantifiable
Q6P4R8	NFRKB	1237	Nuclear factor related to kappa-B-bi	176.68	LIAGNK(1)PVSFLTAQQLQQL	unquantifiable
P30414	NKTR	639	NK-tumor recognition protein;Puta	49.12	AK(1)TTHLLPIQSTYSLANIK	Tip60 OE only
Q9C000	NLRP1	583	NACHT, LRR and PYD domains-conta	50.04	K(1)TLFSPDDLRK	unquantifiable
P22392	NME1;NME2	12	Nucleoside diphosphate kinase A;Nu	74.162	TFIAIK(1)PDGVQR	1.34

P22392	NME1;NME2;NME2P1	100	Nucleoside diphosphate kinase A;Nu	41.052	VMLGETNPADSK(1)PGTIR	0.87
P22392	NME2;NME2P1	49	Nucleoside diphosphate kinase B;Pu	47.545	ASEEHLK(1)QHYIDLK	1.52
P22392	NME2;NME2P1	124	Nucleoside diphosphate kinase B;Pu	96.745	NIIHGSDSVK(1)SAEK	Tip60 OE only
Q13423	NNT	768	NAD(P) transhydrogenase, mitochor	57.019	LQGLLK(1)SAPLLLPGR	0.69
Q76FK4	NOL8	266	Nucleolar protein 8	102.52	TCDSITPSK(1)SSPVPVSDTQI	Tip60 OE only
Q5SY16	NOL9	376	Polynucleotide 5'-hydroxyl-kinase N	56.729	MVYYGK(1)PSCK	1.1
Q14978	NOLC1	579	Nucleolar and coiled-body phospho	40.727	AAVVVSK(1)SGSLK	0.15
Q14978	NOLC1	460	Nucleolar and coiled-body phospho	48.004	AALSLPAK(1)QAPQGSR	0.19
Q14978	NOLC1	347	Nucleolar and coiled-body phospho	50.284	AVVSK(0.981)ATTK(0.019)F	0.21
Q14978	NOLC1	505	Nucleolar and coiled-body phospho	99.5	VAGGAAPSK(1)PASAK	0.23
Q14978	NOLC1	193	Nucleolar and coiled-body phospho	47.302	ITPVTVK(1)AQTK	0.3
Q14978	NOLC1	76	Nucleolar and coiled-body phospho	139.88	LQANGPVAK(1)K	0.41
Q14978	NOLC1	33	Nucleolar and coiled-body phospho	42.001	DNQLSEVANK(1)FAK	Tip60 OE only
Q14978	NOLC1	446	Nucleolar and coiled-body phospho	53.166	MVATTK(1)PK	Tip60 OE only
Q15233	NONO	190	Non-POU domain-containing octam	46.592	GRPSGK(1)GIVEFSGK	0.2
Q15233	NONO	198	Non-POU domain-containing octam	113.22	GIVEFSGK(1)PAAR	0.54
Q15233	NONO	5	Non-POU domain-containing octam	76.143	MQSNK(1)TFNLEK	0.69
Q15233	NONO	243	Non-POU domain-containing octam	124.08	LVIK(1)NQQFHK	0.84
Q15233	NONO	99	Non-POU domain-containing octam	63.283	YGK(1)AGEVFIHK	0.92
P78316	NOP14	643	Nucleolar protein 14	65.395	ALGK(1)NSELLVVSAR	Tip60 OE only
P46087	NOP2	92	Probable 28S rRNA (cytosine(4447)-	43.77	K(1)GPQSLFNAPR	0.64
P46087	NOP2	685	Probable 28S rRNA (cytosine(4447)-	91.032	TQASSSFQDSSQPAGK(1)AE(	Tip60 OE only
P46087	NOP2	655	Probable 28S rRNA (cytosine(4447)-	57.159	LNGISK(1)GADSELSTVPSVTI	Tip60 OE only
O00567	NOP56	240	Nucleolar protein 56	42.001	LEELTMDGAK(1)AK	0.51
Q9Y2X3	NOP58	365	Nucleolar protein 58	47.823	MLAAK(1)TVLAIR	0.91
Q9Y2X3	NOP58	467	Nucleolar protein 58	89.029	VK(1)VEEEEEK	Tip60 OE only
Q9Y314	NOSIP	6	Nitric oxide synthase-interacting pr	56.339	HGK(1)NCTAGAVYTYHEK	0.22
Q14207	NPAT	543	Protein NPAT	40.725	SSQLLSQDTSLTGK(1)PSK	unquantifiable
P06748	NPM1	150	Nucleophosmin	67.704	SAPGGGSK(1)VPQK	0.04
P06748	NPM1	141	Nucleophosmin	113.7	LLSISGK(1)R	0.07
P06748	NPM1	239	Nucleophosmin	142.97	TPK(1)GPSSVEDIK	0.11
P06748	NPM1	212	Nucleophosmin	102.87	SNQNGK(0.997)DSK(0.003	0.13
P06748	NPM1	223	Nucleophosmin	81.548	SK(1)GQESFK	0.13
P06748	NPM1	257	Nucleophosmin	58.676	MQASIEK(1)GGSLPK	0.35

P06748	NPM1	267	Nucleophosmin	122.33	VEAK(1)FINYVK	0.61
P06748	NPM1	248	Nucleophosmin	40.715	GPSSVEDIK(1)AK	0.98
P06748	NPM1	32	Nucleophosmin	196.31	DYHFK(1)VDNDENEHQLSLR	0.98
P06748	NPM1	273	Nucleophosmin	96.253	FINYVK(1)NCFR	0.99
P06748	NPM1	27	Nucleophosmin	85.676	ADK(1)DYHFK	1.12
Q96L73	NSD1	2435	Histone-lysine N-methyltransferase,	71.555	VLSAVVQTLVAK(1)EK	Tip60 OE only
Q9H0G5	NSRP1	499	Nuclear speckle splicing regulatory	128.31	NQEKPSNSESSLGAK(1)HR	unquantifiable
Q08J23	NSUN2	586	tRNA (cytosine(34)-C(5))-methyltran	99.653	VINTGIK(1)VWCR	1.17
Q5TFE4	NT5DC1	171	5'-nucleotidase domain-containing	117.23	DIVAAIQHNYK(1)MSAFK	Tip60 OE only
Q9BSD7	NTPCR	150	Cancer-related nucleoside-triphosp	48.981	GK(1)PLALVEEIR	0.18
Q9H1E3	NUCKS1	184	Nuclear ubiquitous casein and cycli	107.15	ATVTPSPVK(1)GK	unquantifiable
O43809	NUDT21	23	Cleavage and polyadenylation speci	221.09	GVTQFGNK(1)YIQQTK	1.07
Q9UKK9	NUDT5	42	ADP-sugar pyrophosphatase	50.284	TTYMDPTGK(1)TR	Tip60 OE only
P49790	NUP153	384	Nuclear pore complex protein Nup1	66.267	SVYFK(1)PSLTPSGEFR	0.03
P49790	NUP153	15	Nuclear pore complex protein Nup1	140.3	ASGAGGVGGGGGGK(1)IR	Tip60 OE only
P49790	NUP153	294	Nuclear pore complex protein Nup1	83.769	AK(1)QLSAQSYGVTSSTAR	Tip60 OE only
O75694	NUP155	740	Nuclear pore complex protein Nup1	48.112	NSQFAGGPLGNPNTTAK(1)V	Tip60 OE only
Q9UKX7	NUP50	450	Nuclear pore complex protein Nup5	44.318	VK(1)TSEDADELHK	unquantifiable
Q9BW27	NUP85	92	Nuclear pore complex protein Nup8	127.4	IDEELTGK(1)SR	Tip60 OE only
P52948	NUP98	1040	Nuclear pore complex protein Nup9	56.359	LPISASHSSK(1)TR	Tip60 OE only
O15381	NVL	156	Nuclear valosin-containing protein-	79.974	ISSK(1)TGSIPLK	0.04
P04181	OAT	66	Ornithine aminotransferase, mitoch	51.927	GK(1)GIYLWDVEGR	0.93
P04181	OAT	102	Ornithine aminotransferase, mitoch	49.35	IVNALK(1)SQVDK	1.01
O43929	ORC4	7	Origin recognition complex subunit	41.644	SK(1)SNSLIHTECLSQVQR	Tip60 OE only
P07237	P4HB	328	Protein disulfide-isomerase	40.589	YK(1)PESEELTAER	0.88
P22234	PAICS	30	Multifunctional protein ADE2;Phos	46.159	EVYELLDSPGK(1)VLLQSK	0.64
P22234	PAICS	304	Multifunctional protein ADE2;Phos	48.004	VTSAHK(1)GPDETLR	1.12
P22234	PAICS	247	Multifunctional protein ADE2;Phos	98.033	K(1)NFEWVAER	1.12
Q8IXS6	PALM2	195	Paralemmin-2	48.004	SGGTVVENGVHK(1)LSTK	Tip60 OE only
Q99497	PARK7	62	Protein deglycase DJ-1	74.76	DVVICPDASLEDAK(1)K	0.96
P09874	PARP1	97	Poly [ADP-ribose] polymerase 1	163.27	TAEAGGVTGK(1)GQDGIGSK	0.23
P09874	PARP1	683	Poly [ADP-ribose] polymerase 1	77.677	MIFDVESMK(1)K	0.5
P09874	PARP1	748	Poly [ADP-ribose] polymerase 1	93.959	K(1)PPLLNNADSVQAK	0.73
P09874	PARP1	633	Poly [ADP-ribose] polymerase 1	49.358	NFTK(1)YPK	0.8

P09874	PARP1	108	Poly [ADP-ribose] polymerase 1	53.557	AEK(1)TLGDFAAEYAK	0.87
P09874	PARP1	621	Poly [ADP-ribose] polymerase 1	59.542	LYEEK(1)TGNAWHSK	1.09
P09874	PARP1	629	Poly [ADP-ribose] polymerase 1	41.684	TGNAWHSK(1)NFTK	unquantifiable
Q6ZW49	PAXIP1	278	PAX-interacting protein 1	45.915	NLNWTPAEVPQLAAAK(1)R	Tip60 OE only
Q15365	PCBP1	115	Poly(rC)-binding protein 1	76.533	LVVPATQCGSLIGK(1)GGCK	0.16
Q15366	PCBP2	115	Poly(rC)-binding protein 2	64.199	LVVPASQCGSLIGK(1)GGCK	0.5
Q5JVF3	PCID2	133	PCI domain-containing protein 2	77.379	SK(1)VGDMLEK	unquantifiable
P12004	PCNA	80	Proliferating cell nuclear antigen	86.313	ILK(1)CAGNEDIITLR	Tip60 OE only
Q8WW12	PCNP	152	PEST proteolytic signal-containing r	66.299	GK(1)HGFSDNQK	Tip60 OE only
Q14690	PDCD11	1363	Protein RRP5 homolog	214.64	YSHVSQHSPSK(1)K	0.13
P08559	PDHA1	321	Pyruvate dehydrogenase E1 compor	102.06	SDPIMLLK(1)DR	0.94
P08559	PDHA1;PDHA2	77	Pyruvate dehydrogenase E1 compor	66.27	MELK(1)ADQLYK	0.89
P08559	PDHA1;PDHA2	83	Pyruvate dehydrogenase E1 compor	88.942	ADQLYK(1)QK	1.17
P11177	PDHB	68	Pyruvate dehydrogenase E1 compor	115.12	VFLLGEEVAQYDGAYK(1)VSR	1.17
O00330	PDHX	194	Pyruvate dehydrogenase protein X c	43.382	NILEK(1)HSLDASQGTATGPR	1.32
Q29RF7	PDS5A	1134	Sister chromatid cohesion protein P	50.607	VLLLTGK(1)PK	0.24
Q8IZL8	PELP1	490	Proline-, glutamic acid- and leucine-	64.55	GSPDGSLQTGK(1)PSAPK	Tip60 OE only
O00541	PES1	152	Pescadillo homolog	126.1	TGK(1)CHVQTIQLCR	0.59
O00541	PES1	98	Pescadillo homolog	48.284	AYGK(1)SEWNTVER	Tip60 OE only
Q9UHV9	PFDN2	18	Prefoldin subunit 2	101.36	SSGSGAGK(1)GAVSAEQVIA(	Tip60 OE only
P17858	PFKL	677	ATP-dependent 6-phosphofructokir	50.607	NYGTK(1)LGVK	1.04
P07737	PFN1	108	Profilin-1	84.213	TDK(1)TLVLLMGK	1.02
P07737	PFN1	105	Profilin-1	58.32	STGGAPTFNVTVTK(1)TDK	1.02
P18669	PGAM1;PGAM2	251	Phosphoglycerate mutase 1;Phosph	77.677	AMEAVAAQGK(1)AK	0.64
P18669	PGAM1;PGAM4	113	Phosphoglycerate mutase 1;Probab	57.598	HGEAQVK(1)IWR	Tip60 OE only
P18669	PGAM1;PGAM4;PGAN	100	Phosphoglycerate mutase 1;Probab	180.11	HYGGLTGLNK(1)AETAAK	0.81
P18669	PGAM1;PGAM4;PGAN	106	Phosphoglycerate mutase 1;Probab	70.977	AETAAK(1)HGEAQVK	0.87
P52209	PGD	38	6-phosphogluconate dehydrogenas	54.7	TVSK(1)VDDFLANEAK	0.81
P00558	PGK1	75	Phosphoglycerate kinase 1	143.35	SVVLMSHLGRPDGVPMPDK	0.47
P00558	PGK1	30	Phosphoglycerate kinase 1	68.171	VDFNVPMK(1)NNQITNNQR	0.83
P00558	PGK1	353	Phosphoglycerate kinase 1	45.137	GTK(1)ALMDEVVK	0.84
P00558	PGK1	131	Phosphoglycerate kinase 1	97.431	FHVEEEGK(1)GK	1.06
P00558	PGK1	220	Phosphoglycerate kinase 1	75.695	VADK(1)IQLINNMLDK	Tip60 OE only
O95394	PGM3	350	Phosphoacetylglucosamine mutase	51.927	VPVYCTK(1)TGVK	0.93

O00264	PGRMC1	105	Membrane-associated progesterone	40.589	K(1)FYGPEGPYGVFAGR	Tip60 OE only
Q8IXK0	PHC2	702	Polyhomeotic-like protein 2	74.944	ASK(1)ASLPPLTK	Tip60 OE only
Q92576	PHF3	520	PHD finger protein 3	63.216	YEVIHSK(1)TK	unquantifiable
Q7RTV0	PHF5A	3	PHD finger-like domain-containing p	133.23	AK(1)HHPDLIFCR	0.09
Q7RTV0	PHF5A	95	PHD finger-like domain-containing p	85.288	IVNLGSSK(1)TDLFYER	0.31
Q9UPP1	PHF8	219	Histone lysine demethylase PHF8	70.268	YYYSGK(1)R	unquantifiable
O43175	PHGDH	58	D-3-phosphoglycerate dehydrogena	69.602	SATK(1)VTADVINAAEK	1.43
Q8N2W9	PIAS4	125	E3 SUMO-protein ligase PIAS4	57.785	LPAK(0.994)TLK(0.006)PEV	unquantifiable
Q13526	PIN1	82	Peptidyl-prolyl cis-trans isomerase N	61.435	TK(1)EEALELINGYIQK	Tip60 OE only
P48739	PITPNB	44	Phosphatidylinositol transfer protei	49.627	NETGGGEGIEVLK(1)NEPYEK	1.13
Q5JRX3	PITRM1	770	Presequence protease, mitochondri	55.567	K(1)HLLNGDNMR	0.8
P14618	PKM	115	Pyruvate kinase PKM	57.019	PVAVALDTK(1)GPEIR	0.54
P14618	PKM	188	Pyruvate kinase PKM	55.841	QK(1)GADFLVTEVENGGSLG	0.87
P14618	PKM	322	Pyruvate kinase PKM	72.937	AGK(1)PVICATQMLESMIK	0.98
P14618	PKM	66	Pyruvate kinase PKM	78.934	EMIK(1)SGMNVAR	1.28
P14618	PKM	498	Pyruvate kinase PKM	46.891	VNFAMNVGK(1)AR	1.47
P14618	PKM	270	Pyruvate kinase PKM	47.559	IISK(1)IENHEGVR	Tip60 OE only
Q16513	PKN2	247	Serine/threonine-protein kinase N2	80.688	LLGSGK(1)VTDR	0.04
Q16513	PKN2	77	Serine/threonine-protein kinase N2	42.395	K(1)SLAYVDNILK	0.75
Q13835	PKP1	595	Plakophilin-1	44.395	EK(1)GLPQIAR	unquantifiable
O60664	PLIN3	65	Perilipin-3	50.353	TVCDAAEK(1)GVR	1.17
P53350	PLK1	474	Serine/threonine-protein kinase PLK	87.18	DGTESYLTVSSHPNSLMK(1)K	unquantifiable
P13797	PLS3	587	Plastin-3	42.309	SGNLTEDDK(0.043)HNNAK	0.85
P13797	PLS3	471	Plastin-3	42.813	KLENCNYAVELGK(1)HPAK	1.16
Q9H307	PNN	553	Pinin	99.531	LTEVPVEPVLTVHPESK(1)SK	Tip60 OE only
Q8TCS8	PNPT1	306	Polyribonucleotide nucleotidyltran	41.283	LYAVFTDYEHDK(1)VSR	1.03
Q9BY77	POLDIP3	178	Polymerase delta-interacting protei	95.255	INVVNNHQAK(1)QNLYDLDE	Tip60 OE only
O14802	POLR3A	398	DNA-directed RNA polymerase III sul	52.716	VNK(1)ANINFLR	1.06
O14802	POLR3A	1220	DNA-directed RNA polymerase III sul	71.806	AVIHIDEQSGK(1)EK	Tip60 OE only
Q9H1D9	POLR3F	84	DNA-directed RNA polymerase III sul	66.435	IKDSQNAGK(1)MK	Tip60 OE only
O00411	POLRMT	402	DNA-directed RNA polymerase, mito	44.005	TLQCLFEK(1)QLHMELASR	Tip60 OE only
A8CG34	POM121C	99	Nuclear envelope pore membrane p	89.466	TLFASPPAK(1)STANGNLLEP	Tip60 OE only
Q06203	PPAT	81	Amidophosphoribosyltransferase	42.743	K(1)LYVSNLGIGHTR	0.8
Q8NEY8	PPHLN1	160	Periphilin-1	69.01	SK(1)SYSFHQSQHR	Tip60 OE only

P62937	PPIA	76	Peptidyl-prolyl cis-trans isomerase A	306.08	HNGTGGK(1)SIYGEK	0.67
P62937	PPIA	31	Peptidyl-prolyl cis-trans isomerase A	78.334	VPK(1)TAENFR	0.89
P62937	PPIA	155	Peptidyl-prolyl cis-trans isomerase A	142.92	K(1)ITIADCGQLE	0.9
P62937	PPIA	131	Peptidyl-prolyl cis-trans isomerase A	70.912	HVVFGK(1)VK	0.95
P62937	PPIA	82	Peptidyl-prolyl cis-trans isomerase A	66.498	SIYGEK(1)FEDENFILK	1
P62937	PPIA	44	Peptidyl-prolyl cis-trans isomerase A	98.249	ALSTGEK(1)GFGYK	1.09
P23284	PPIB	165	Peptidyl-prolyl cis-trans isomerase B	49.35	TAWLDGK(1)HVVFGK	1.59
P30405	PPIF	73	Peptidyl-prolyl cis-trans isomerase F	47.302	ADVVPK(1)TAENFR	0.84
Q13427	PPIG	52	Peptidyl-prolyl cis-trans isomerase G	62.14	GTGK(1)STQKPLHYK	Tip60 OE only
Q8WUA2	PPIL4	321	Peptidyl-prolyl cis-trans isomerase-l	43.308	IHVDFSQSVAK(1)VK	0.3
O15355	PPM1G	172	Protein phosphatase 1G	50.464	GPPHSK(1)SGGGTGEEPGSC	0.13
O15355	PPM1G	339	Protein phosphatase 1G	57.019	GK(1)QLIVANAGDSR	0.6
O15355	PPM1G	166	Protein phosphatase 1G	40.589	YGQNCHK(1)GPPHSK	0.72
O15355	PPM1G	383	Protein phosphatase 1G	64.82	NAGGK(1)VTMDGR	0.86
O15355	PPM1G	247	Protein phosphatase 1G	69.356	GTEAGQVGEPGIPTGEAGPS(	1.53
P62136	PPP1CA	305	Serine/threonine-protein phosphata	56.783	GK(1)YGQFSGLNPGGR	Tip60 OE only
P62136	PPP1CA;PPP1CC	238	Serine/threonine-protein phosphata	58.978	FLHK(1)HDLDLICR	1.47
P62140	PPP1CA;PPP1CC;PPP1	259	Serine/threonine-protein phosphata	42.568	AHQVVEDGYEFFAK(1)R	0.34
P62140	PPP1CA;PPP1CC;PPP1	140	Serine/threonine-protein phosphata	40.352	IYGFYDECK(1)R	Tip60 OE only
P62140	PPP1CB	25	Serine/threonine-protein phosphata	50.284	PGK(1)IVQMTEAEVR	0.77
P62140	PPP1CB	303	Serine/threonine-protein phosphata	155.15	AK(1)YQYGGLNSGR	Tip60 OE only
P30153	PPP2R1A	280	Serine/threonine-protein phosphata	100.19	AVGPEITK(1)TDLVPAFQNLM	0.69
P30153	PPP2R1A	307	Serine/threonine-protein phosphata	57.836	VK(1)EFCENLSADCR	1.42
P63151	PPP2R2A	105	Serine/threonine-protein phosphata	47.915	WLPQK(1)NAAQFLLSTNDK	0.78
O60828	PQBP1	228	Polyglutamine-binding protein 1	68.809	NEAK(1)TGADTTAAGPLFQQ	Tip60 OE only
Q92733	PRCC	250	Proline-rich protein PRCC	107.06	AAAK(1)SAALQVTK	0.45
Q92733	PRCC	225	Proline-rich protein PRCC	77.222	TK(1)TSSLAPVVGTTTTTPSPS	Tip60 OE only
Q06830	PRDX1	120	Peroxiredoxin-1	83.769	TIAQDYGVLK(1)ADEGISFR	0.75
Q06830	PRDX1	35	Peroxiredoxin-1	49.715	DISLSDYK(1)GK	1.19
Q06830	PRDX1;PRDX4	136	Peroxiredoxin-1;Peroxiredoxin-4	43.512	GLFIIDDK(1)GILR	0.99
P32119	PRDX2	119	Peroxiredoxin-2	86.028	LSEDYGVLK(1)TDEGIAYR	1.77
P32119	PRDX2	135	Peroxiredoxin-2	53.683	GLFIIDGK(1)GVLR	Tip60 OE only
P30048	PRDX3	91	Thioredoxin-dependent peroxide re	75.378	DLSLDDFK(1)GK	0.84
P10644	PRKAR1A	367	cAMP-dependent protein kinase typ	42.743	VLGPCSDILK(1)R	1

P78527	PRKDC	838	DNA-dependent protein kinase catal	44.616	TK(1)NLSSNEAISLEEIR	0.84
Q9UMS4	PRPF19	192	Pre-mRNA-processing factor 19	58.83	GK(1)TVPEELVKPEELSK	0.35
Q5VTL8	PRPF38B	42	Pre-mRNA-splicing factor 38B	60.628	PAVSGK(1)QGNVLPLWGNE	Tip60 OE only
Q13523	PRPF4B	170	Serine/threonine-protein kinase PRI	70.816	GK(1)LELVDNK	Tip60 OE only
Q13523	PRPF4B	656	Serine/threonine-protein kinase PRI	60.019	AAGIGK(1)DFK	Tip60 OE only
P48634	PRRC2A	49	Protein PRRC2A	60.434	HGLQSLGK(1)VAIAR	1.07
Q9Y520	PRRC2C	47	Protein PRRC2C	49.5	HGLQSLGK(1)VGISR	1.05
Q9Y617	PSAT1	333	Phosphoserine aminotransferase	49.5	ALELNMLSLK(1)GHR	0.93
O75475	PSIP1	360	PC4 and SFRS1-interacting protein	81.338	IHAEIK(1)NSLK	1.12
O75475	PSIP1	476	PC4 and SFRS1-interacting protein	42.121	EQTGSK(1)TLNGGSDAQDGI	Tip60 OE only
O75475	PSIP1;HDGF;HDGFRP2	16	PC4 and SFRS1-interacting protein;I	122.52	MK(1)GYPHWPAR	0.02
P25786	PSMA1	115	Proteasome subunit alpha type-1	121.08	LVSLIGSK(1)TQIPTQR	0.75
P25787	PSMA2	53	Proteasome subunit alpha type-2	45.137	QK(1)SILYDER	1.04
P25787	PSMA2	171	Proteasome subunit alpha type-2	47.844	NYVNGK(1)TFLEK	1.11
P25788	PSMA3	57	Proteasome subunit alpha type-3	45.359	LVLSK(0.999)LYEEGSNK(0.(	0.87
P25789	PSMA4	127	Proteasome subunit alpha type-4	69.423	QAYTQFGGK(1)R	Tip60 OE only
P60900	PSMA6	104	Proteasome subunit alpha type-6	80.979	YK(1)YGYEIPVDMLCK	0.85
P49720	PSMB3	77	Proteasome subunit beta type-3	81.338	LNLYELK(1)EGR	0.9
Q99460	PSMD1	310	26S proteasome non-ATPase regulat	52.555	TSSAFVGK(1)TPEASPEPK	Tip60 OE only
O00231	PSMD11	32	26S proteasome non-ATPase regulat	72.2	EASIDILHSIVK(1)R	Tip60 OE only
P51665	PSMD7	204	26S proteasome non-ATPase regulat	44.318	GLNSK(1)LLDIR	0.86
P51665	PSMD7	279	26S proteasome non-ATPase regulat	79.693	SVVALHNLINNK(1)IANR	1.17
P48556	PSMD8	138	26S proteasome non-ATPase regulat	60.973	LTK(1)QQLILAR	1.19
Q8WXF1	PSPC1	206	Paraspeckle component 1	80.239	GFVEFAAK(1)PPAR	Tip60 OE only
Q8WXF1	PSPC1	519	Paraspeckle component 1	48.44	GSQGGNFEGPNK(1)R	unquantifiable
P26599	PTBP1	410	Polypyrimidine tract-binding protei	53.756	ITLSK(1)HQNVQLPR	0.74
P26599	PTBP1	137	Polypyrimidine tract-binding protei	43.512	ELK(1)TDSSPNQAR	1.02
Q15185	PTGES3	33	Prostaglandin E synthase 3	129.85	DVNVNFEK(1)SK	0.96
Q15185	PTGES3	65	Prostaglandin E synthase 3	44.577	HLNEIDLFHCIDPNDSK(1)HI	0.98
Q15185	PTGES3	7	Prostaglandin E synthase 3	52.79	MQPASAK(1)WYDR	1.07
Q15185	PTGES3	35	Prostaglandin E synthase 3	101.39	SK(1)LTFSCLGGSDNFK	1.13
P06454	PTMA	15	Prothymosin alpha;Prothymosin alp	106.17	SDAAVDTSSEITTK(1)DLK	1.16
P20962	PTMS	15	Parathymosin	105.17	SVEAAAELSAK(1)DLK	unquantifiable
O95997	PTTG1	101	Securin	52.344	AK(1)SSVPASDDAYPEIEK	Tip60 OE only

Q96PZ0	PUS7	69	Pseudouridylate synthase 7 homolo	49.486	GQDGLQNDFLSISEDVPRPP	0.63
P06737	PYGL	29	Glycogen phosphorylase, liver form	42.743	GIVGVENVAELK(1)K	0.54
Q2KHR3	QSER1	1419	Glutamine and serine-rich protein 1	62.891	EFAATNSYLGYFGDAK(1)SK	Tip60 OE only
P51153	RAB13	3	Ras-related protein Rab-13	64.711	AK(1)AYDHLFK	unquantifiable
P54727	RAD23B	76	UV excision repair protein RAD23 ho	76.228	NFVVVMVTK(1)PK	0.08
Q92878	RAD50	321	DNA repair protein RAD50	65.038	K(1)LVDCHR	1.21
Q9Y4B4	RAD54L2	665	Helicase ARIP4	40.025	GK(1)GEDSTLASSMGEATNS	Tip60 OE only
Q9Y4B4	RAD54L2	1105	Helicase ARIP4	75.109	GTK(1)GTYIR	unquantifiable
Q7Z5J4	RAI1	774	Retinoic acid-induced protein 1	47.288	GLEQGGK(1)ASDGISK	Tip60 OE only
P62826	RAN	134	GTP-binding nuclear protein Ran	75.854	AK(1)SIVFHR	0.28
P62826	RAN	99	GTP-binding nuclear protein Ran	161.79	VTYK(1)NVPNWHR	0.77
P62826	RAN	71	GTP-binding nuclear protein Ran	93.959	FNVWDTAGQEK(1)FGGLR	0.96
P62826	RAN	123	GTP-binding nuclear protein Ran	59.003	VCENIPIVLCGNK(1)VDIK	0.99
P62826	RAN	142	GTP-binding nuclear protein Ran	206.11	K(0.005)K(0.995)NLQYYDIS	1.05
P62826	RAN	60	GTP-binding nuclear protein Ran	44.616	GPIK(1)FNVWDTAGQEK	1.06
P62826	RAN	37	GTP-binding nuclear protein Ran	112.84	HLTGEFEK(1)K	1.08
Q6VN20	RANBP10	293	Ran-binding protein 10	53.528	MTETPIQEEQASIK(1)NR	unquantifiable
Q96S59	RANBP9	405	Ran-binding protein 9	43.991	STDQTVLEELASIK(1)NR	unquantifiable
P46060	RANGAP1	524	Ran GTPase-activating protein 1	90.685	LLVHMGLLK(1)SEDK	0.73
P46060	RANGAP1	279	Ran GTPase-activating protein 1	52.247	SK(1)GAVAIADAIR	0.96
P54136	RARS	393	Arginine--tRNA ligase, cytoplasmic	97.953	SDGGYTYDTSDLAAIK(1)QR	Tip60 OE only
Q09028	RBBP4	4	Histone-binding protein RBBP4	56.225	ADK(1)EAAFDDAVEER	1.32
Q15291	RBBP5	500	Retinoblastoma-binding protein 5	48.794	EKDSPFK(1)PK	0.31
Q7Z6E9	RBBP6	232	E3 ubiquitin-protein ligase RBBP6	58.676	YAIPTIDAEAYAIGK(1)K	Tip60 OE only
Q16576	RBBP7	119	Histone-binding protein RBBP7	109.81	IECEIK(1)INHEGEVNR	0.92
Q16576	RBBP7	4	Histone-binding protein RBBP7	56.225	ASK(1)EMFEDTVEER	Tip60 OE only
Q96PK6	RBM14	149	RNA-binding protein 14	67.334	INVELSTK(1)GQK	0.48
Q96T37	RBM15	450	Putative RNA-binding protein 15	60.518	IGYGK(1)ATPTTR	unquantifiable
Q8NDT2	RBM15B	98	Putative RNA-binding protein 15B	60.157	GGK(1)ASGDPGASGMSPR	Tip60 OE only
Q8NDT2	RBM15B	46	Putative RNA-binding protein 15B	66.595	ASGGAK(1)HPVPAR	Tip60 OE only
Q96I25	RBM17	276	Splicing factor 45	120.09	GGK(1)IIVGDATEK	Tip60 OE only
Q96I25	RBM17	206	Splicing factor 45	69.763	SQSSK(1)AAIPPPVYEEQDRP	Tip60 OE only
Q96I25	RBM17	256	Splicing factor 45	48.112	EGQGLGK(1)HEQGLSTALSV	Tip60 OE only
P49756	RBM25	135	RNA-binding protein 25	109.22	LQAFGFCEYK(1)EPESTLR	1.48

P49756	RBM25	125	RNA-binding protein 25	52.359	VQGASGK(1)LQAFGFCEYKEI	Tip60 OE only
Q5T8P6	RBM26	709	RNA-binding protein 26	81.619	AAQK(1)TLLVSTSAVDNNEA(	0.95
Q9NW13	RBM28	746	RNA-binding protein 28	59.426	LLGPSK(1)GAPLAK	Tip60 OE only
Q14498	RBM39	291	RNA-binding protein 39	77.912	SK(1)GYGFITFSDSECAK	0.43
Q14498	RBM39	232	RNA-binding protein 39	44.616	VLGVPIIVQASQAEK(1)NR	0.73
P38159	RBMX	86	RNA-binding motif protein, X chrom	118.48	VEQATK(1)PSFESGR	0.04
P38159	RBMX	30	RNA-binding motif protein, X chrom	56.563	ALEAVFGK(1)YGR	Tip60 OE only
Q9Y388	RBMX2	77	RNA-binding motif protein, X-linked	48.004	SK(1)GFCFLCYEDQR	Tip60 OE only
P62877	RBX1	105	E3 ubiquitin-protein ligase RBX1;E3	40.103	EWEFQK(1)YGH	1.27
Q9P258	RCC2	77	Protein RCC2	59.469	PATAGK(1)AGGAAVVITEPEH	0.52
Q9H6H4	REEP4	123	Receptor expression-enhancing prot	116.3	SYETVLSFGK(1)R	Tip60 OE only
Q9BWE0	REPIN1	33	Replication initiator 1	49.31	LLSGPSQESPQTLGK(1)ESR	unquantifiable
P35251	RFC1	94	Replication factor C subunit 1	57.96	LPVSSK(1)PGK	0.37
P35251	RFC1	638	Replication factor C subunit 1	56.043	FSGK(1)DDGSSFK	Tip60 OE only
P35249	RFC4	13	Replication factor C subunit 4	63.283	GTSISTK(1)PPLTK	0.76
Q8IXI1	RHOT2	315	Mitochondrial Rho GTPase 2	69.954	VFEK(1)HDQDR	Tip60 OE only
Q05823	RNASEL	684	2-5A-dependent ribonuclease	52.79	LK(1)IGDPSLYFQK	unquantifiable
Q15287	RNPS1	218	RNA-binding protein with serine-ric	132.79	GYAYVEFENPDEAEK(1)ALK	0.6
P27694	RPA1	163	Replication protein A 70 kDa DNA-b	56.432	AYGASK(1)TFGK	0.19
P27635	RPL10	188	60S ribosomal protein L10	98.353	FNADEFEDMVAEK(1)R	1.13
P62906	RPL10A	130	60S ribosomal protein L10a	56.729	ILGPGLNK(1)AGK	1.05
P62913	RPL11	52	60S ribosomal protein L11	53.528	VLEQLTGQTPVFSK(1)AR	1.12
P62913	RPL11	85	60S ribosomal protein L11	45.368	AEEILEK(1)GLK	unquantifiable
P26373	RPL13	123	60S ribosomal protein L13	51.854	SK(1)LILFPR	0.38
P40429	RPL13A	197	60S ribosomal protein L13a	66.435	YTEVLK(1)THGLLV	0.77
P50914	RPL14	85	60S ribosomal protein L14	63.283	ADINTK(1)WAATR	1.36
P61313	RPL15	83	60S ribosomal protein L15	45.864	GATYGK(1)PVHHGVNQLK	0.4
P61313	RPL15	56	60S ribosomal protein L15	86.953	AK(1)QGYVIYR	Tip60 OE only
P18621	RPL17	159	60S ribosomal protein L17	50.284	EQIVPK(1)PEEEVAQK	0.7
P18621	RPL17	13	60S ribosomal protein L17	73.781	YSLDPENPTK(1)SCK	0.87
P18621	RPL17	55	60S ribosomal protein L17	80.239	DVTLQK(1)QCVPFR	1.06
Q07020	RPL18	78	60S ribosomal protein L18	75.479	ENK(1)TAVVVGTITDDVR	1.15
Q07020	RPL18	119	60S ribosomal protein L18	62.14	AGGK(1)ILTFDQLALDSPK	Tip60 OE only
Q02543	RPL18A	136	60S ribosomal protein L18a	118.18	VEEIAASK(1)CR	0.76

Q02543	RPL18A	41	60S ribosomal protein L18a	50.292	IFAPNHVVAK(1)SR	1.02
Q02543	RPL18A	170	60S ribosomal protein L18a	54.608	FTTK(1)RPNTFF	1.6
Q02543	RPL18A	76	60S ribosomal protein L18a	42.109	VK(1)NFGIWLR	1.72
P84098	RPL19	144	60S ribosomal protein L19	48.796	ILMEHIHK(1)LK	0.84
P46778	RPL21	21	60S ribosomal protein L21	44.318	K(1)HGVVPLATYMR	0.78
P46778	RPL21	55	60S ribosomal protein L21	41.029	GMPHK(1)CYHGK	1.16
P62829	RPL23	43	60S ribosomal protein L23	76.465	NLYIISVK(1)GIK	1.68
P62750	RPL23A	70	60S ribosomal protein L23a	121.6	NK(1)LDHYAIIK	0.38
P83731	RPL24	27	60S ribosomal protein L24	123.35	TDGK(1)VFQFLNAK	0.68
P83731	RPL24	12	60S ribosomal protein L24	86.313	VELCSFSGYK(1)IYPGHGR	0.99
P61254	RPL26;RPL26L1	77	60S ribosomal protein L26;60S ribo	79.906	K(1)YVIYIER	Tip60 OE only
P61254	RPL26;RPL26L1	69	60S ribosomal protein L26;60S ribo	66.299	GQQIGK(1)VVQVYR	Tip60 OE only
P61353	RPL27	27	60S ribosomal protein L27	55.67	AVIVK(1)NIDDGTSDRPYSHA	1.02
P61353	RPL27	3	60S ribosomal protein L27	40.268	GK(0.999)FMK(0.001)PGK	Tip60 OE only
P46776	RPL27A	47	60S ribosomal protein L27a	40.725	INFDK(1)YHPGYFGK	1.13
P39023	RPL3	66	60S ribosomal protein L3	87.298	EVDRPGSK(1)VNK	0.2
P39023	RPL3	39	60S ribosomal protein L3	54.7	DDPSK(1)PVHLTAFLGYK	0.86
P39023	RPL3	103	60S ribosomal protein L3	61.435	TFK(1)TVFAEHISDECK	1.5
P49207	RPL34	62	60S ribosomal protein L34	52.786	AVRPK(1)VLMR	0.26
P49207	RPL34	43	60S ribosomal protein L34	44.863	APK(1)SACGVCPGR	0.62
P49207	RPL34	36	60S ribosomal protein L34	65.252	IVYLYTK(1)K	0.89
P49207	RPL34	19	60S ribosomal protein L34	82.261	LSYNTASNK(1)TR	Tip60 OE only
P18077	RPL35A	8	60S ribosomal protein L35a	58.676	LWSK(1)AIFAGYK	1.66
P61513	RPL37A;RPL37AP8	44	60S ribosomal protein L37a;Putativ	64.297	YTCSFCGK(1)TK	1.11
P36578	RPL4	364	60S ribosomal protein L4	70.488	AAAAAAALQAK(1)SDEK	0.45
P36578	RPL4	106	60S ribosomal protein L4	78.334	MFAPTK(1)TWR	0.63
P36578	RPL4	353	60S ribosomal protein L4	47.559	VDK(1)AAAAAAALQAK	1.1
P36578	RPL4	239	60S ribosomal protein L4	63.283	LNILK(1)LAPGGHVGR	1.4
P36578	RPL4	20	60S ribosomal protein L4	53.528	GESSGK(1)NVTLPAVFK	Tip60 OE only
P36578	RPL4	14	60S ribosomal protein L4	40.589	PLISVYSEK(1)GESSGK	Tip60 OE only
P62424	RPL7A	101	60S ribosomal protein L7a	45.997	LAHK(1)YRPETK	0.98
P62424	RPL7A	212	60S ribosomal protein L7a	52.265	TCTTVAFTQVNSEDK(1)GALA	1.17
P62917	RPL8	60	60S ribosomal protein L8	51.346	GAPLAK(1)VVFR	0.78
P62917	RPL8	46	60S ribosomal protein L8	69.979	GIVK(1)DIIHDPGR	0.92

P32969	RPL9	174	60S ribosomal protein L9	48.091	K(1)FLDGIYVSEK	0.52
P32969	RPL9	121	60S ribosomal protein L9	49.418	NFLGEK(1)YIR	1.1
P05388	RPLP0;RPLP0P6	106	60S acidic ribosomal protein P0;60S	55.567	DMLLANK(1)VPAAAR	0.43
P04843	RPN1	564	Dolichyl-diphosphooligosaccharide	53.683	ELVLK(1)SAVEAER	1.06
P04843	RPN1	538	Dolichyl-diphosphooligosaccharide	137.98	LK(1)TEGSDLCDR	1.12
P04844	RPN2	311	Dolichyl-diphosphooligosaccharide	46.462	LEHAK(1)SVASR	unquantifiable
P78345	RPP38	241	Ribonuclease P protein subunit p38	79.82	ELLDTSFEDLSK(1)PK	Tip60 OE only
P46783	RPS10	107	40S ribosomal protein S10	48.741	PK(1)GLEGERPAR	0.27
P46783	RPS10	59	40S ribosomal protein S10	57.598	GYVK(1)EQFAWR	Tip60 OE only
P62280	RPS11	30	40S ribosomal protein S11	52.278	VLLGETGK(1)EK	0.56
P62280	RPS11	45	40S ribosomal protein S11	93.649	NIGLGFK(1)TPK	0.69
P62277	RPS13	9	40S ribosomal protein S13	99.481	MHAPGK(1)GLSQSALPYR	0.28
P62277	RPS13	43	40S ribosomal protein S13	52.79	K(1)GLTPSQIGVILR	1.03
P62263	RPS14	96	40S ribosomal protein S14	54.608	ELGITALHIK(1)LR	1.5
P62249	RPS16	4	40S ribosomal protein S16	200.93	PSK(1)GPLQSVQVFGR	0.07
P62249	RPS16	60	40S ribosomal protein S16	51.927	LLEPVLLLGK(1)ER	Tip60 OE only
P15880	RPS2	275	40S ribosomal protein S2	90.561	SPYQEFTDHLVK(1)THTR	0.86
P60866	RPS20	75	40S ribosomal protein S20	42.095	TPCGEGSK(1)TWDR	0.75
P63220	RPS21	27	40S ribosomal protein S21	172.24	IIGAK(1)DHASIQMNVAEVDI	0.38
P63220	RPS21	41	40S ribosomal protein S21	67.449	DHASIQMNVAEVDK(1)VTGI	0.78
P63220	RPS21	74	40S ribosomal protein S21	41.704	LAK(1)ADGIVSK	0.97
P62266	RPS23	135	40S ribosomal protein S23	42.336	VANVSLLALYK(1)GK	1.19
P62847	RPS24	37	40S ribosomal protein S24	49.715	ATVPK(1)TEIR	0.96
P62851	RPS25	94	40S ribosomal protein S25	60.434	AALQELLSK(1)GLIK	0.98
P62851	RPS25	43	40S ribosomal protein S25	42.743	DK(1)LNNLVLFDK	unquantifiable
Q5JNZ5	RPS26P11;RPS26	82	Putative 40S ribosomal protein S26	64.394	LHYCVSCAIHSK(1)VVR	0.97
P62979	RPS27A	152	Ubiquitin-40S ribosomal protein S2	50.284	CCLTYCFNK(1)PEDK	1.13
P62979	RPS27A;UBA52;UBB;U	48	Ubiquitin-40S ribosomal protein S2	67.726	LIFAGK(1)QLEDGR	0.63
P62979	RPS27A;UBA52;UBB;U	6	Ubiquitin-40S ribosomal protein S2	145.25	MQIFVK(1)TLTGK	1.16
P62979	RPS27A;UBA52;UBB;U	27	Ubiquitin-40S ribosomal protein S2	76.326	TITLEVEPSDTIENVK(1)AK	Tip60 OE only
P62979	RPS27A;UBA52;UBB;U	11	Ubiquitin-40S ribosomal protein S2	101.36	TLTGK(1)TITLEVEPSDTIENVI	Tip60 OE only
P23396	RPS3	75	40S ribosomal protein S3	41.029	ELTAVVQK(1)R	1
P23396	RPS3	108	40S ribosomal protein S3	41.029	YK(1)LLGGLAVR	1.13
P61247	RPS3A	46	40S ribosomal protein S3a	56.258	NIGK(1)TLVTR	0.91

P61247	RPS3A	144	40S ribosomal protein S3a	78.334	LFCVGFTK(1)K	1.08
P61247	RPS3A	56	40S ribosomal protein S3a	49.5	TQGTK(1)IASDGLK	unquantifiable
P62701	RPS4X	134	40S ribosomal protein S4, X isoform	123.02	IFVGTK(1)GIPHLVTHDAR	0.18
P62753	RPS6	79	40S ribosomal protein S6	125.54	LLLSK(1)GHSCYRPR	0.89
P62753	RPS6	203	40S ribosomal protein S6	64.711	NK(1)EEAAEYAK	1.06
P62753	RPS6	149	40S ribosomal protein S6	46.158	LFNLSK(1)EDDVR	unquantifiable
P62081	RPS7	74	40S ribosomal protein S7	41.227	SFQK(1)IQVR	1.24
P62241	RPS8	98	40S ribosomal protein S8	93.959	TLVK(1)NCIVLIDSTPYR	0.91
P46781	RPS9	155	40S ribosomal protein S9	49.35	LDSQK(1)HIDFSLR	0.66
P23921	RRM1	754	Ribonucleoside-diphosphate reduct	69.594	TRPAANPIQFTLNK(1)EK	Tip60 OE only
Q9Y3B9	RRP15	108	RRP15-like protein	72.006	TPESK(1)PTILVK	0.12
Q9Y3B9	RRP15	179	RRP15-like protein	51.276	GVVQLFNAVQK(1)HQK	1.38
Q14684	RRP1B	238	Ribosomal RNA processing protein 1	81.311	TK(1)VGDGDLSAEEIPENEVS	Tip60 OE only
Q15050	RRS1	279	Ribosome biogenesis regulatory pro	87.308	K(1)PQLDVTR	unquantifiable
Q96T23	RSF1	1061	Remodeling and spacing factor 1	135.66	GK(1)DISTILDEERK	0.74
Q96T23	RSF1	1050	Remodeling and spacing factor 1	192.26	GK(1)DISTITGHR	0.76
O76021	RSL1D1	395	Ribosomal L1 domain-containing pr	87.568	SPAK(1)SPNPSTPR	0.03
O76021	RSL1D1	391	Ribosomal L1 domain-containing pr	79.639	K(0.893)SPAK(0.107)SPNP	Tip60 OE only
O76021	RSL1D1	38	Ribosomal L1 domain-containing pr	74.841	K(1)AVDALLTHCK	Tip60 OE only
Q92541	RTF1	600	RNA polymerase-associated protein	54.539	QCK(1)PTIVSNSR	Tip60 OE only
Q92541	RTF1	413	RNA polymerase-associated protein	140.74	VAEITGVVETAK(1)VYQLGGT	Tip60 OE only
Q9NR31	SAR1A;SAR1B	46	GTP-binding protein SAR1a;GTP-bin	58.699	TTLLHMLK(1)DDR	Tip60 OE only
P82979	SARNP	17	SAP domain-containing ribonucleo	52.79	LAELK(1)QECLAR	1.29
O95104	SCAF4	41	Splicing factor, arginine/serine-rich	45.368	LYK(1)HVVQIVEK	1.49
P31040	SDHA	538	Succinate dehydrogenase [ubiquino	55.401	VGSVLQEGCGK(1)ISK	0.89
Q15019	SEPTIN2	3	Septin-2	48.337	SK(1)QQPTQFINPETPGYVG	0.91
Q8NC51	SERBP1	329	Plasminogen activator inhibitor 1 R	47.09	SK(1)SEEAHAEDSVMDHHFR	0.37
Q8NC51	SERBP1	68	Plasminogen activator inhibitor 1 R	124.51	SAAQAAAQTNSNAAGK(1)Q	0.66
Q8NC51	SERBP1	122	Plasminogen activator inhibitor 1 R	60.474	RPDQQLQGEGK(1)IIDR	0.8
Q01105	SET	167	Protein SET	119.68	EFHLNESGDPSSK(1)STEIK	1.05
Q01105	SET;SETSIP	132	Protein SET;Protein SETSIP	119.76	VEVTEFEDIK(1)SGYR	1.14
Q15459	SF3A1	223	Splicing factor 3A subunit 1	67.113	ILIPPK(1)GLFSK	0.1
Q15459	SF3A1	55	Splicing factor 3A subunit 1	61.353	NIVDK(1)TASFVAR	1.08
Q15428	SF3A2	10	Splicing factor 3A subunit 2	159.02	PGGK(1)TGSGGVASSSESNR	0.09

Q12874	SF3A3	264	Splicing factor 3A subunit 3	46.462	LK(1)SALLALGLK	1.23
O75533	SF3B1	6	Splicing factor 3B subunit 1	75.78	IAK(1)THEDIEAQIR	0.28
O75533	SF3B1	554	Splicing factor 3B subunit 1	40.002	HLLVK(1)VIDR	1.29
O75533	SF3B1	240	Splicing factor 3B subunit 1	98.902	AK(1)GSETPGATPGSK	Tip60 OE only
O75533	SF3B1	3	Splicing factor 3B subunit 1	75.78	AK(1)IAK(1)THEDIEAQIR	Tip60 OE only
O75533	SF3B1	81	Splicing factor 3B subunit 1	103.86	K(1)PGYHAPVALLNDIPQSTE	Tip60 OE only
P23246	SFPQ	232	Splicing factor, proline- and glutami	73.296	PGGGPGLSTPGGHPK(1)PPI	0.37
P23246	SFPQ	703	Splicing factor, proline- and glutami	75.378	EEYEGPNK(0.91)K(0.09)PR	0.55
P23246	SFPQ	421	Splicing factor, proline- and glutami	51.927	GIVEFASK(1)PAAR	0.78
Q12872	SFSWAP	18	Splicing factor, suppressor of white-	79.804	SGAK(1)EEAGPGGAGGGGSI	Tip60 OE only
P34896	SHMT1	386	Serine hydroxymethyltransferase, cy	42.789	VLEACSIACNK(1)NTCPGDR	Tip60 OE only
P34897	SHMT2	103	Serine hydroxymethyltransferase, m	52.937	YSEGYPGK(1)R	0.64
P34897	SHMT2	200	Serine hydroxymethyltransferase, m	73.887	LNPK(1)TGLIDYNQLALTAR	0.67
P34897	SHMT2	356	Serine hydroxymethyltransferase, m	47.603	EYSLQVLK(1)NAR	0.82
P34897	SHMT2	469	Serine hydroxymethyltransferase, m	62.463	LQDFK(1)SFLLK	0.86
P78324	SIRPA	483	Tyrosine-protein phosphatase non-r	45.081	TPK(1)QPAPK	unquantifiable
P42285	SKIV2L2	78	Superkiller viralicidic activity 2-like	46.592	DVDFEGTDEPIFGK(1)K	0.55
Q00325	SLC25A3	218	Phosphate carrier protein, mitocho	46.891	AFYK(1)GVAPLWMR	1
P05141	SLC25A5	23	ADP/ATP translocase 2;ADP/ATP tra	368.47	DFLAGGVAAAISK(1)TAVAPI	0.76
P05141	SLC25A5	96	ADP/ATP translocase 2;ADP/ATP tra	68.893	YK(1)QIFLGGVDK	0.87
P05141	SLC25A5	163	ADP/ATP translocase 2;ADP/ATP tra	40.724	GLGDCLVK(1)IYK	0.95
P05141	SLC25A5	166	ADP/ATP translocase 2;ADP/ATP tra	47.082	IYK(1)SDGIK	0.95
P05141	SLC25A5	245	ADP/ATP translocase 2;ADP/ATP tra	86.313	K(1)GTDIMYTGTLDCWR	1.03
P05141	SLC25A5	105	ADP/ATP translocase 2;ADP/ATP tra	46.462	QIFLGGVDK(1)R	1.06
P05141	SLC25A5	147	ADP/ATP translocase 2;ADP/ATP tra	66.262	LAADVGK(1)AGAER	1.17
P05141	SLC25A5	199	ADP/ATP translocase 2;ADP/ATP tra	51.218	AAYFGIYDTAK(1)GMLPDPK	Tip60 OE only
P05141	SLC25A5;SLC25A31;SL	92	ADP/ATP translocase 2;ADP/ATP tra	97.911	YFPTQALNFAFK(1)DK	0.91
P05141	SLC25A5;SLC25A6	268	ADP/ATP translocase 2;ADP/ATP tra	61.999	DEGGK(1)AFFK	1.02
P05141	SLC25A5;SLC25A6;SLC	272	ADP/ATP translocase 2;ADP/ATP tra	132.88	AFFK(1)GAWSNVLR	0.85
P05141	SLC25A5;SLC25A6;SLC	33	ADP/ATP translocase 2;ADP/ATP tra	61.435	VK(1)LLLQVQHASK	0.99
P12236	SLC25A6	105	ADP/ATP translocase 3;ADP/ATP tra	116.01	QIFLGGVDK(1)HTQFWR	1.09
P12236	SLC25A6	96	ADP/ATP translocase 3;ADP/ATP tra	68.893	YK(1)QIFLGGVDK	Tip60 OE only
P12236	SLC25A6;SLC25A4	199	ADP/ATP translocase 3;ADP/ATP tra	75.045	AAYFGVYDTAK(1)GMLPDPK	0.58
Q9BWU0	SLC4A1AP	76	Kanadaptin	61.65	K(1)PALPVSPAAR	0.24

Q9NWH9	SLTM	1024	SAFB-like transcription modulator	57.59	GSGSGFK(1)PFK	0.2
O00193	SMAP	91	Small acidic protein	82.908	INEELESQYQQSMDSK(1)LSG	Tip60 OE only
P28370	SMARCA1	443	Probable global transcription activa	49.35	DIDVLNSSGK(1)MDK	Tip60 OE only
O60264	SMARCA5	647	SWI/SNF-related matrix-associated a	54.608	IGK(1)DEMLQMIR	Tip60 OE only
Q92922	SMARCC1	65	SWI/SNF complex subunit SMARCC1	71.349	K(1)YVHADAPTNK	Tip60 OE only
Q92925	SMARCD2	514	SWI/SNF-related matrix-associated a	54.549	HIFAK(1)VQQR	Tip60 OE only
Q9UQE7	SMC3	105	Structural maintenance of chromos	97.911	VIGAK(1)K(1)DQYFLDK	1.04
Q9UQE7	SMC3	106	Structural maintenance of chromos	114.19	VIGAK(1)K(1)DQYFLDK	1.27
Q9NTJ3	SMC4	1273	Structural maintenance of chromos	48.44	TYNITK(1)SVAVNPK	0.86
Q6IN85	SMEK1	467	Serine/threonine-protein phosphata	70.412	TLVDPENMLATANK(1)TEK	Tip60 OE only
Q16637	SMN1	209	Survival motor neuron protein	45.081	LGPGK(1)PGLK	0.04
P53814	SMTN	506	Smoothelin	62.14	APPTLLSTSSGGK(1)STITR	Tip60 OE only
Q7KZF4	SND1	515	Staphylococcal nuclease domain-co	49.813	AK(1)QFLPFLQR	Tip60 OE only
O75643	SNRNP200	1557	U5 small nuclear ribonucleoprotein	52.278	K(1)PVIVFVPSR	0.86
Q16560	SNRNP35	147	U11/U12 small nuclear ribonucleop	67.032	LGGGLGGK(1)K	Tip60 OE only
P08621	SNRNP70	130	U1 small nuclear ribonucleoprotein	50.878	EFEVYGPIK(1)R	unquantifiable
P09661	SNRPA1	179	U2 small nuclear ribonucleoprotein	48.741	SK(1)TFNPGAGLPTDK	0.06
P09661	SNRPA1	221	U2 small nuclear ribonucleoprotein	51.092	LK(1)GLLQSGQIPGR	unquantifiable
P08579	SNRPB2	111	U2 small nuclear ribonucleoprotein	62.408	AK(1)TVEQTATTTNK	Tip60 OE only
P62308	SNRPG;SNRPGP15	3	Small nuclear ribonucleoprotein G;I	93.111	SK(1)AHPPELK	unquantifiable
P63162	SNRPN;SNRPB	32	Small nuclear ribonucleoprotein-as	58.37	IFIGTFK(1)AFDK	Tip60 OE only
Q13573	SNW1	441	SNW domain-containing protein 1	186.58	GGK(1)DMAQSIYRPSK	0.02
Q13573	SNW1	193	SNW domain-containing protein 1	103.16	YTPSQQGVAFNSGAK(1)QR	0.03
P04179	SOD2	130	Superoxide dismutase [Mn], mitoch	56.729	DFGSFDK(1)FK	0.86
P18583	SON	2055	Protein SON	69.721	LTDLDK(1)AQLLEIAK	0.53
P08047	SP1;SP3;SP4	639	Transcription factor Sp1;Transcripti	67.993	VYGK(1)TSHLR	Tip60 OE only
Q9NUQ6	SPATS2L	488	SPATS2-like protein	83	GGAK(1)NQEASLGMK	Tip60 OE only
Q96T58	SPEN	1563	Msx2-interacting protein	46.956	IYGK(1)QTSEGANSTTDSIQEI	Tip60 OE only
Q68D10	SPTY2D1	532	Protein SPT2 homolog	42.039	TVSNSVPGRPVSSLGPGQTVS	Tip60 OE only
Q6ZRS2	SRCAP	1205	Helicase SRCAP	51.218	PVANAGGSK(1)PLTFQIQGN	Tip60 OE only
P49458	SRP9	52	Signal recognition particle 9 kDa pr	118.51	VTDDLVCLVYK(1)TDQAQDV	0.86
Q9UQ35	SRRM2	285	Serine/arginine repetitive matrix pr	95.094	SAAAK(1)THTTALAGR	0.16
Q9UQ35	SRRM2	1022	Serine/arginine repetitive matrix pr	131.66	SK(1)DSLVQSCPGSLSLCAGV	Tip60 OE only
Q16629	SRSF7	24	Serine/arginine-rich splicing factor 7	45.915	VYVGNLGTGAGK(1)GELER	0.45

Q16629	SRSF7	70	Serine/arginine-rich splicing factor 7	51.092	GLDGK(1)VICGSR	1.34
Q15532	SS18	13	Protein SSXT	134.84	GK(1)GEITPAAIQK	unquantifiable
O75177	SS18L1	13	Calcium-responsive transactivator	49.472	GK(1)GEVTQQTIQK	unquantifiable
P05455	SSB	354	Lupus La protein	94.407	GK(1)VQFQGK	0.11
P05455	SSB	360	Lupus La protein	88.734	VQFQGK(1)K	0.88
P05455	SSB	287	Lupus La protein	114.5	AK(1)DANNGNLQLR	Tip60 OE only
Q04837	SSBP1	113	Single-stranded DNA-binding protei	60.628	IYLEGK(1)IDYGEYMDK	0.93
Q04837	SSBP1	103	Single-stranded DNA-binding protei	61.999	DVAYQYVK(1)K	1.1
P81877	SSBP2	6	Single-stranded DNA-binding protei	164.48	GK(1)SNSSAVPSDSQAR	Tip60 OE only
Q9BWW4	SSBP3	6	Single-stranded DNA-binding protei	168.3	GK(1)GSAVPSDGQAR	unquantifiable
Q9BWG4	SSBP4	7	Single-stranded DNA-binding protei	53.569	GGK(1)GSAVPSDSQAR	Tip60 OE only
Q8N3U4	STAG2	607	Cohesin subunit SA-2	73.985	LEK(1)HLDALLR	Tip60 OE only
Q8N3U4	STAG2	55	Cohesin subunit SA-2	46.592	GK(0.997)GGNGGGK(0.003	Tip60 OE only
Q9P2P6	STARD9	570	StAR-related lipid transfer protein 9	56.548	LTQGAVITLGK(1)AQK	Tip60 OE only
P31948	STIP1	429	Stress-induced-phosphoprotein 1	55.314	DCEECIQLEPTFIK(1)GYTR	0.79
P31948	STIP1	100	Stress-induced-phosphoprotein 1	46.926	TYEEGLK(1)HEANNPQLK	1.07
P31948	STIP1	246	Stress-induced-phosphoprotein 1	103.22	DFDTALK(1)HYDK	1.21
P16949	STMN1	128	Stathmin	41.029	DK(1)HIEEVR	0.86
Q9P2R7	SUCLA2	88	Succinyl-CoA ligase [ADP-forming] su	89.247	SPDEAYAIAK(1)K	0.7
P53597	SUCLG1	66	Succinyl-CoA ligase [ADP/GDP-formi	51.645	IICQGFTGK(1)QGTFHSQQAL	0.95
Q96I99	SUCLG2	338	Succinyl-CoA ligase [GDP-forming] su	97.271	PANFLDLGGGVK(1)EAQVYC	unquantifiable
Q8IX01	SUGP2	1035	SURP and G-patch domain-containi	42.395	EGHGLGSLGK(1)GIR	0.41
Q8IX01	SUGP2	238	SURP and G-patch domain-containi	53.569	GETQGLLTAK(1)GGVGK	Tip60 OE only
Q8IX01	SUGP2	243	SURP and G-patch domain-containi	52.49	GGVGK(1)LVTLR	Tip60 OE only
P61956	SUMO2	11	Small ubiquitin-related modifier 2	112.15	EGVK(1)TENNDHINLK	Tip60 OE only
Q9Y5B9	SUPT16H	696	FACT complex subunit SPT16	52.79	GDK(1)VDILYNNIK	Tip60 OE only
Q9BSH4	TACO1	132	Translational activator of cytochron	41.283	SK(1)DTYLLYEGR	1.53
O75528	TADA3	97	Transcriptional adapter 3	49.483	DHELGAPPK(0.772)HGK(0.	unquantifiable
O75528	TADA3	109	Transcriptional adapter 3	87.932	LEGK(1)AGHGPGPGPGRPK	unquantifiable
P21675	TAF1	544	Transcription initiation factor TFIID	54.416	ILLGK(1)TGVIK	unquantifiable
Q6P1X5	TAF2	422	Transcription initiation factor TFIID	44.567	TGGVLLHPIFGGGK(1)EK	Tip60 OE only
Q6P1X5	TAF2	1075	Transcription initiation factor TFIID	108.98	PSTPGLSK(1)YR	unquantifiable
Q5VWG9	TAF3	266	Transcription initiation factor TFIID	72.643	PLETK(1)SFTPK	unquantifiable
Q16594	TAF9	5	Transcription initiation factor TFIID	60.518	MESGK(1)TASPK	unquantifiable

Q14241	TCEB3	697	Transcription elongation factor B po	44.309	MAFVNSVAK(1)PPR	unquantifiable
O14776	TCERG1	753	Transcription elongation regulator 1	42.743	ATFSEFAAK(1)HAK	1
Q9UGU0	TCF20	664	Transcription factor 20	67.396	ETSHASLPQPEPPGGGGSK(1	Tip60 OE only
P15923	TCF3	101	Transcription factor E2-alpha	59.598	TFSEGTHFTESHSSLSSSTFLGI	Tip60 OE only
Q13428	TCOF1	519	Treacle protein	71.558	PASTMGMGPLGK(1)GAGPV	0.2
Q13428	TCOF1	146	Treacle protein	63.184	TGNSMPHPATGK(1)TVANLL	0.21
Q13428	TCOF1	811	Treacle protein	86.803	GTISAPGK(1)VVTAAAQAK	0.27
Q13428	TCOF1	155	Treacle protein	196.88	TVANLLSGK(1)SPR	0.68
Q13428	TCOF1	746	Treacle protein	107.34	GSLGQGTAPVLPGK(1)TGPT	0.71
Q13428	TCOF1	803	Treacle protein	134.98	APSAK(1)GTISAPGK	Tip60 OE only
Q13428	TCOF1	432	Treacle protein	98.933	PSGK(1)APQVR	Tip60 OE only
Q13428	TCOF1	1034	Treacle protein	91.961	IAPK(1)ASMAGASSSK	Tip60 OE only
Q13428	TCOF1	313	Treacle protein	84.479	GTPGK(1)GATPAPPGK	Tip60 OE only
Q13428	TCOF1	732	Treacle protein	56.482	AASVPVK(1)GSLGQGTAPVLI	Tip60 OE only
Q13428	TCOF1	1263	Treacle protein	58.674	TGGK(1)EAASGTTPQK	Tip60 OE only
Q13428	TCOF1	892	Treacle protein	47.198	PSGK(1)THQIR	unquantifiable
P17987	TCP1	400	T-complex protein 1 subunit alpha	103.55	SLHDALCVVK(1)R	1.04
P17987	TCP1	494	T-complex protein 1 subunit alpha	66.435	WIGLDLSNGK(1)PR	Tip60 OE only
Q9NZ01	TECR	22	Very-long-chain enoyl-CoA reductas	81.431	LCFLDK(1)VEPHATIAEIK	0.83
Q9UGI8	TES	298	Testin	60.91	HYCDSEK(1)PR	1.04
Q9UBB9	TFIP11	170	Tuftelin-interacting protein 11	90.614	GLGK(1)NAQGIINPIEAK	Tip60 OE only
Q9GZN2	TGIF2	99	Homeobox protein TGIF2	73.985	GGK(1)ASDVALPR	Tip60 OE only
Q8IYQ7	THNSL1	281	Threonine synthase-like 1	82.908	LSCGEWK(1)SLVGATYVER	unquantifiable
Q9Y2W1	THRAP3	455	Thyroid hormone receptor-associat	59.597	FMSK(1)VIGANK	0.07
Q9Y2W1	THRAP3	811	Thyroid hormone receptor-associat	59.426	EESTTGFDK(1)SR	0.89
Q9Y2W1	THRAP3	551	Thyroid hormone receptor-associat	146.11	LGAK(1)GDFPTGK(1)SSFSIT	Tip60 OE only
Q9Y2W1	THRAP3	709	Thyroid hormone receptor-associat	57.159	AEGK(0.991)YK(0.009)DDP	Tip60 OE only
Q9Y2W1	THRAP3	346	Thyroid hormone receptor-associat	61.435	EESAASGGAAYTK(1)R	Tip60 OE only
Q9Y2W1	THRAP3	558	Thyroid hormone receptor-associat	58.487	LGAK(1)GDFPTGK(1)SSFSIT	Tip60 OE only
O43615	TIMM44	138	Mitochondrial import inner membr	74.162	ESLHEVSK(1)SDLGR	1.06
P04183	TK1	16	Thymidine kinase, cytosolic	64.199	SCINLPTVLPGSPSK(1)TR	Tip60 OE only
P29401	TKT	260	Transketolase	69.423	ESWHGK(1)PLPK	0.45
P29401	TKT	232	Transketolase	96.64	AFGQAK(1)HQPTAIIAK	0.97
P29401	TKT	6	Transketolase	48.091	MESYHK(1)PDQQK	1.08

Q04726	TLE3	316	Transducin-like enhancer protein 3	42.041	SSTPGLK(1)SNTPTPR	Tip60 OE only
P49755	TMED10	133	Transmembrane emp24 domain-cor	41.283	HGVEAK(1)NYEEIAK	unquantifiable
Q8N2U0	TMEM256	43	Transmembrane protein 256	65.676	ELFDK(1)ANK	unquantifiable
P42167	TMPO	239	Lamina-associated polypeptide 2, is	44.615	VEHNQSYSQAGITETEWTSGS	Tip60 OE only
Q92973	TNPO2;TNPO1	81	Transportin-2;Transportin-1	72.928	SLSGLILK(1)NNVK	1.31
P11387	TOP1	148	DNA topoisomerase 1	66.435	DEDDADYK(1)PK	0.32
P11388	TOP2A	1240	DNA topoisomerase 2-alpha	58.752	IK(1)NENTEGSPQEDGVELEG	Tip60 OE only
P11388	TOP2A;TOP2B	971	DNA topoisomerase 2-alpha;DNA to	40.002	FVVK(1)MTEEK	1.15
Q02880	TOP2B	1214	DNA topoisomerase 2-beta	42.395	EDVLAGMSGK(1)AIK	0.6
Q92547	TOPBP1	834	DNA topoisomerase 2-binding prote	76.827	LFK(1)PSFDVK	unquantifiable
P04637	TP53	120	Cellular tumor antigen p53	137.89	LGFLHSGTAK(1)SVTCTYSPA	0.05
P04637	TP53	381	Cellular tumor antigen p53	75.109	HK(1)K(1)LMFK	0.37
P04637	TP53	382	Cellular tumor antigen p53	75.109	HK(1)K(1)LMFK	0.37
P60174	TPI1	212	Triosephosphate isomerase	73.655	VVLAYEPVWAIGTGK(1)TATF	0.21
P60174	TPI1	168	Triosephosphate isomerase	61.222	VAHALAEGLGVIACIGEK(1)LI	0.8
P60174	TPI1	43	Triosephosphate isomerase	43.246	K(1)FFVGGNWK	0.86
O94811	TPPP	187	Tubulin polymerization-promoting	58.167	GK(1)GK(1)AGR	unquantifiable
O94811	TPPP	189	Tubulin polymerization-promoting	58.167	GK(1)GK(1)AGR	unquantifiable
P12270	TPR	713	Nucleoprotein TPR	80.469	SQNTK(1)ISTQLDFASK	0.89
P12270	TPR	723	Nucleoprotein TPR	60.434	ISTQLDFASK(1)R	1.08
P12270	TPR	1680	Nucleoprotein TPR	47.96	GIASTSDPPTANIK(0.001)PT	Tip60 OE only
P12270	TPR	755	Nucleoprotein TPR	114.43	LTATTQK(1)QEQIINTMTQDL	Tip60 OE only
Q12931	TRAP1	324	Heat shock protein 75 kDa, mitocho	107.15	YVAQAHDK(1)PR	1.02
Q13263	TRIM28	213	Transcription intermediary factor 1-	82.849	TVYCNVHK(1)HEPLVLFCESC	1.05
Q13263	TRIM28	779	Transcription intermediary factor 1-	46.592	LTEDK(1)ADVQSIIGLQR	1.17
Q13263	TRIM28	366	Transcription intermediary factor 1-	98.997	K(1)LIYFQLHR	1.28
Q13263	TRIM28	340	Transcription intermediary factor 1-	157.66	IQK(1)HQEHILR	1.31
Q13263	TRIM28	266	Transcription intermediary factor 1-	131.82	LGDK(1)HATLQK	1.56
Q9UPN9	TRIM33	763	E3 ubiquitin-protein ligase TRIM33	44.318	TAEK(1)TSLSFK	0.85
Q9UPN9	TRIM33	953	E3 ubiquitin-protein ligase TRIM33	189.57	GK(1)TAQGLSPVDQR	0.86
Q9NXH9	TRMT1	361	tRNA (guanine(26)-N(2))-dimethyltra	52.278	LGK(1)ASGVPSGR	Tip60 OE only
P10155	TROVE2	170	60 kDa SS-A/Ro ribonucleoprotein	56.359	GGMALALAVTK(1)YK	Tip60 OE only
Q15631	TSN	187	Translin	78.934	LLNLK(1)NDSLR	1.01
P68363	TUBA1B;TUBA1A;TUBA	60	Tubulin alpha-1B chain;Tubulin alph	67.864	TIGGGDDSFNTFFSETGAGK(	0.63

P68363	TUBA1B;TUBA1A;TUBA	336	Tubulin alpha-1B chain;Tubulin alpł	75.695	DVNAAIATIK(1)TK	1.25
P68363	TUBA1B;TUBA4A;TUBA	96	Tubulin alpha-1B chain;Tubulin alpł	48.315	QLFHPEQLITGK(1)EDAANN	0.92
P68363	TUBA1B;TUBA4A;TUBA	311	Tubulin alpha-1B chain;Tubulin alpł	49.448	HGK(1)YMACCLLYR	1.3
P68363	TUBAL3;TUBA1B;TUBA	394	Tubulin alpha chain-like 3;Tubulin a	171.92	LDHK(1)FDLMYAK	0.81
P68363	TUBAL3;TUBA1B;TUBA	401	Tubulin alpha chain-like 3;Tubulin a	65.038	FDLMYAK(1)R	1.22
P07437	TUBB	58	Tubulin beta chain	64.55	ISVYYNEATGGK(1)YVPR	0.59
P68371	TUBB4B	58	Tubulin beta-4B chain	86.313	INVYYNEATGGK(1)YVPR	0.79
P49411	TUFM	347	Elongation factor Tu, mitochondrial	88.819	PGSIK(1)PHQK	0.61
P49411	TUFM	234	Elongation factor Tu, mitochondrial	85.355	DPELGLK(1)SVQK	Tip60 OE only
P10599	TXN	39	Thioredoxin	94.297	MIK(1)PFFHSLSEK	0.99
P10599	TXN	94	Thioredoxin	99.973	VGEFSGANK(1)EK	1
P10599	TXN	8	Thioredoxin	146	QIESK(1)TAFQEALDAAGDK	1.02
Q9BRA2	TXNDC17	40	Thioredoxin domain-containing prc	109.07	DAGGK(1)SWCPDCVQAEPV	0.32
P0DN76	U2AF1	175	Splicing factor U2AF 35 kDa subunit	65.472	GGFCNFMHLK(1)PISR	1.1
P0DN76	U2AF1	39	Splicing factor U2AF 35 kDa subunit	40.085	LHNK(1)PTFSQTIALLNIYR	1.28
P0DN76	U2AF1;U2AF1L4	15	Splicing factor U2AF 35 kDa subunit	96.331	DK(1)VNCSFYFK	1.27
P26368	U2AF2	70	Splicing factor U2AF 65 kDa subunit	123.79	GAK(1)EEHGGLIR	0.02
O15042	U2SURP	156	U2 snRNP-associated SURP motif-co	73.665	GK(1)IYKPSSR	Tip60 OE only
P22314	UBA1	671	Ubiquitin-like modifier-activating e	48.112	QPAENVNQYLTDPK(1)FVER	1.15
Q8TBC4	UBA3	409	NEDD8-activating enzyme E1 catalyt	58.676	SPAITATLEGK(1)NR	0.74
P49459	UBE2A	66	Ubiquitin-conjugating enzyme E2 A	89.959	LTIEFTEEYPNK(1)PPTVR	Tip60 OE only
P61088	UBE2N	92	Ubiquitin-conjugating enzyme E2 N	53.327	ICLDILK(1)DK	0.63
Q9NPD8	UBE2T	91	Ubiquitin-conjugating enzyme E2 T	57.598	ICLDVLK(1)LPPK	Tip60 OE only
Q13404	UBE2V1	10	Ubiquitin-conjugating enzyme E2 va	41.54	AATTGSGVK(1)VPR	Tip60 OE only
P17480	UBTF	279	Nucleolar transcription factor 1	114.4	HPELNISEEGITK(1)STLTK	0.18
Q9Y5K5	UCHL5	158	Ubiquitin carboxyl-terminal hydrol	190.11	TSAK(1)EEDAFHFVSYVPVNG	0.74
Q9NYU1	UGGT2	5	UDP-glucose:glycoprotein glucosylt	69.628	APAK(1)ATNVVR	Tip60 OE only
Q96PU4	UHRF2	12	E3 ubiquitin-protein ligase UHRF2	75.045	TIDGSK(1)TCTIEDVSR	0.62
P22695	UQCRC2	92	Cytochrome b-c1 complex subunit 2	66.262	LTSSLTTK(1)GASSFK	0.97
O60287	URB1	1142	Nucleolar pre-ribosomal-associated	41.482	EVTLALLSLPETHLVTQQPTK(	Tip60 OE only
Q9Y5T5	USP16	9	Ubiquitin carboxyl-terminal hydrol	41.052	GK(1)TVPIDDSSETLEPVCR	unquantifiable
Q9UPT9	USP22	59	Ubiquitin carboxyl-terminal hydrol	292.79	AK(1)SCICHVCGVHLNR	0.11
Q9UPT9	USP22	159	Ubiquitin carboxyl-terminal hydrol	49.097	ELELLK(1)HNPK	unquantifiable
Q9Y6I4	USP3	354	Ubiquitin carboxyl-terminal hydrol	127.87	SK(1)NQENGPVCSLR	Tip60 OE only

Q93009	USP7	869	Ubiquitin carboxyl-terminal hydrol	53.166	DLLQFFK(1)PR	Tip60 OE only
O75691	UTP20	1960	Small subunit processome compone	49.35	SK(1)SYDSYEILGK	0.11
O75691	UTP20	1811	Small subunit processome compone	84.17	SK(1)VVNDEEVVR	Tip60 OE only
Q9NQZ2	UTP3	144	Something about silencing protein :	42.743	LYYDTDYGSK(1)SR	0.02
O95292	VAPB	3	Vesicle-associated membrane protei	74.326	AK(1)VEQVLSLEPQHELK	0.04
P55072	VCP	231	Transitional endoplasmic reticulum	41.644	HPALFK(0.994)AIGVK(0.00	0.71
P21796	VDAC1	252	Voltage-dependent anion-selective c	41.092	VNNSSLIGLGYTQTLK(1)PGII	0.67
P21796	VDAC1	224	Voltage-dependent anion-selective c	68.809	FGIAAK(1)YQIDPDACFSAK	0.79
P21796	VDAC1	12	Voltage-dependent anion-selective c	47.288	AVPPTYADLGK(1)SAR	1.15
P45880	VDAC2	74	Voltage-dependent anion-selective c	112.13	YK(1)WCEYGLTFTEK	0.84
P45880	VDAC2	277	Voltage-dependent anion-selective c	51.211	LTLSALVDGK(1)SINAGGHK	1.14
P45880	VDAC2	45	Voltage-dependent anion-selective c	63.709	TK(1)SCSGVEFSTSGSSNTDT(	Tip60 OE only
Q9Y277	VDAC3	63	Voltage-dependent anion-selective c	73.781	YK(1)VCNYGLTFTQK	0.89
Q9Y277	VDAC3	12	Voltage-dependent anion-selective c	80.979	CNTPTYCDLGK(1)AAK	1.1
Q9Y277	VDAC3	28	Voltage-dependent anion-selective c	67.726	GYGFGMVK(1)IDLK	2.09
Q9Y277	VDAC3	20	Voltage-dependent anion-selective c	70.1	DVFNK(1)GYGFGMVK	Tip60 OE only
P08670	VIM	139	Vimentin	56.783	ILLAELEQLK(1)GQGK	0.84
P08670	VIM	445	Vimentin	53.683	TLLIK(1)TVETR	1.14
P08670	VIM	120	Vimentin	67.993	FANYIDK(1)VR	1.18
Q9Y2W2	WBP11	13	WW domain-binding protein 11	42.001	SGK(1)FMNPTDQAR	0.81
O43709	WBSCR22	134	Probable 18S rRNA (guanine-N(7))-m	65.246	K(1)SENPAK(1)R	unquantifiable
O43709	WBSCR22	140	Probable 18S rRNA (guanine-N(7))-m	65.246	K(1)SENPAK(1)R	unquantifiable
O75717	WDHD1	899	WD repeat and HMG-box DNA-bindi	90.367	STNSSDVSAK(1)SGAVTFSSQ	Tip60 OE only
Q8NEZ3	WDR19	588	WD repeat-containing protein 19	69.01	VYTYVFHK(1)DTIQGAK	unquantifiable
Q64LD2	WDR25	195	WD repeat-containing protein 25	59.542	GK(1)DVEPQGPPAGR	Tip60 OE only
Q9UNX4	WDR3	5	WD repeat-containing protein 3	81.297	GLTK(1)QYLR	0.04
Q8TAF3	WDR48	578	WD repeat-containing protein 48	64.55	IPFYLQPHASSGAK(1)TLK	0.57
P61964	WDR5	112	WD repeat-containing protein 5	41.283	TLK(1)IWDVSSGK	0.64
Q8IWA0	WDR75	466	WD repeat-containing protein 75	62.042	SEQPTLVTASK(1)DGYFK	0.67
Q9BZ95	WHSC1L1	790	Histone-lysine N-methyltransferase I	102.52	FPTAIFESK(1)GFR	unquantifiable
O95785	WIZ	1322	Protein Wiz	72.643	PGK(1)PGAGPAQVPR	unquantifiable
O14980	XPO1	693	Exportin-1	73.327	QLGSILK(1)TNVR	Tip60 OE only
Q9HAV4	XPO5	1160	Exportin-5	58.676	LIAGCIGK(1)PLGEQFR	1.07
Q9HAV4	XPO5	41	Exportin-5	40.002	FCEEFK(1)EK	1.15

P18887	XRCC1	298	DNA repair protein XRCC1	43.282	AQGAVTGK(1)PR	0.77
P13010	XRCC5	534	X-ray repair cross-complementing p	43.512	IK(1)TLFPLIEAK	0.96
P13010	XRCC5	338	X-ray repair cross-complementing p	57.348	SEGK(1)CFSVLGFCK	1.08
P13010	XRCC5	265	X-ray repair cross-complementing p	48.284	IAAYK(1)SILQER	1.21
P12956	XRCC6	605	X-ray repair cross-complementing p	85.845	KQELLEALTK(1)HFQD	1.29
P54577	YARS	427	Tyrosine--tRNA ligase, cytoplasmic;T	58.676	LVVVLCNLK(1)PQK	0.97
P54577	YARS	146	Tyrosine--tRNA ligase, cytoplasmic;T	87.777	LSSVVTQHDSK(1)K	1.12
Q9ULM3	YEATS2	773	YEATS domain-containing protein 2	65.347	GK(1)LLLIPQGAILR	unquantifiable
O95070	YIF1A	13	Protein YIF1A	52.693	AYHSGYGAHGSK(1)HR	Tip60 OE only
P49750	YLPM1	270	YLP motif-containing protein 1	144.1	TTVQQEPLESGAK(1)NK	0.04
P63104	YWHAB;YWHAG;YWHA	49	14-3-3 protein beta/alpha;14-3-3 pr	73.067	NLLSVAYK(1)NVVGAR	0.96
P62258	YWHAE	106	14-3-3 protein epsilon	133.1	LICCDILDVLDK(1)HLIPAANT	0.74
P62258	YWHAE	118	14-3-3 protein epsilon	53.569	HLIPAANTGESK(1)VFYYK	0.91
P62258	YWHAE	12	14-3-3 protein epsilon	54.7	EDLVYQAK(1)LAEQAER	1.17
P62258	YWHAE	50	14-3-3 protein epsilon	107.24	NLLSVAYK(1)NVIGAR	1.41
P27348	YWHAQ	49	14-3-3 protein theta	87.298	NLLSVAYK(1)NVVGGR	1.17
P63104	YWHAQ;SFN;YWHAB;Y	120	14-3-3 protein theta;14-3-3 protein	60.961	VFYLK(1)MK	0.98
P63104	YWHAZ	9	14-3-3 protein zeta/delta	71.451	MDKNELVQK(1)AK	0.96
Q9Y2K1	ZBTB1	3	Zinc finger and BTB domain-contain	43.991	AK(1)PSHSSYVLQQLNNQR	Tip60 OE only
Q9P1Z0	ZBTB4	892	Zinc finger and BTB domain-contain	81.494	GK(1)SGSEGPVGAGEGDR	Tip60 OE only
Q8NCP5	ZBTB44	4	Zinc finger and BTB domain-contain	77.39	GVK(1)TFTHSSSSHSQEMLGI	Tip60 OE only
O75152	ZC3H11A	387	Zinc finger CCCH domain-containing	92.925	TEGPSK(1)TDDSTSGAR	Tip60 OE only
Q6PJT7	ZC3H14	198	Zinc finger CCCH domain-containing	65.5	K(1)PTVTLTYGSSR	Tip60 OE only
Q6PJT7	ZC3H14	555	Zinc finger CCCH domain-containing	59.542	PVNQTAASNK(1)GLR	unquantifiable
Q86VM9	ZC3H18	622	Zinc finger CCCH domain-containing	71.349	TK(1)GEPAPPPGK	0.06
Q86VM9	ZC3H18	789	Zinc finger CCCH domain-containing	125.35	SAK(0.022)PPAGGK(0.978)	Tip60 OE only
Q9UPT8	ZC3H4	1159	Zinc finger CCCH domain-containing	55.841	TPNAGGK(1)ATEPAADTGAC	Tip60 OE only
Q9HCK1	ZDBF2	1666	DBF4-type zinc finger-containing pro	96.89	GK(1)FNLEDTSHR	Tip60 OE only
Q9HCK1	ZDBF2	1701	DBF4-type zinc finger-containing pro	42.789	GK(1)SCQSSASAVDFGASSK	Tip60 OE only
Q96JP5	ZFP91	236	E3 ubiquitin-protein ligase ZFP91	80.239	DETYK(1)PHLER	Tip60 OE only
Q96KR1	ZFR	509	Zinc finger RNA-binding protein	50.354	INFVGGNK(1)LQSTGNK	0.78
Q96NC0	ZMAT2	8	Zinc finger matrin-type protein 2	40.589	ASGSGTK(1)NLDFR	unquantifiable
Q9ULU4	ZMYND8	286	Protein kinase C-binding protein 1	49.813	LK(1)GFPFWPAK	unquantifiable
O43670	ZNF207	36	BUB3-interacting and GLEBS motif-c	54.549	HFK(1)CHICHK	1.19

Q8ND82	ZNF280C	180	Zinc finger protein 280C	47.559	HPSTSK(1)VNSVTPK	Tip60 OE only
Q8ND82	ZNF280C	113	Zinc finger protein 280C	42.454	FHLVSK(0.999)SSQSSVTVEN	Tip60 OE only
A6NFI3	ZNF316	569	Zinc finger protein 316	58.32	EEAAVAAPTPSGK(1)VDPAPI	Tip60 OE only
Q5VUA4	ZNF318	1578	Zinc finger protein 318	97.86	DLYDIFYSSGGK(1)GAPETK	Tip60 OE only
P13682	ZNF35	202	Zinc finger protein 35	102.53	SGGK(1)YSLNSGAVK	Tip60 OE only
Q96F45	ZNF503	265	Zinc finger protein 503	53.865	DTDVGGGGK(1)GTGGASAE	unquantifiable
Q96KM6	ZNF512B	17	Zinc finger protein 512B	42.395	LPGSSK(1)SGPGK	unquantifiable
Q96KM6	ZNF512B	304	Zinc finger protein 512B	44.309	PIVVSK(1)PVTVSR	unquantifiable
Q96KM6	ZNF512B	332	Zinc finger protein 512B	41.502	SENK(1)APR	unquantifiable
Q14966	ZNF638	794	Zinc finger protein 638	41.54	AVEIVTSTSAAK(1)TGQAK	Tip60 OE only
Q9H582	ZNF644	727	Zinc finger protein 644	104.2	YFHQAAK(1)EK	Tip60 OE only
Q8N1G0	ZNF687	951	Zinc finger protein 687	128.52	RELGSK(1)GLK	Tip60 OE only
Q9H7S9	ZNF703	141	Zinc finger protein 703	65.172	SAPGAASAAAALK(1)QLGDS	Tip60 OE only
Q9H7S9	ZNF703	189	Zinc finger protein 703	86.378	DSGSSSVSSTSSSSSSSPGDK(	Tip60 OE only
Q9Y5V0	ZNF706	34	Zinc finger protein 706	59.57	AAAK(1)AALIYTCTVCR	unquantifiable
Q6ZMW2	ZNF782	292	Zinc finger protein 782	41.54	TLTGGK(1)SFSQK	unquantifiable
O95218	ZRANB2	137	Zinc finger Ran-binding domain-con	67.334	GK(1)AVGPASILK	0.16
O95218	ZRANB2	54	Zinc finger Ran-binding domain-con	40.589	AGGTEIGK(1)TLAEK	0.66
P0DP25		22		86.467	EAFSLFDK(1)DGDGTITTK	1.18
P0DP25		95		65.395	VFDK(1)DGNGYISAAELR	Tip60 OE only
