# Supplementary figures and images for: Quantitative Proteomics Reveals the Role of Lysine 2-Hydroxyisobutyrylation Pathway Mediated by Tip60
Source: Oxid Med Cell Longev. 2022 Feb 8;2022:4571319. doi: 10.1155/2022/4571319 (PMC8847014; doi:10.1155/2022/4571319)

**Figure S1.** The representative MS2 spectra of the Tip60-targeted Khib peptides

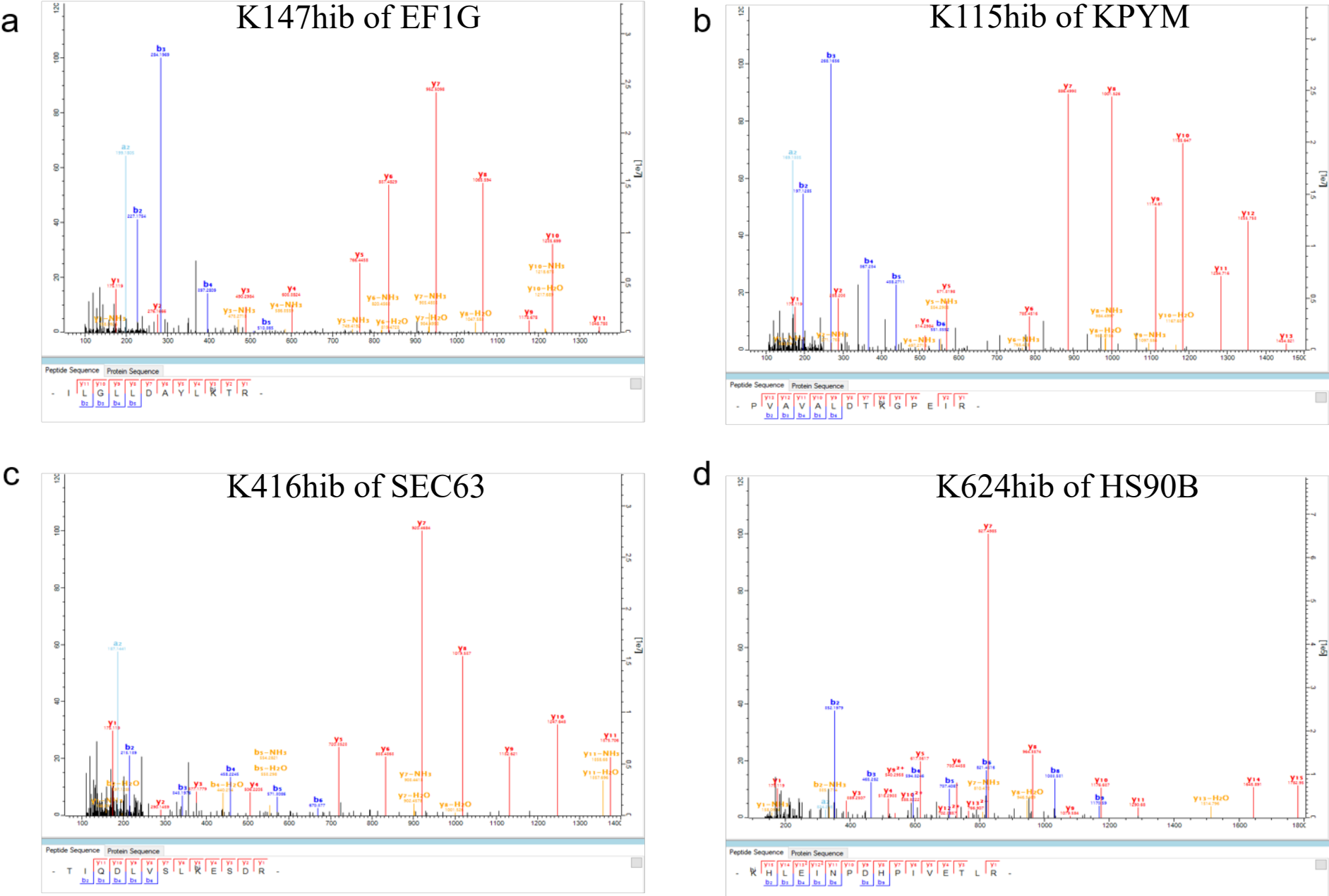

Supplement: Supplementary Materials — Tables S1 and S2 are the complete lists of identified Khib and Kac sites in WT and Tip60 OE cells. Supplementary Materials Figure S1 is the representative MS2 spectra of the Tip60-targeted Khib peptides. Table S1: complete list of identified Khib sites in WT and Tip60 OE cells. Table S2: complete list of identified Kac sites in WT and Tip60 OE cells. Figure S1: the representative MS2 spectra of the Tip60-targeted Khib peptides, including K147hib of EF1G, K115hib of KPYM, K416hib of SEC63, and K624hib of HS90B. [file 4571319.f1.zip › SI_Figure S1.pdf]
